# Supplementary material for: Diversity-oriented functionalization of 2-pyridones and uracils
Source: Nat Commun. 2021 May 20;12:2988. doi: 10.1038/s41467-021-23058-3 (PMC8137914; doi:10.1038/s41467-021-23058-3)
Supplement: Supplementary file 1 — Supplementary Information [file 41467_2021_23058_MOESM1_ESM.pdf]

Supporting Information

Diversity-Oriented Functionalization of 2-Pyridones and Uracils

Yong Shang[1,#], Chenggui Wu[2,#], Qianwen Gao[1], Chang Liu[1], Lisha Li[1], Xinping Zhang[1], Hong-Gang Cheng[1], Shanshan Liu[1] and Qianghui Zhou[1,★]

[1]Sauvage Center for Molecular Sciences, Engineering Research Center of Organosilicon Compounds & Materials (Ministry of Education), College of Chemistry and Molecular Sciences, and The Institute for Advanced Studies, Wuhan, 430072;

[2]Key Laboratory of Xin'an Medicine, Ministry of Education, Anhui University of Chinese Medicine, Hefei, Anhui 230038;

[#]These authors contributed equally to this work.

[★]To whom correspondence should be addressed. Email: qhzhou@whu.edu.cn

Content

1. Supplementary Figures 1-111 2

2. Supplementary Tables 1-8 113

3. Supplementary Methods 117

4. Supplementary Notes 1-18 118

5. Supplementary References 169

1. Supplementary Figures

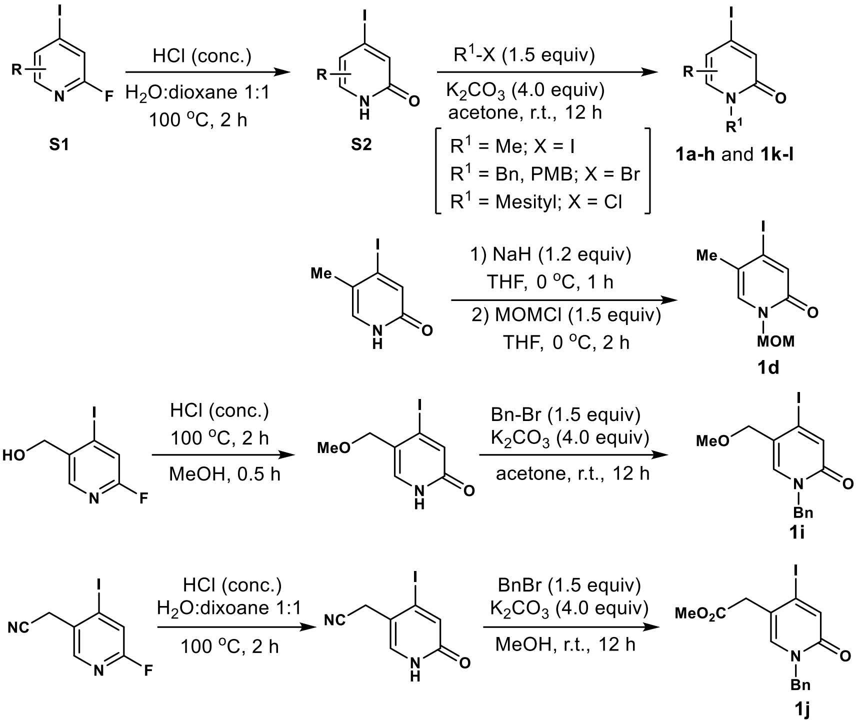

Supplementary Figure 1. Preparation of substrates

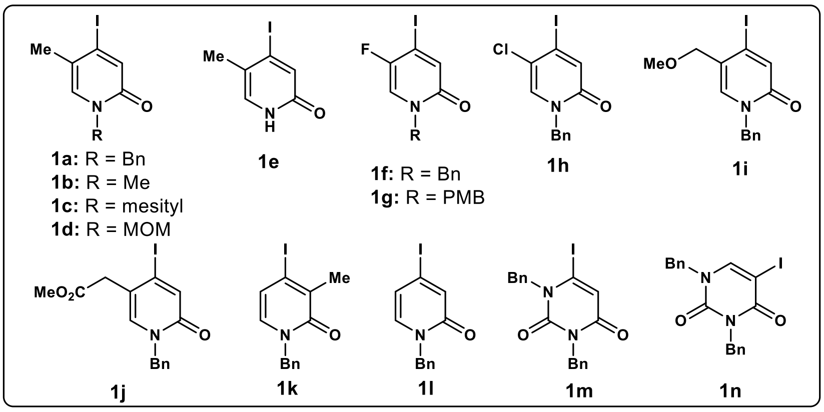

Supplementary Figure 2. Substrates involved in this work

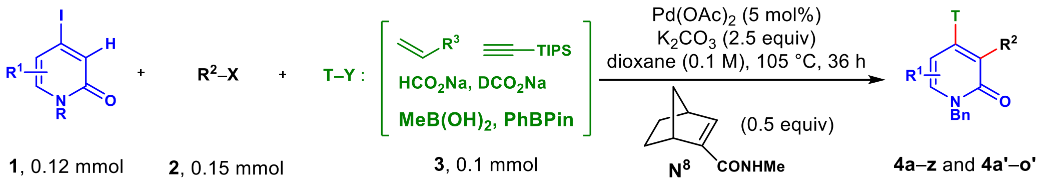

Supplementary Figure 3. *Ortho*-alkylating of 2-pyridones

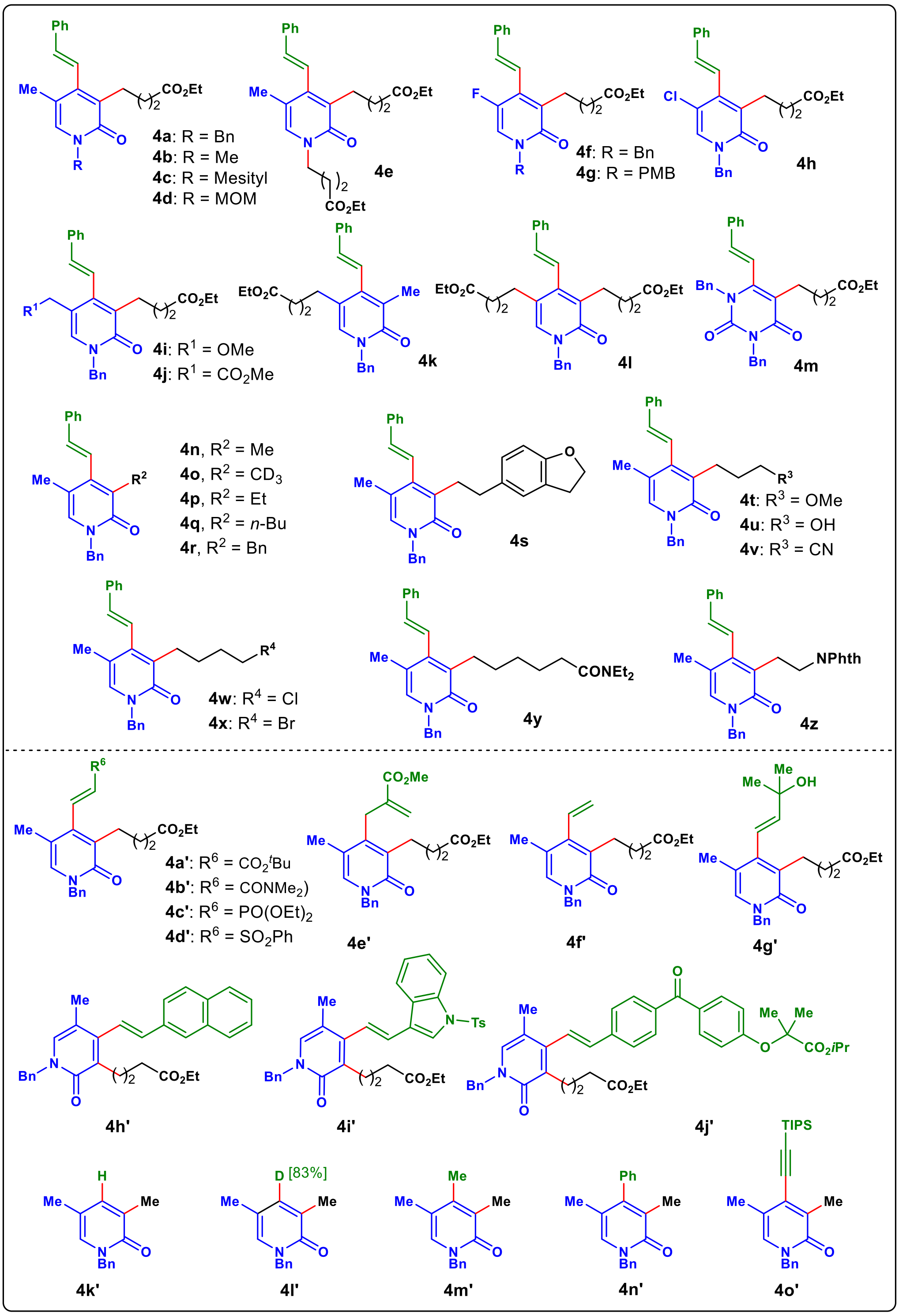

Supplementary Figure 4. Products archive of *ortho*-alkylation

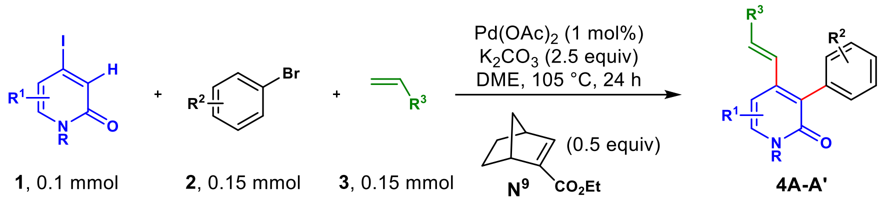

Supplementary Figure 5. *Ortho*-arylation of 2-pyridones

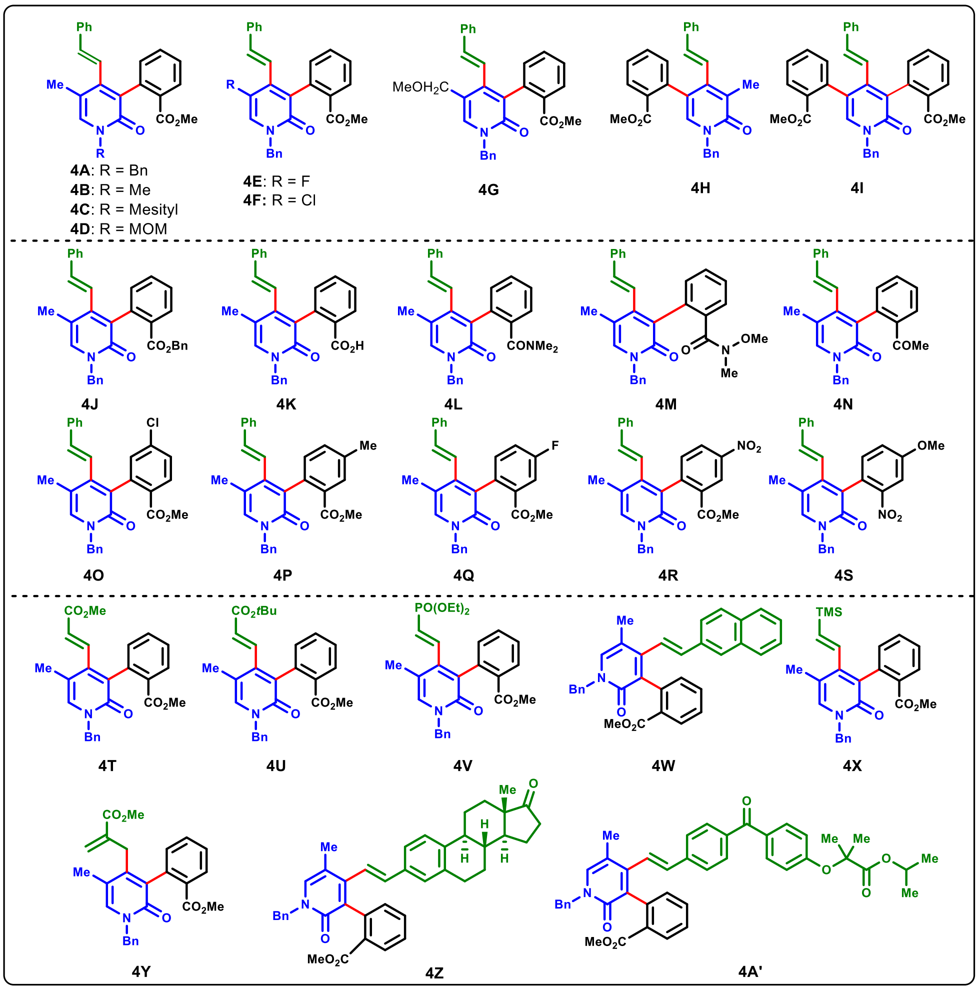

Supplementary Figure 6. Products archive of *ortho*-arylation.

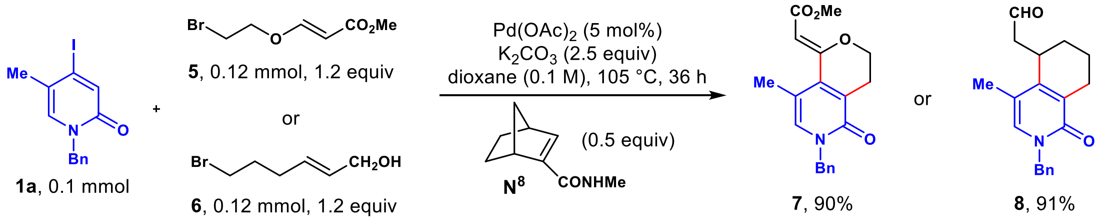

Supplementary Figure 7. Two-component annulation

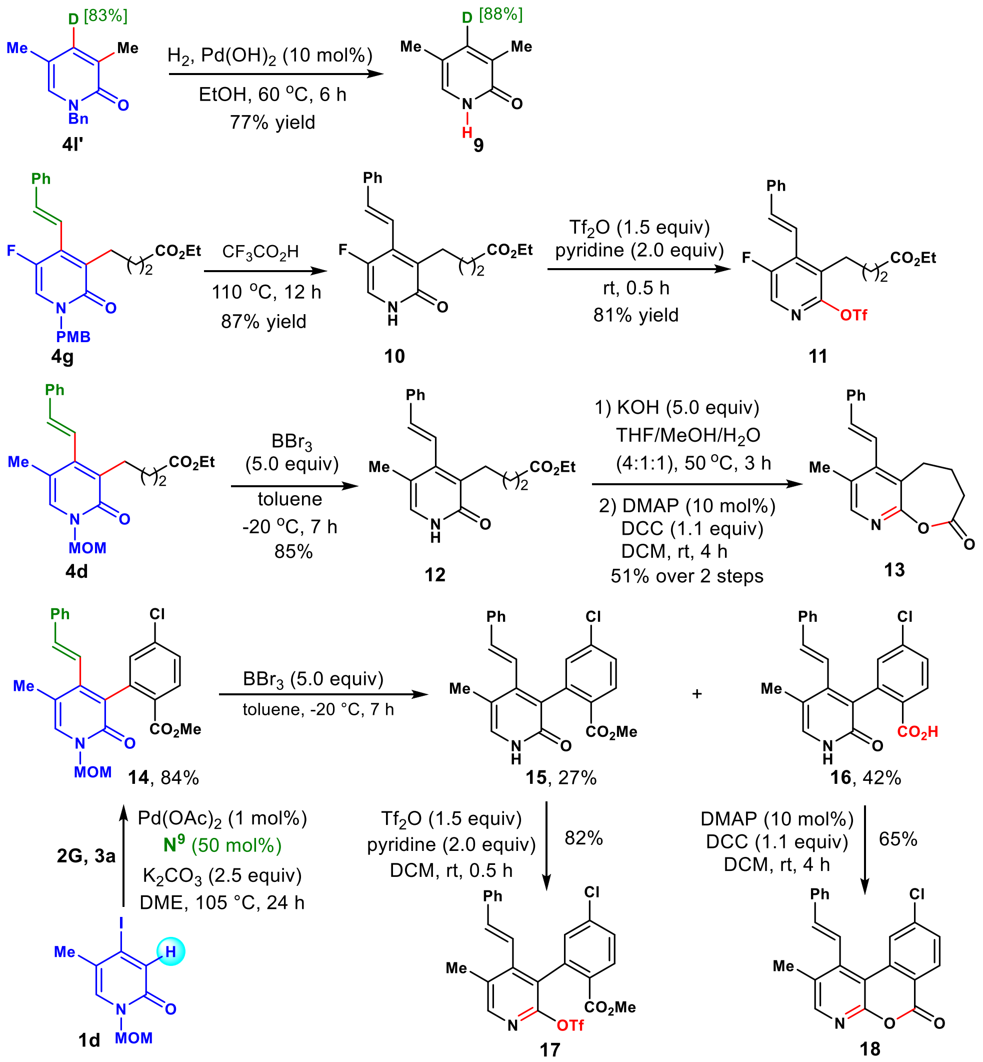

Supplementary Figure 8. *N*-deprotection and follow-up transformations of the obtained 2-pyridones

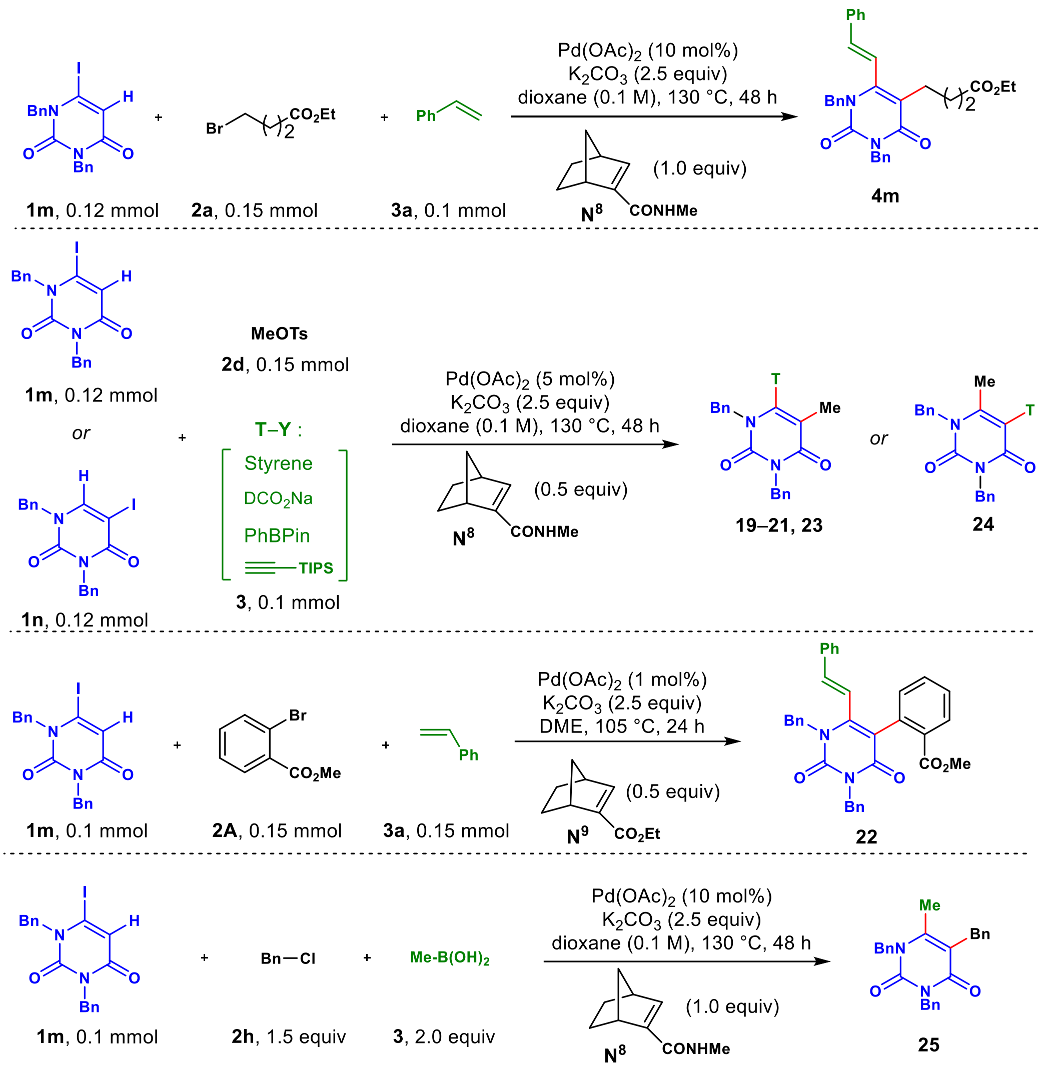

Supplementary Figure 9. Diversity-oriented functionalization of uracils

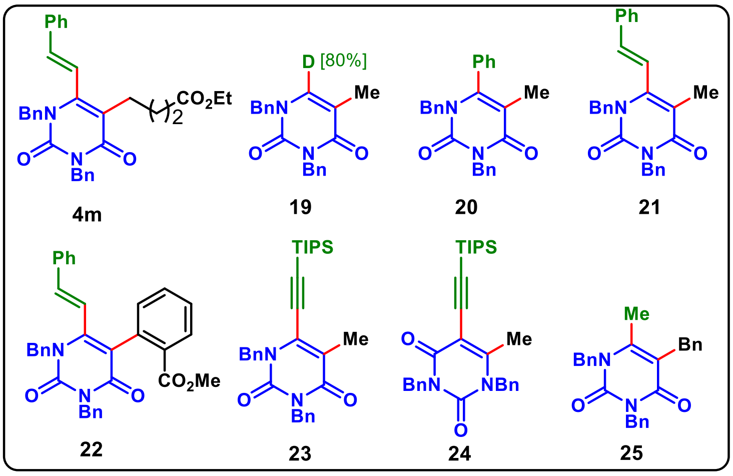

Supplementary Figure 10. Functionalization products of uracils

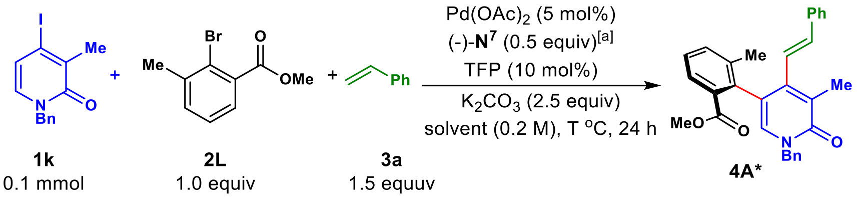

1k 0.1 mmol | **2L** 1.0 equiv | **3a** 1.5 equuv | **4A***

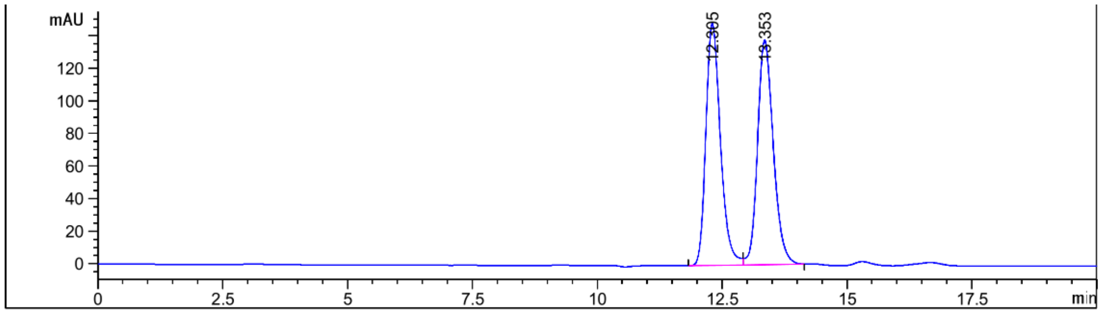

Peak #	RetTime [min]	Type	Width [min]	Area [mAU*s]	Height [mAU]	Area %
1	12.305	BV	0.3125	3011.61328	148.57301	49.9114
2	13.353	VB	0.3376	3022.30737	137.94293	50.0886

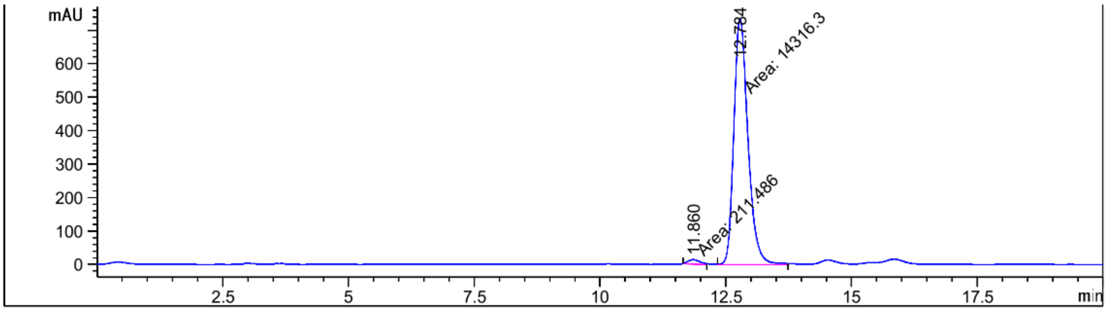

Peak #	RetTime [min]	Type	Width [min]	Area [mAU*s]	Height [mAU]	Area %
1	11.860	MM	0.2753	211.48581	12.80482	1.4557
2	12.784	MM	0.3244	1.43163e4	735.43585	98.5443

Supplementary Figure 11. Asymmetric study and HPLC of **4A***

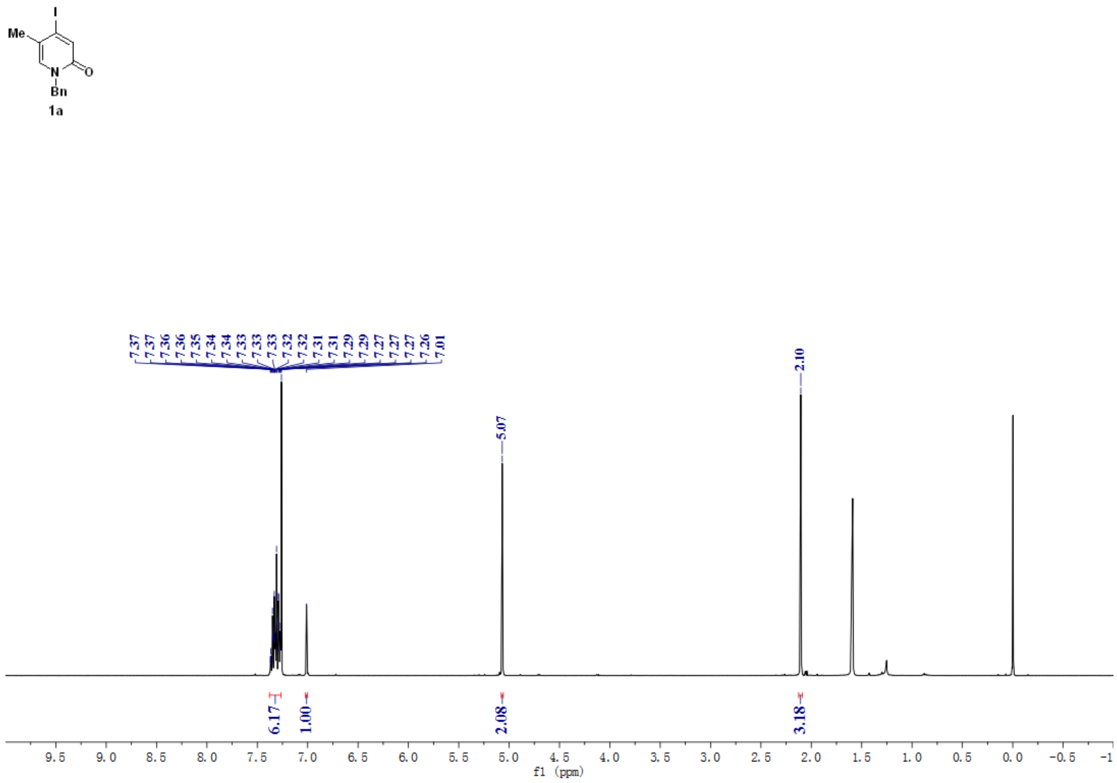

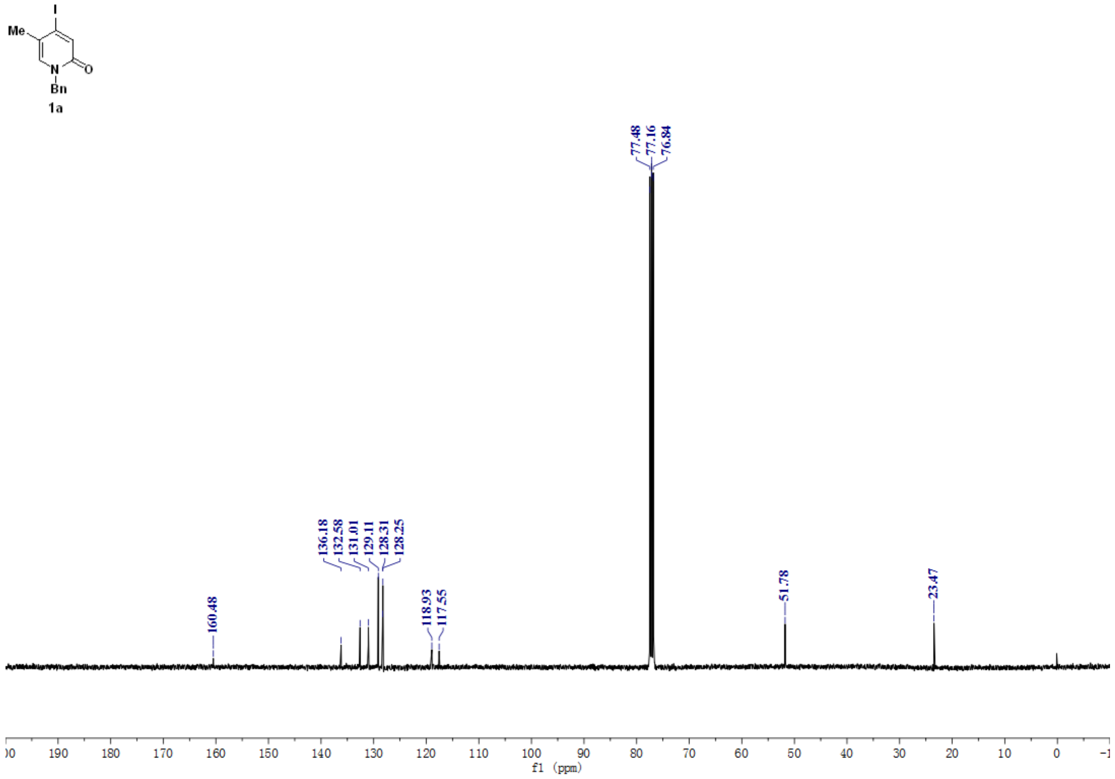

Supplementary Figure 12. NMR of **1a**

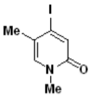

1b

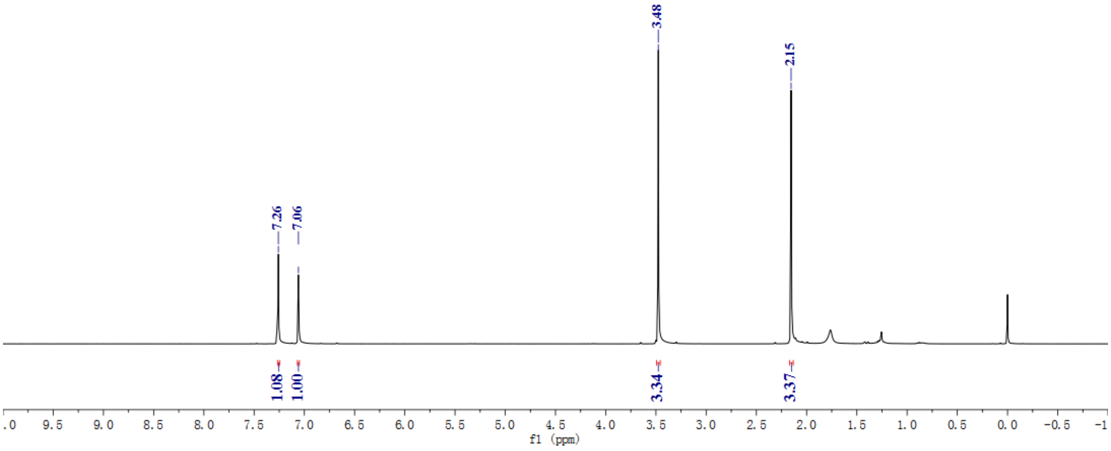

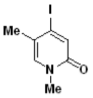

1b

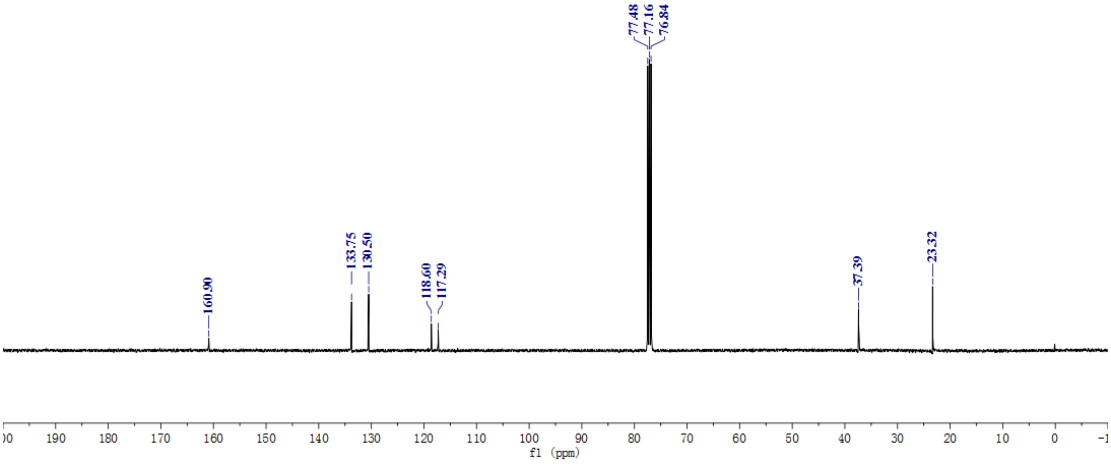

Supplementary Figure 13. NMR of **1b**

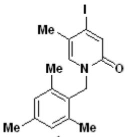

1c

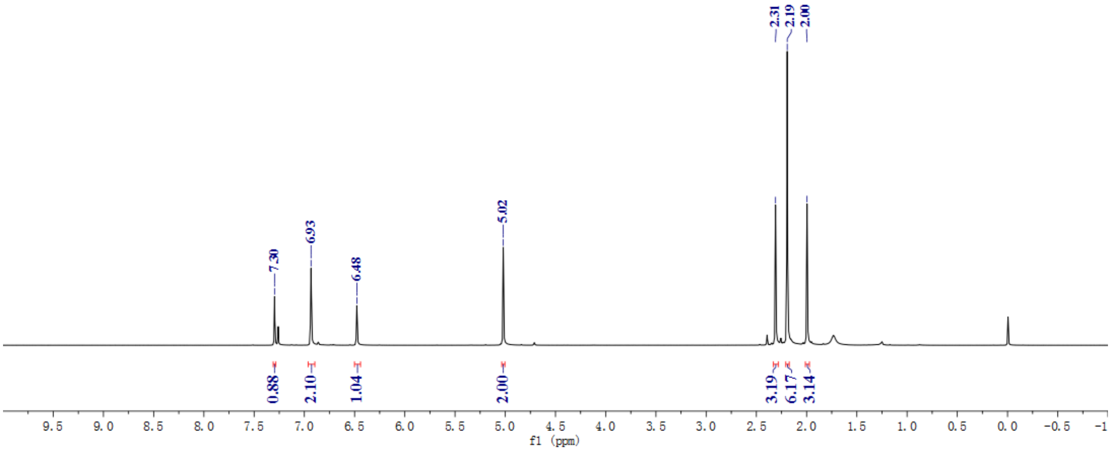

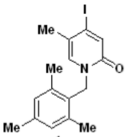

1c

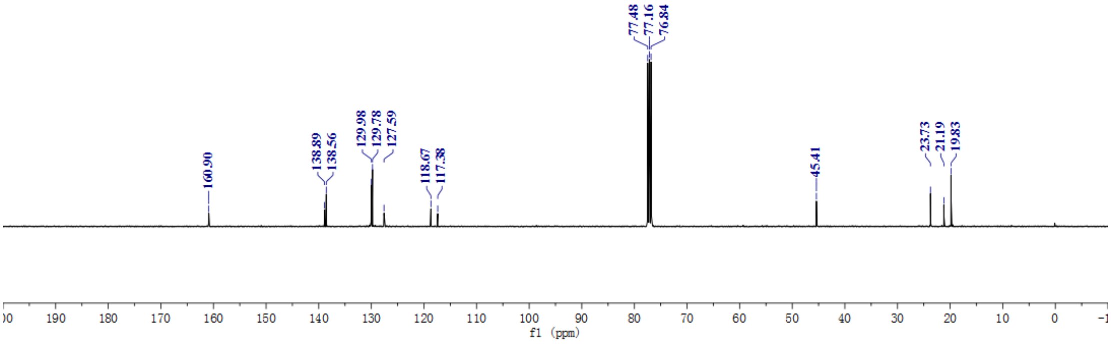

Supplementary Figure 14. NMR of **1c**

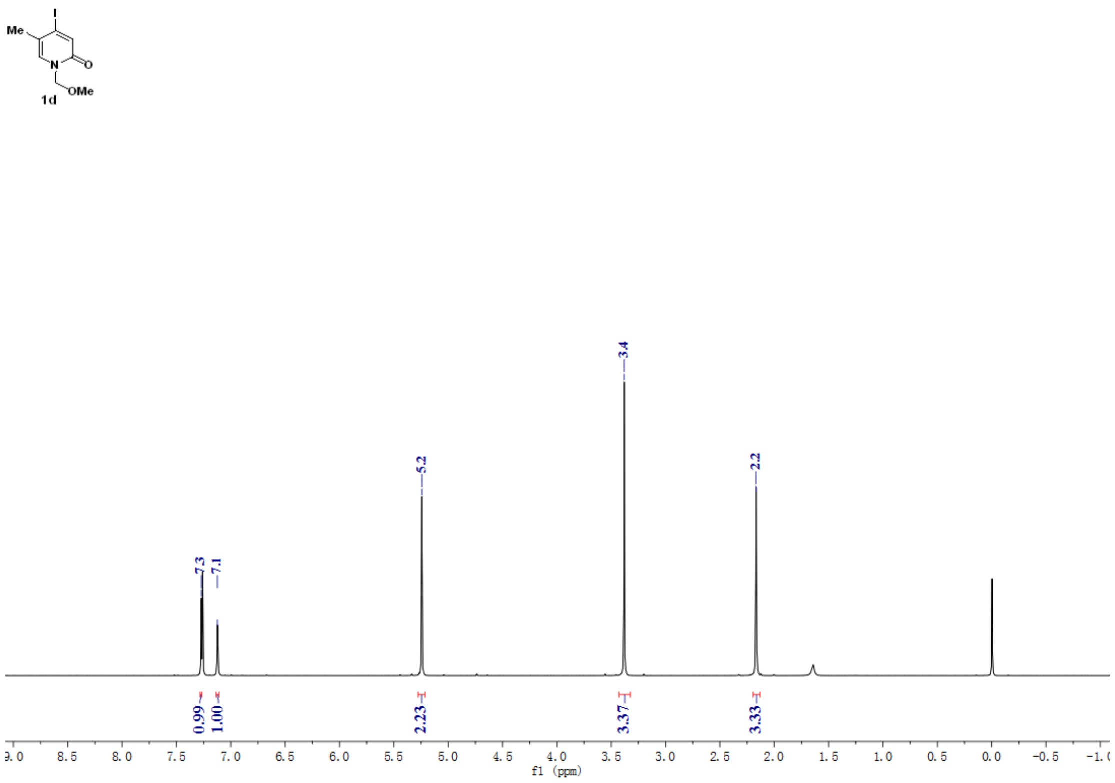

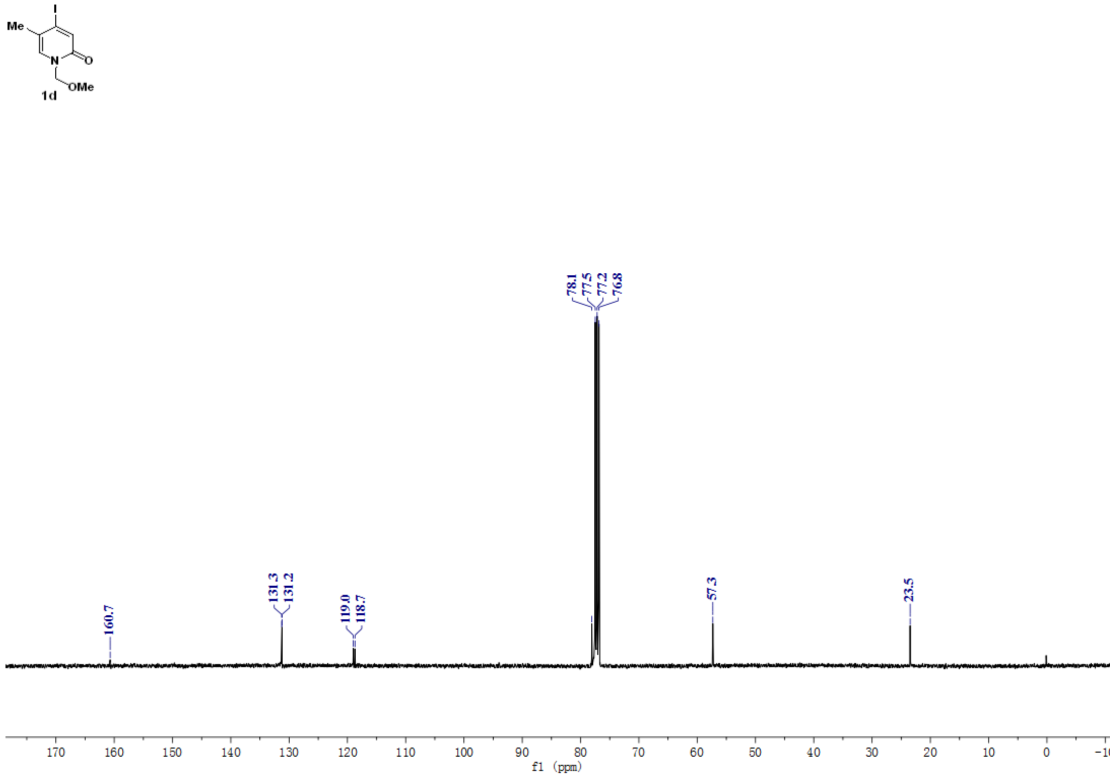

Supplementary Figure 15. NMR of **1d**

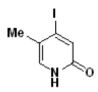

1e

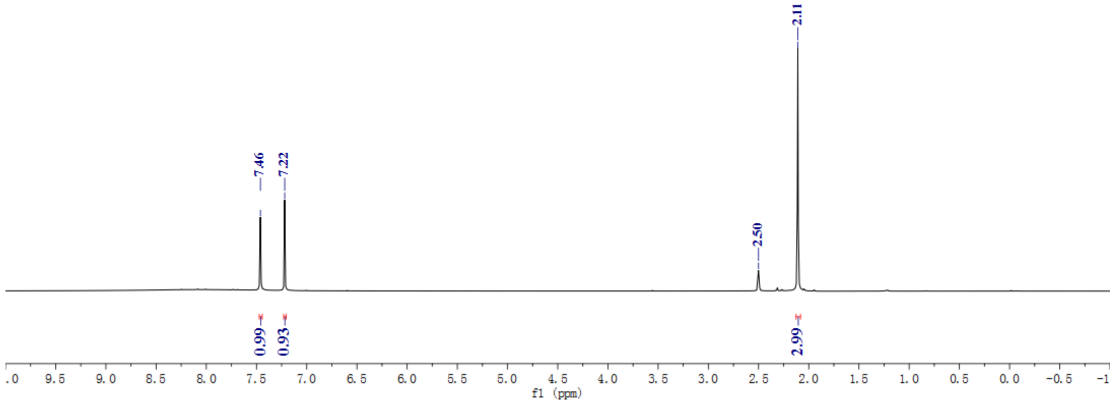

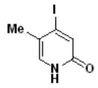

1e

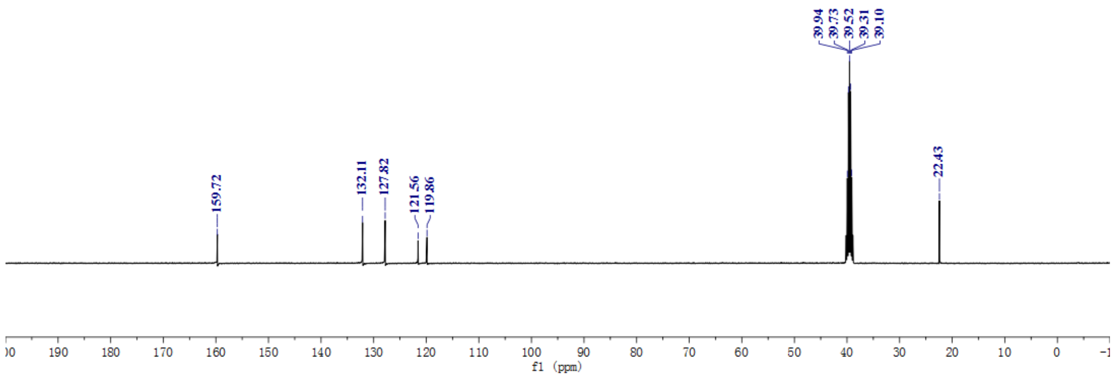

Supplementary Figure 16. NMR of **1e**

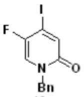
I
F
N
O
Bn

1f

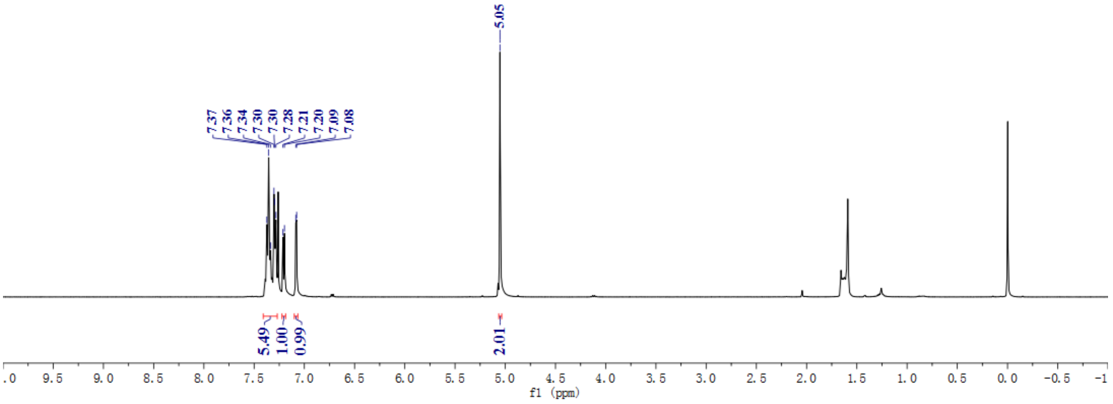
7.37
7.36
7.34
7.30
7.30
7.28
7.21
7.20
7.09
7.08
5.05
5.49
1.00
0.99
2.01
f1 (ppm)

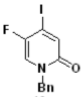
I
F
N
O
Bn

1f

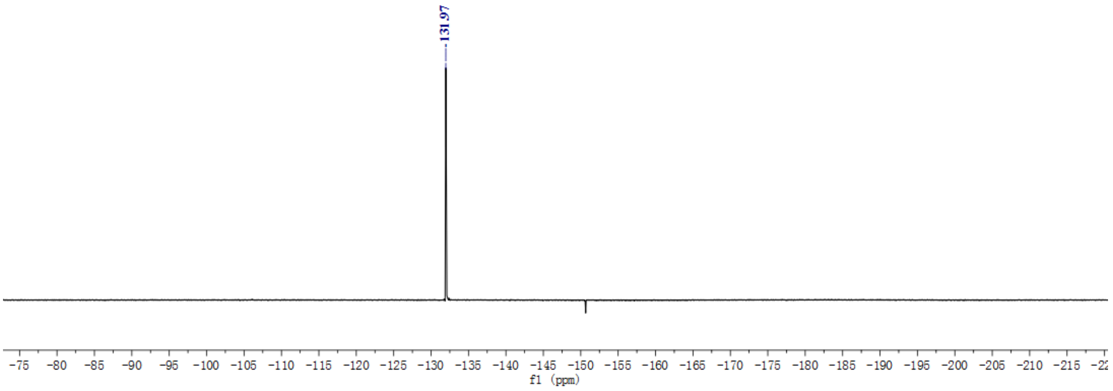
-131.97
f1 (ppm)

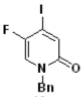

1f

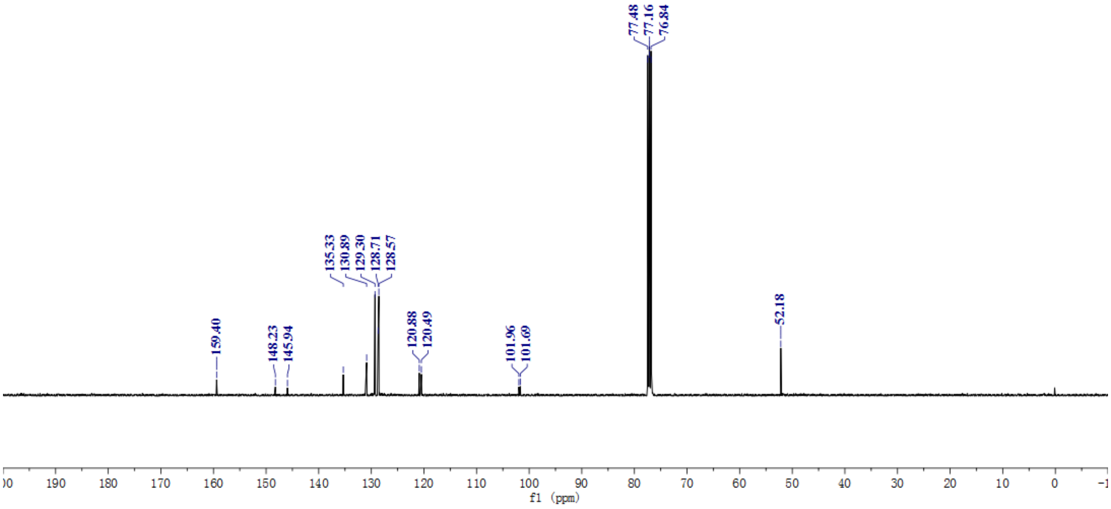

Supplementary Figure 17. NMR of **1f**

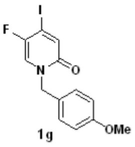

1g

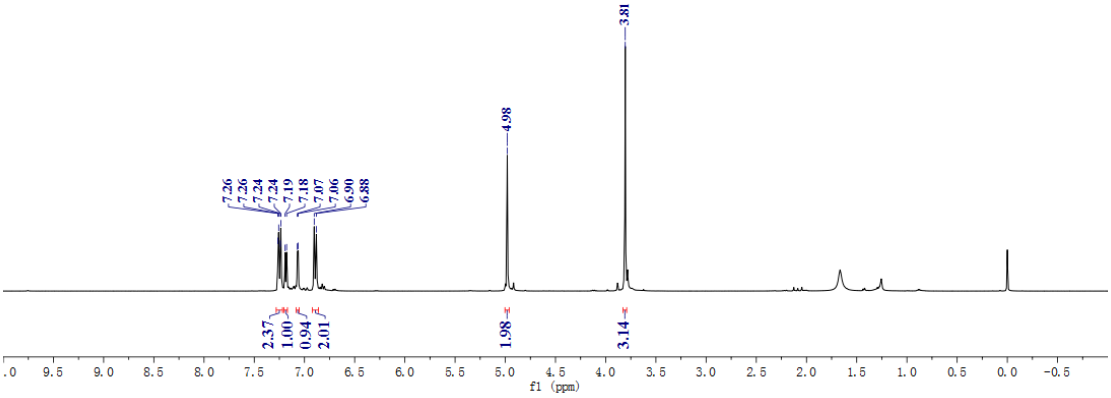

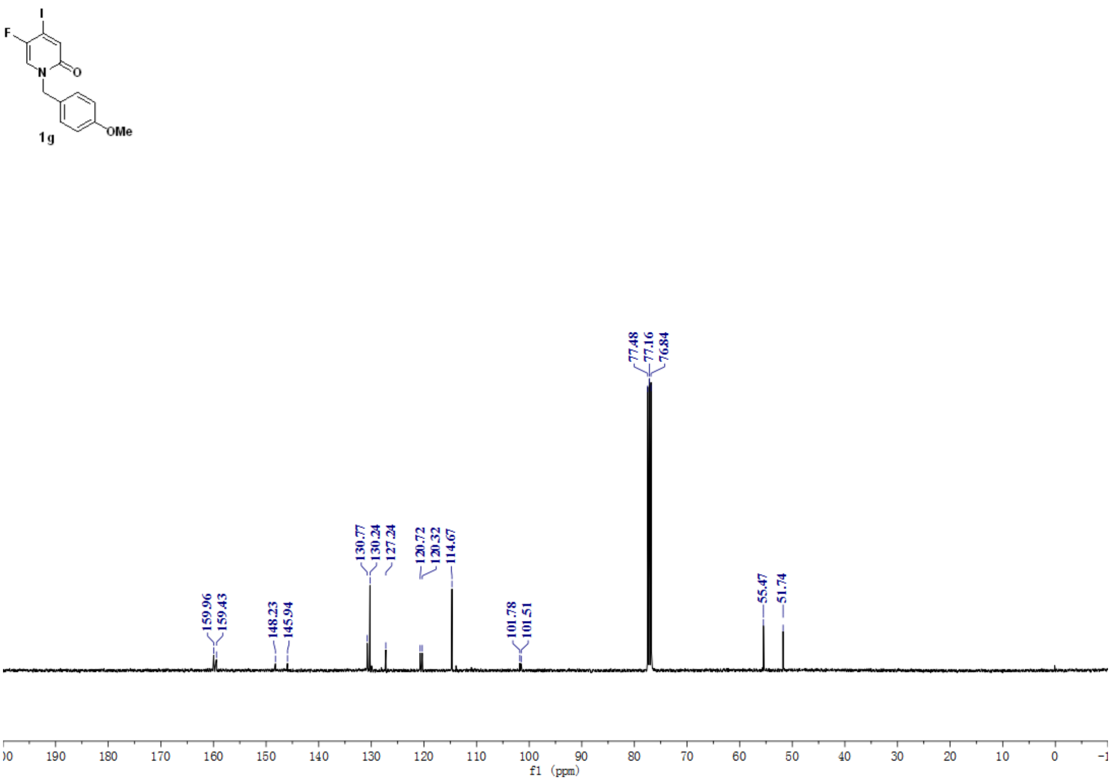

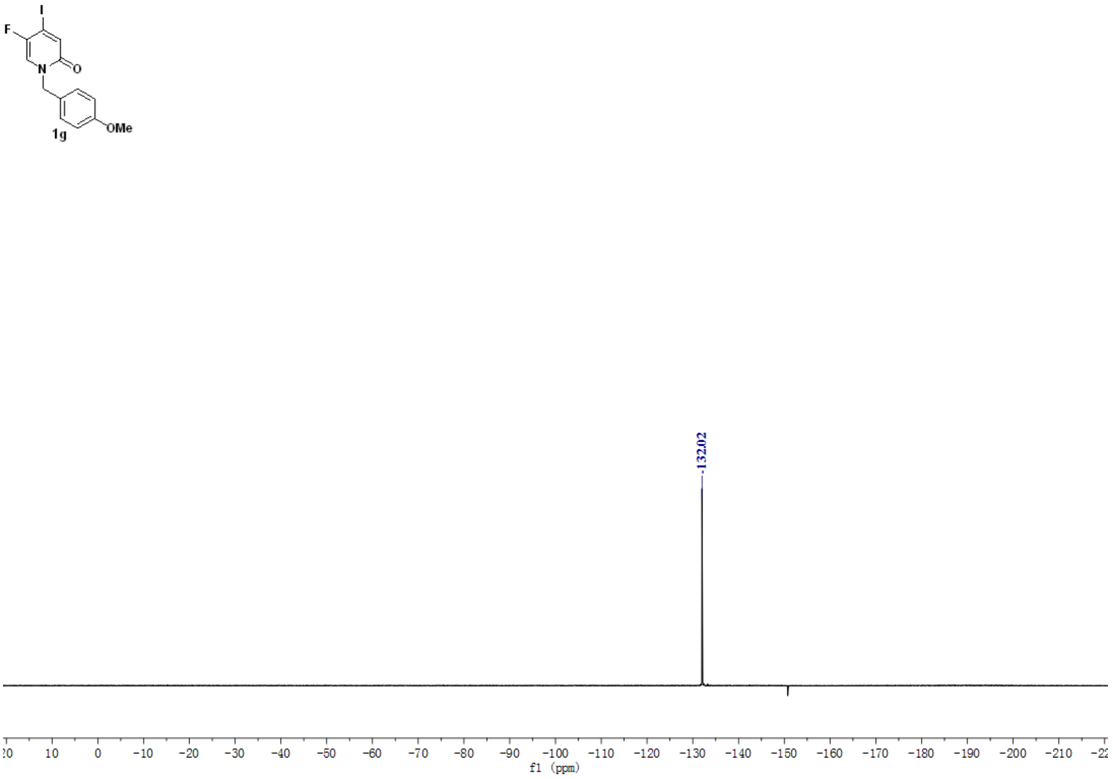

Supplementary Figure 18. NMR of **1g**

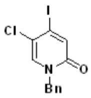

1h

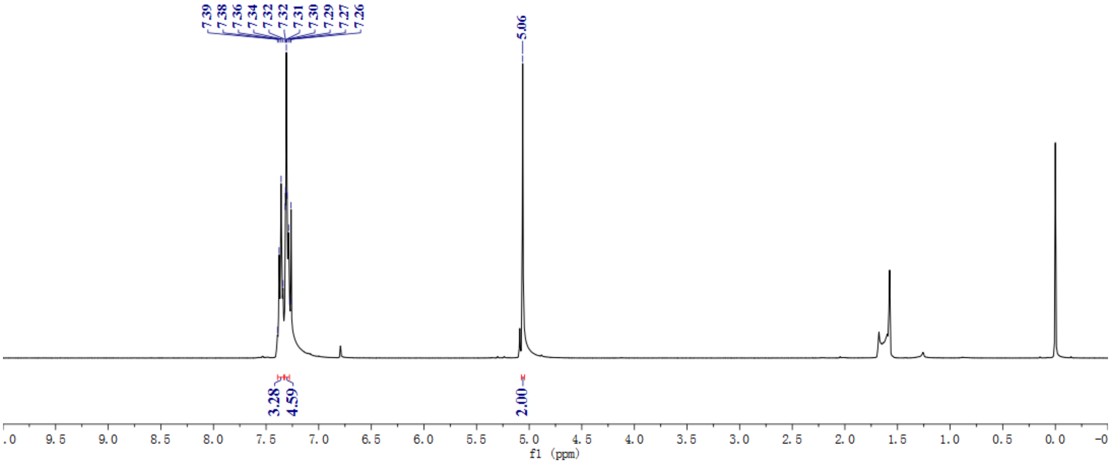

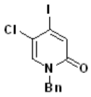

1h

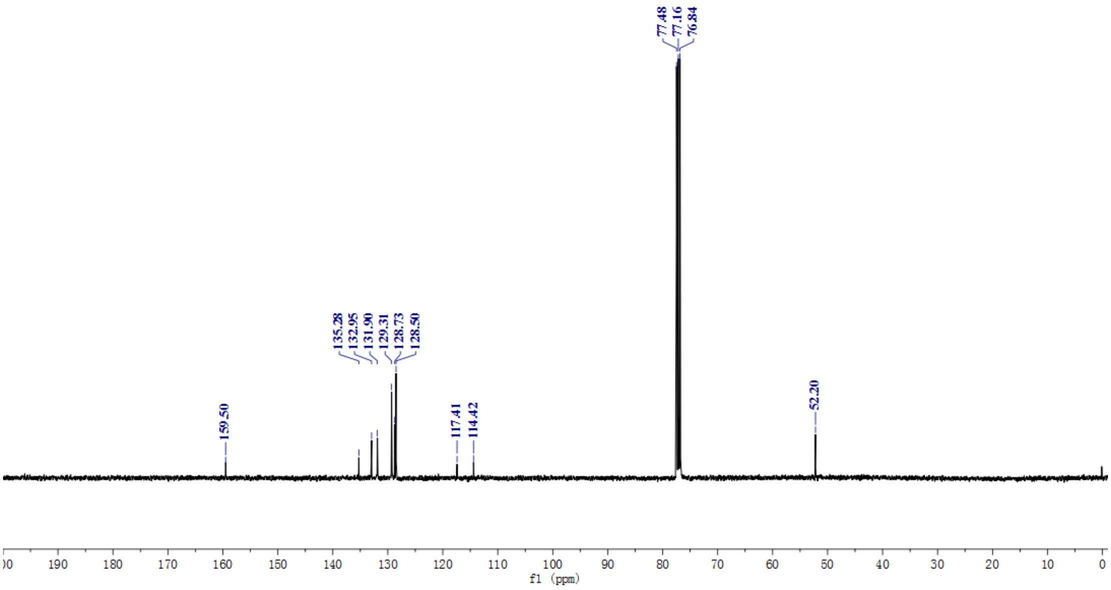

Supplementary Figure 19. NMR of **1h**

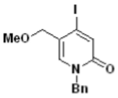

1i

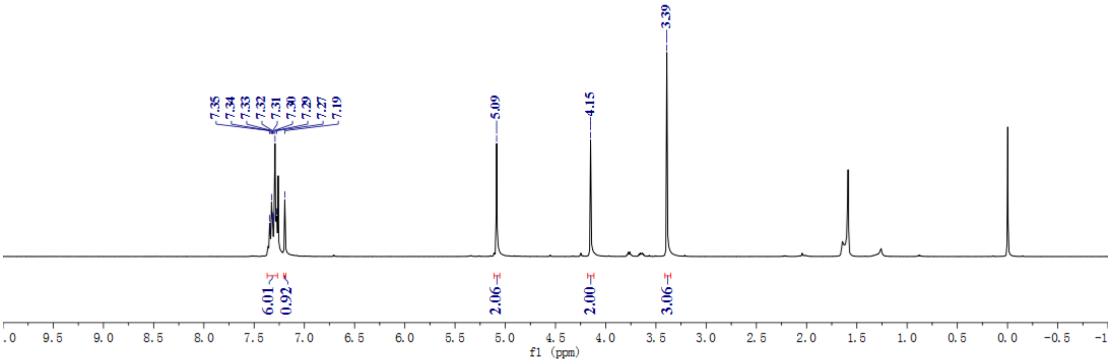

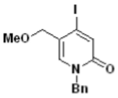

1i

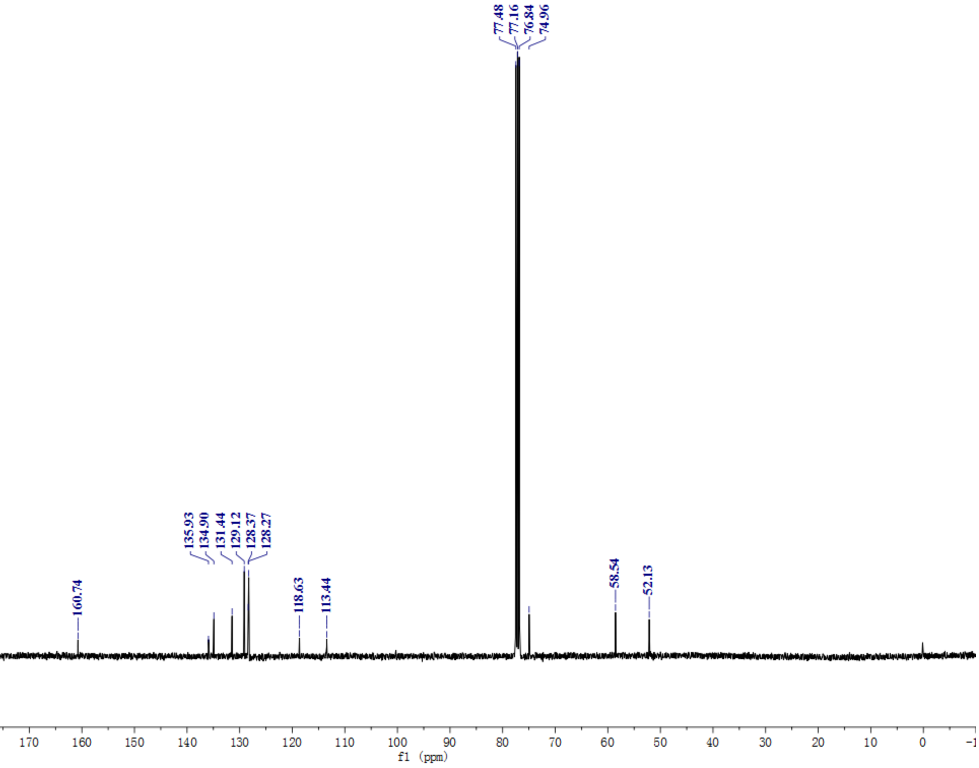

Supplementary Figure 20. NMR of **1i**

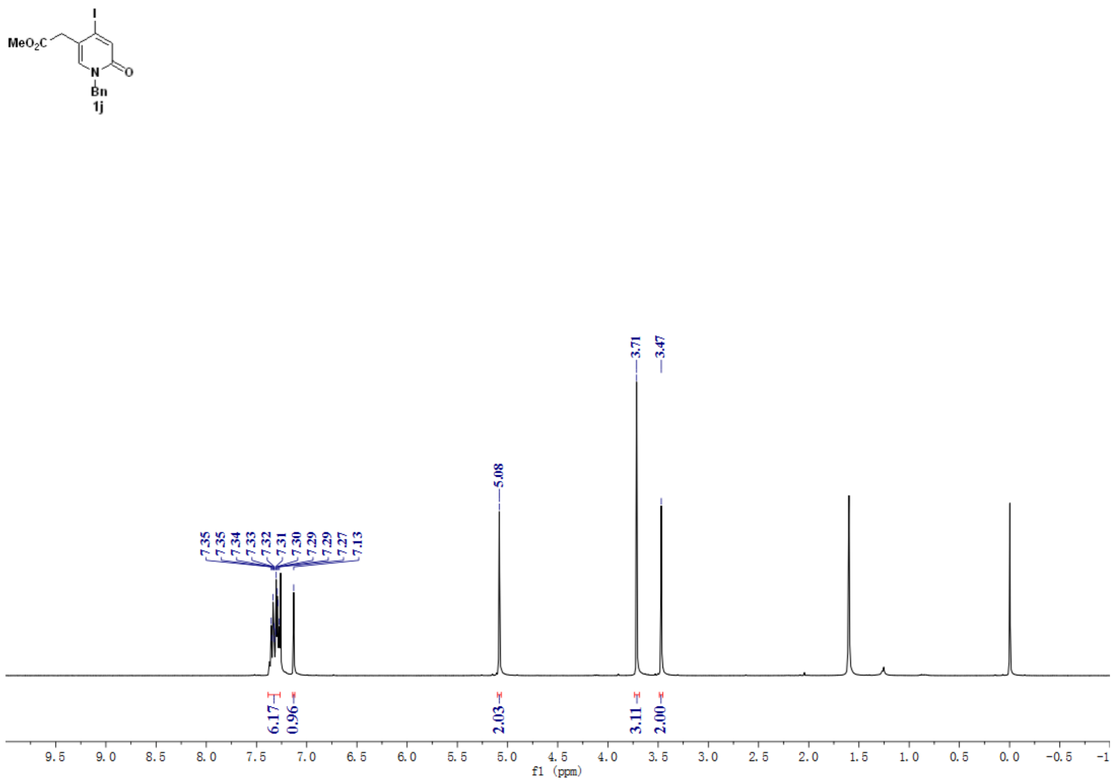

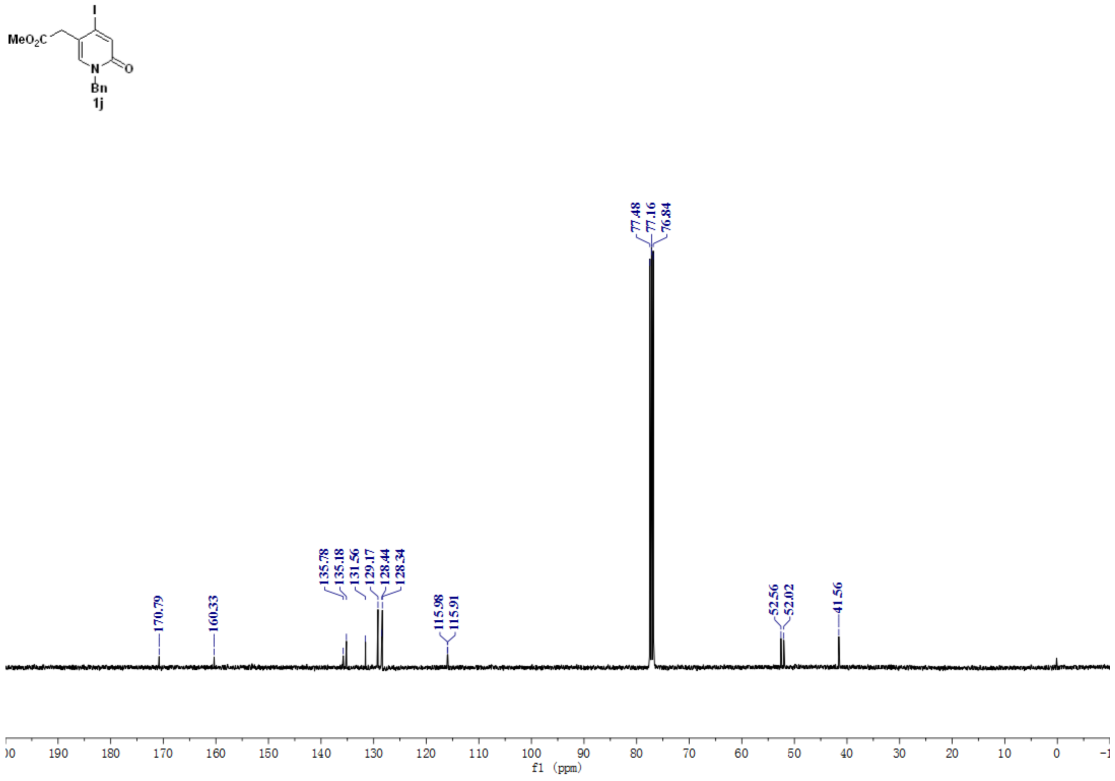

Supplementary Figure 21. NMR of **1j**

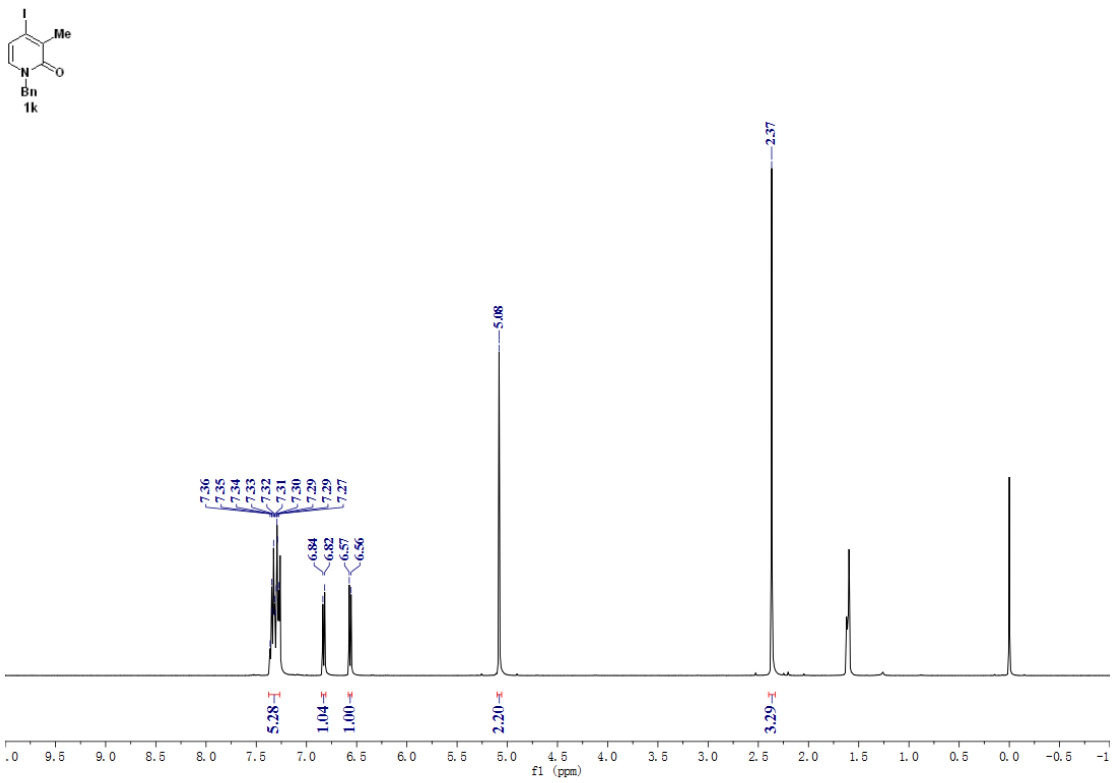

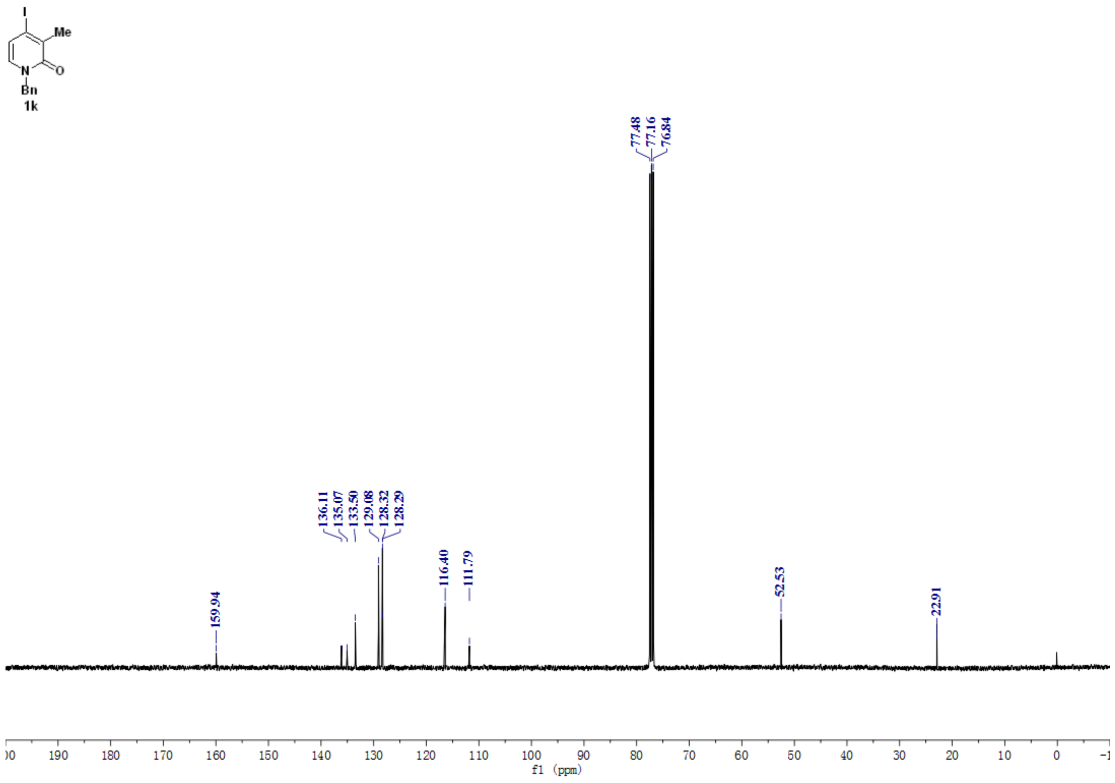

Supplementary Figure 12. NMR of **1k**

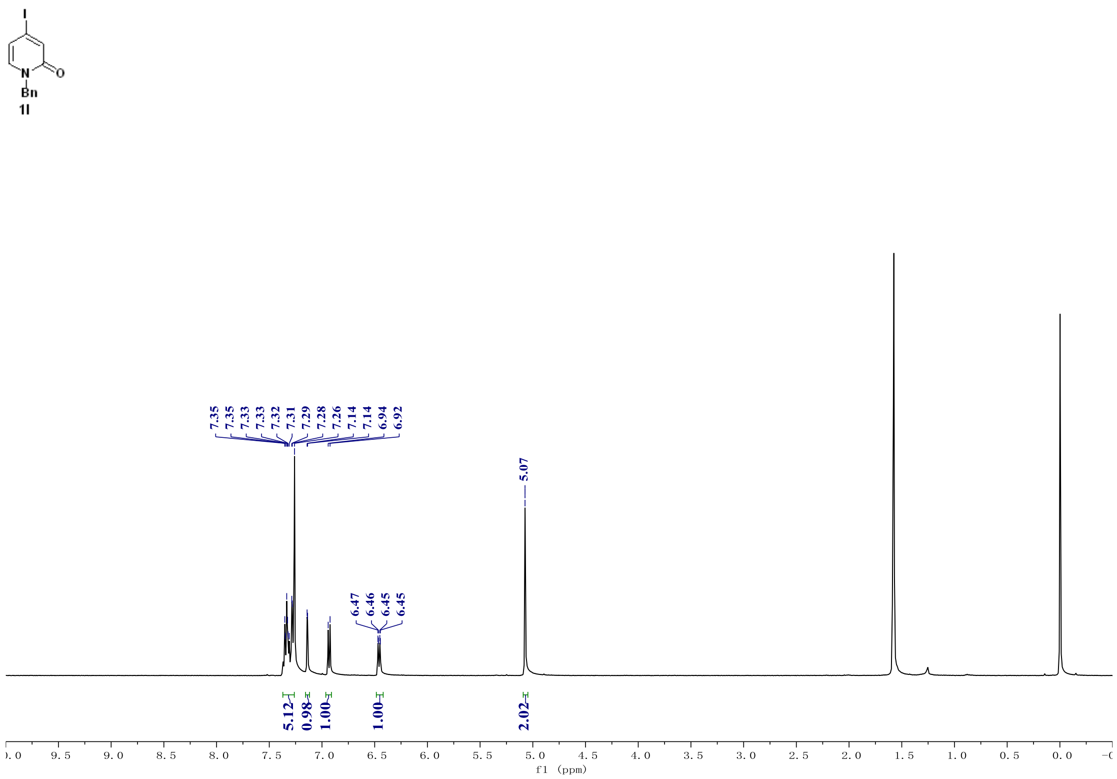

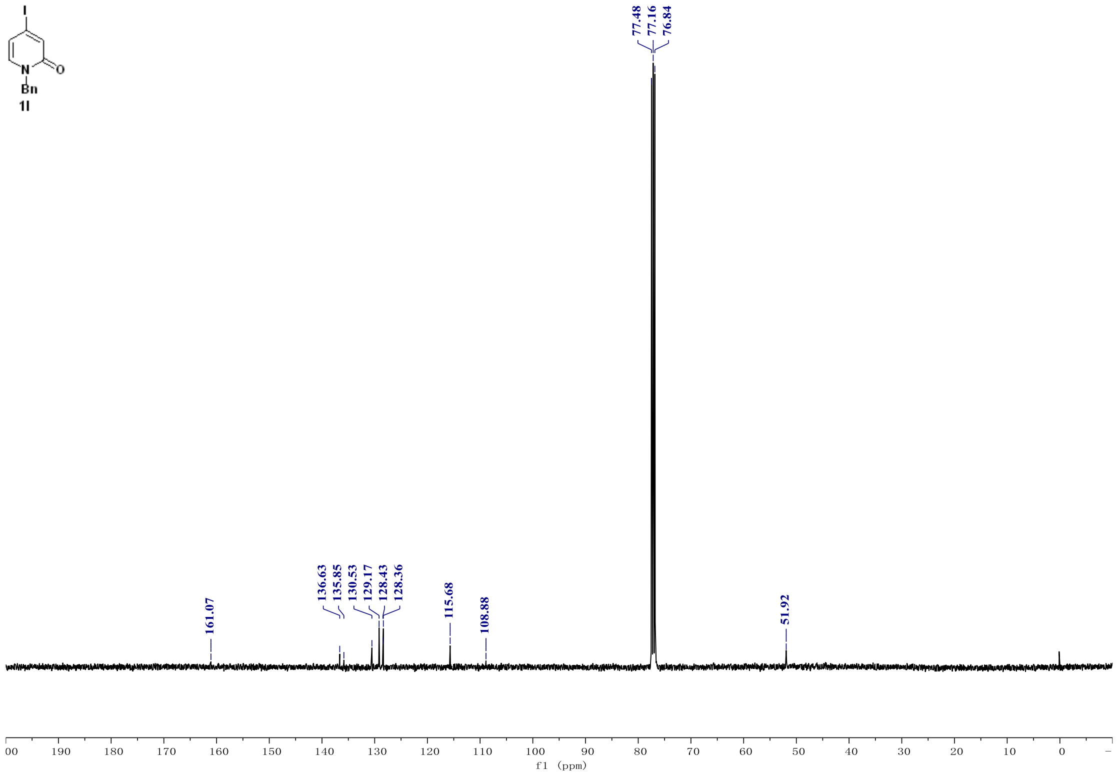

Supplementary Figure 23. NMR of **1l**

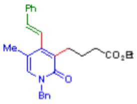

4a

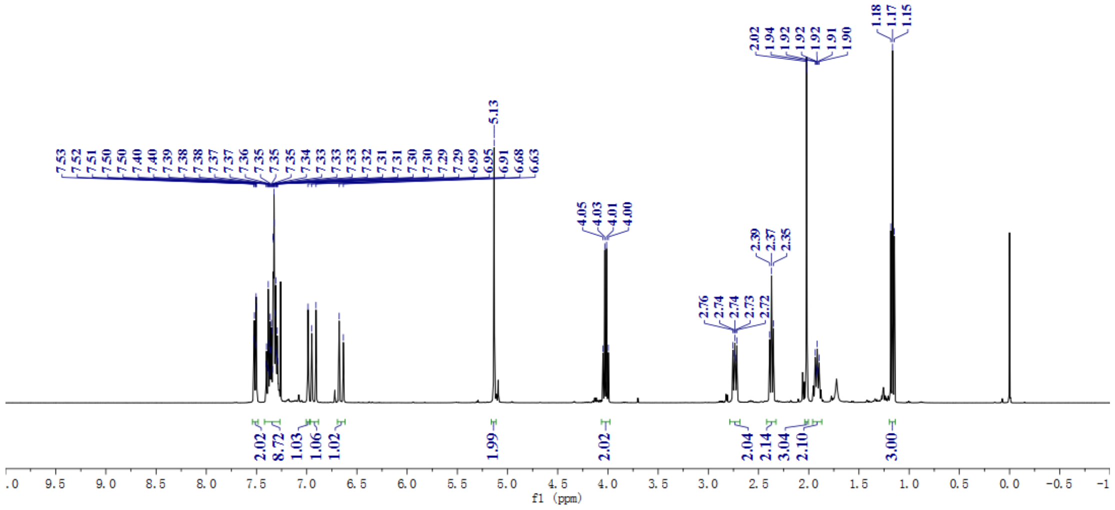

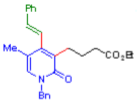

4a

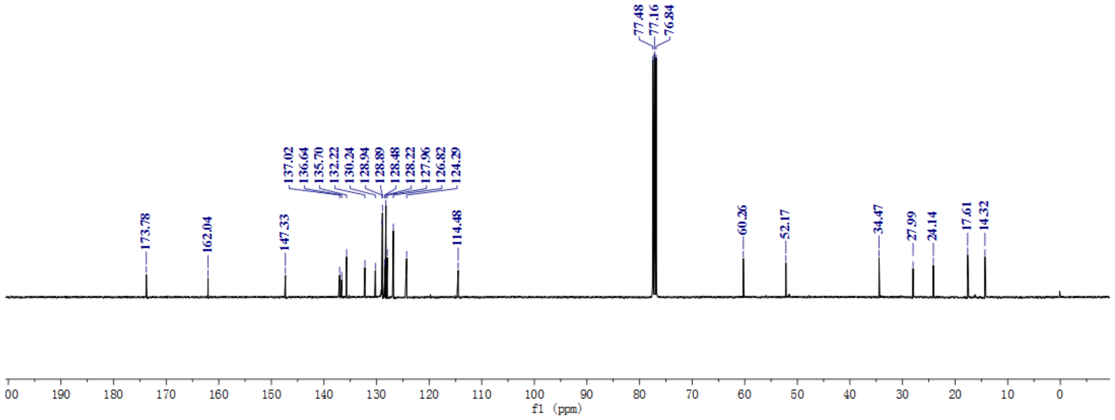

Supplementary Figure 24. NMR of **4a**

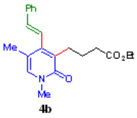

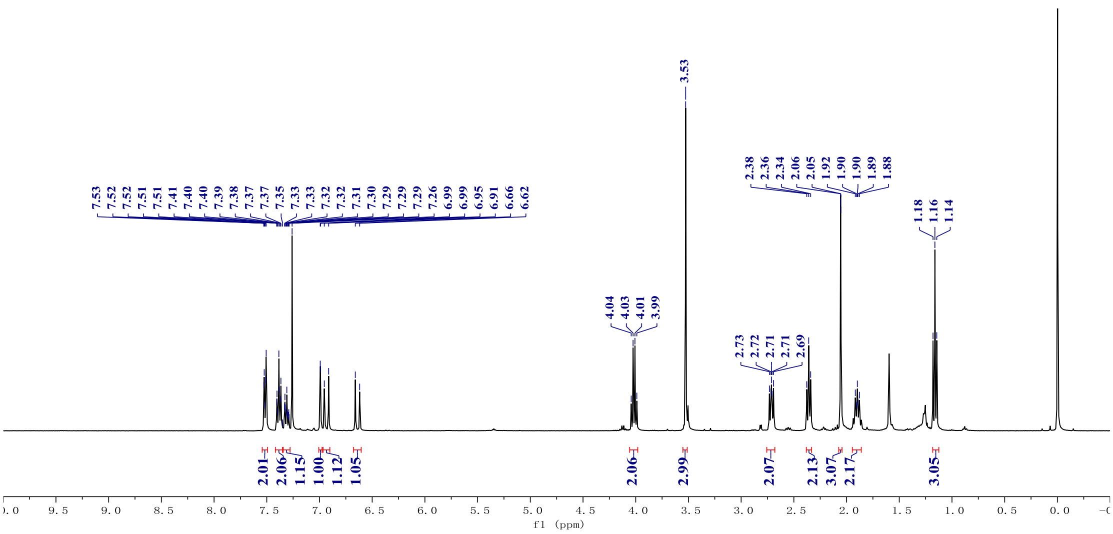

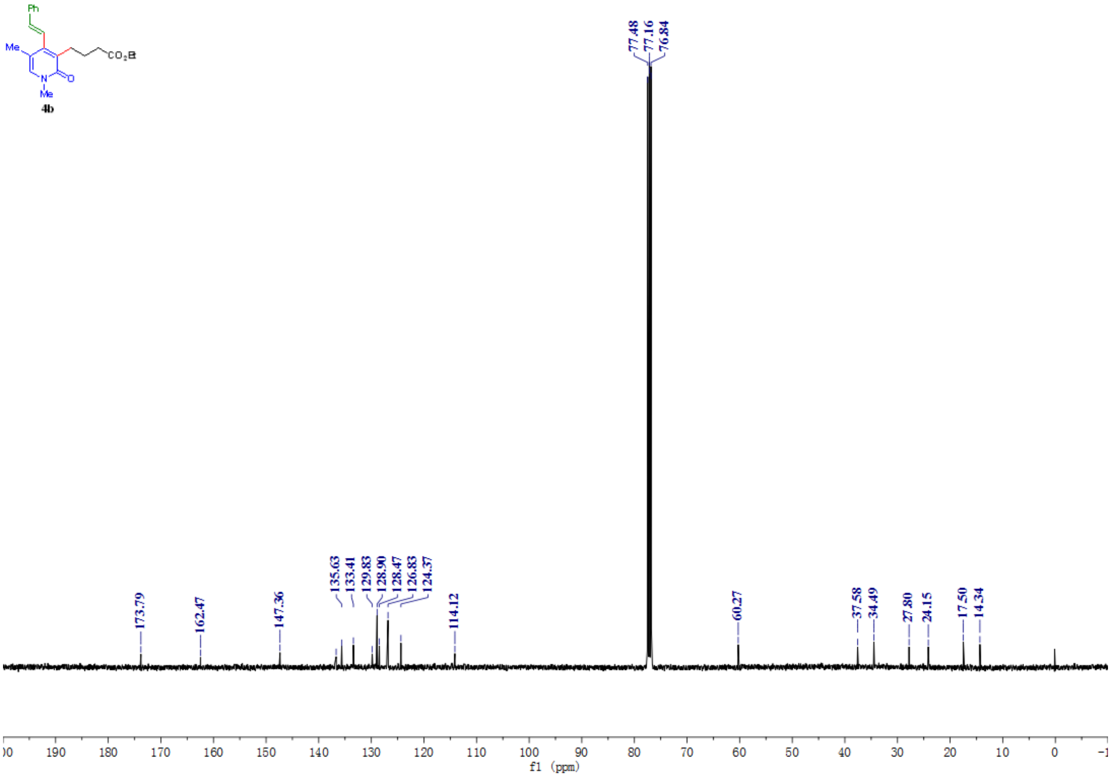

Supplementary Figure 25. NMR of **4b**

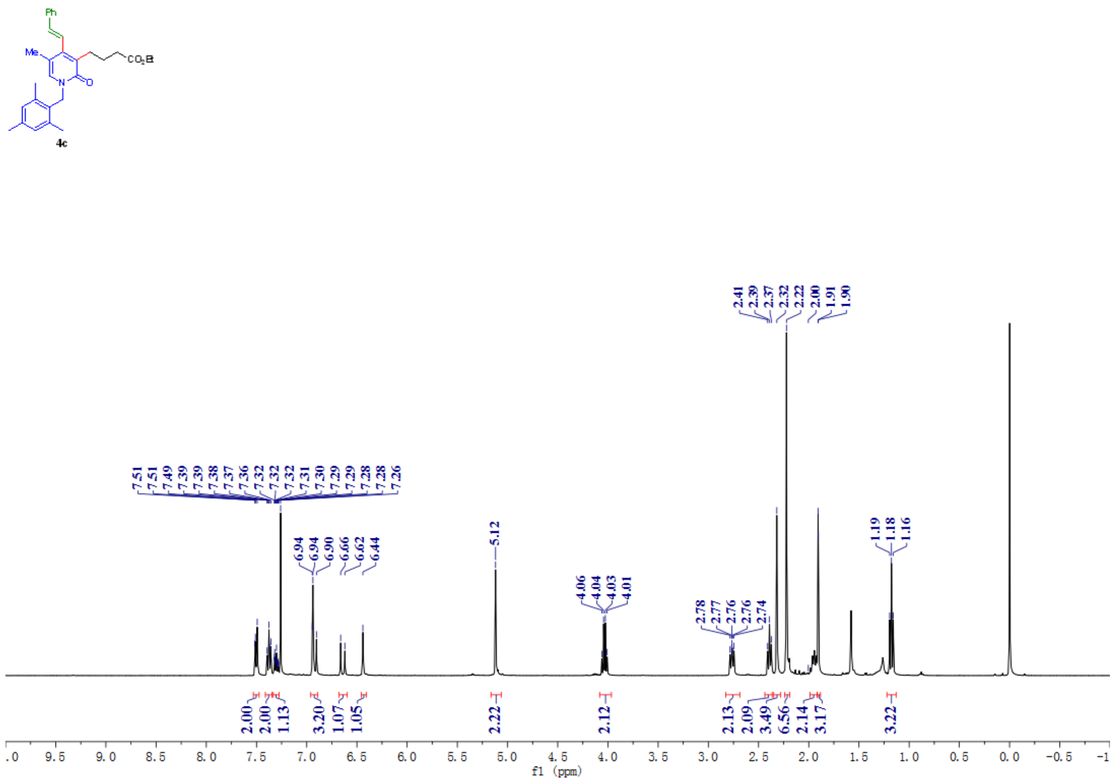

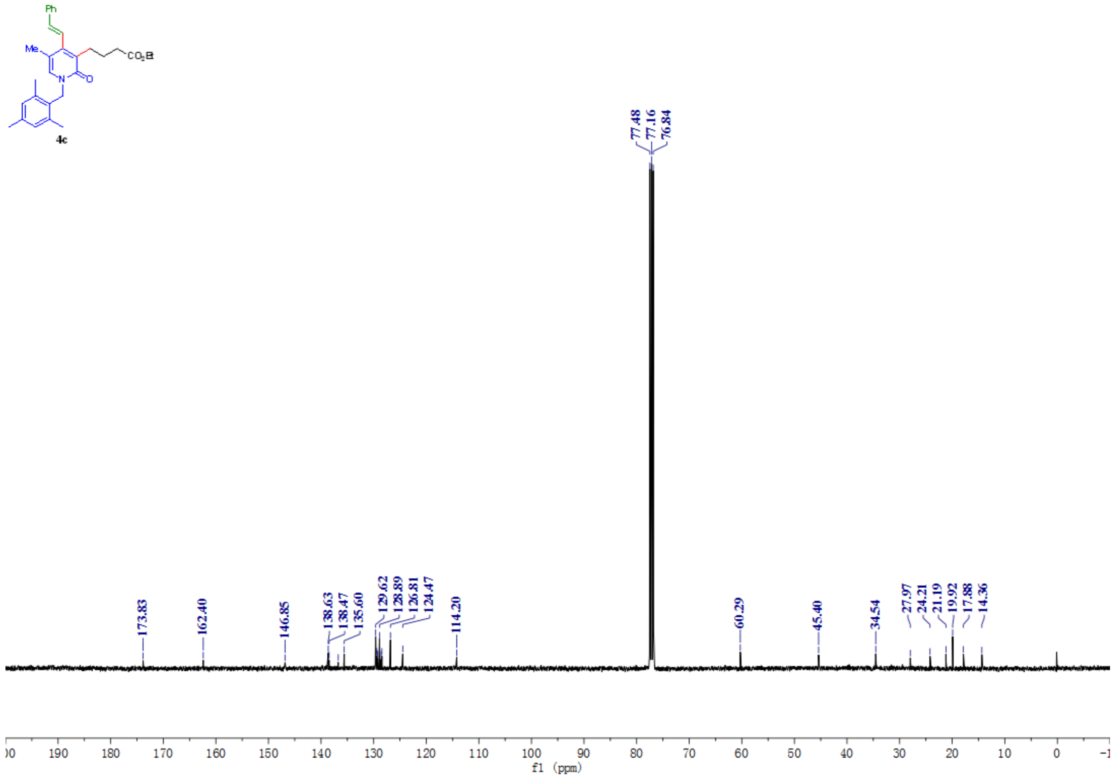

Supplementary Figure 26. NMR of **4c**

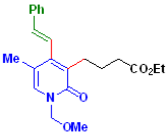

4d

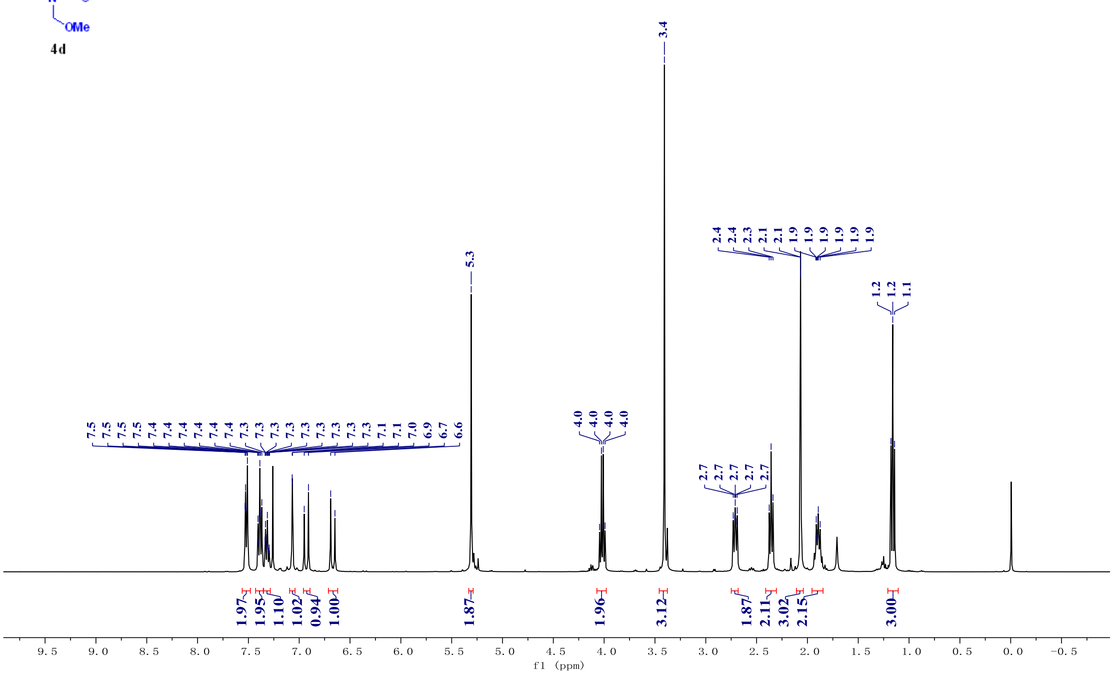

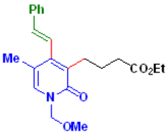

4d

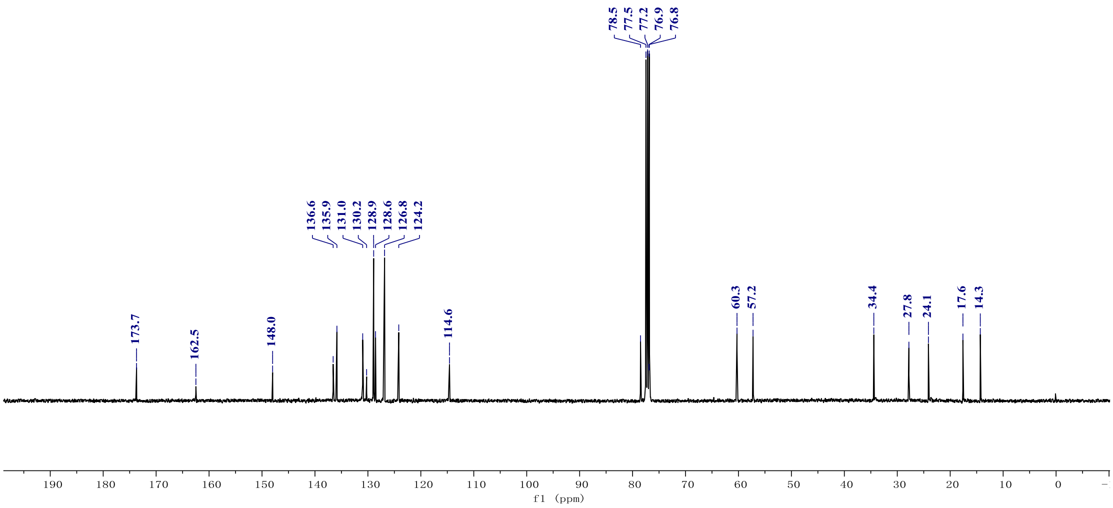

Supplementary Figure 27. NMR of **4d**

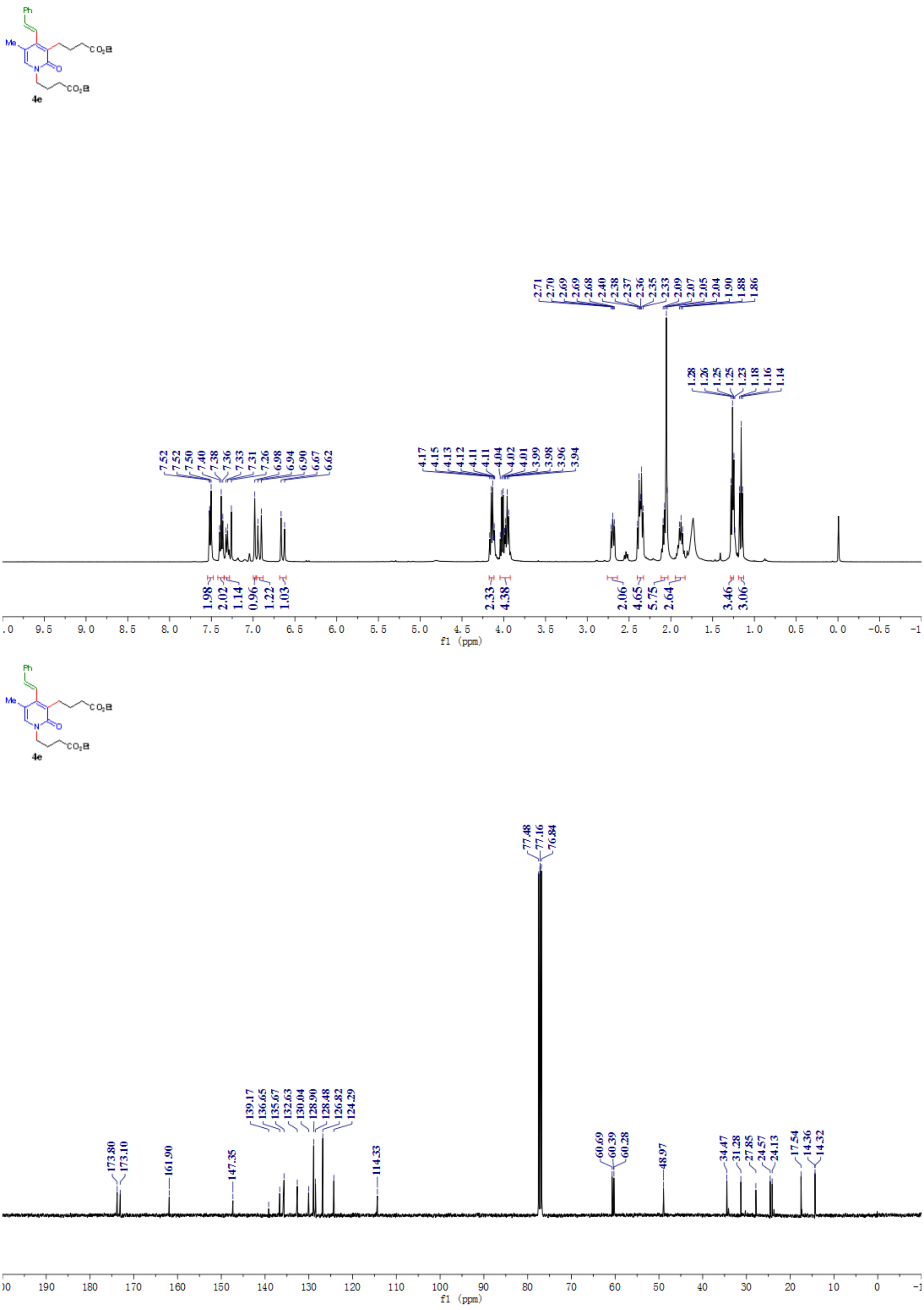

Supplementary Figure 28. NMR of **4e**

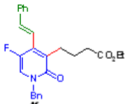

4f

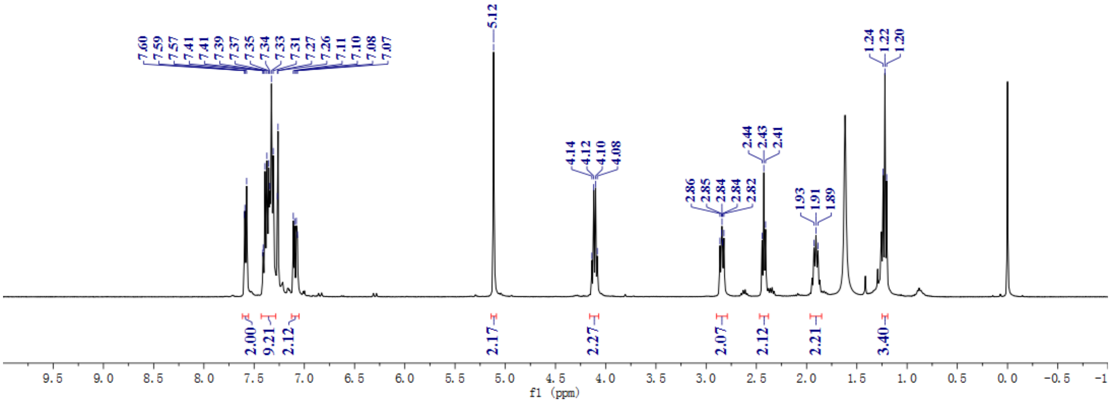

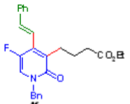

4f

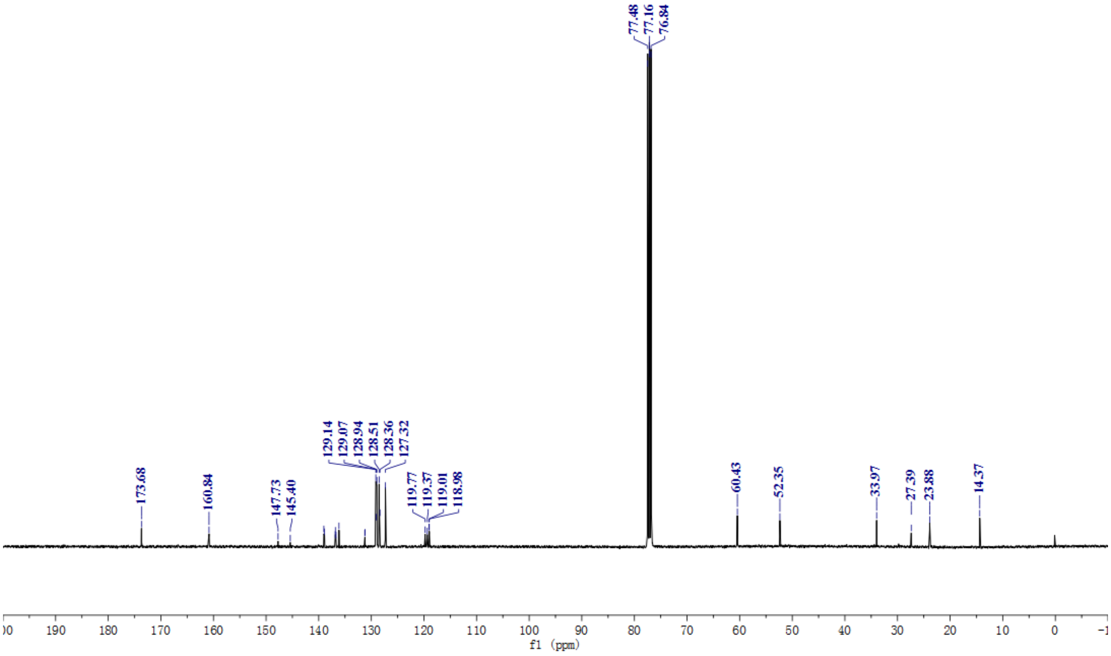

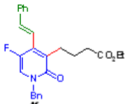

4f

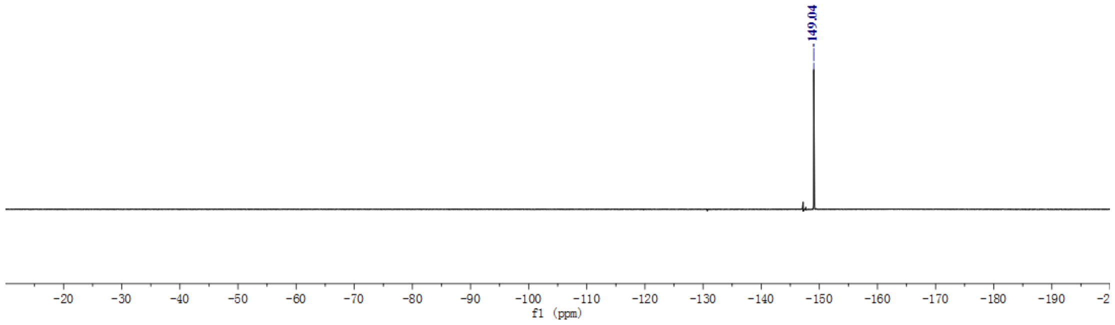

Supplementary Figure 29. NMR of **4f**

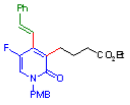

4g

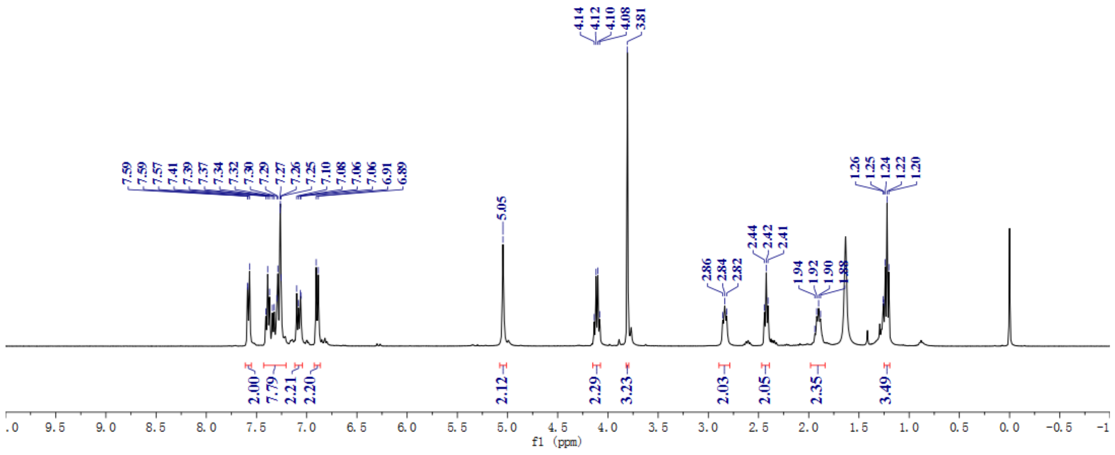

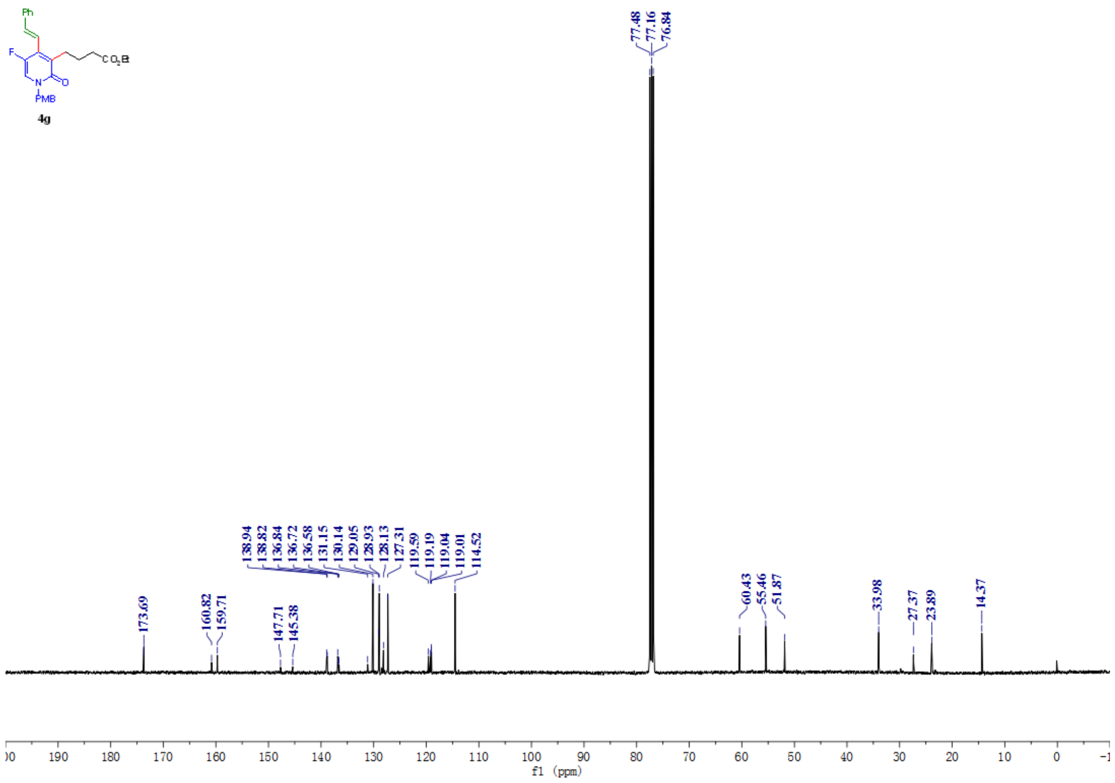

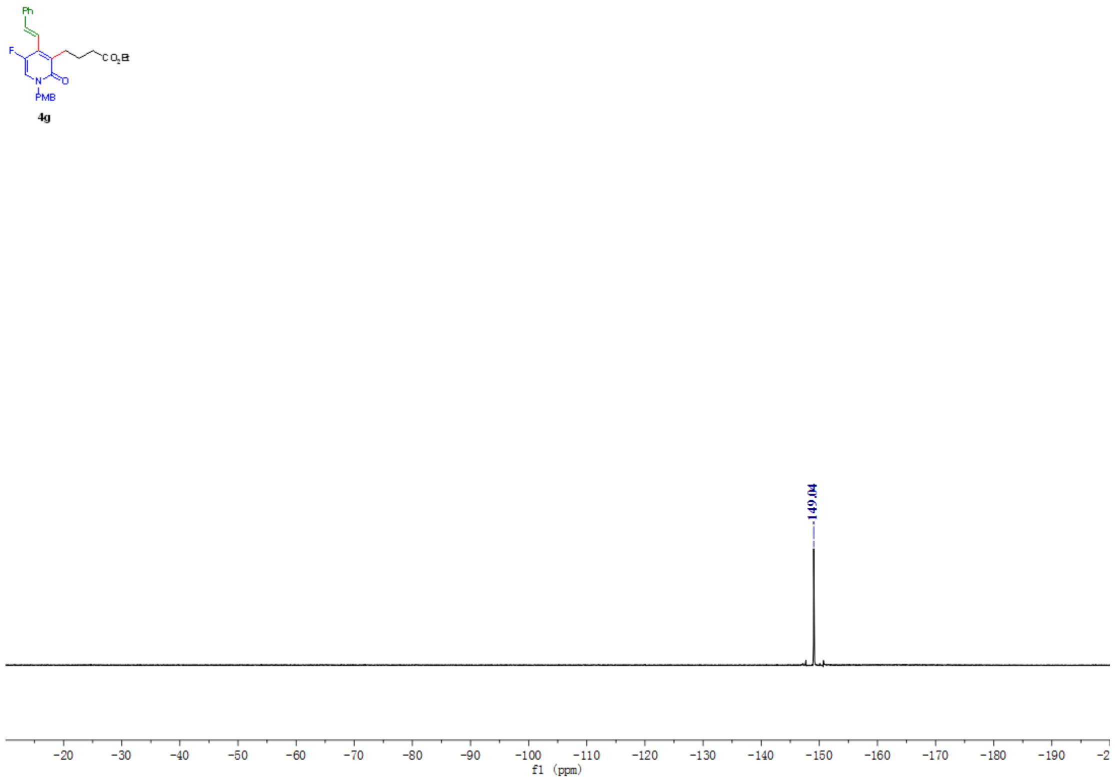

Supplementary Figure 30. NMR of **4g**

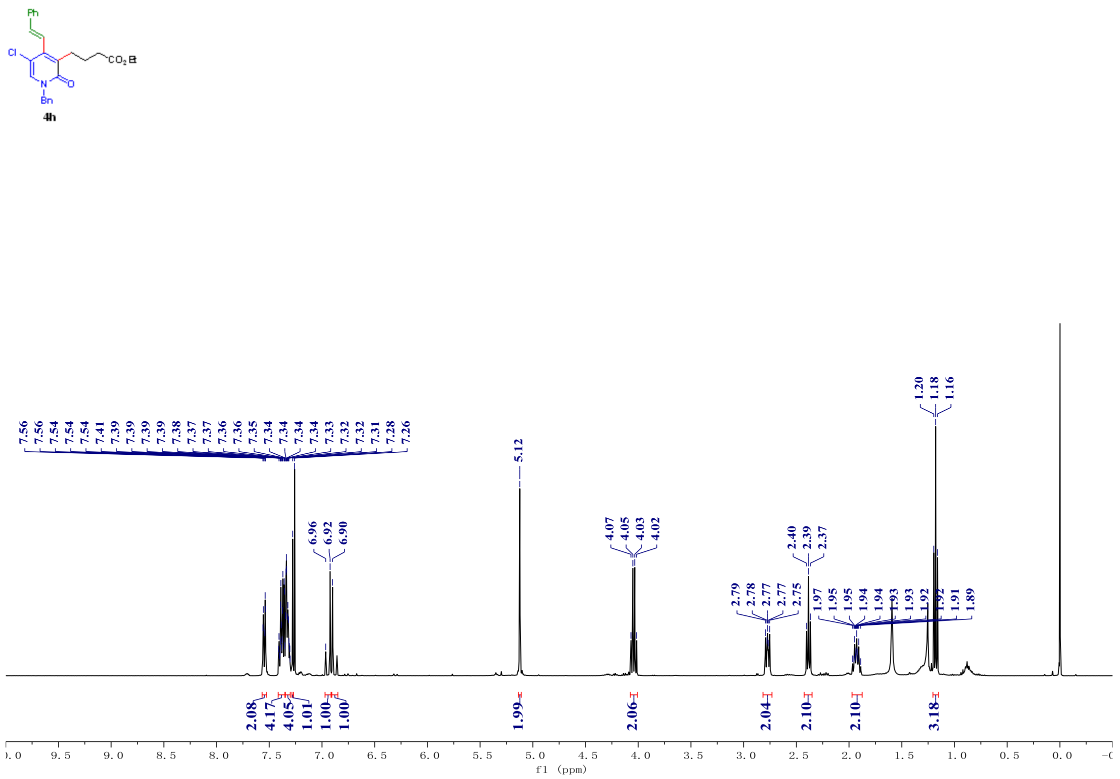

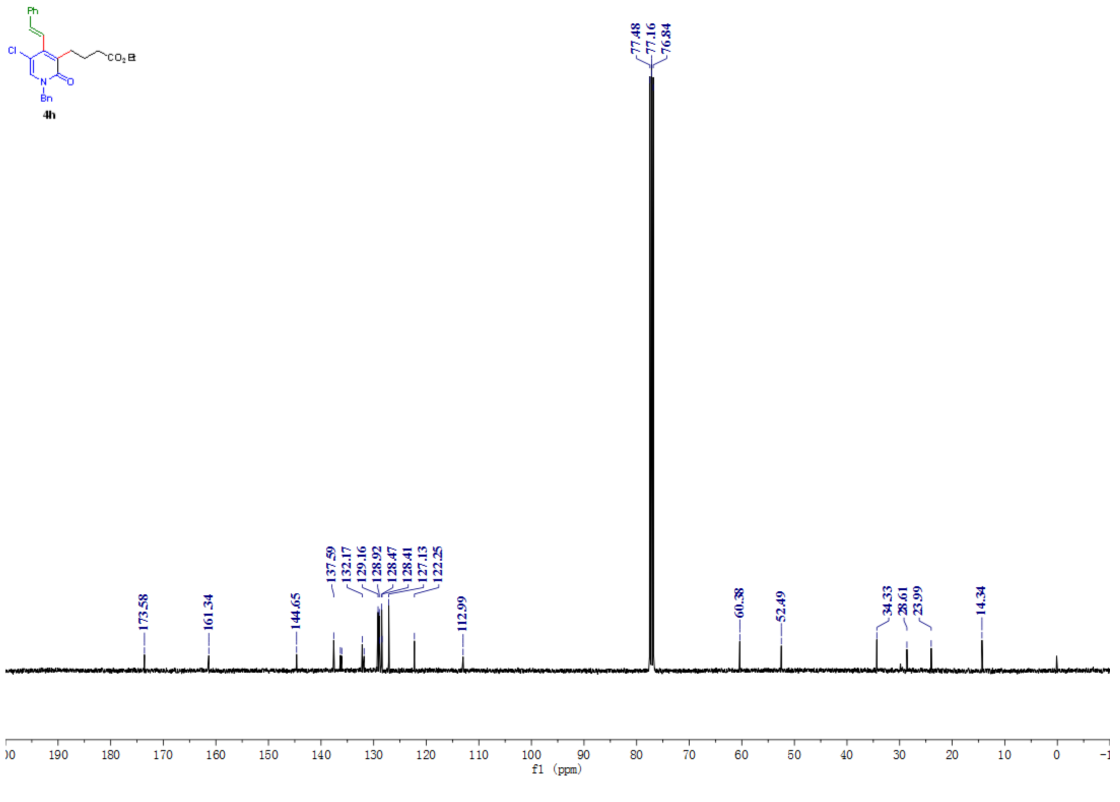

Supplementary Figure 31. NMR of **4h**

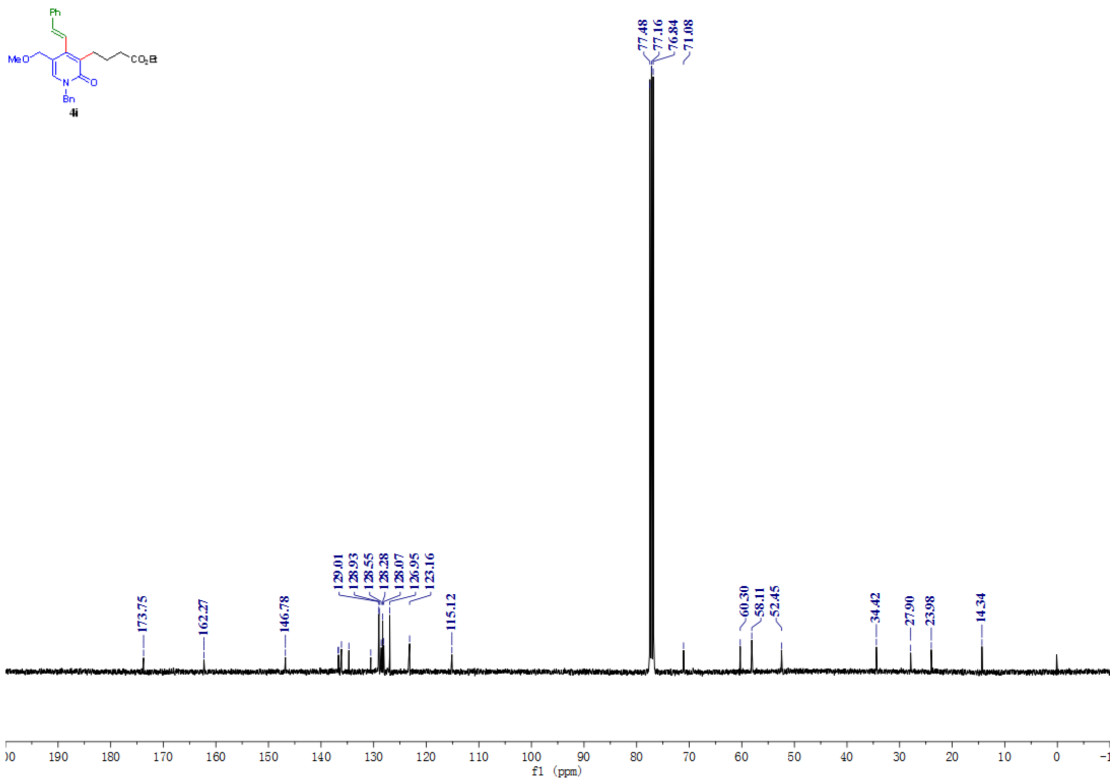

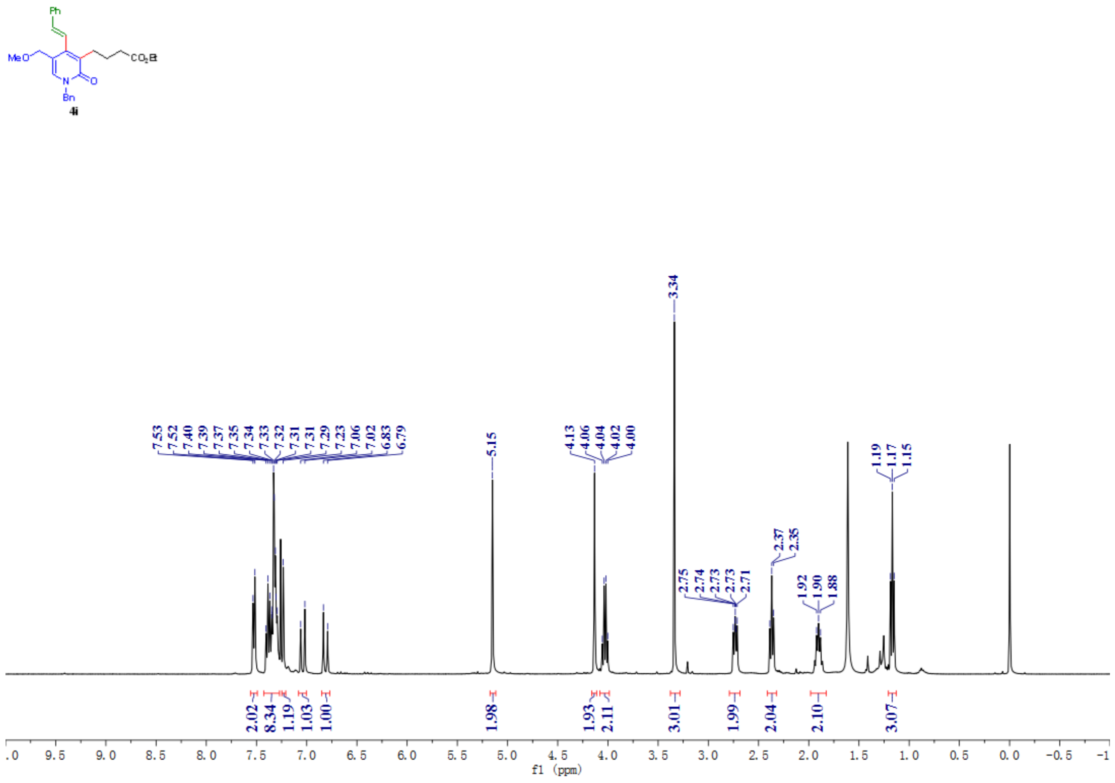

Supplementary Figure 32. NMR of **4i**

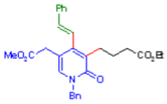

4j

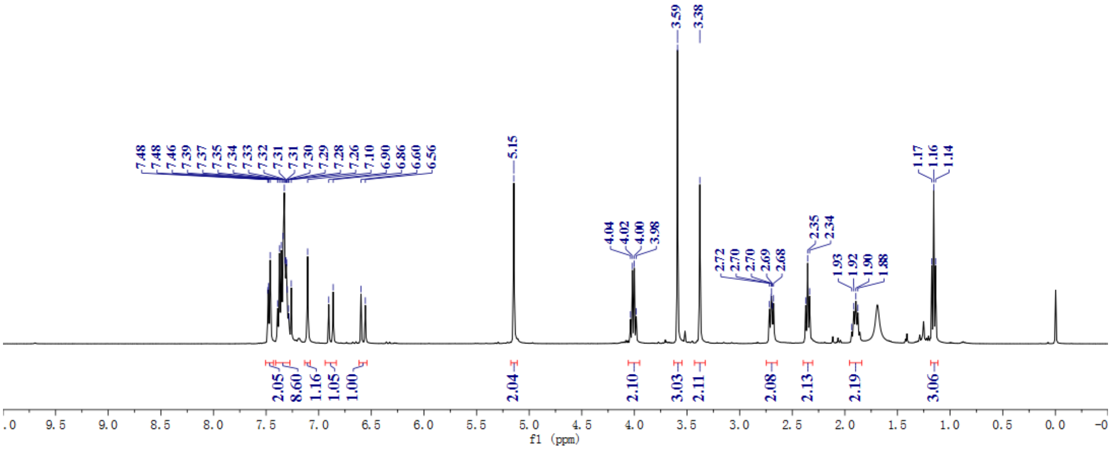

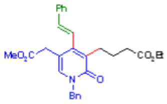

4j

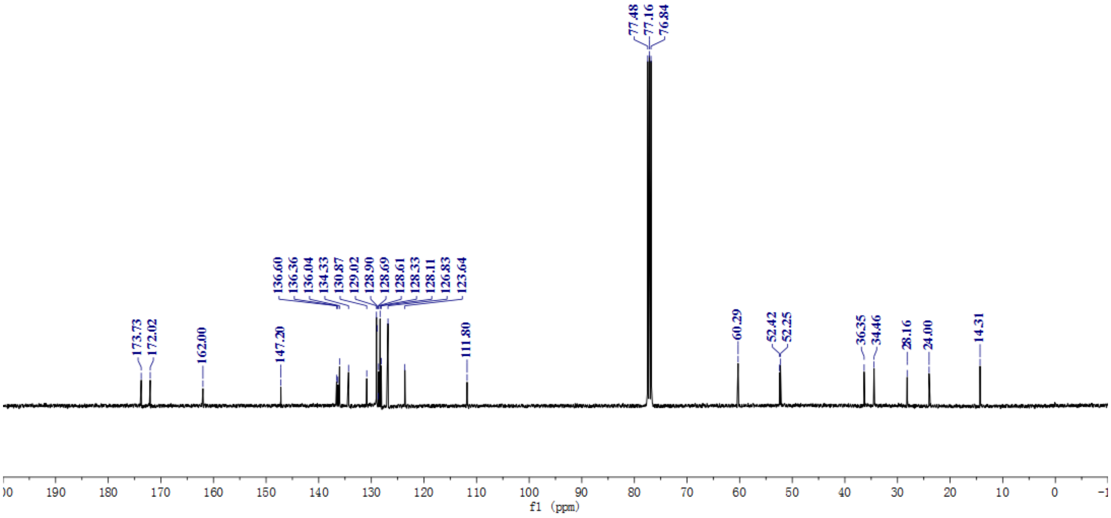

Supplementary Figure 33. NMR of **4j**

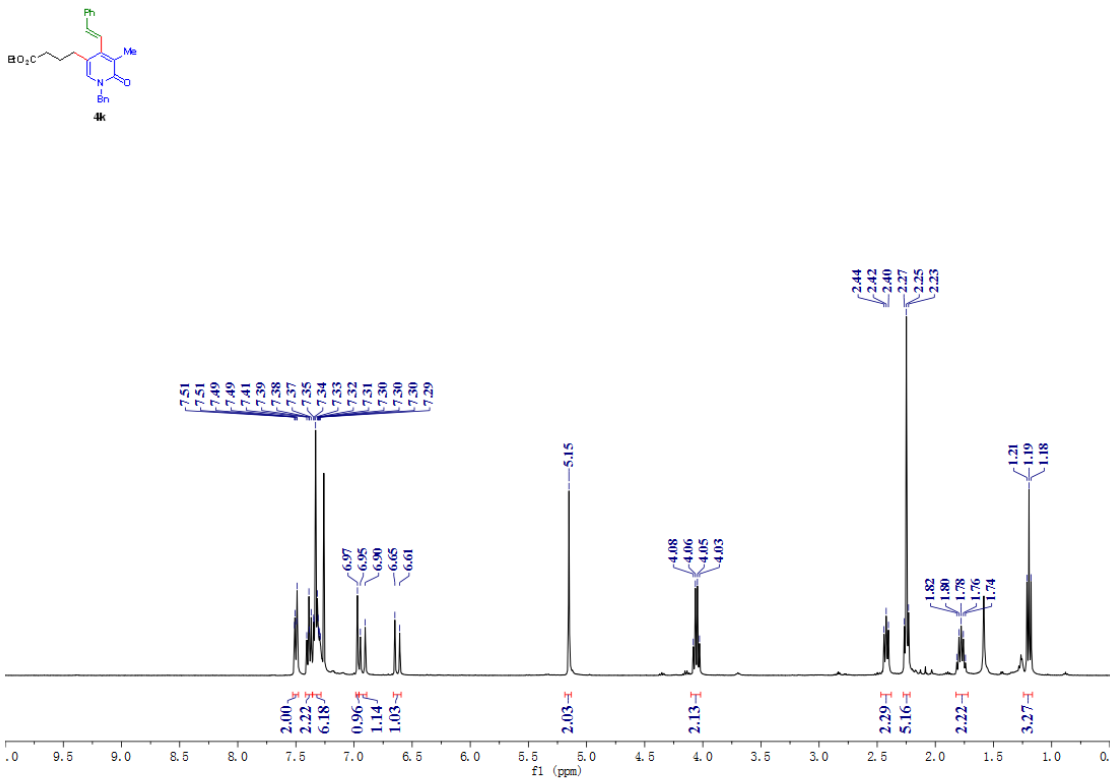

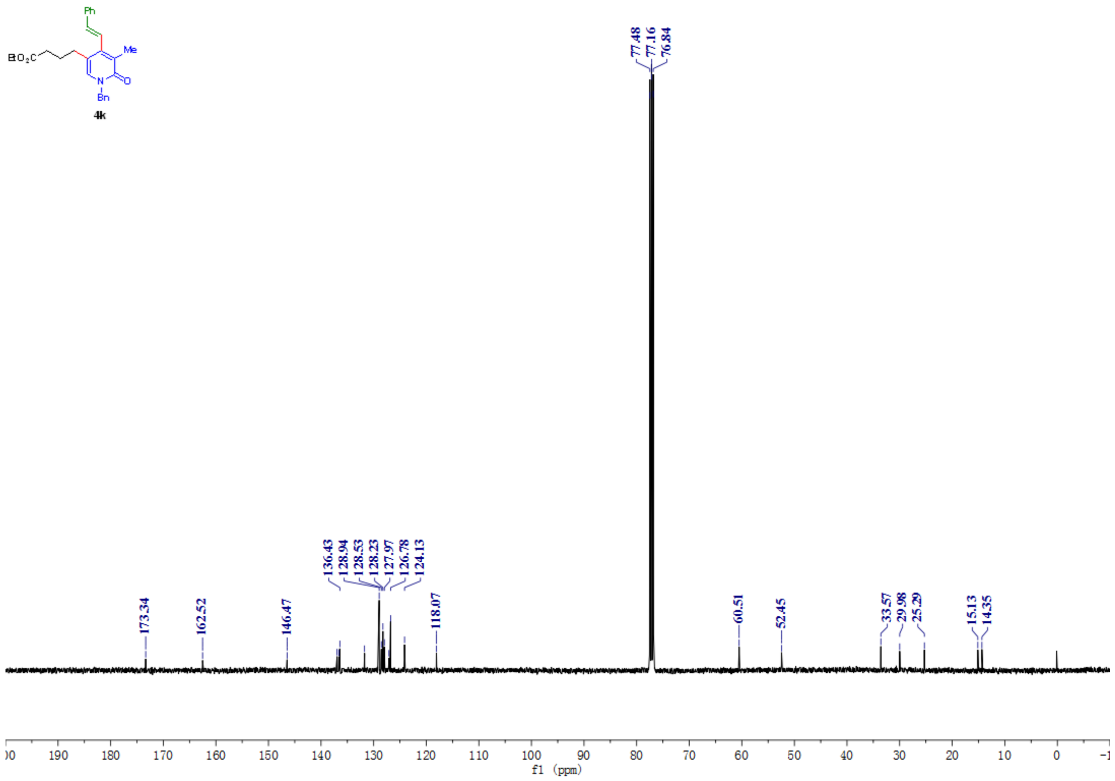

Supplementary Figure 34. NMR of **4k**

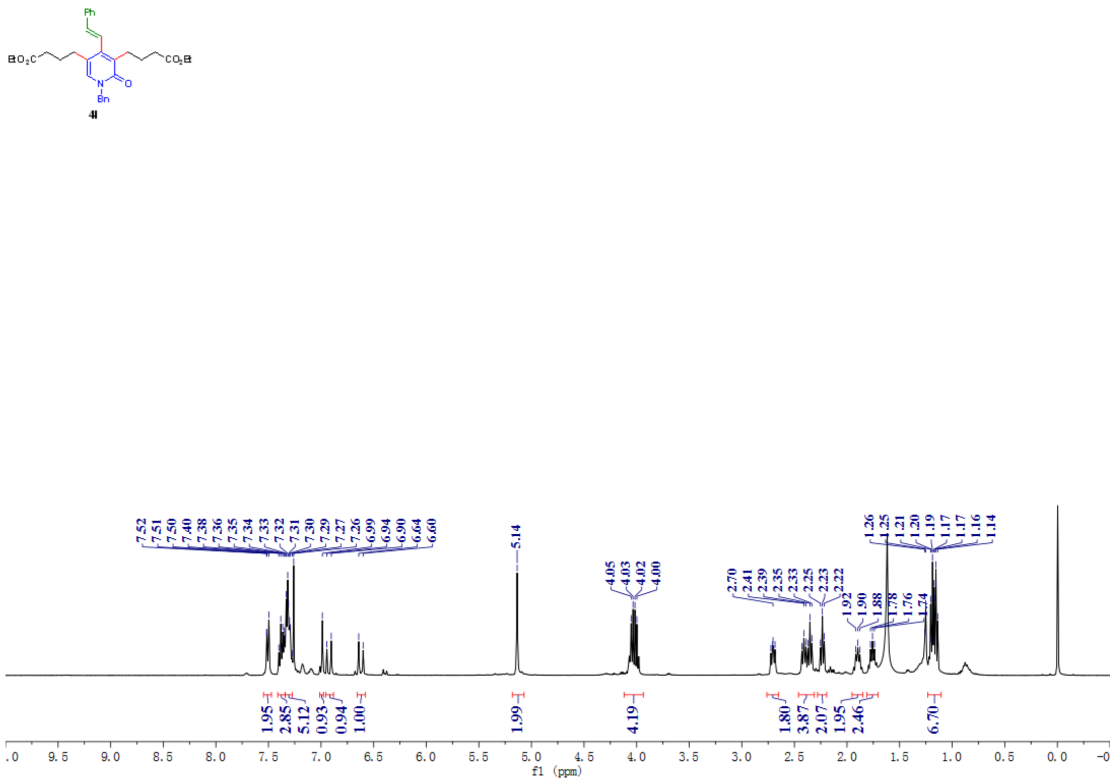

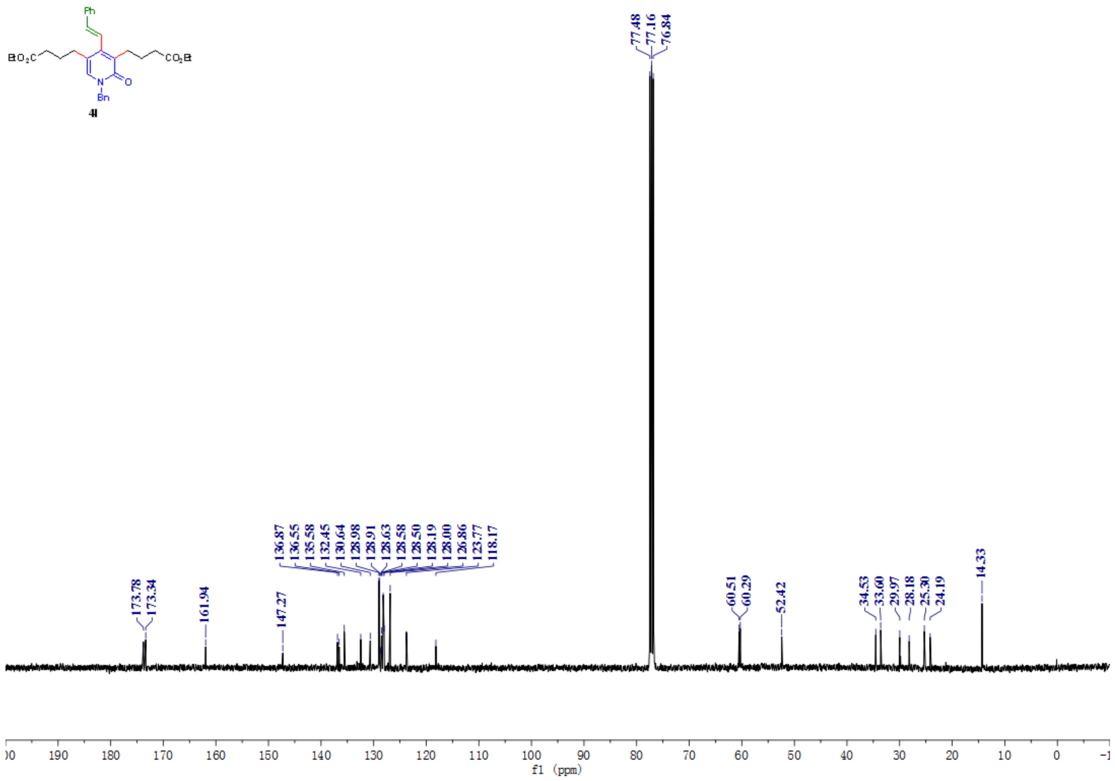

Supplementary Figure 35. NMR of **4l**

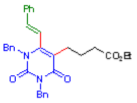

4m

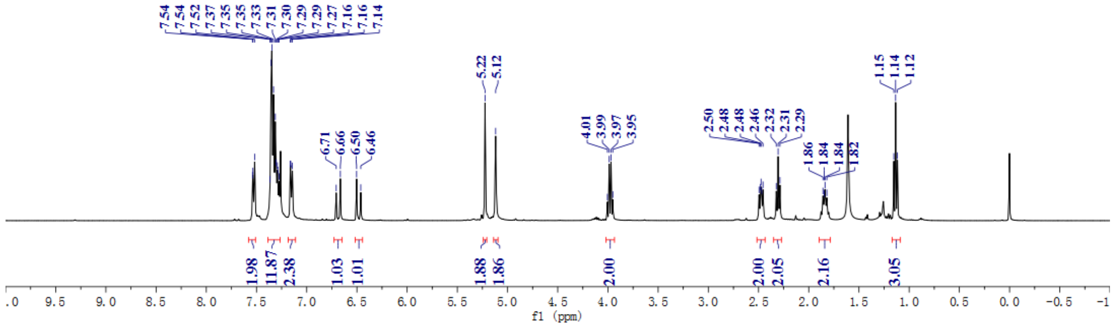

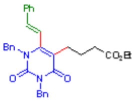

4m

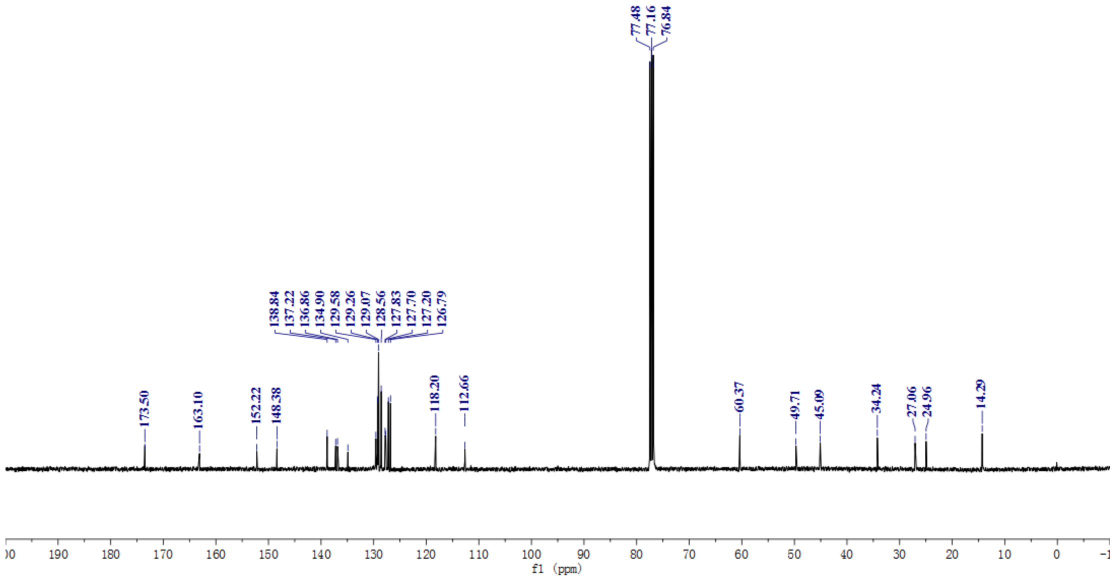

Supplementary Figure 36. NMR of **4m**

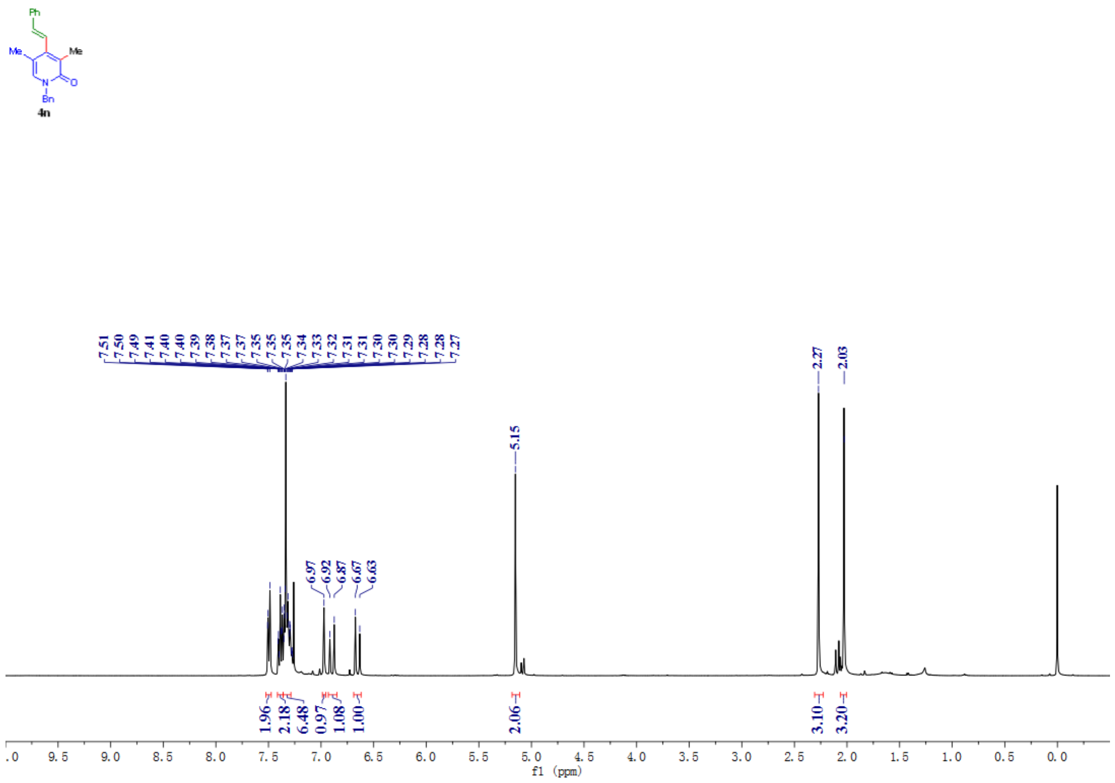

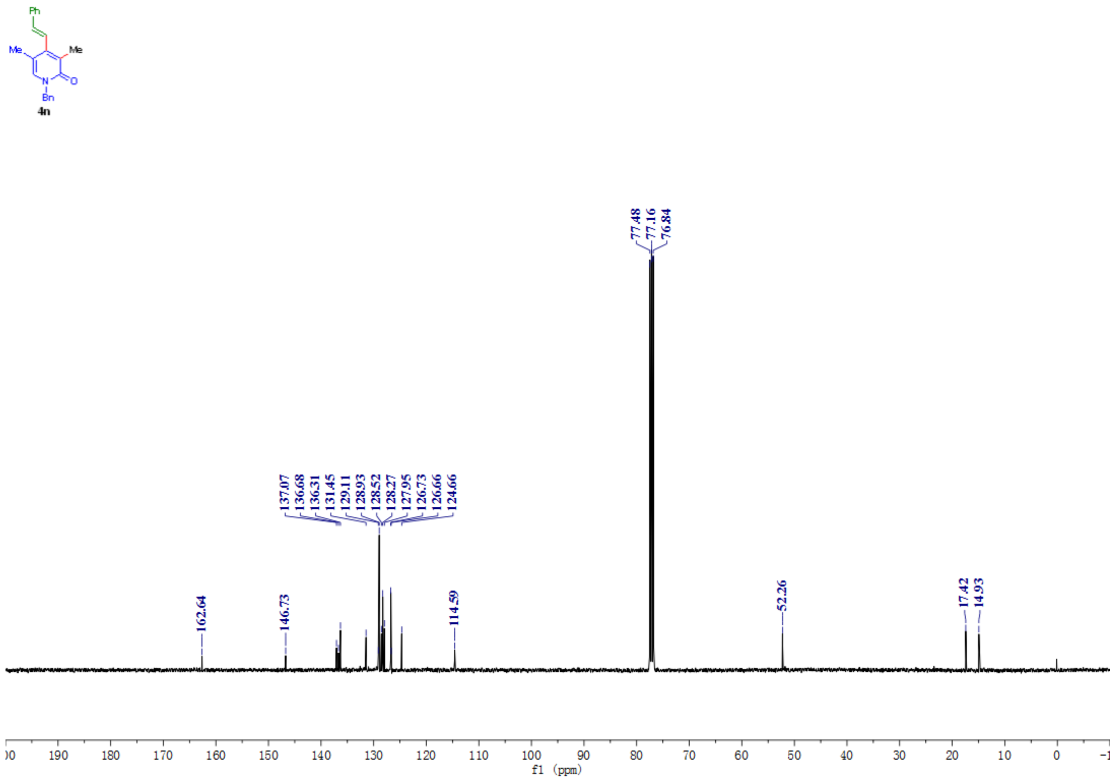

Supplementary Figure 37. NMR of **4n**

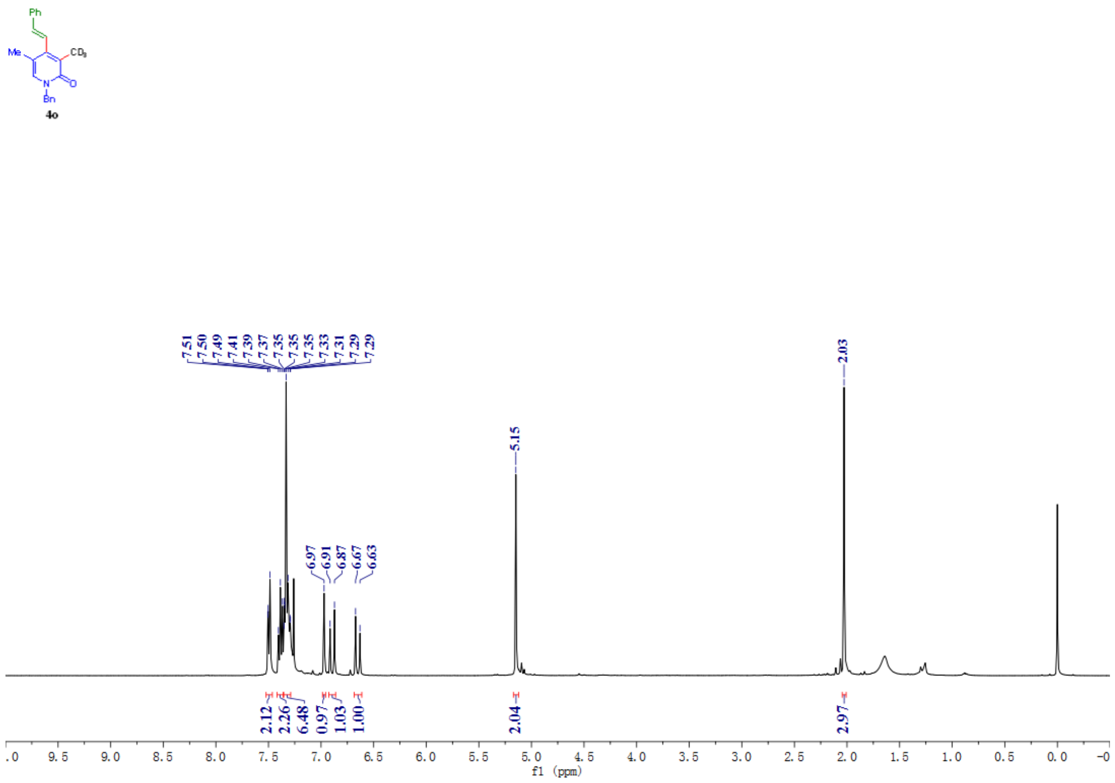

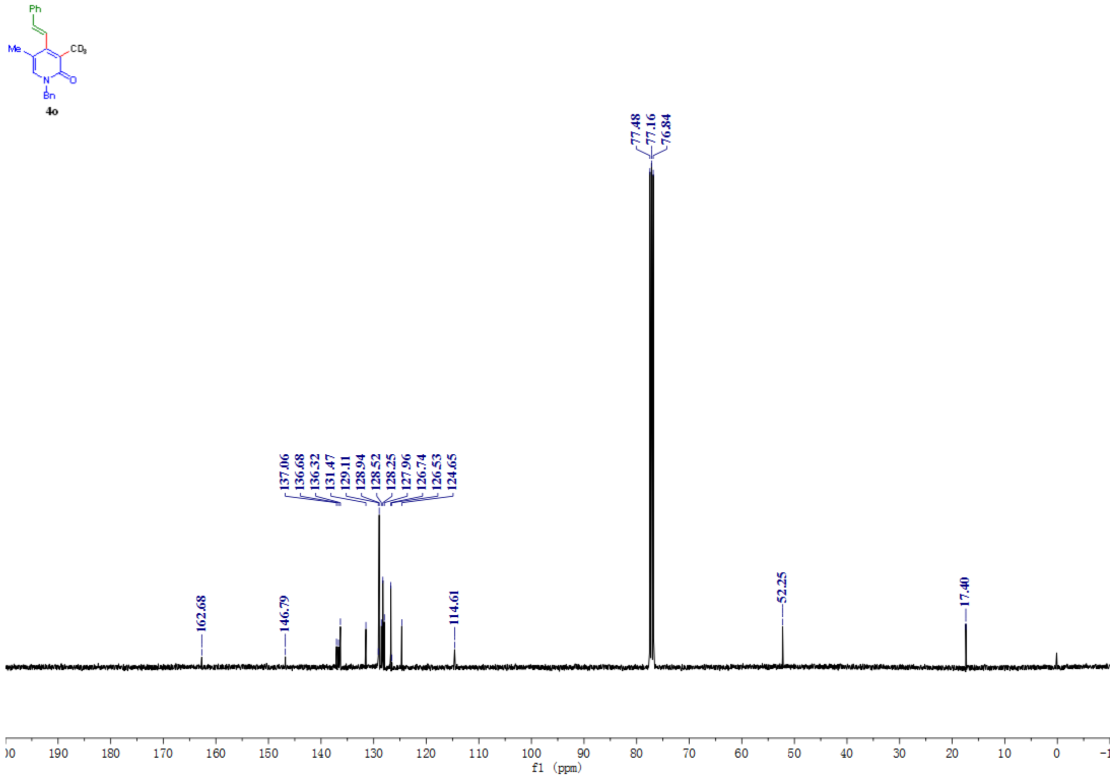

Supplementary Figure 38. NMR of **4o**

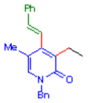

4p

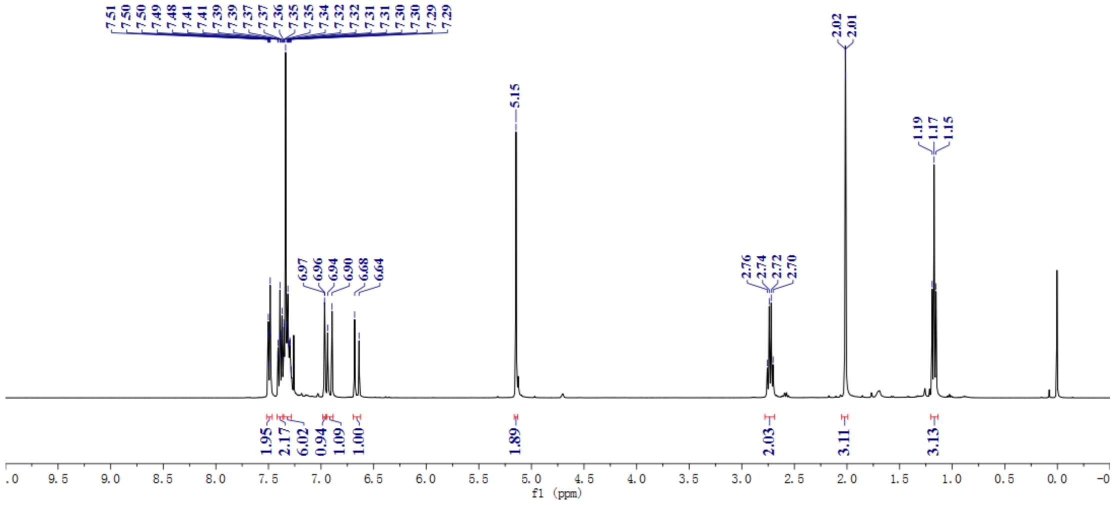

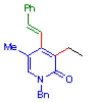

4p

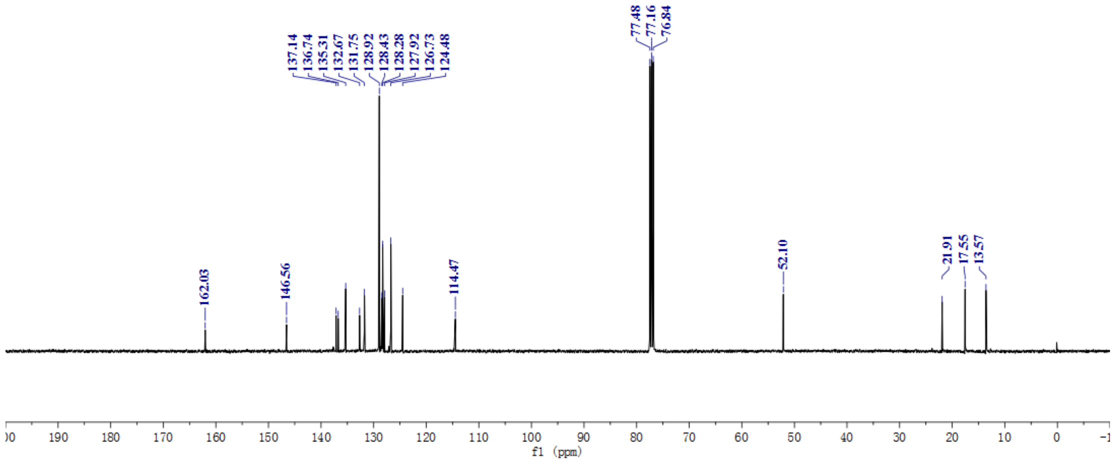

Supplementary Figure 39. NMR of **4p**

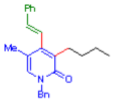

4q

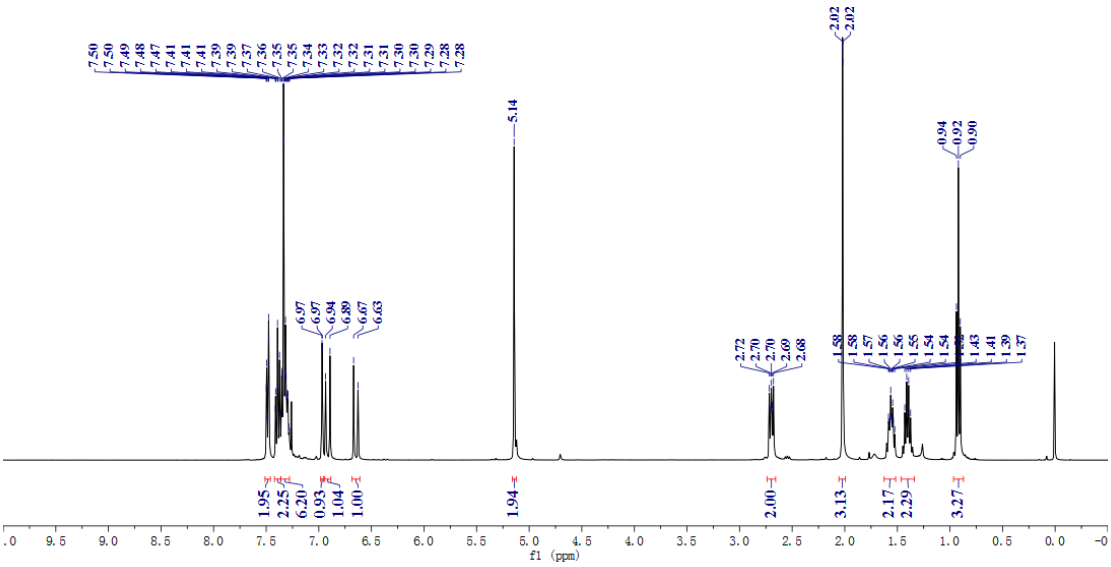

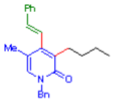

4q

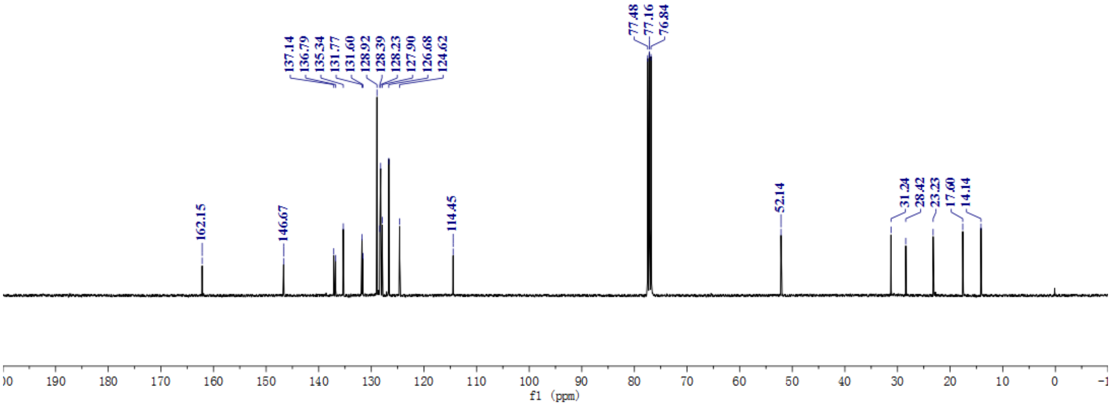

Supplementary Figure 40. NMR of **4q**

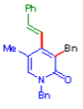

4r

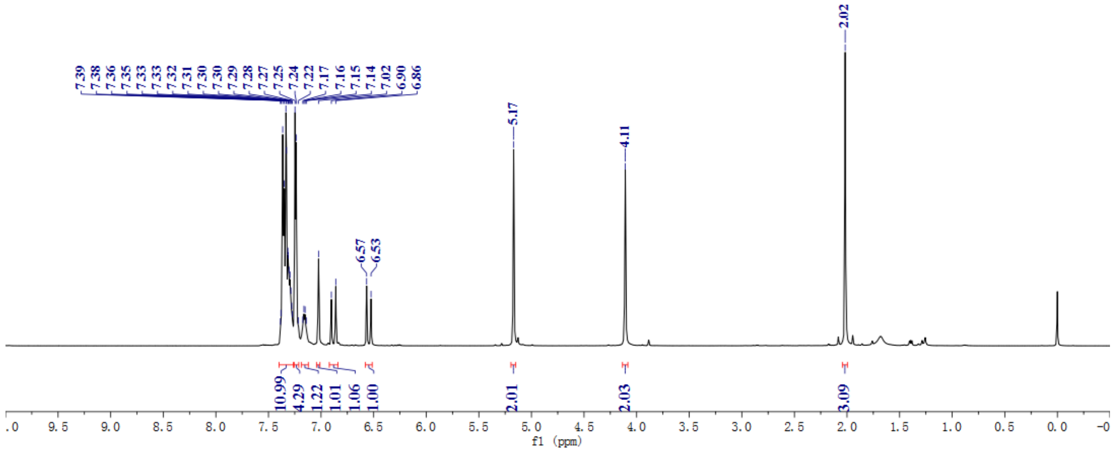

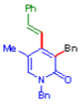

4r

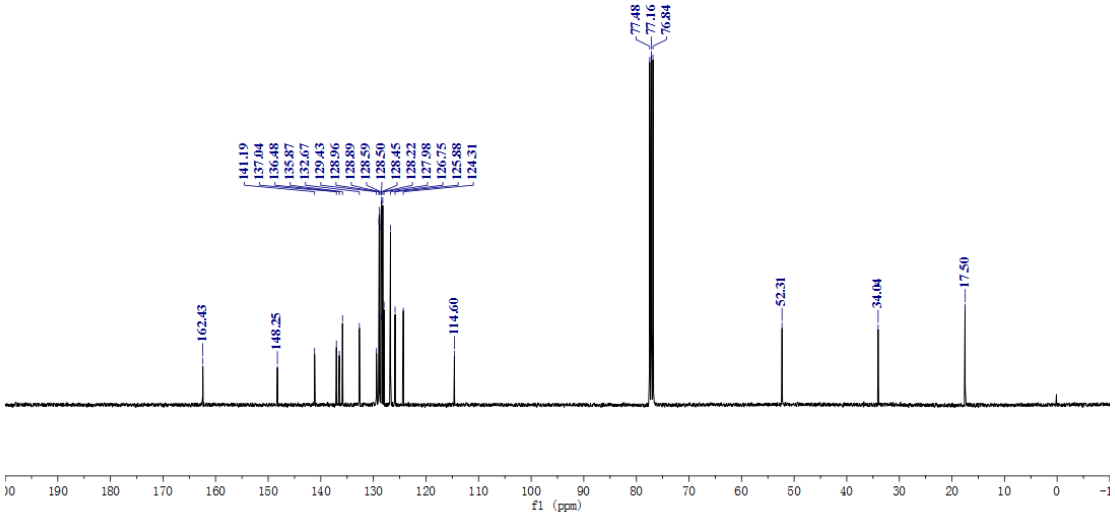

Supplementary Figure 41. NMR of **4r**

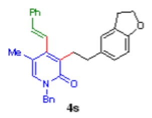

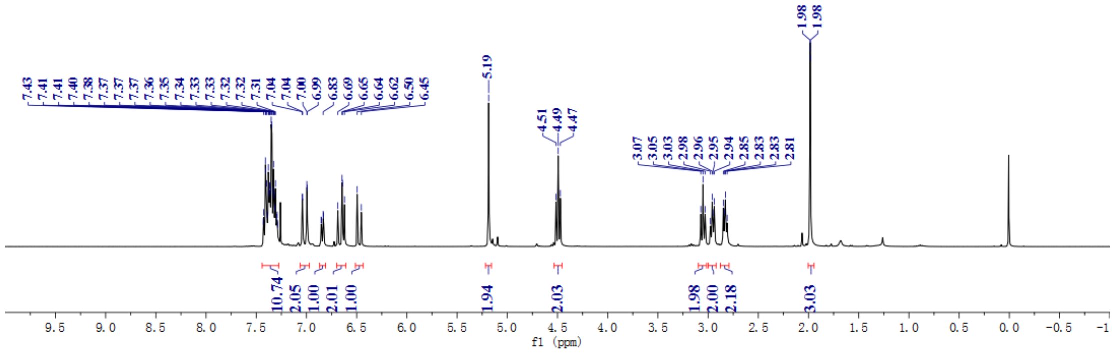

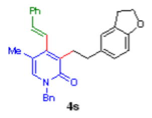

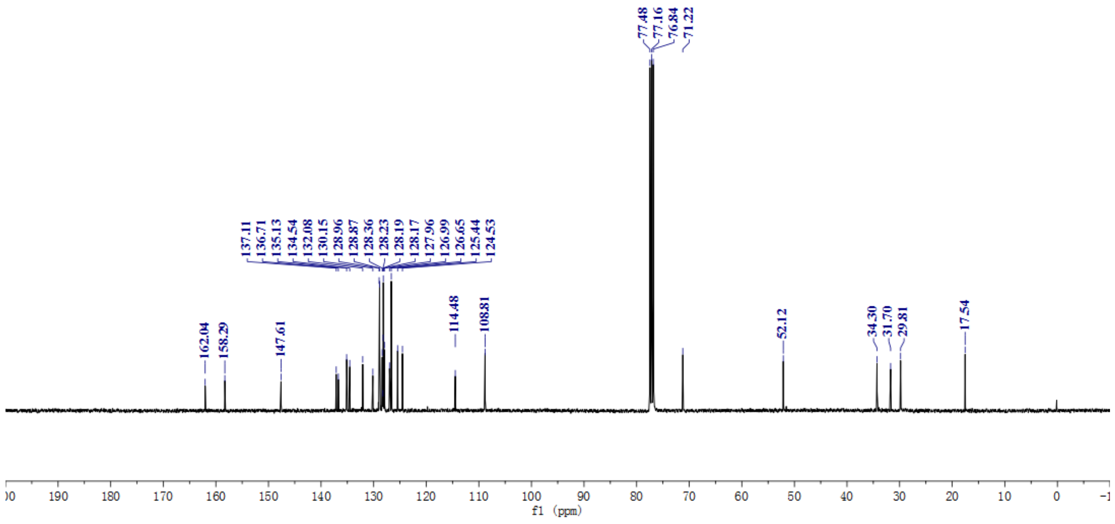

Supplementary Figure 42. NMR of **4s**

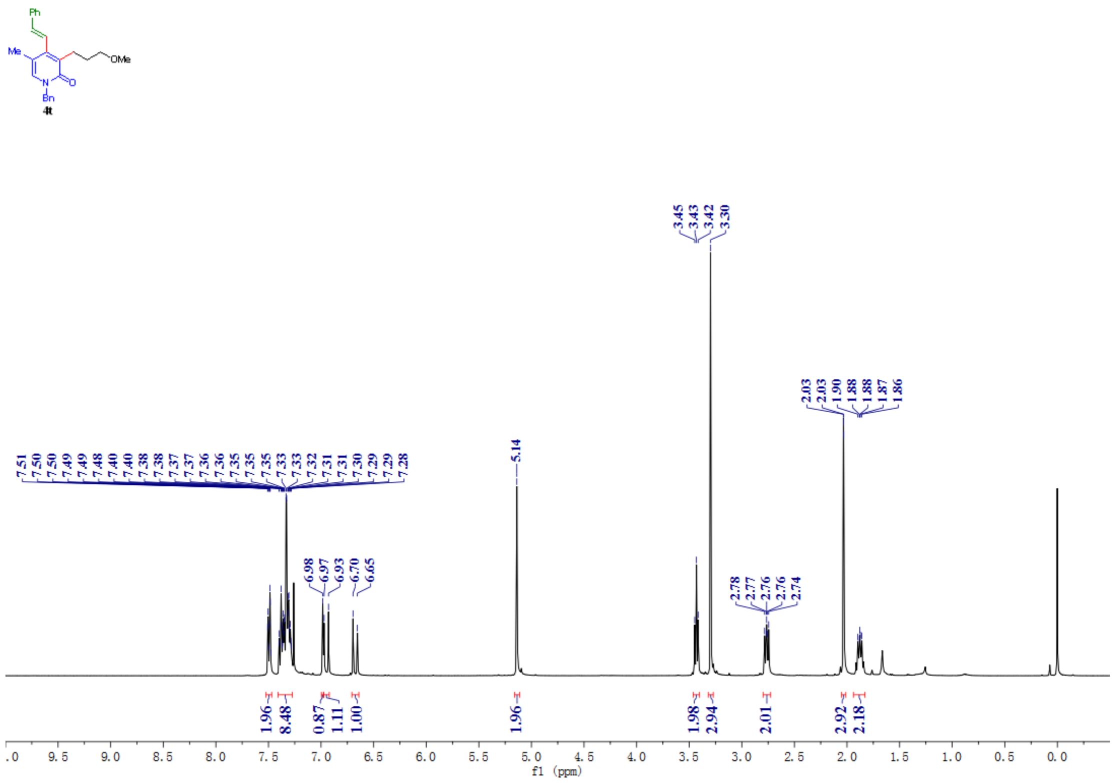

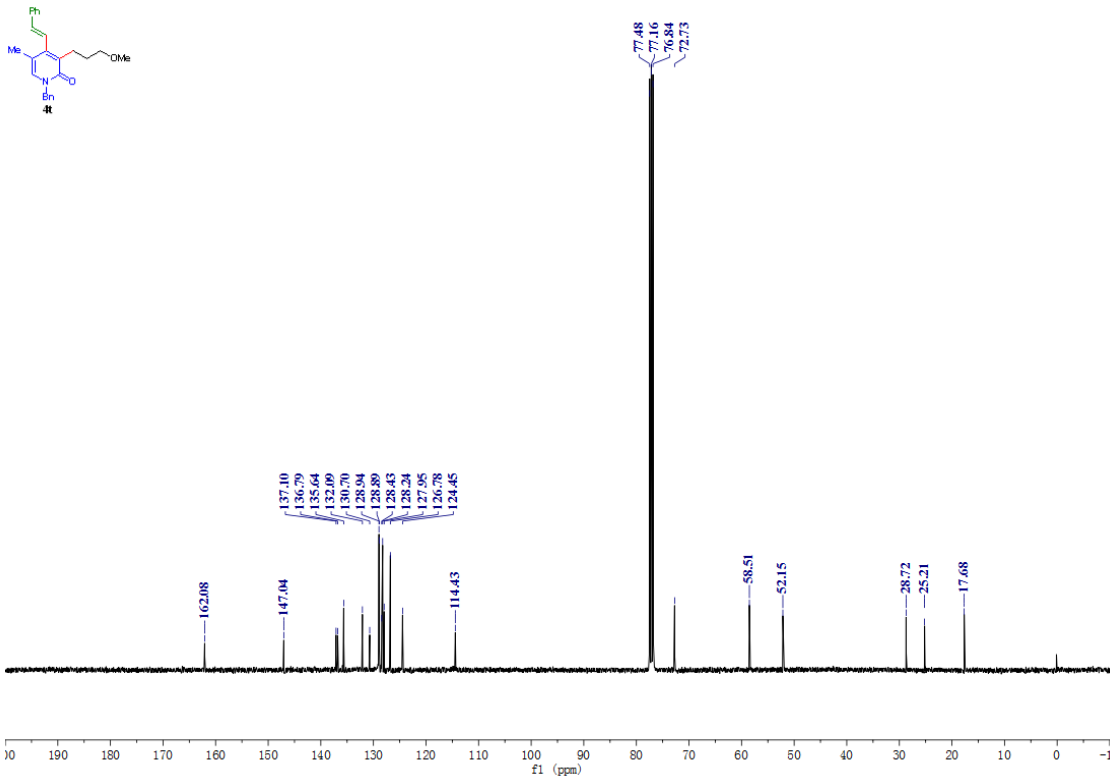

Supplementary Figure 43. NMR of **4t**

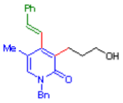

4u

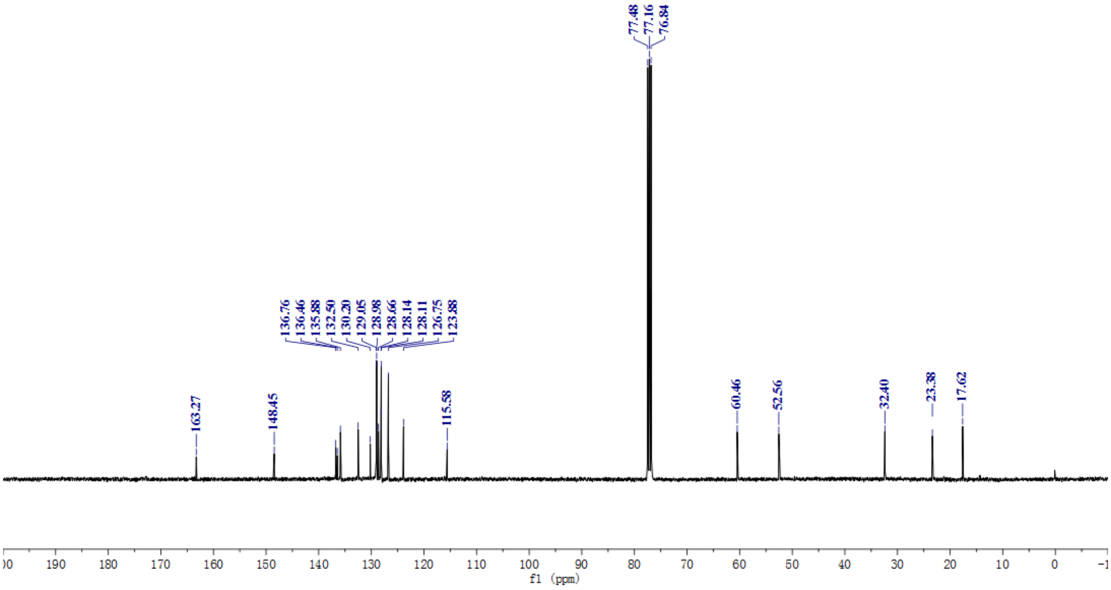

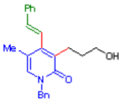

4u

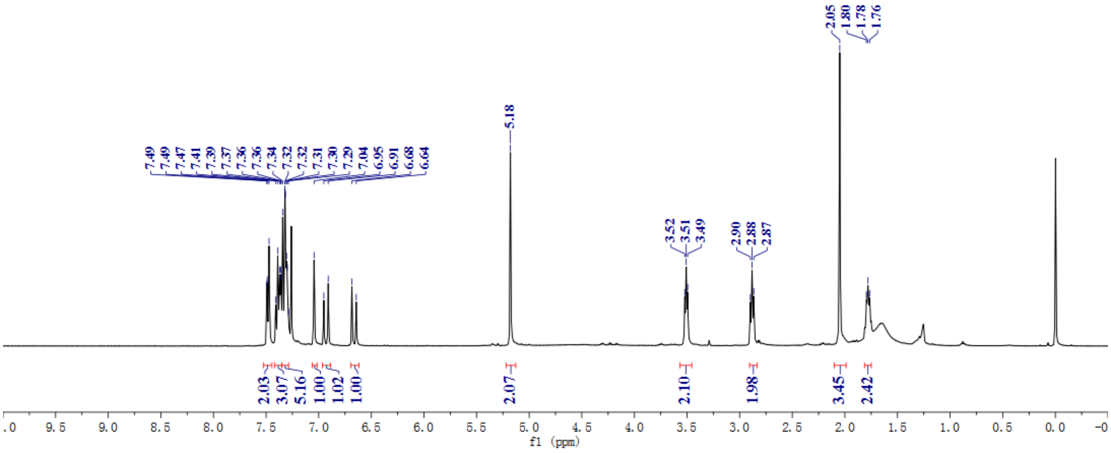

Supplementary Figure 44. NMR of **4u**

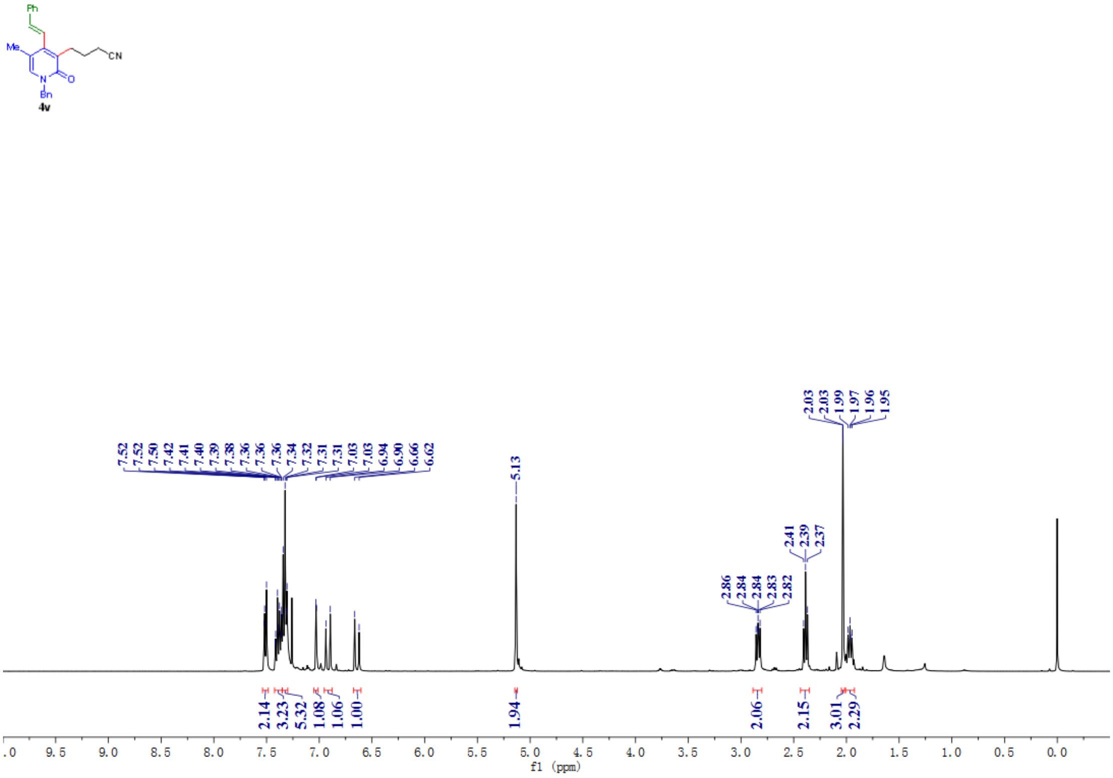

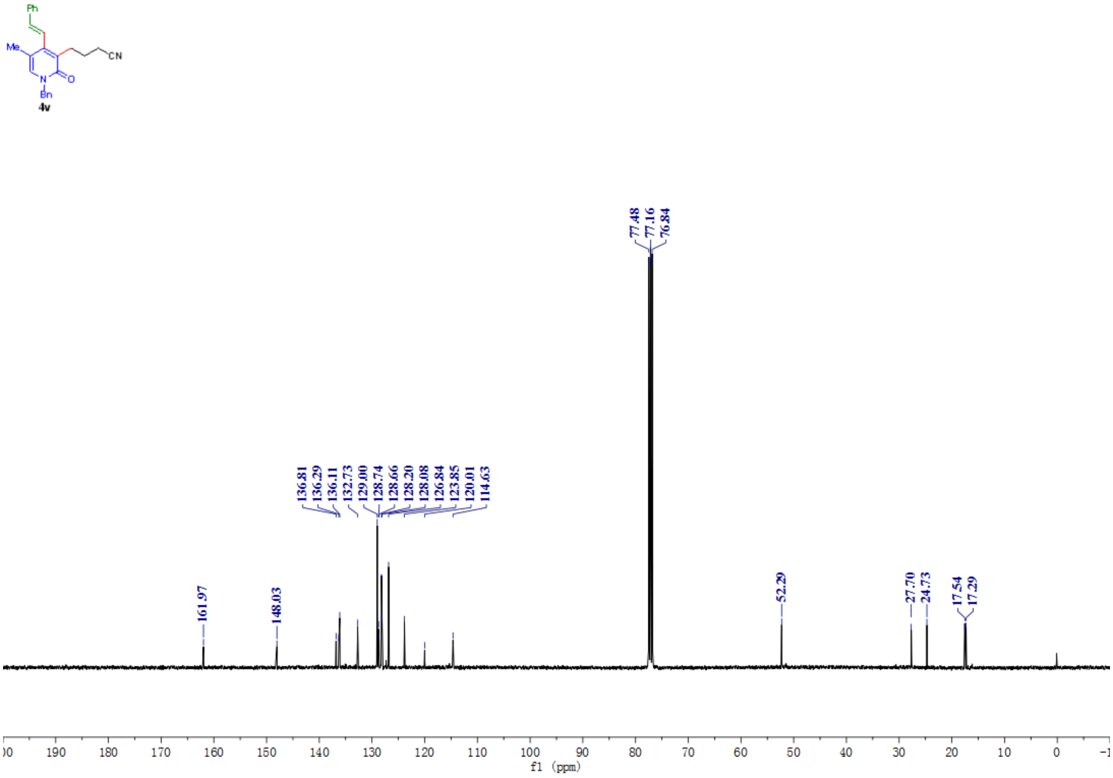

Supplementary Figure 45. NMR of **4v**

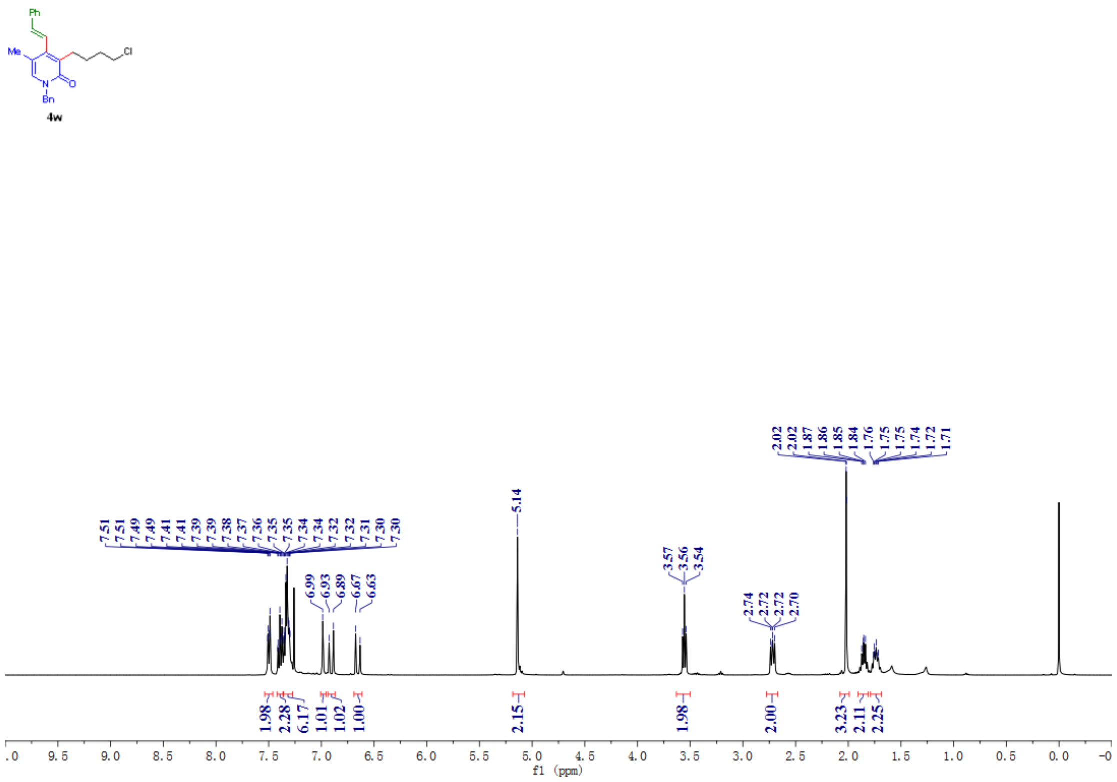

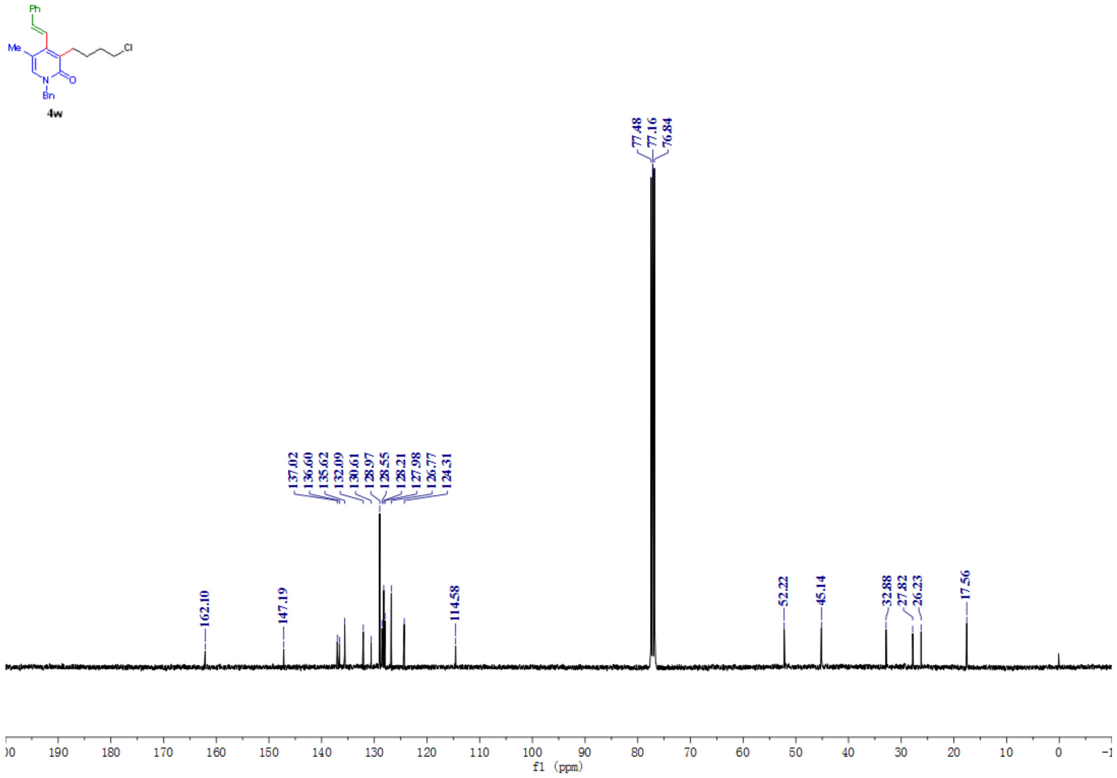

Supplementary Figure 46. NMR of **4w**

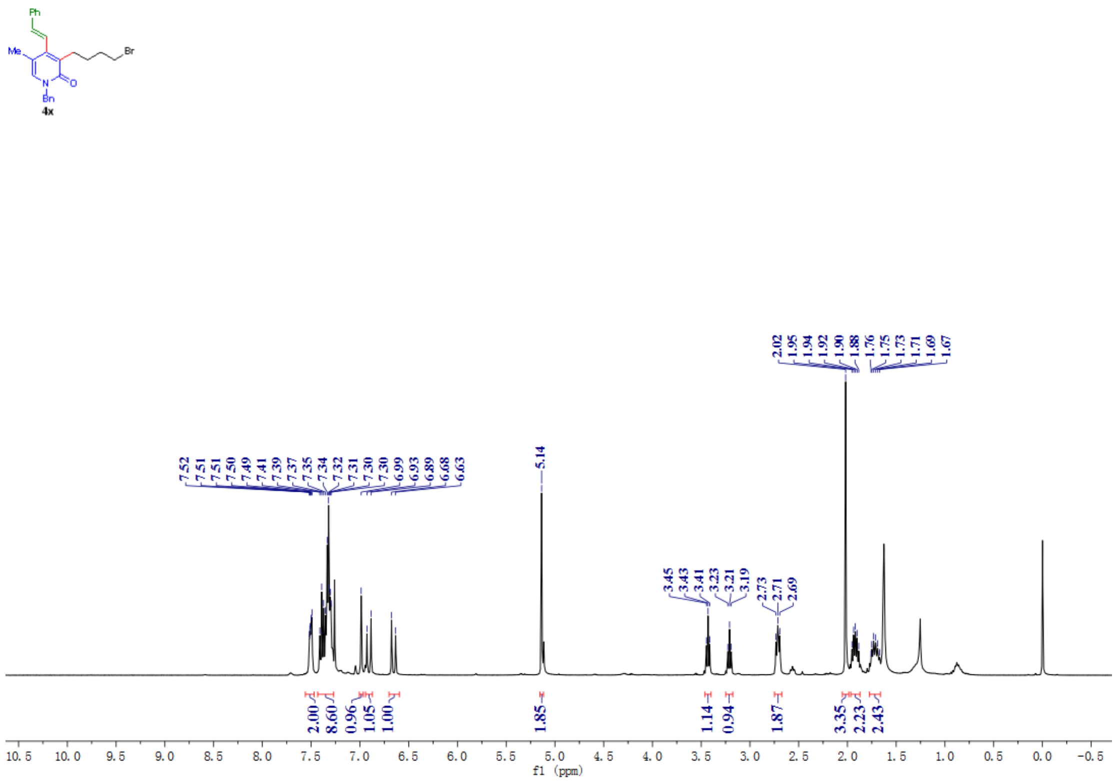

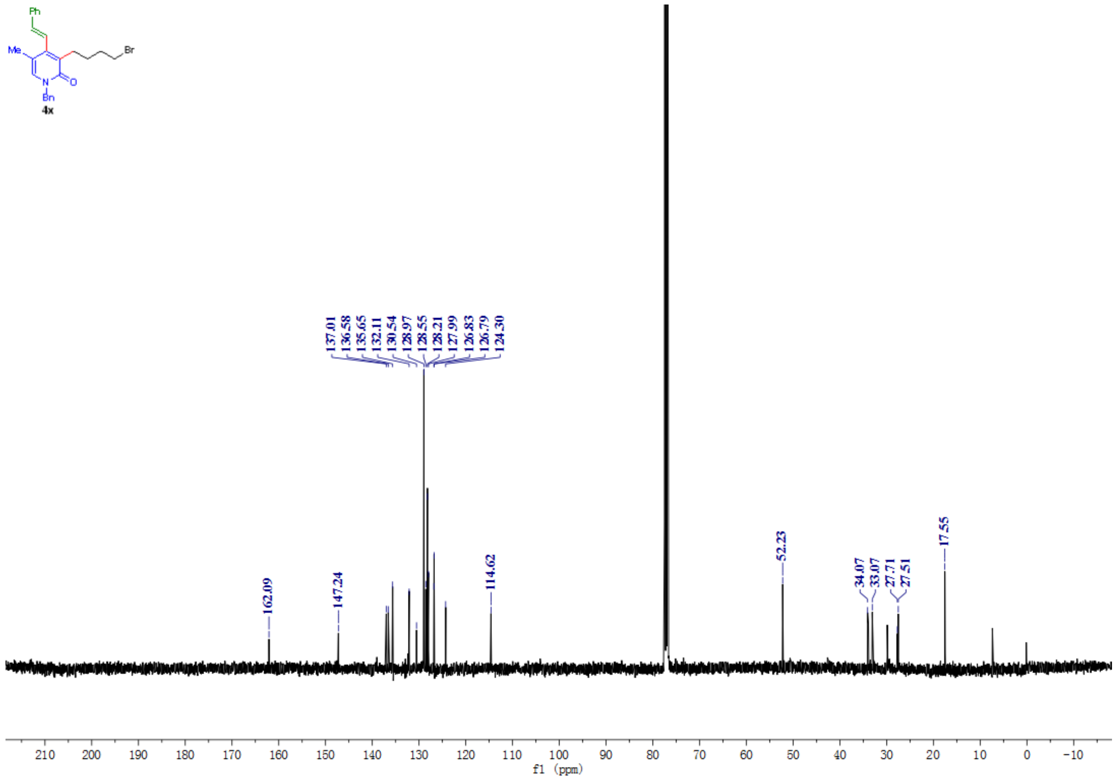

Supplementary Figure 47. NMR of **4x**

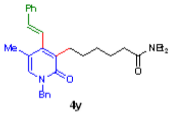

4y

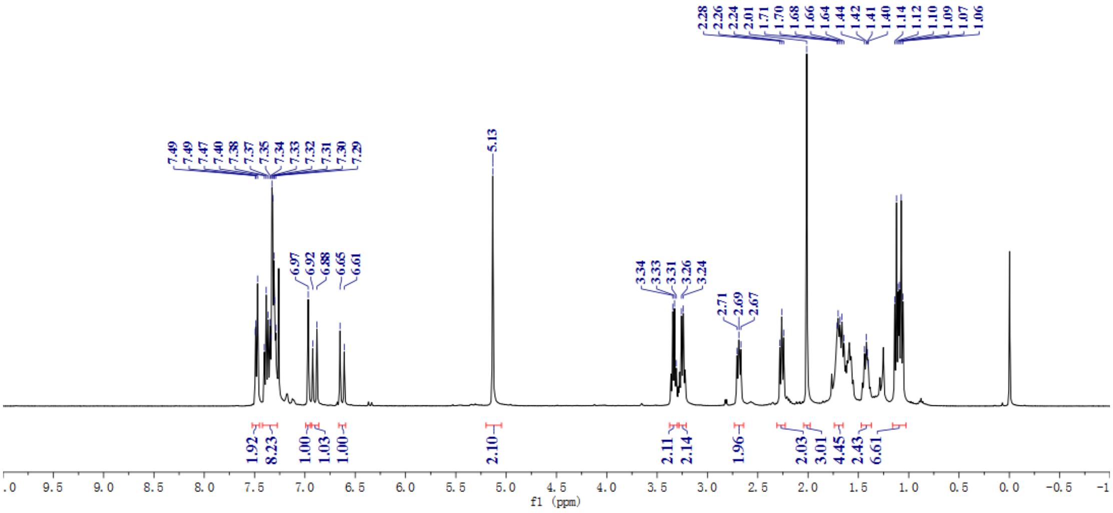

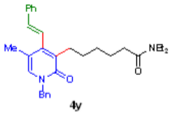

4y

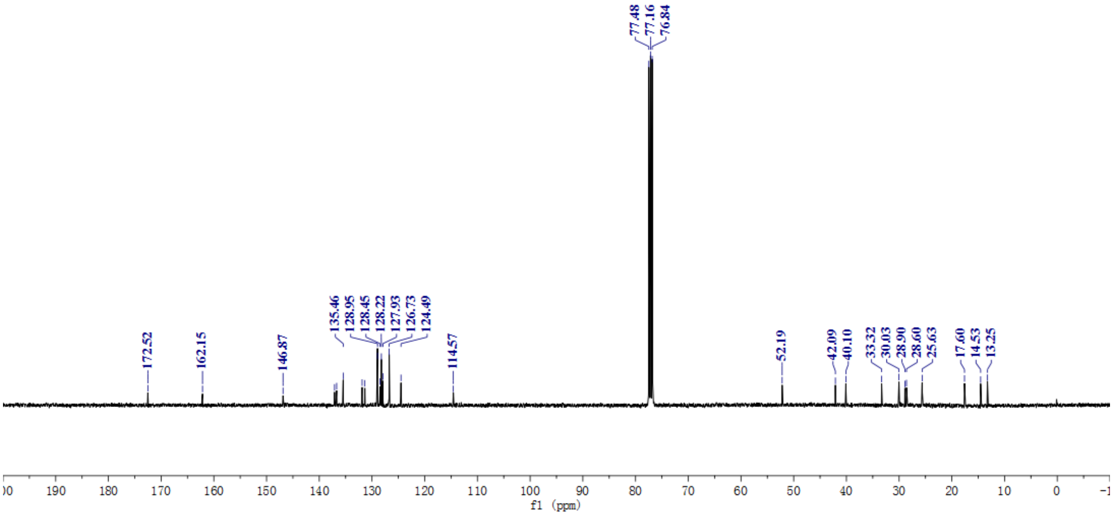

Supplementary Figure 48. NMR of **4y**

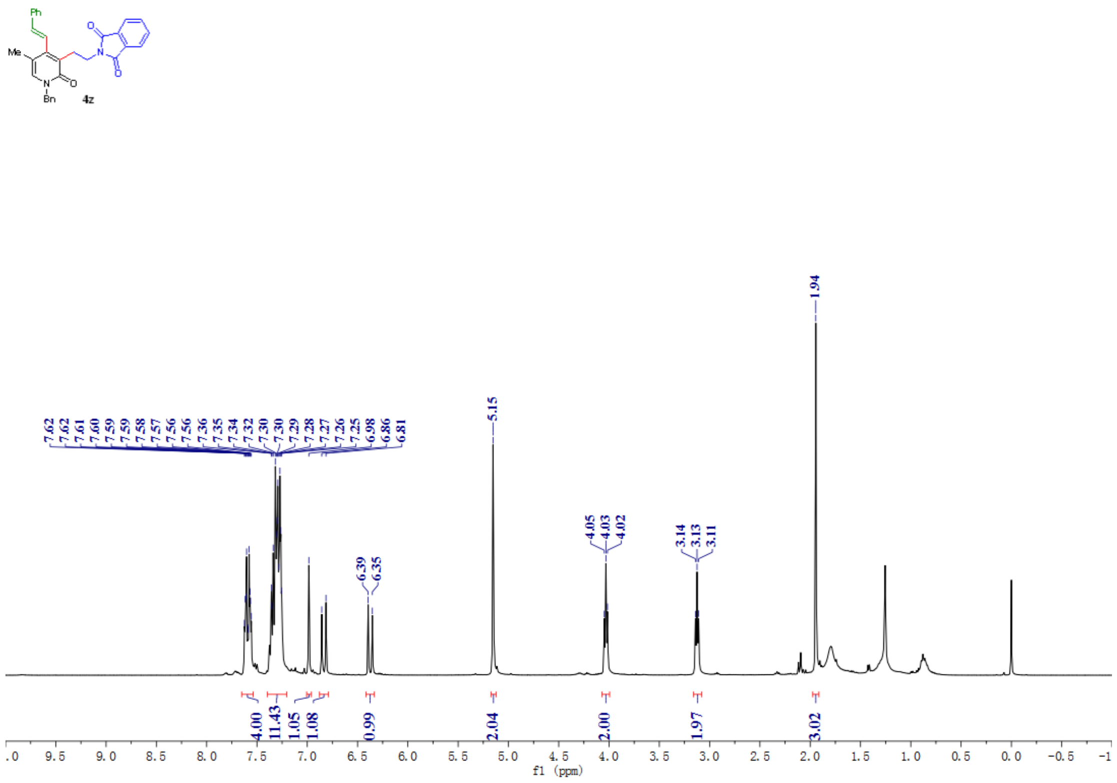

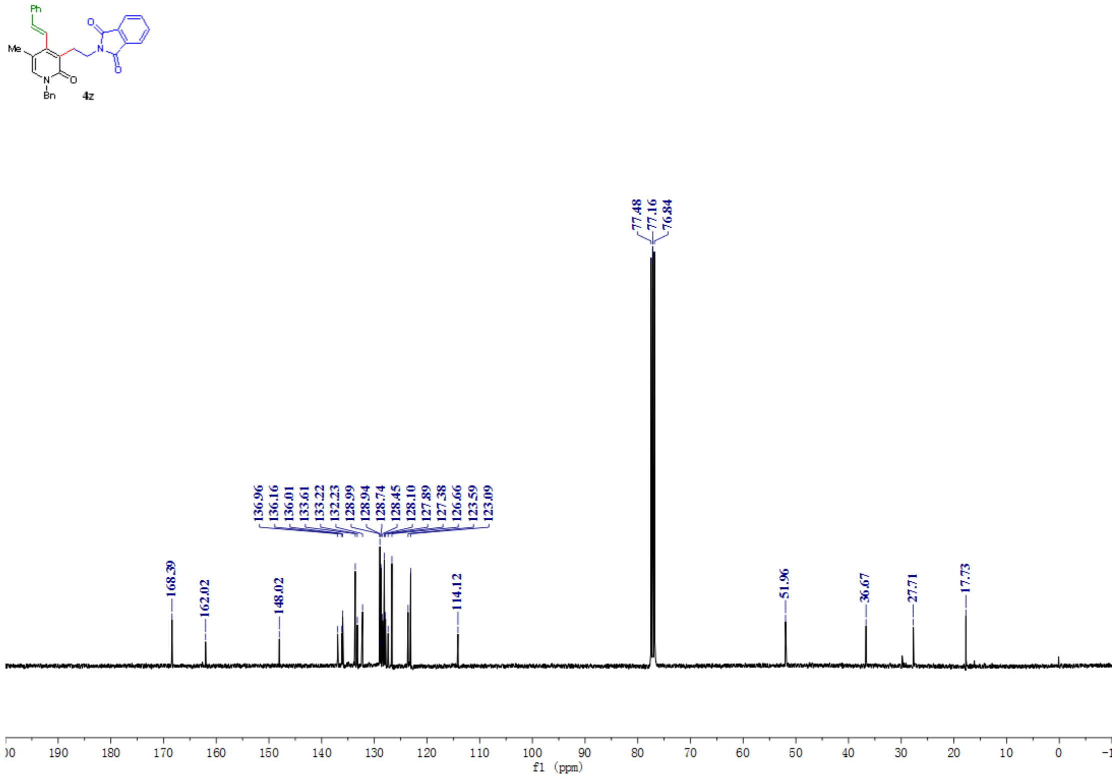

Supplementary Figure 49. NMR of **4z**

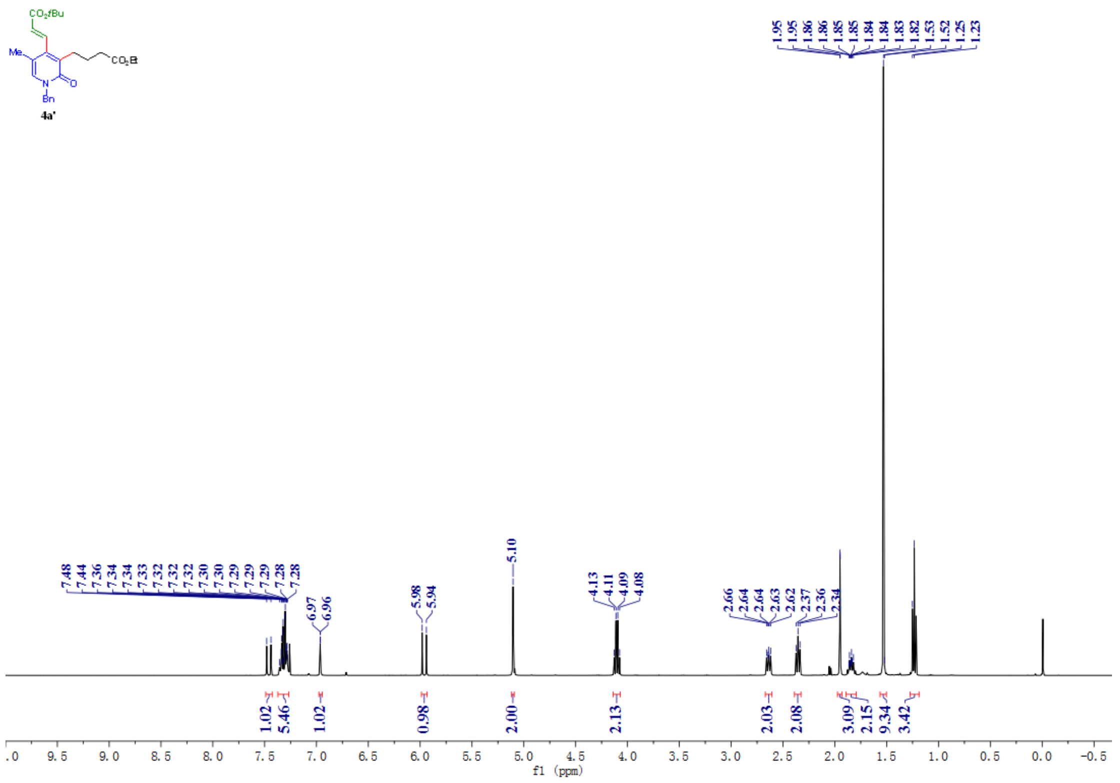

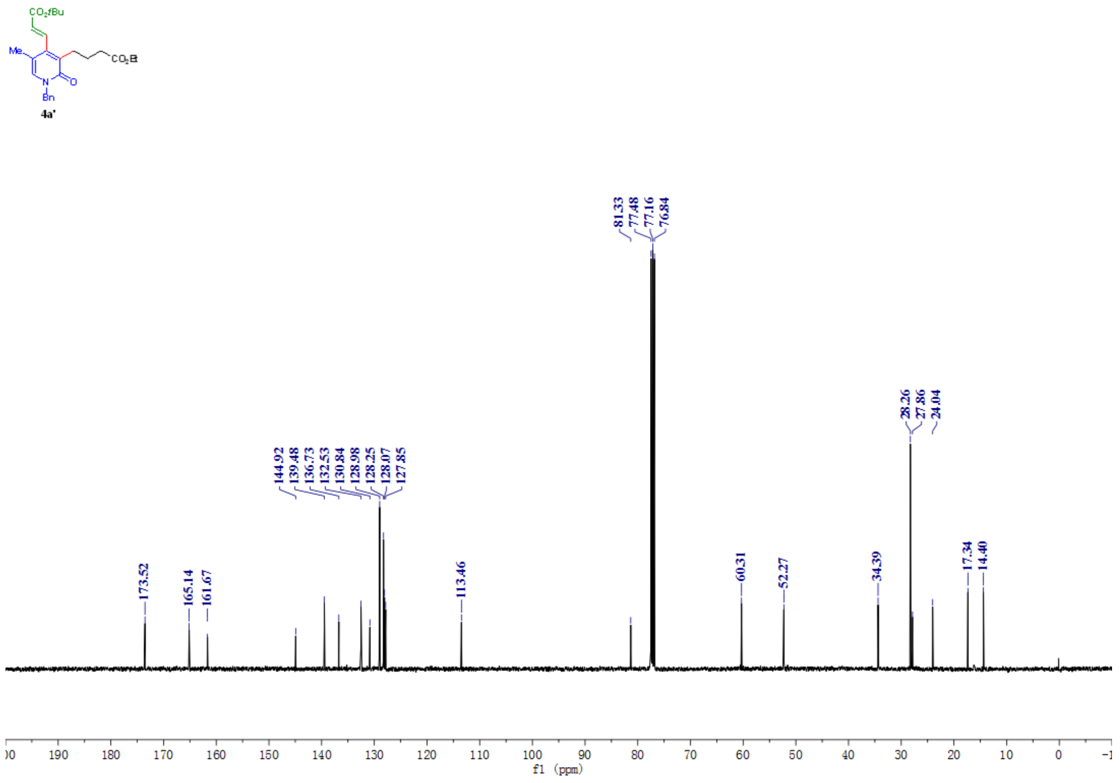

Supplementary Figure 50. NMR of **4a'**

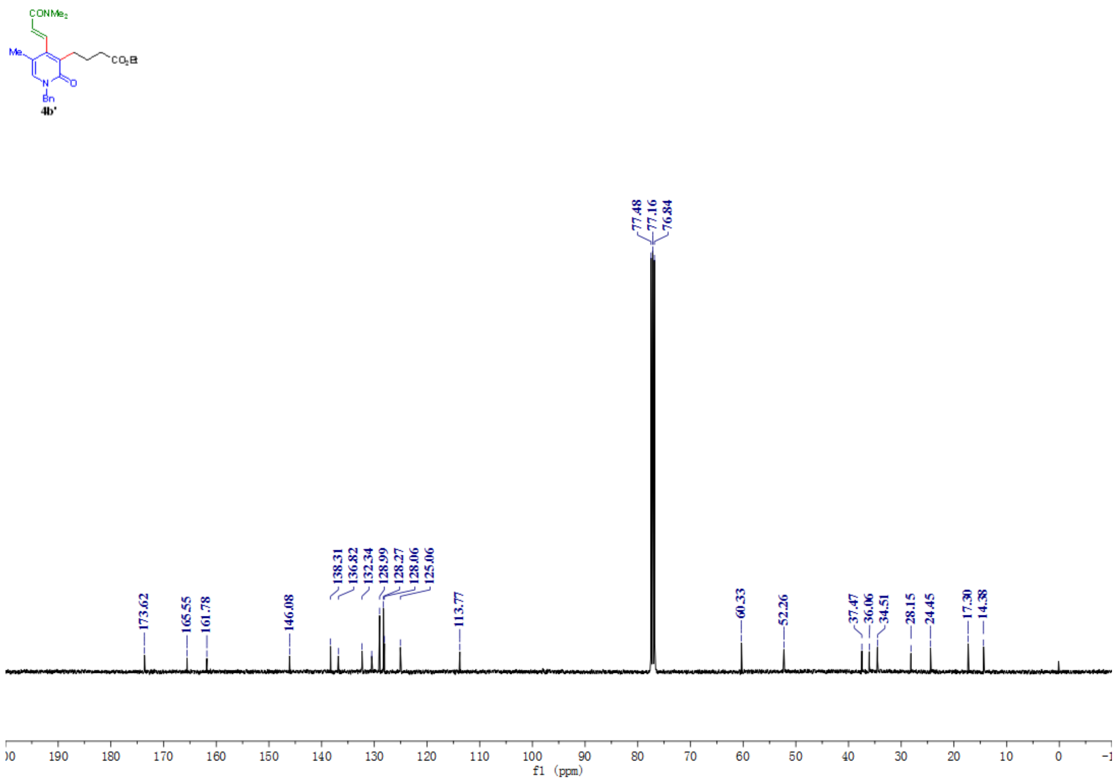

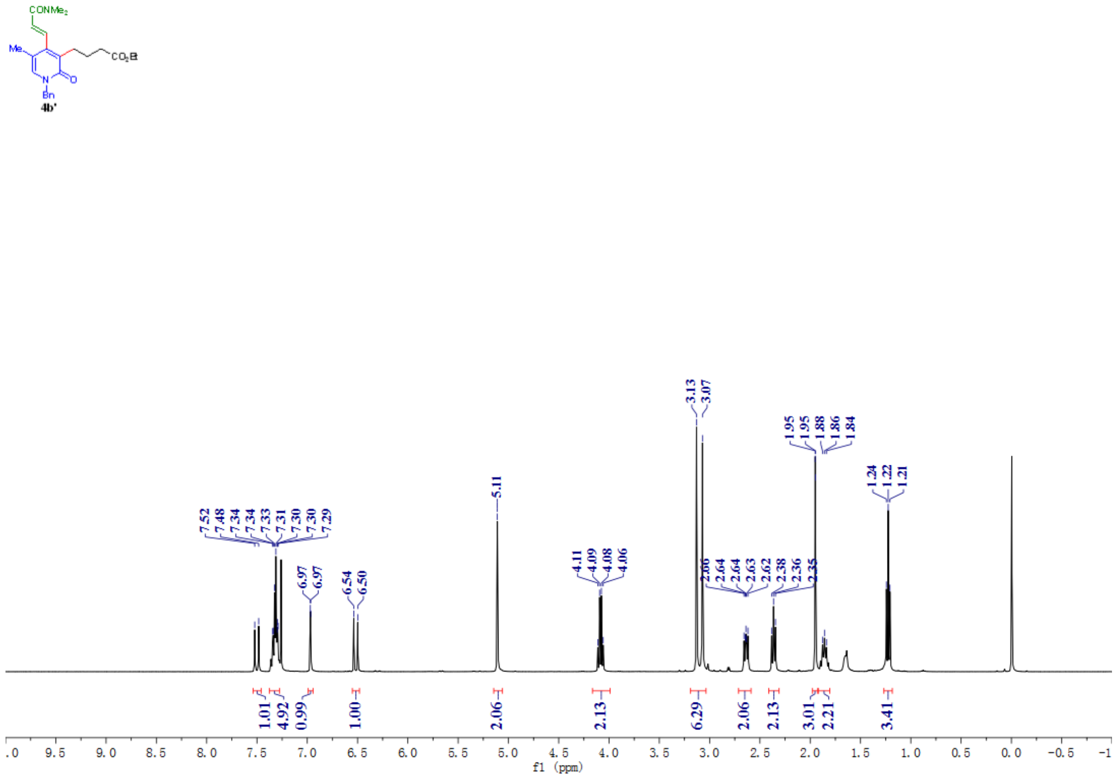

Supplementary Figure 51. NMR of **4b'**

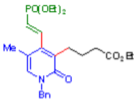
PO(OEt)2
Me
CO2Et
N
Bn
O

4c'

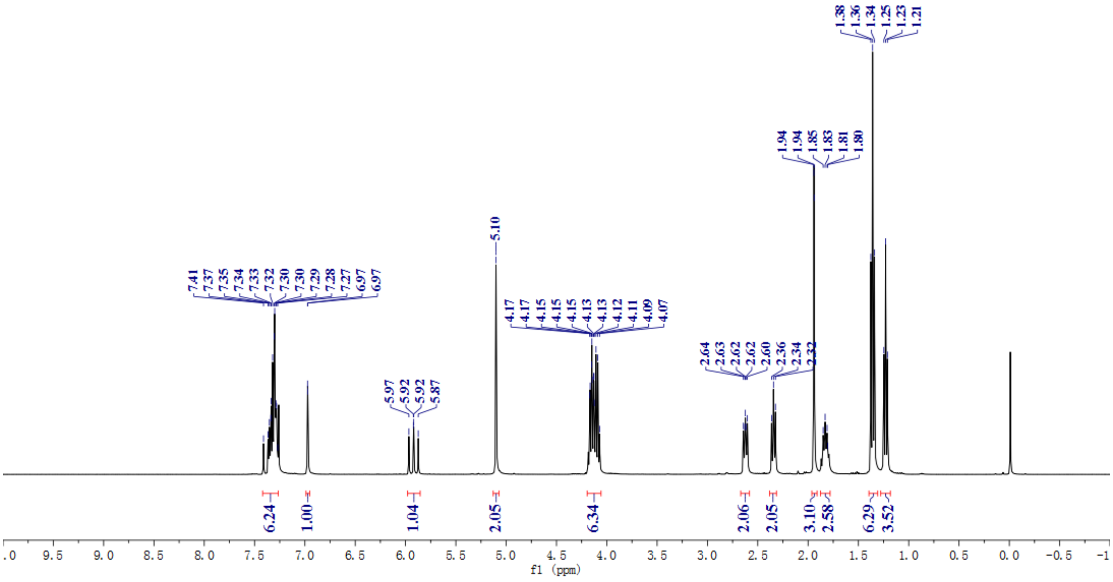
7.41 7.37 7.35 7.34 7.33 7.32 7.30 7.30 7.29 7.28 7.27 6.97 6.97
5.97 5.92 5.92 5.87
5.10
4.17 4.17 4.15 4.15 4.15 4.13 4.13 4.12 4.11 4.09 4.07
2.64 2.63 2.62 2.62 2.60 2.36 2.34 2.32
1.94 1.94 1.85 1.83 1.81 1.80
1.38 1.36 1.34 1.25 1.23 1.21
6.24 1.00 1.04 2.05 6.34 2.06 2.05 3.10 2.58 6.29 3.52
f1 (ppm)

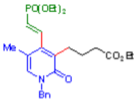
PO(OEt)2
Me
CO2Et
N
Bn
O

4c'

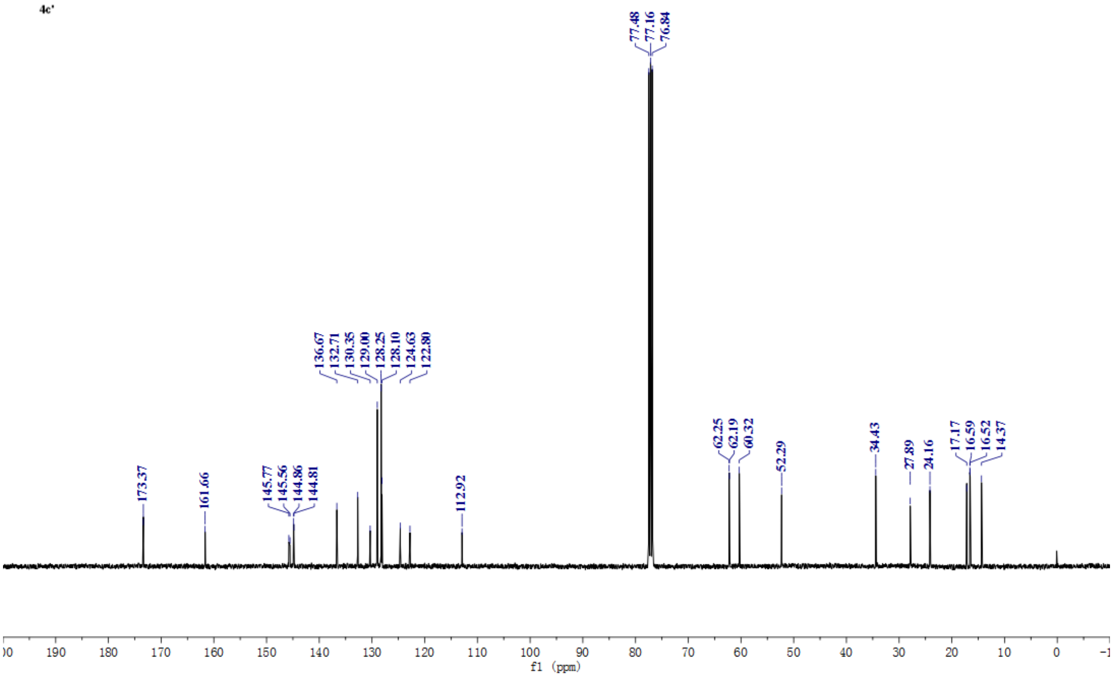
77.48 77.16 76.84
136.67 132.71 130.35 129.00 128.25 128.10 124.63 122.80
173.37
161.66
145.77 145.56 144.86 144.81
112.92
62.25 62.19 60.32
52.29
34.43
27.89
24.16
17.17 16.59 16.52 14.37
f1 (ppm)

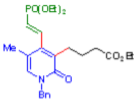

4c'

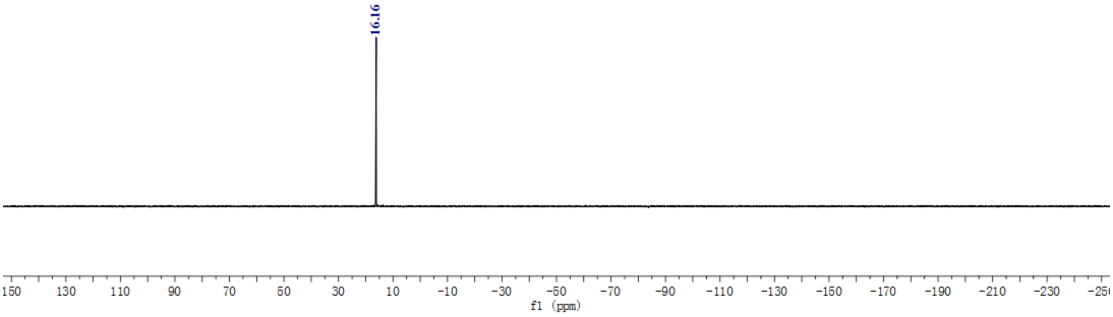

Supplementary Figure 52. NMR of **4c'**

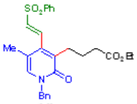

4d'

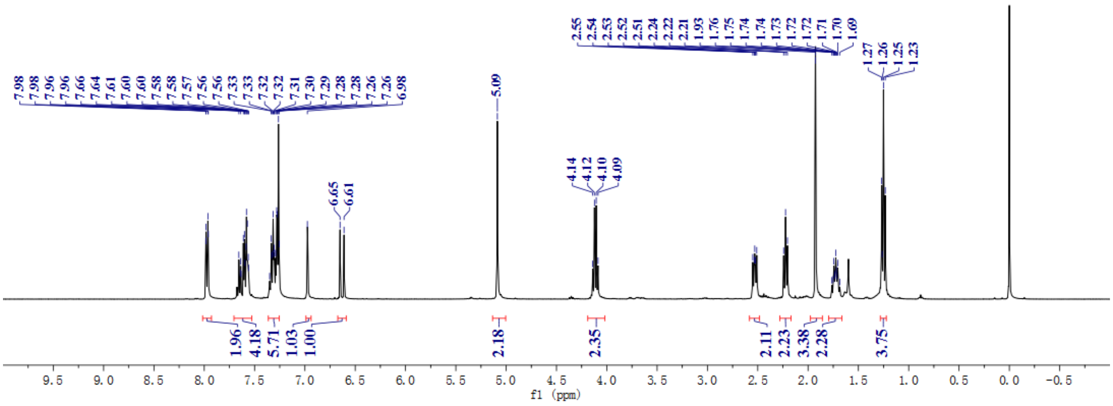

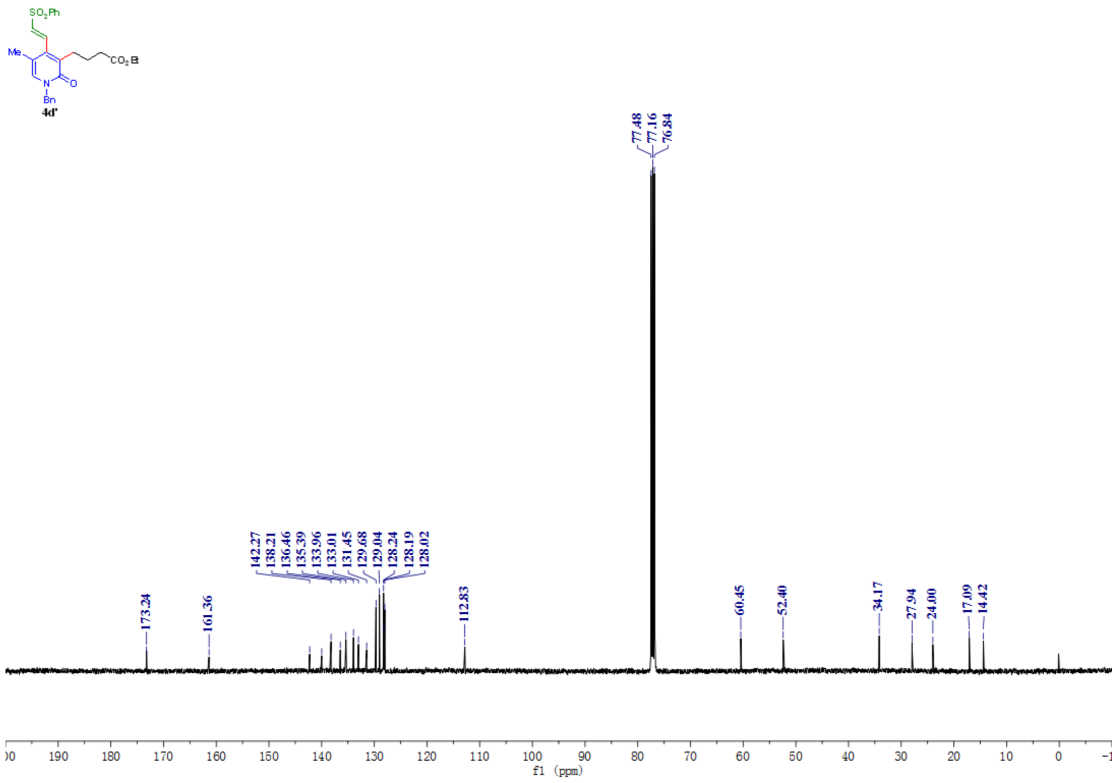

Supplementary Figure 53. NMR of **4d'**

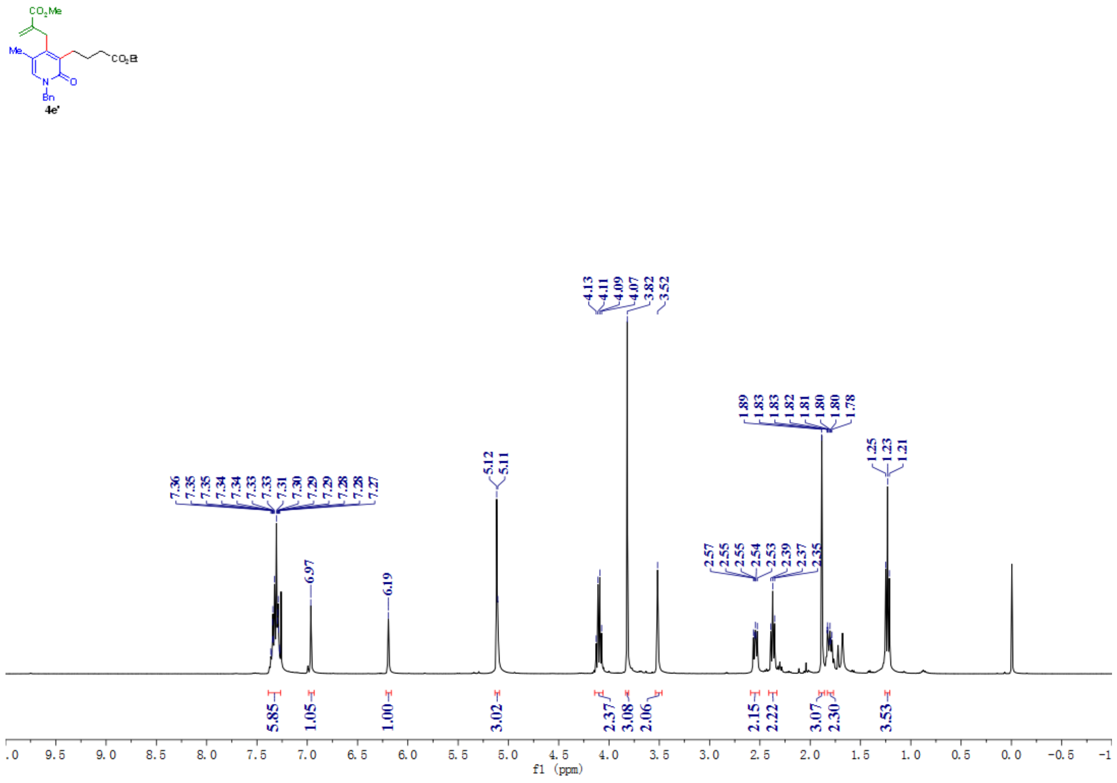

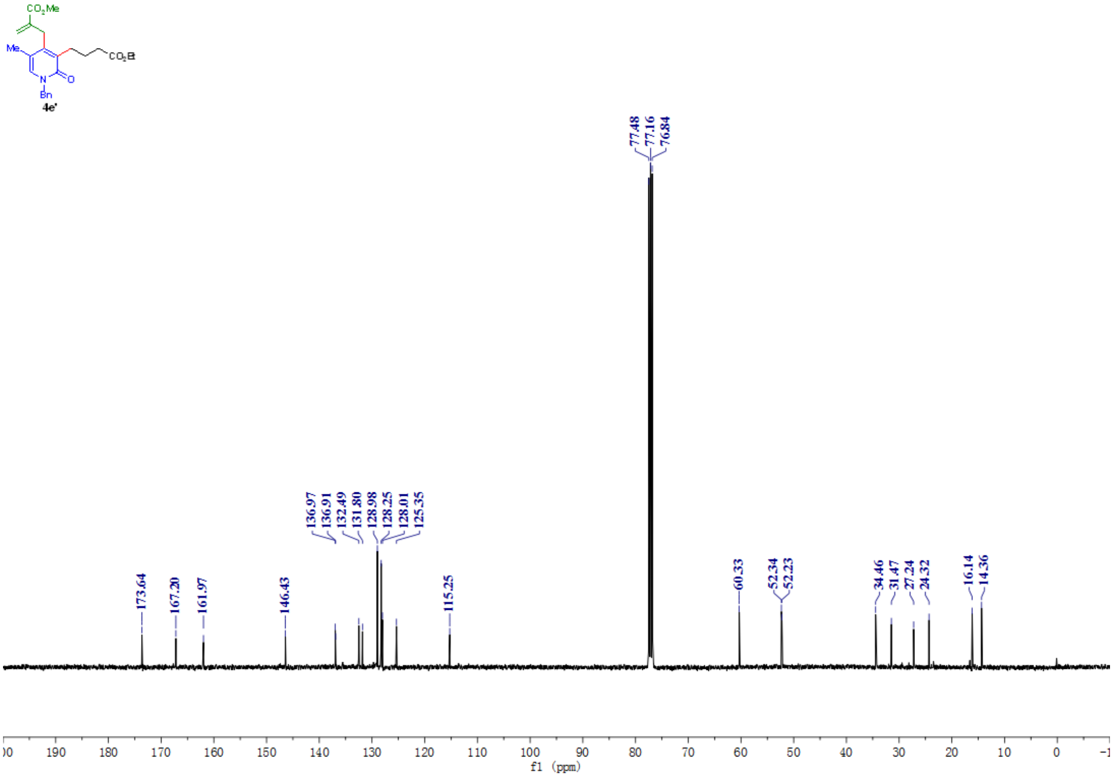

Supplementary Figure 54. NMR of **4e'**

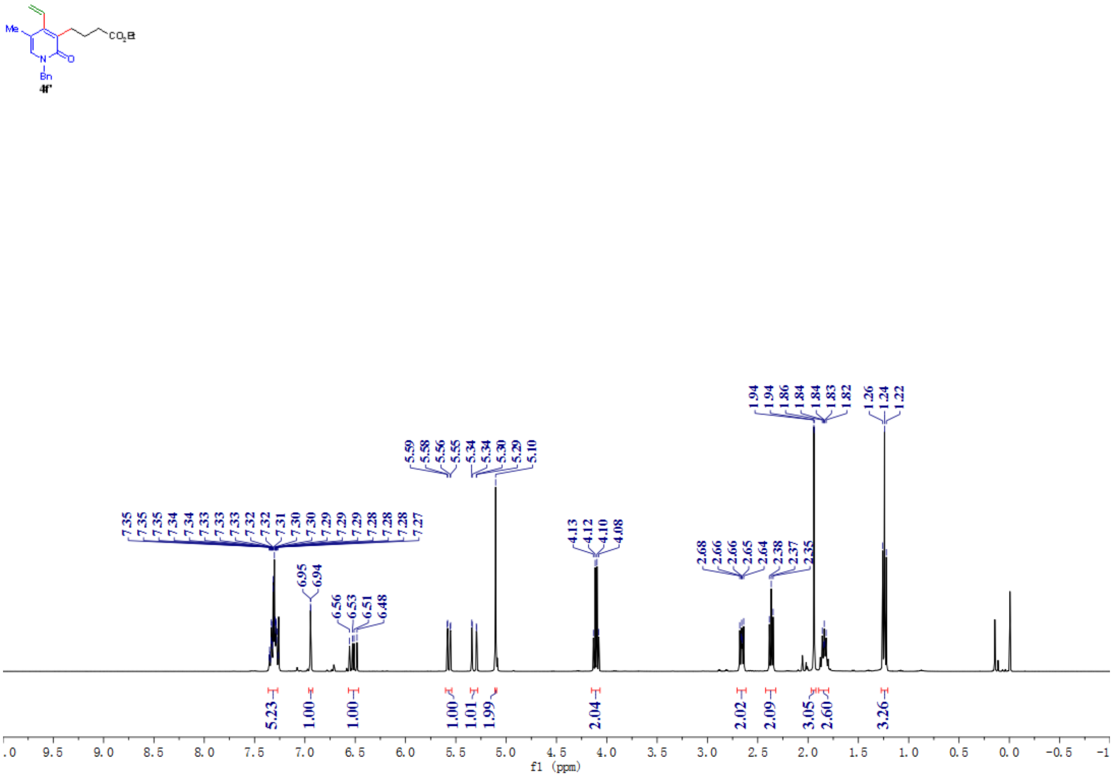

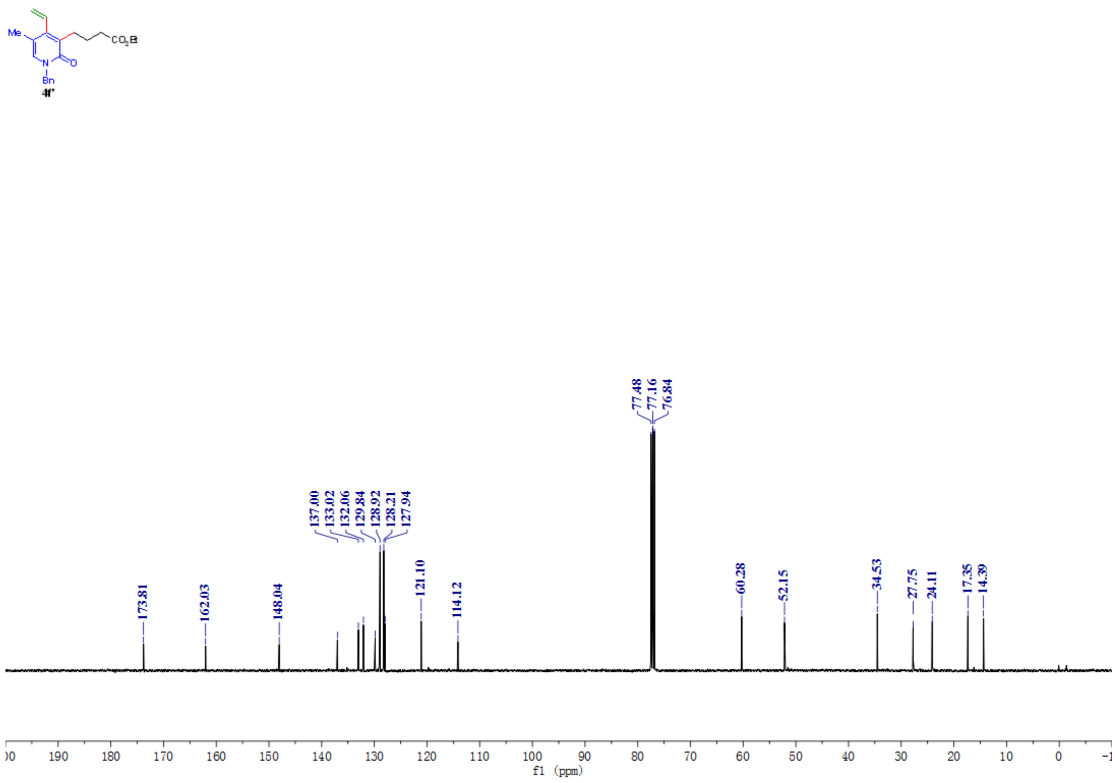

Supplementary Figure 55. NMR of **4f'**

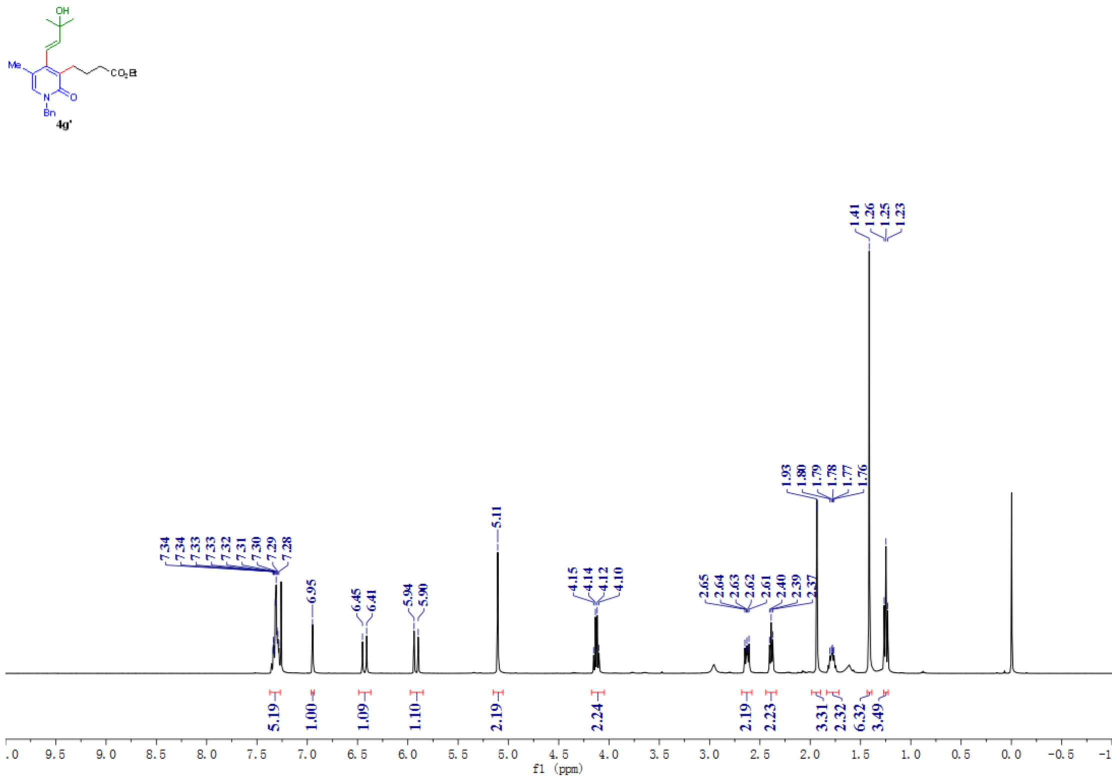

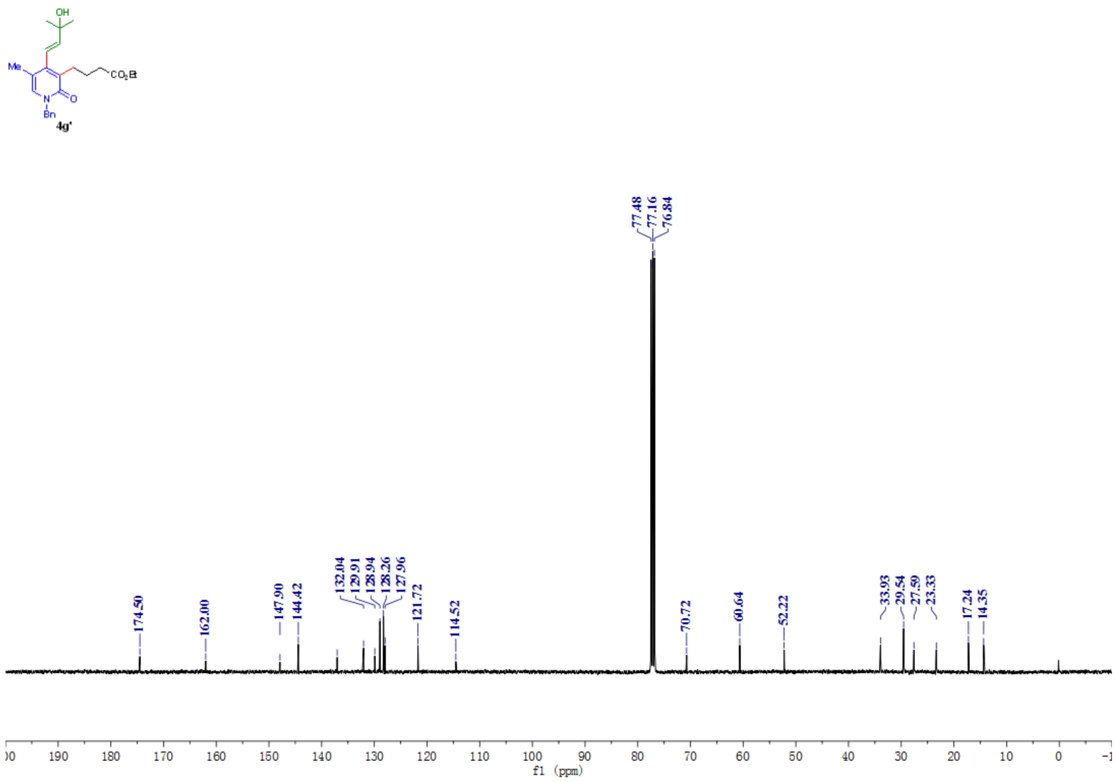

Supplementary Figure 56. NMR of **4g'**

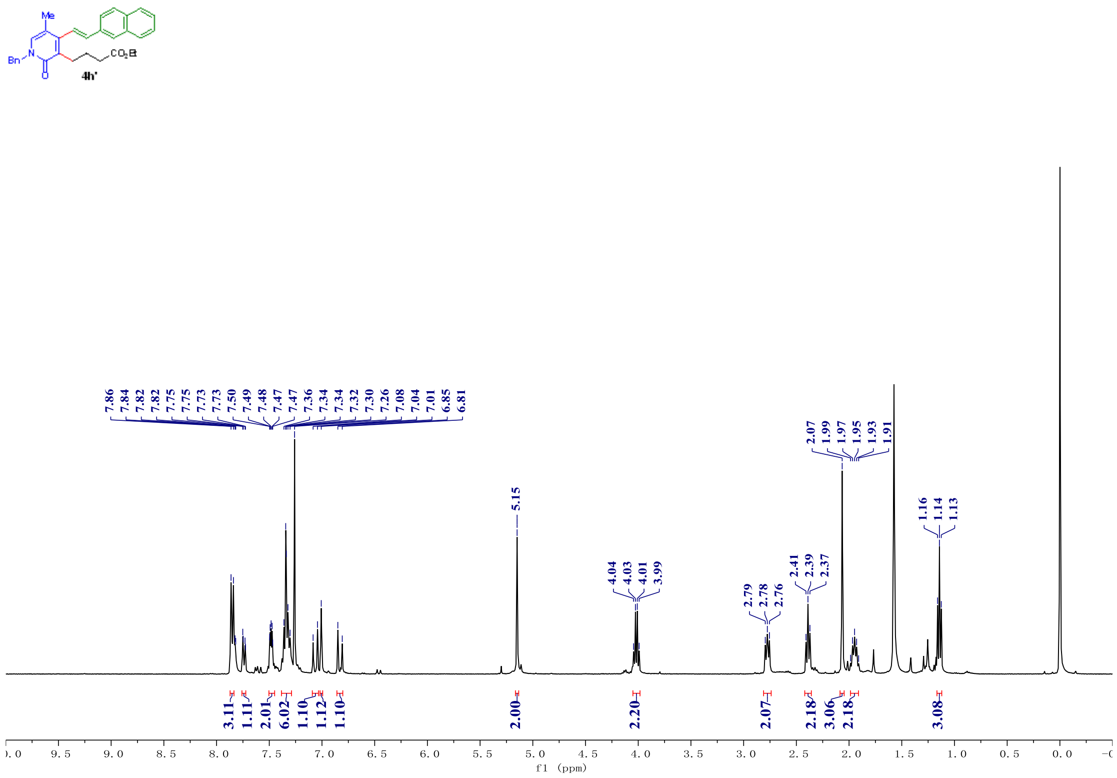

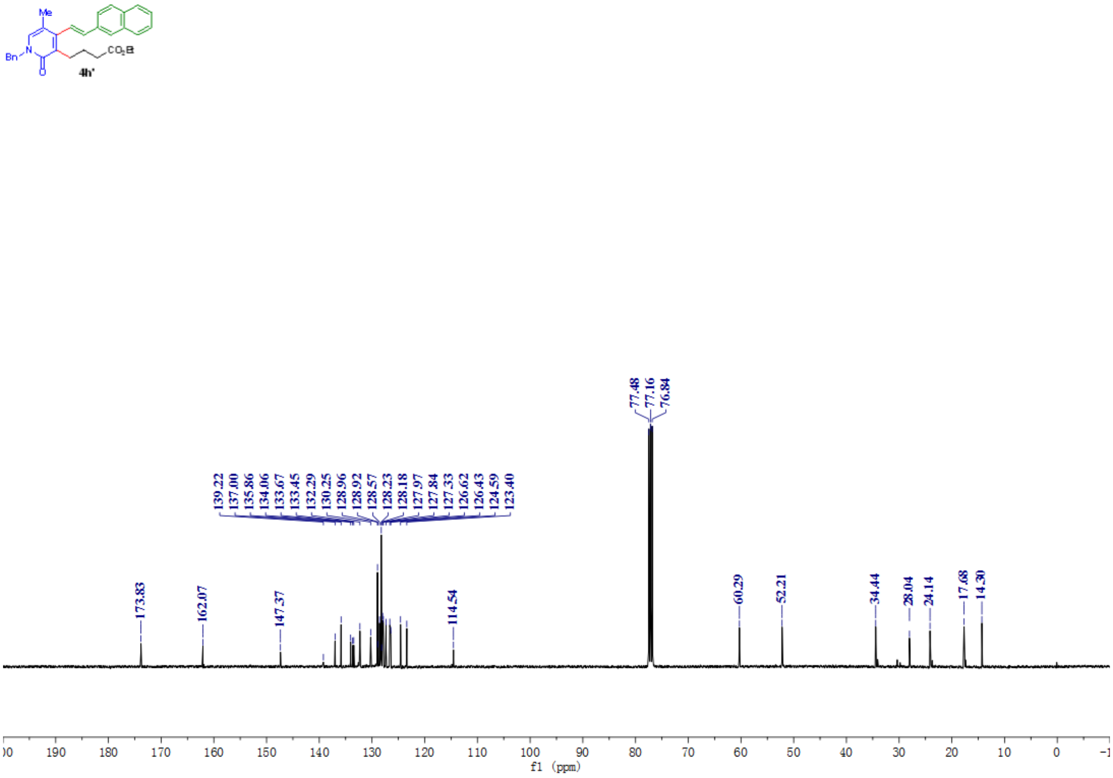

Supplementary Figure 57. NMR of **4h'**

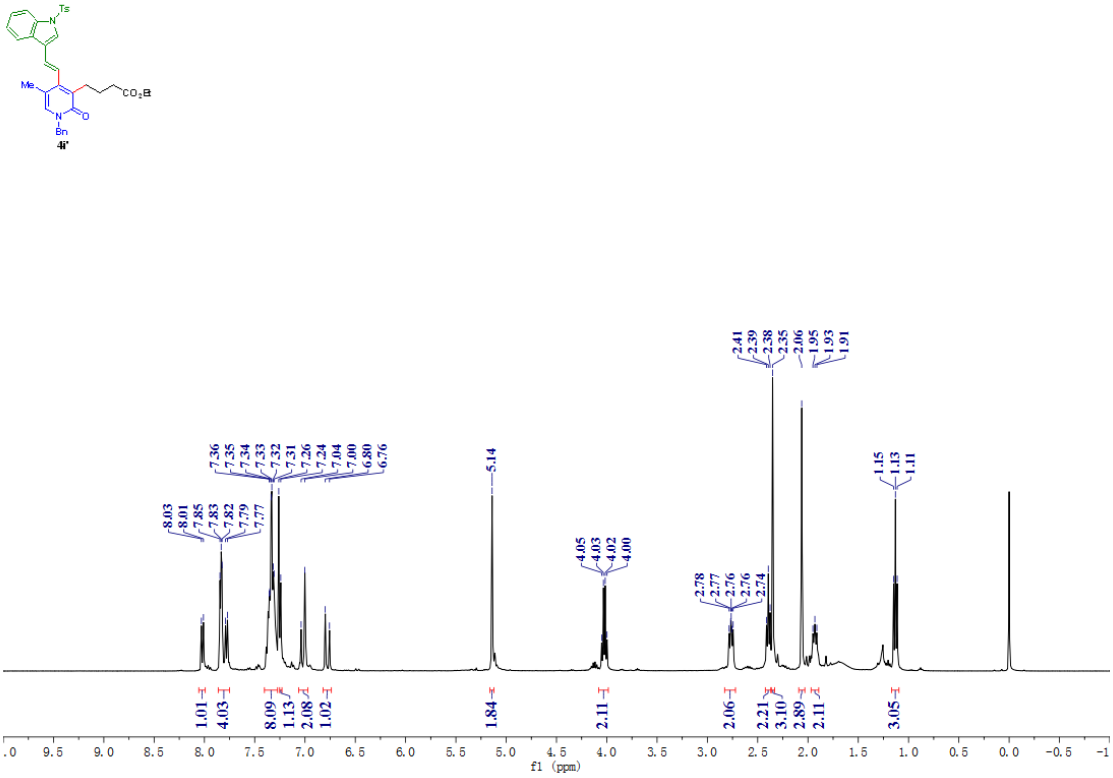

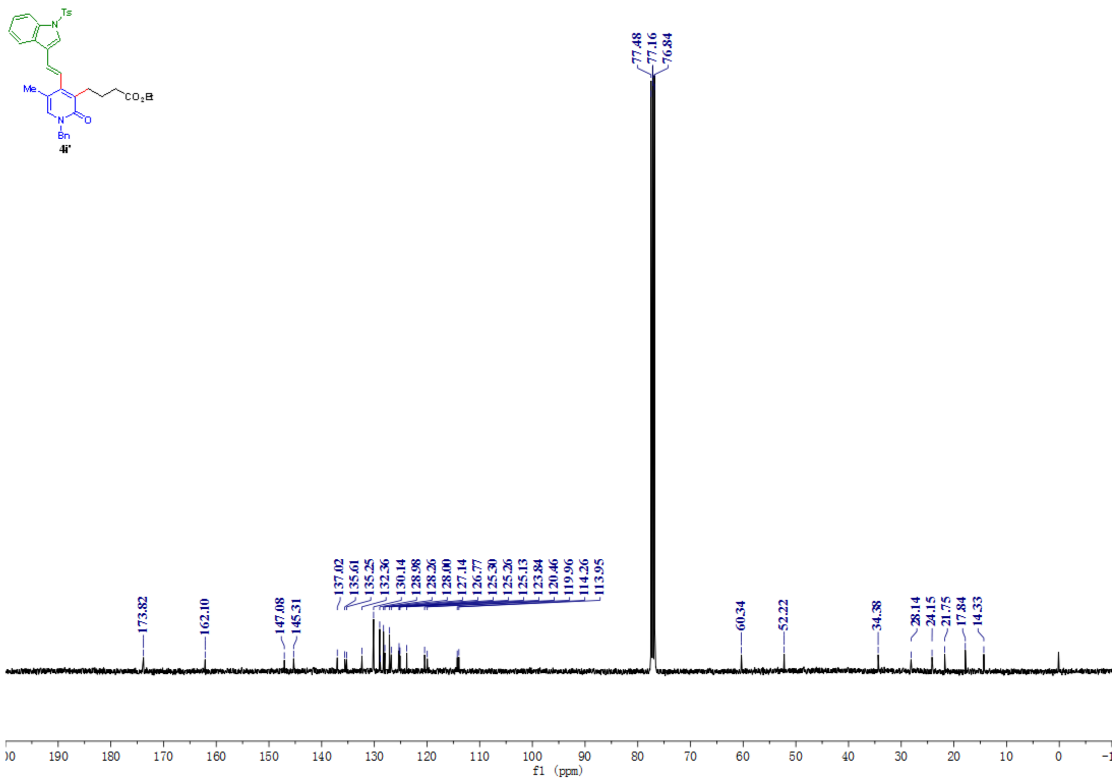

Supplementary Figure 58. NMR of **4i'**

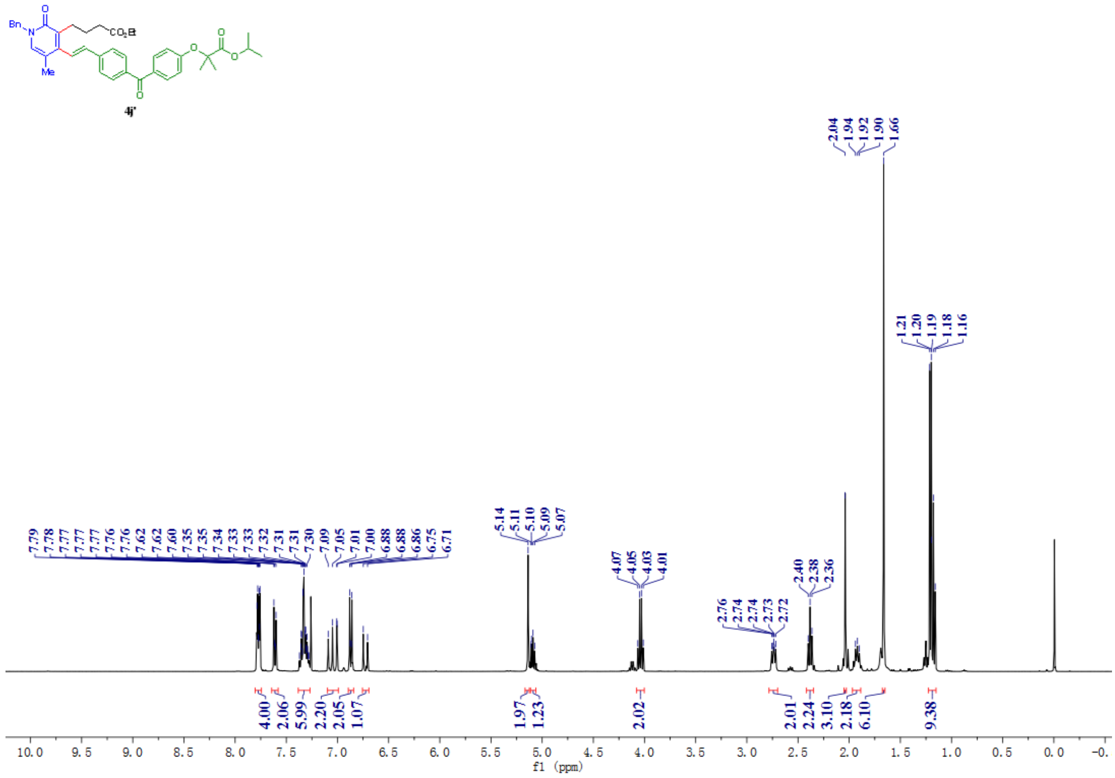

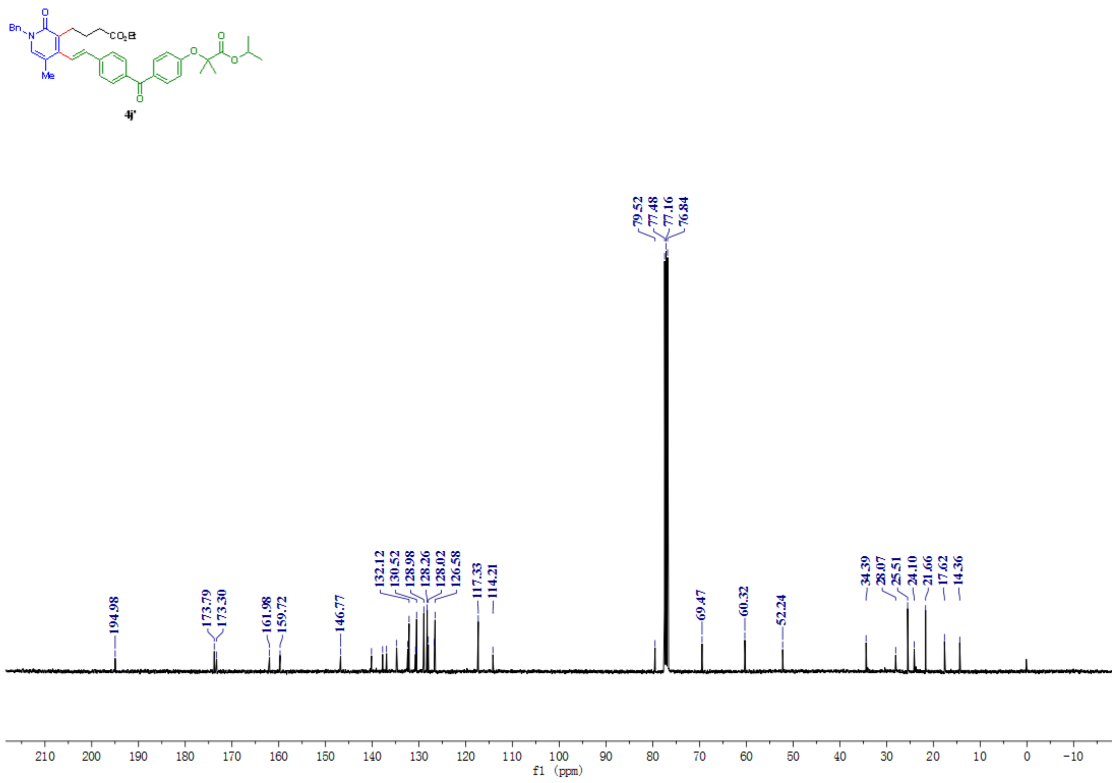

Supplementary Figure 59. NMR of **4j'**

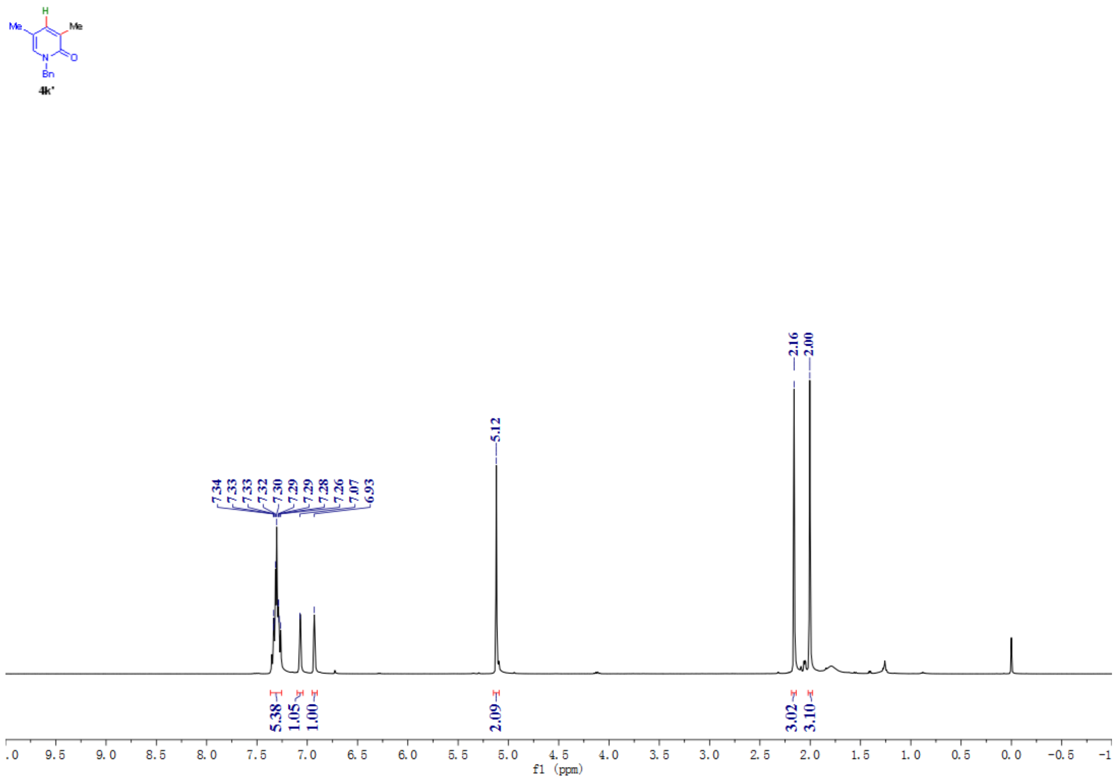

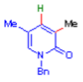

4k'

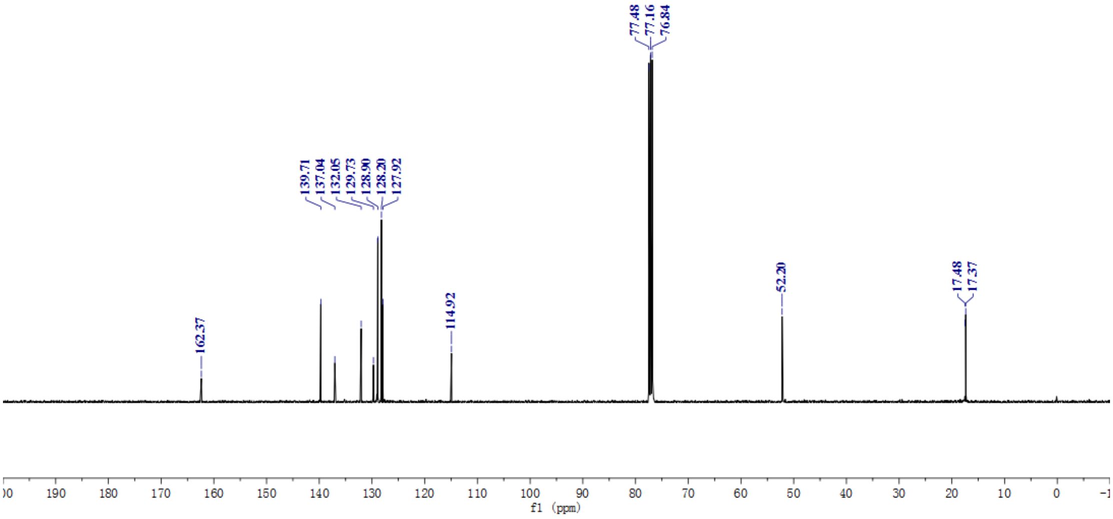

Supplementary Figure 60. NMR of **4k'**

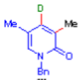

4l'

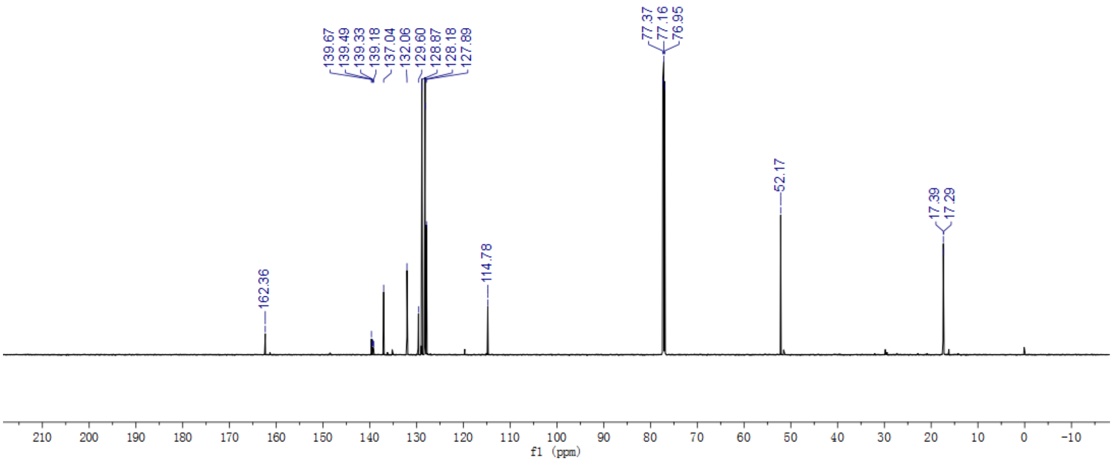

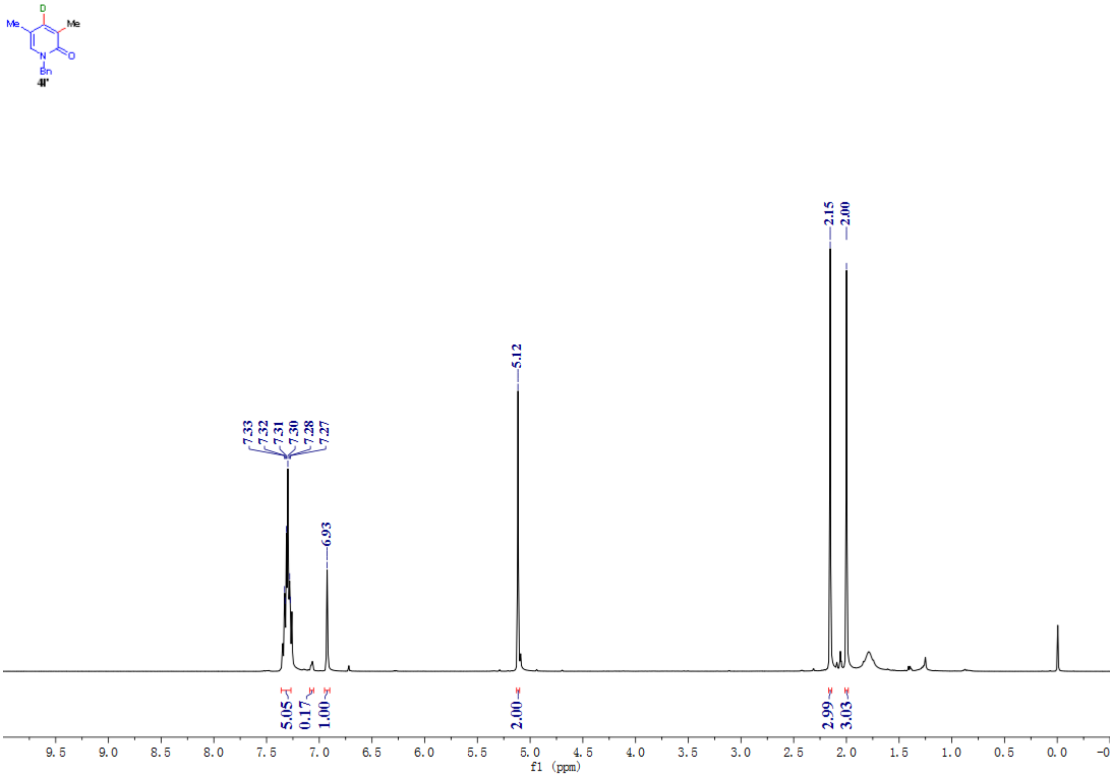

Supplementary Figure 61. NMR of **4l'**

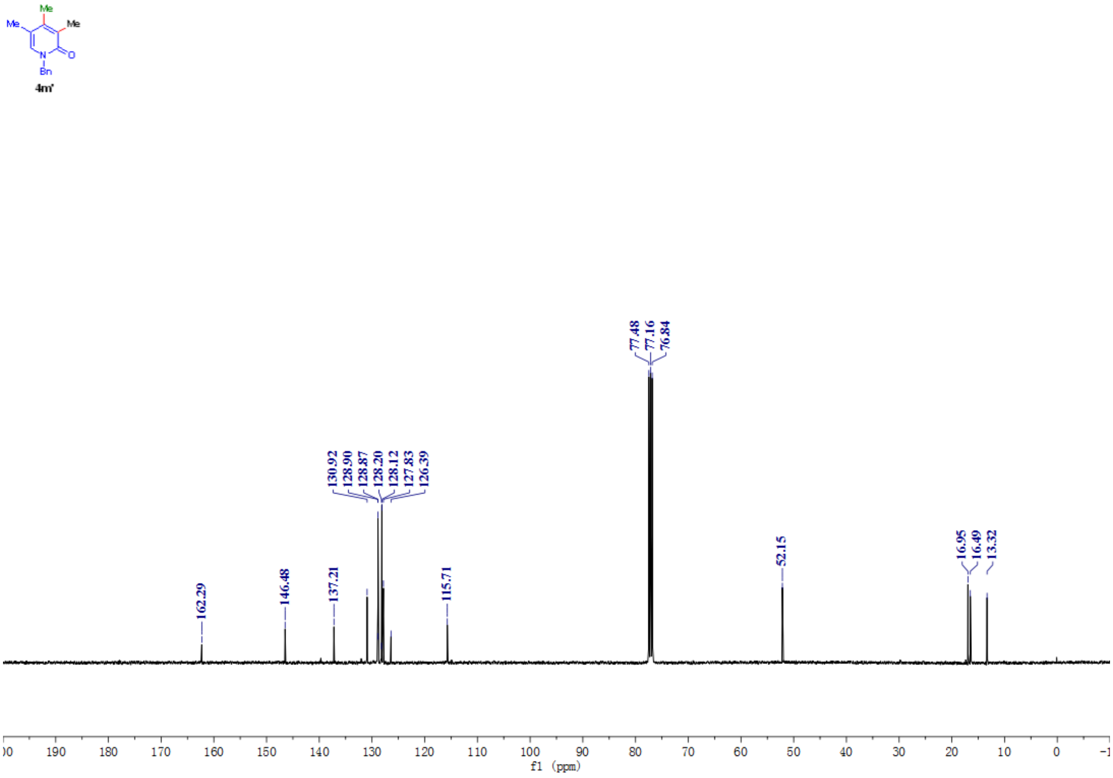

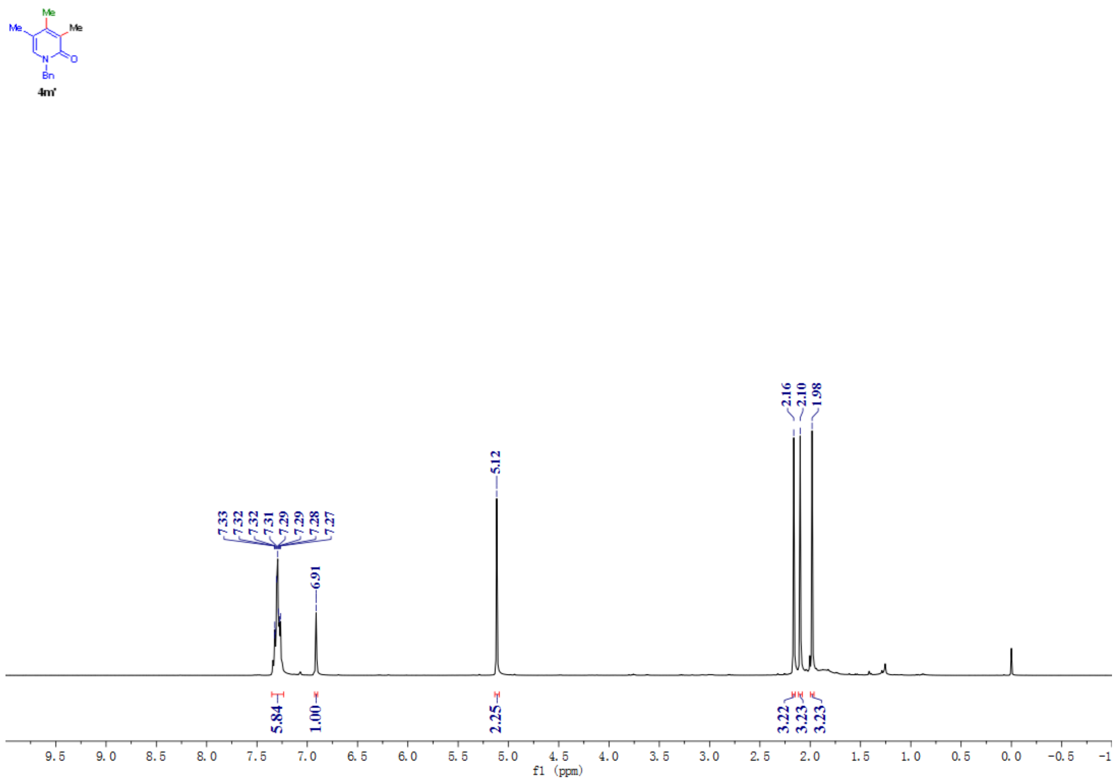

Supplementary Figure 62. NMR of **4m'**

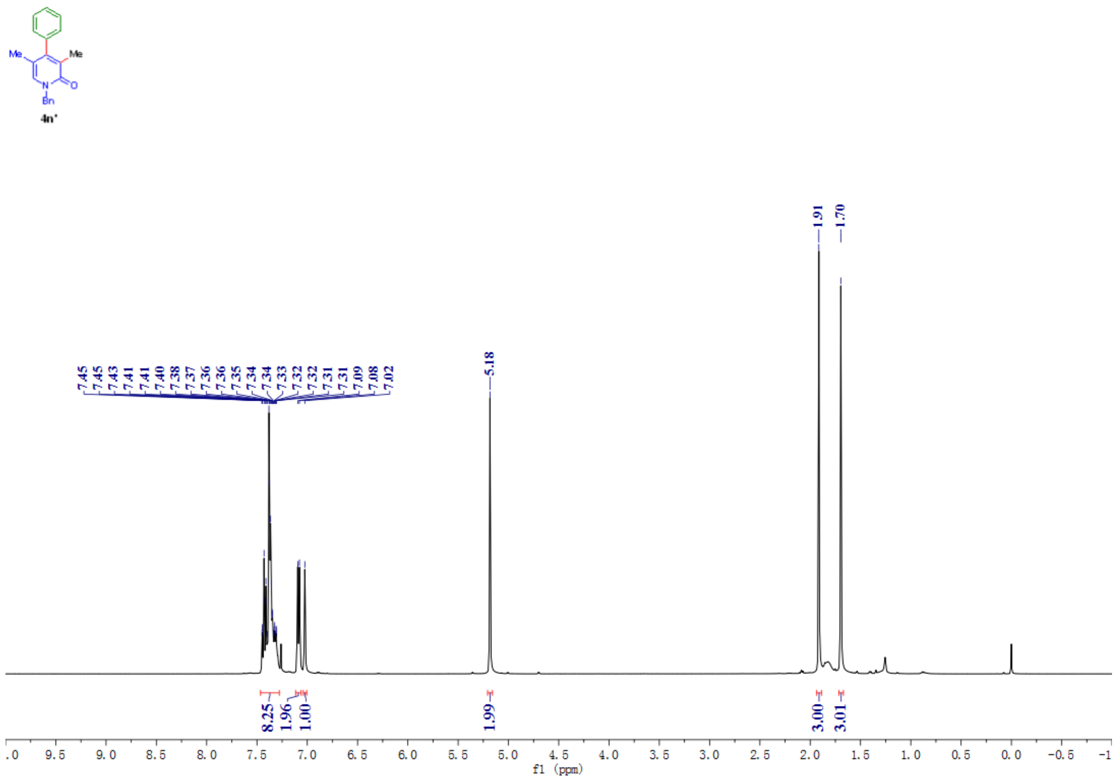

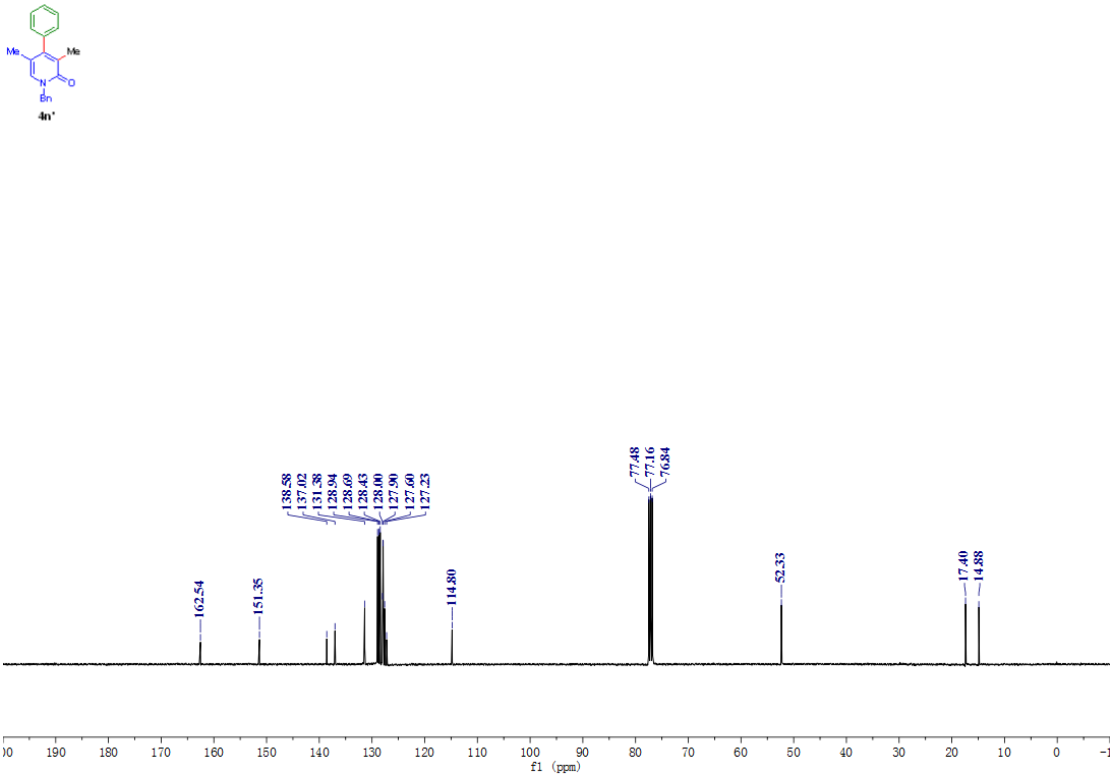

Supplementary Figure 63. NMR of **4n'**

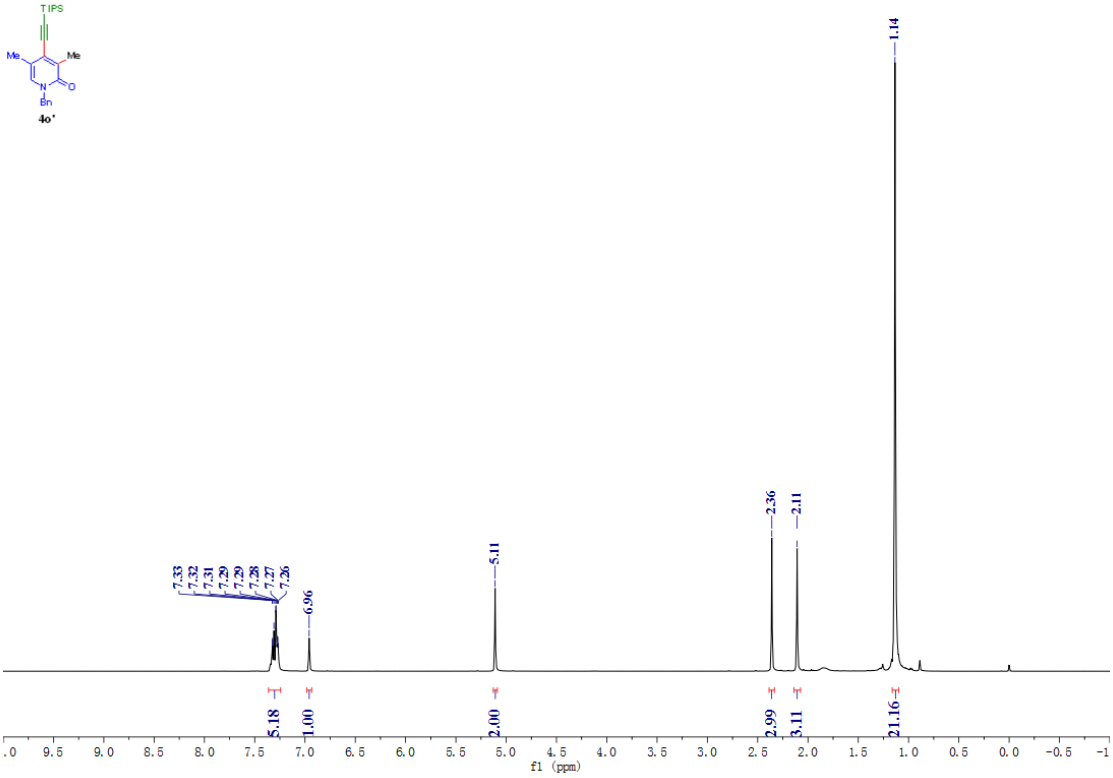

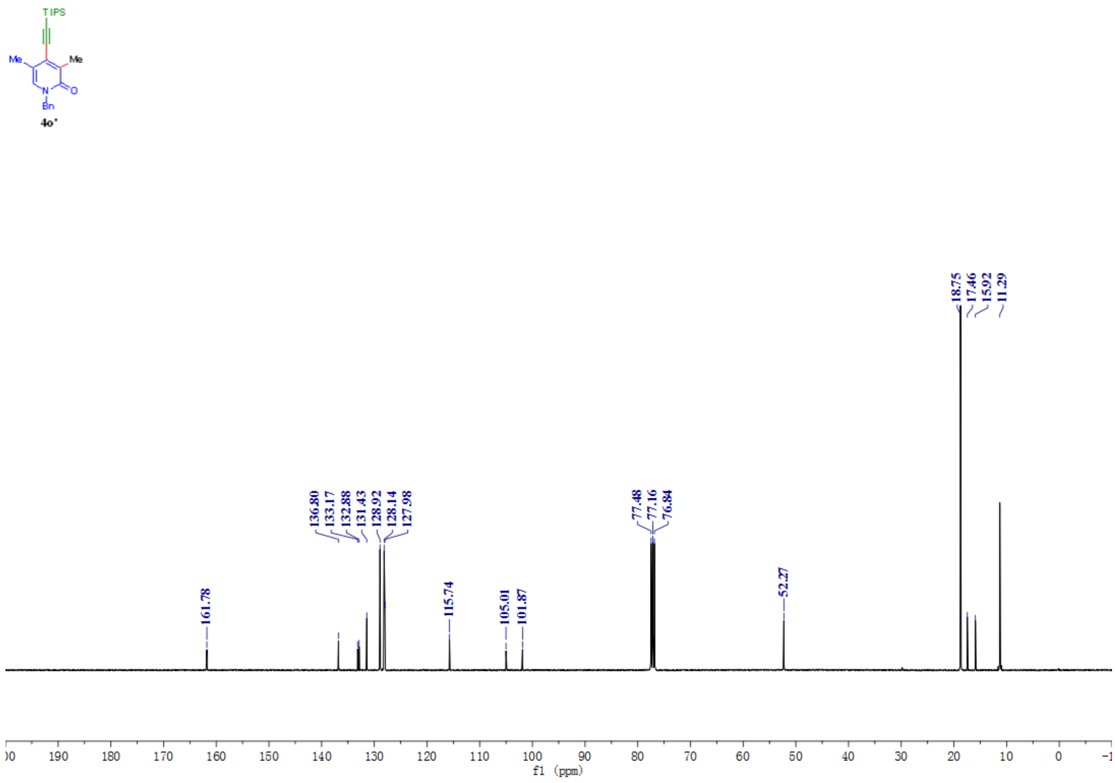

Supplementary Figure 64. NMR of **4o'**

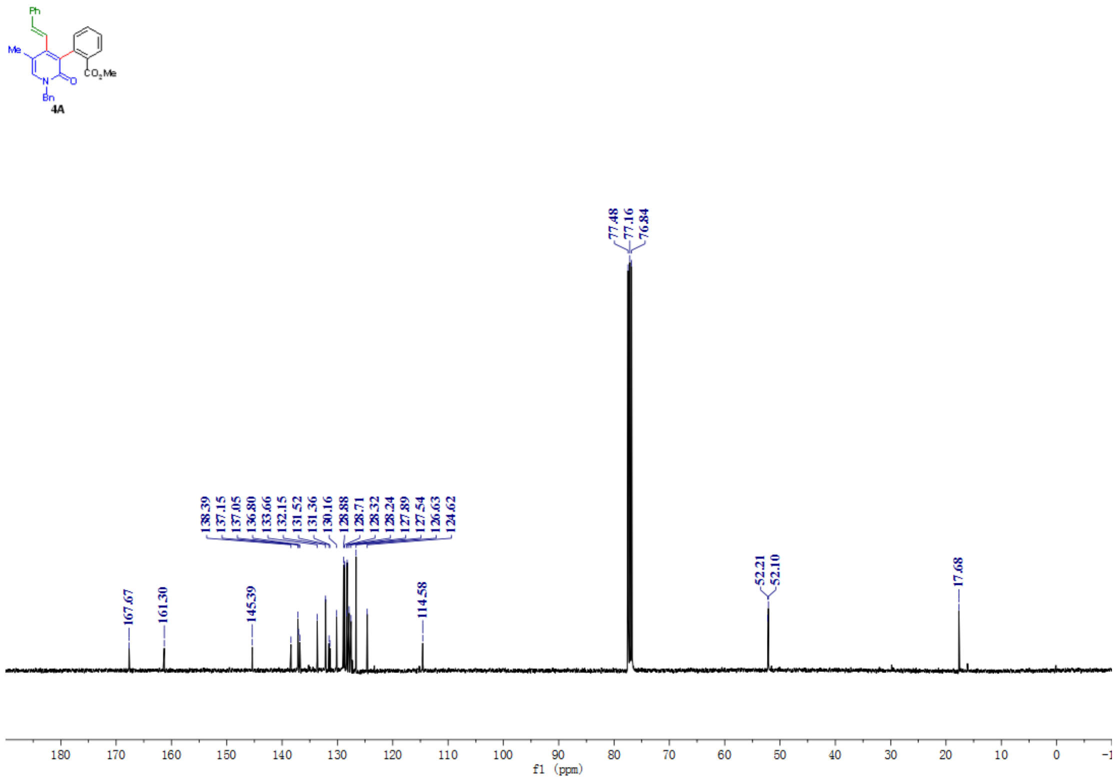

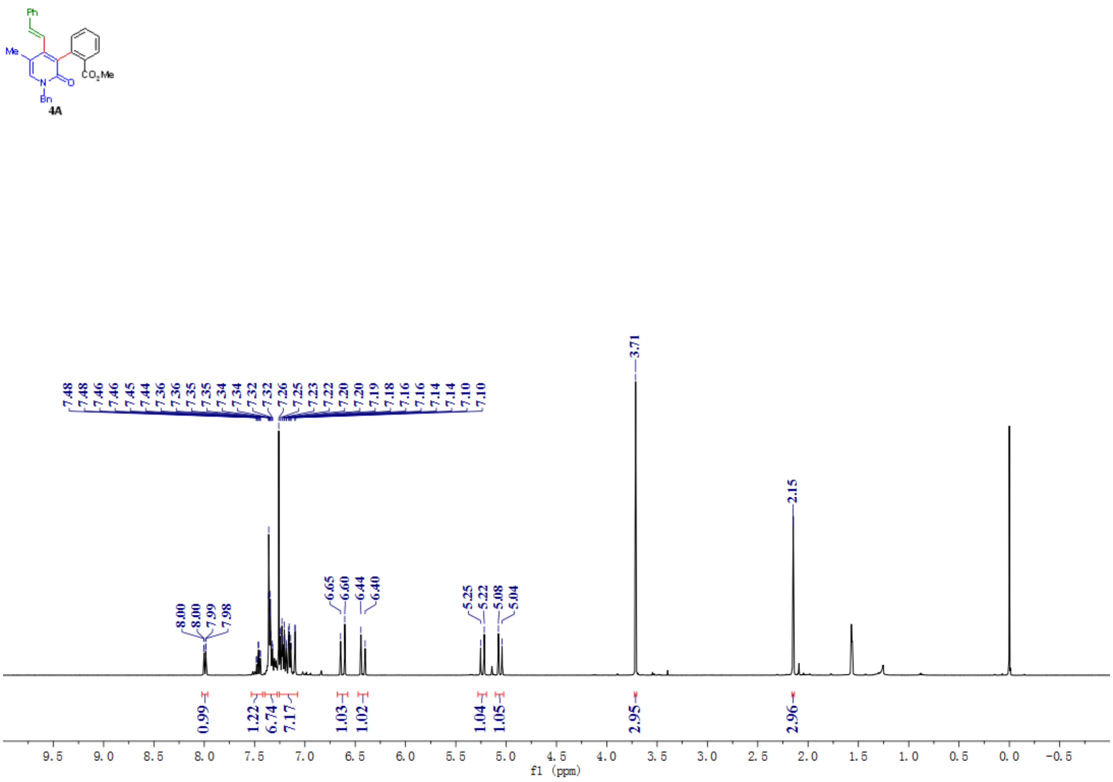

Supplementary Figure 65. NMR of **4A**

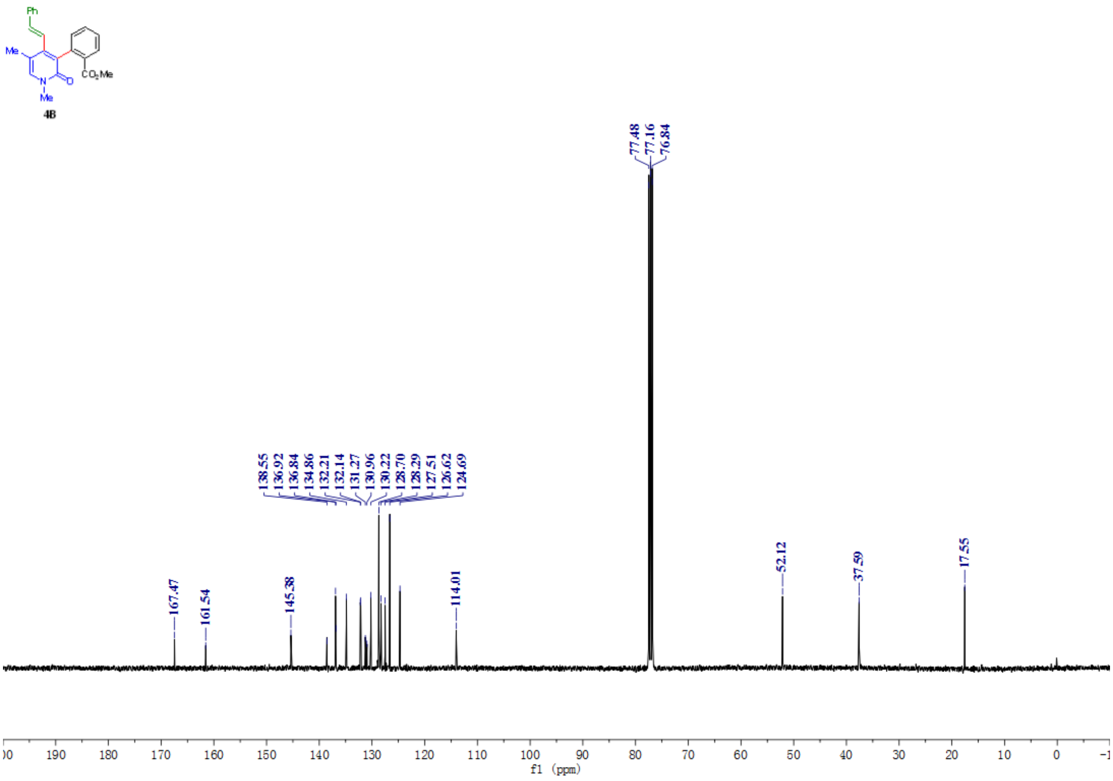

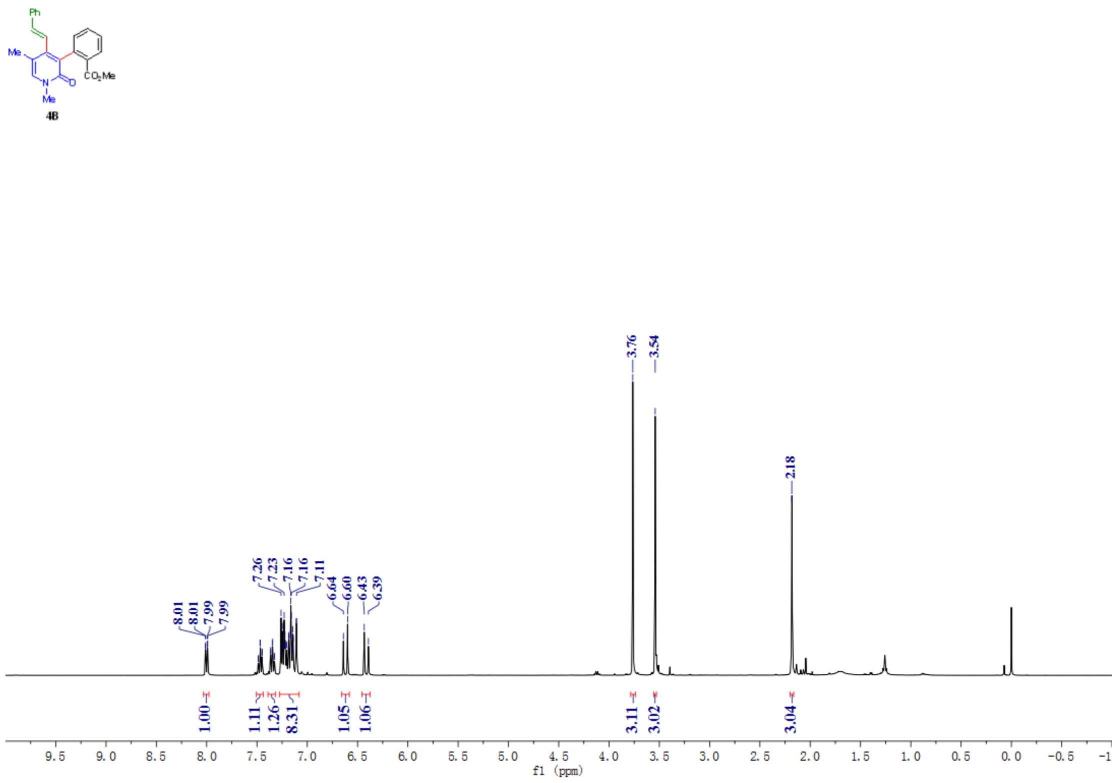

Supplementary Figure 66. NMR of **4B**

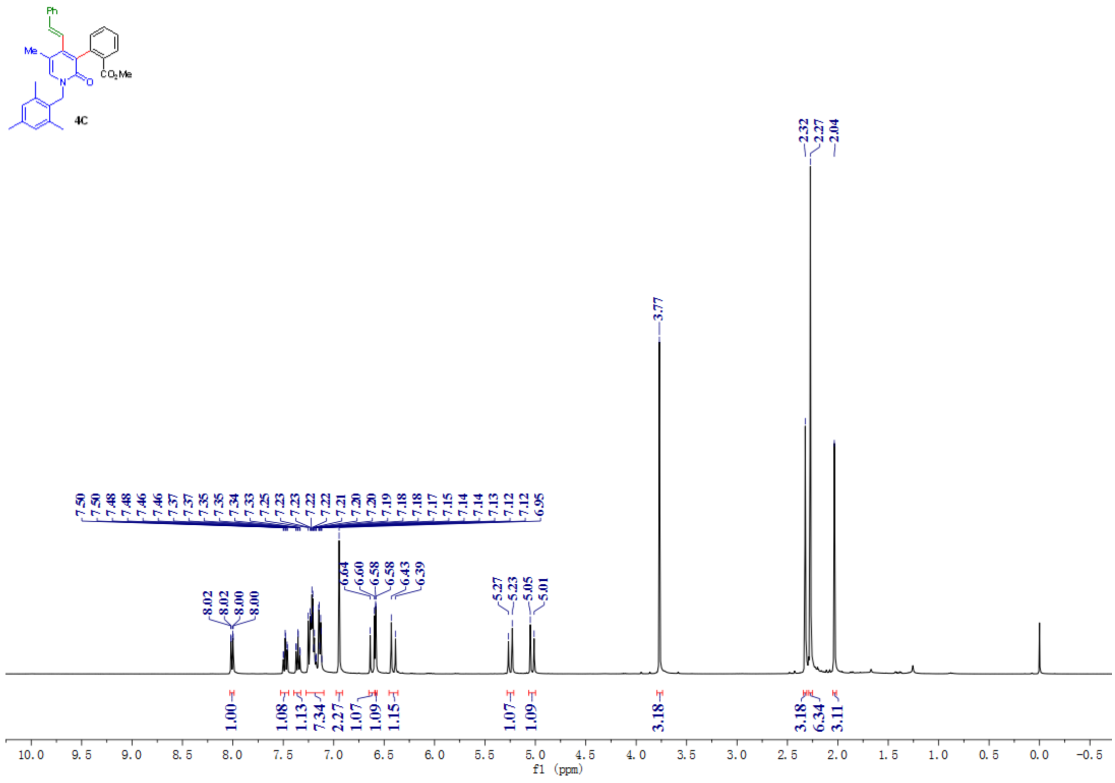

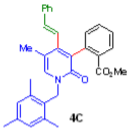

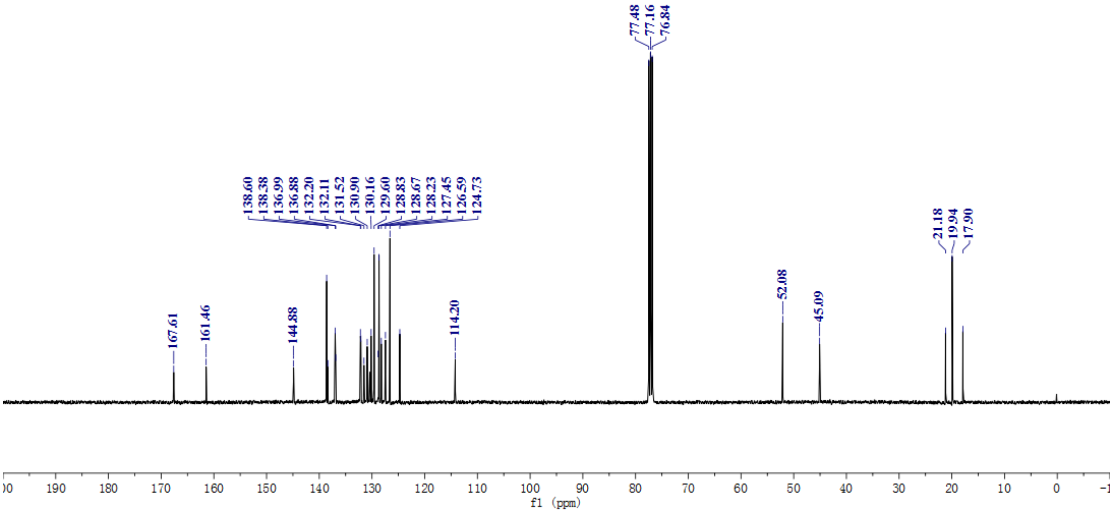

Supplementary Figure 67. NMR of **4C**

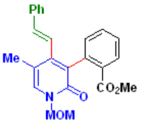

4D

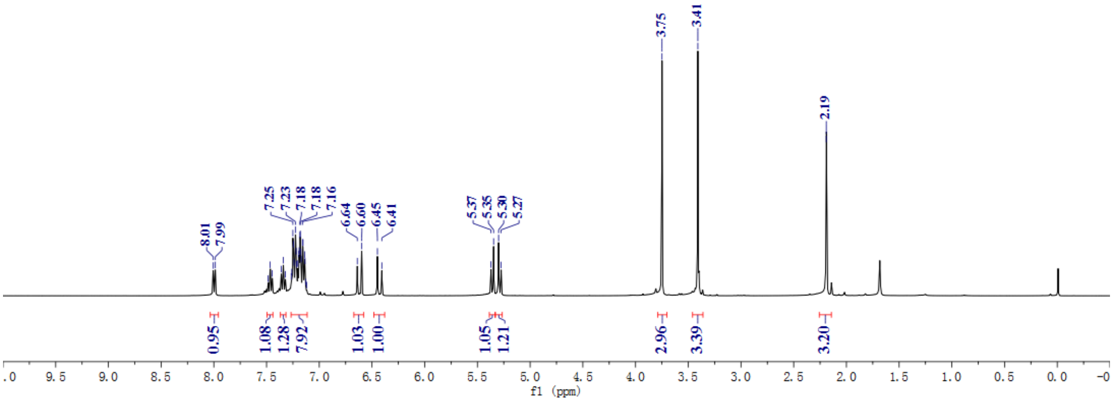

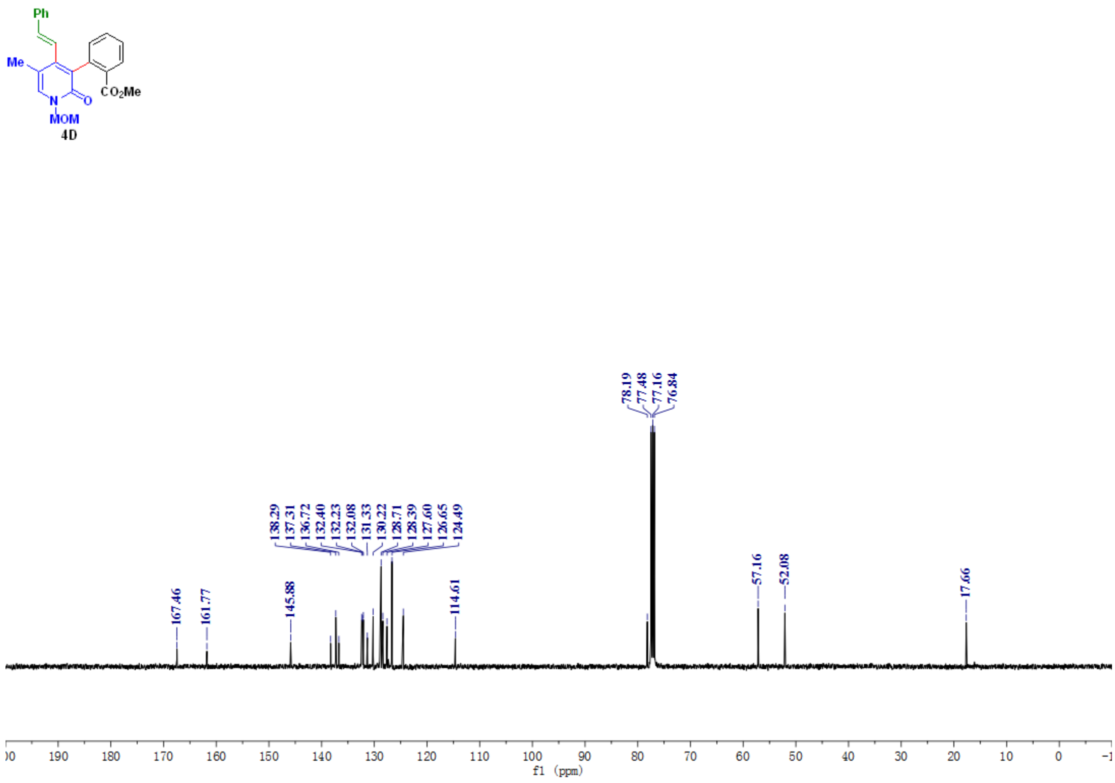

Supplementary Figure 68. NMR of **4D**

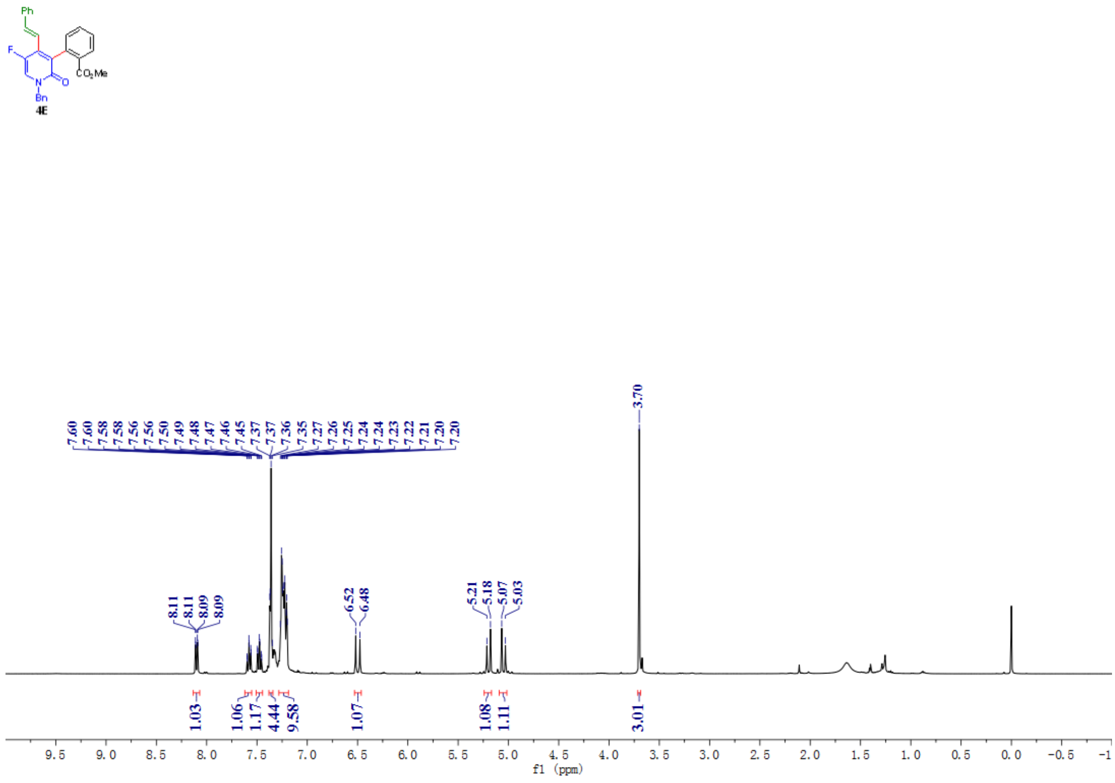

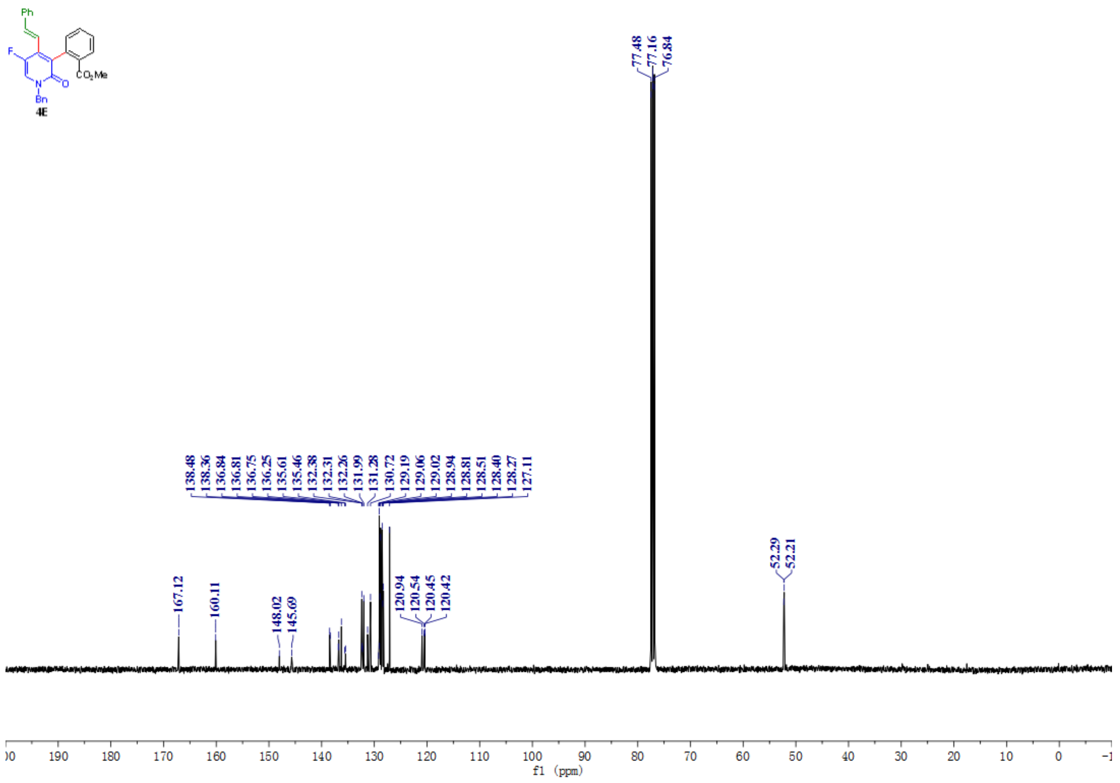

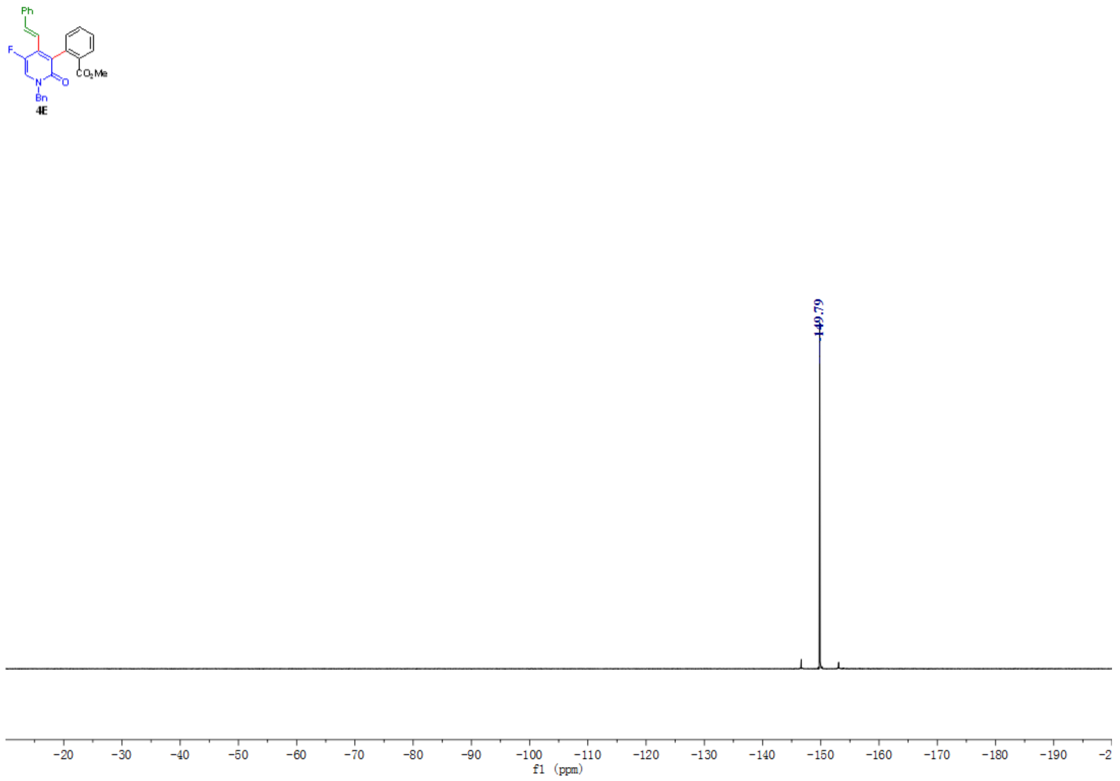

Supplementary Figure 69. NMR of **4E**

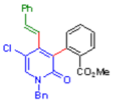

4F

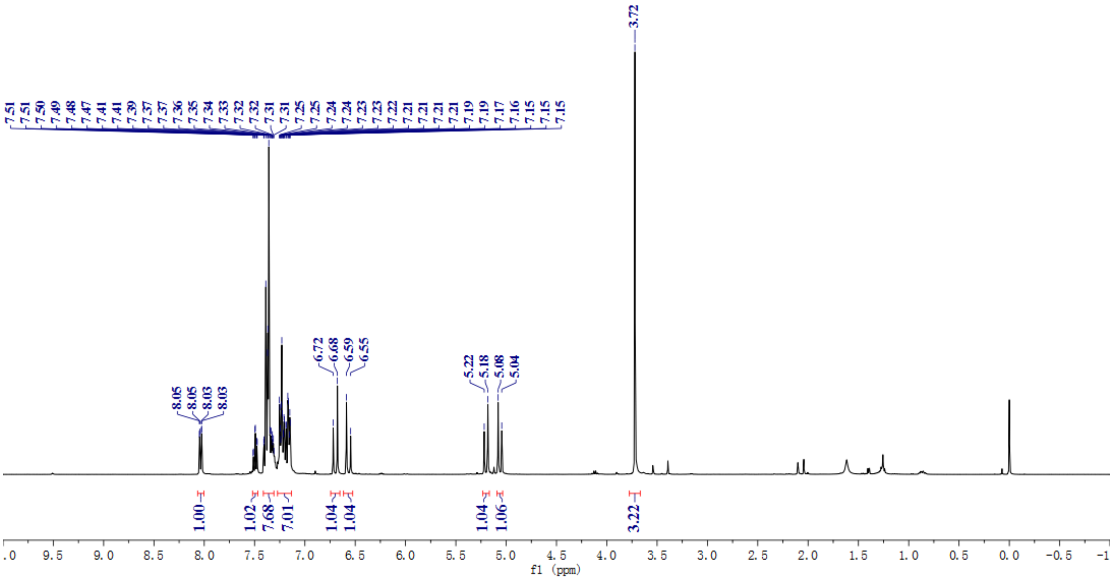

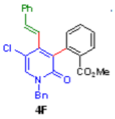

4F

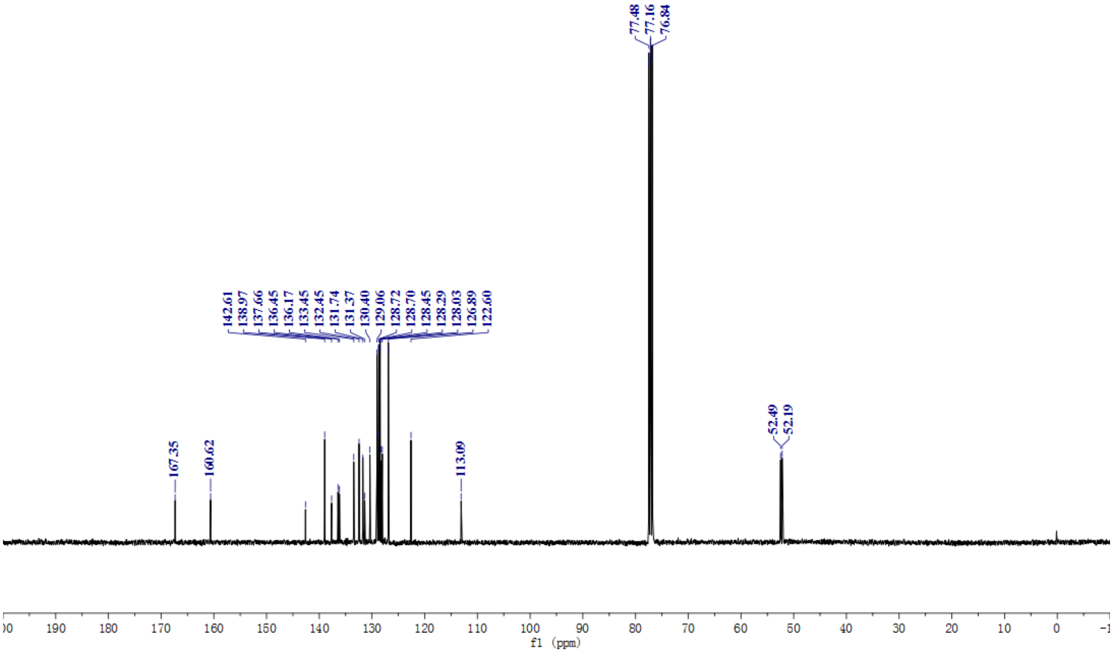

Supplementary Figure 70. NMR of **4F**

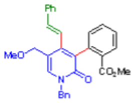

4G

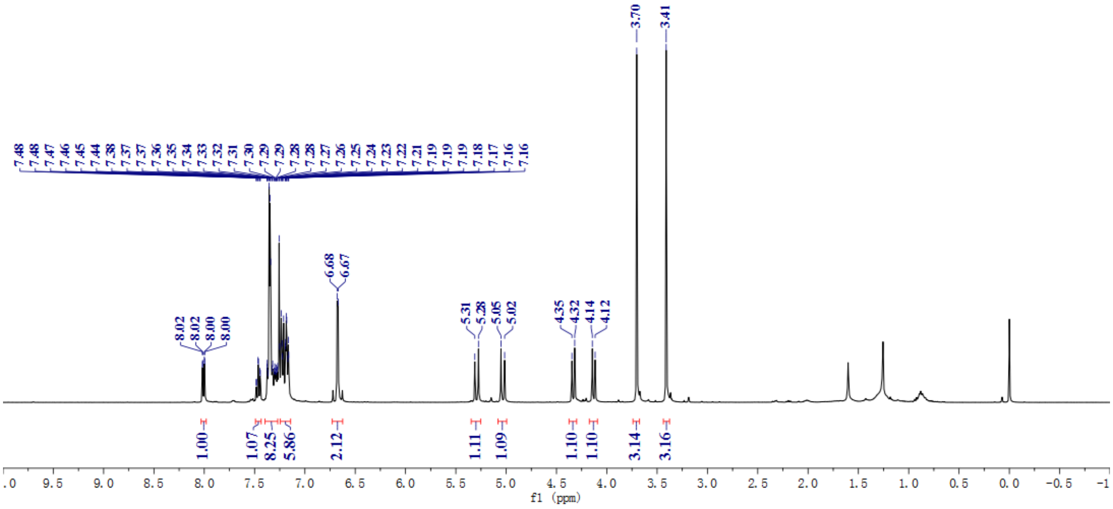

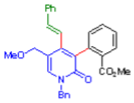

4G

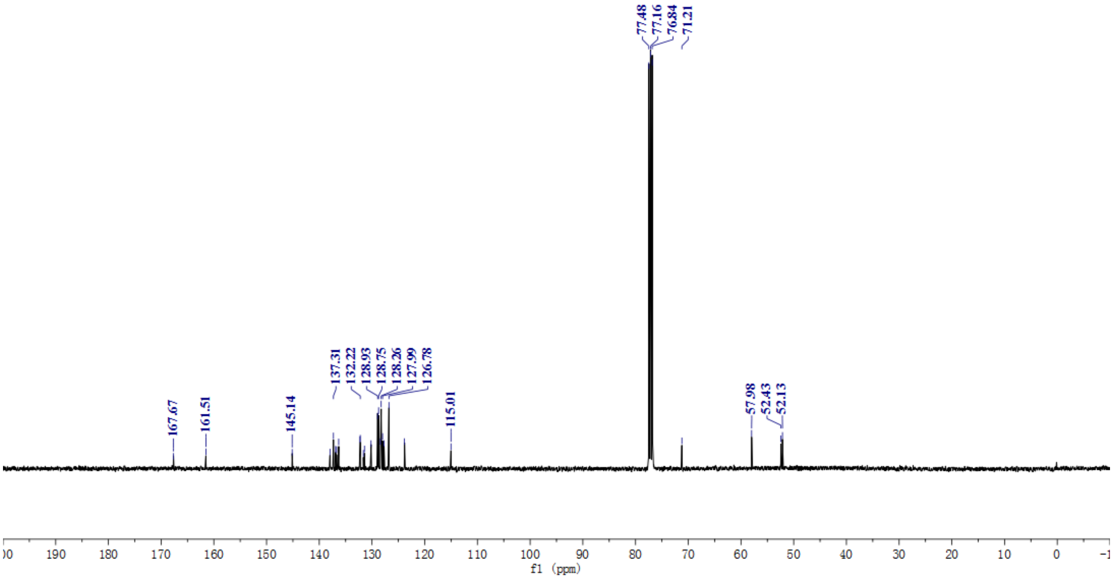

Supplementary Figure 71. NMR of **4G**

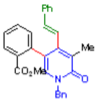

4H

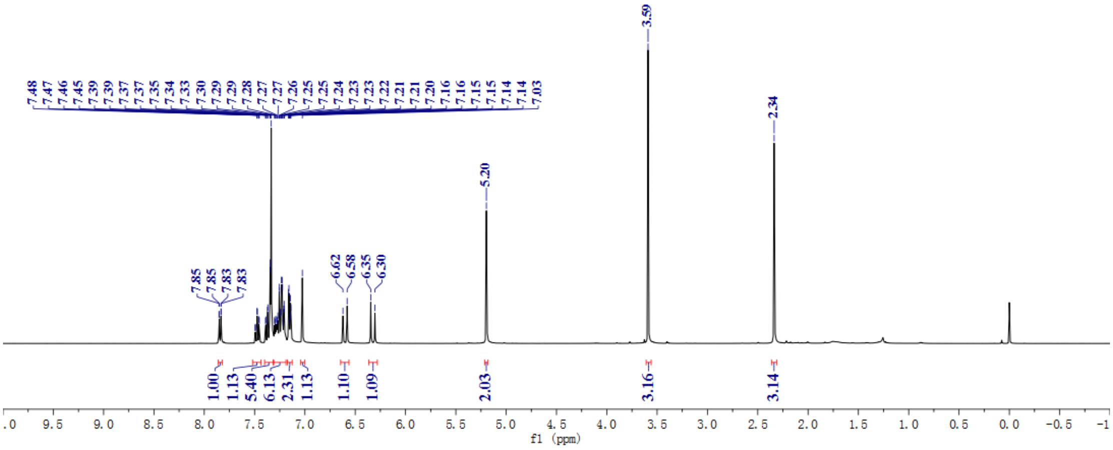

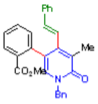

4H

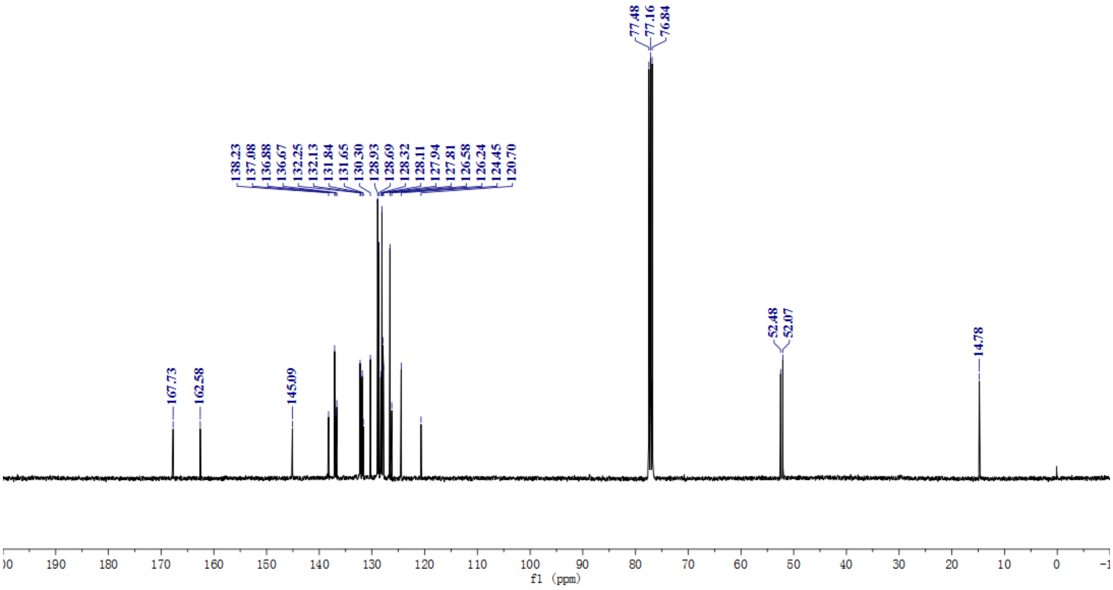

Supplementary Figure 72. NMR of **4H**

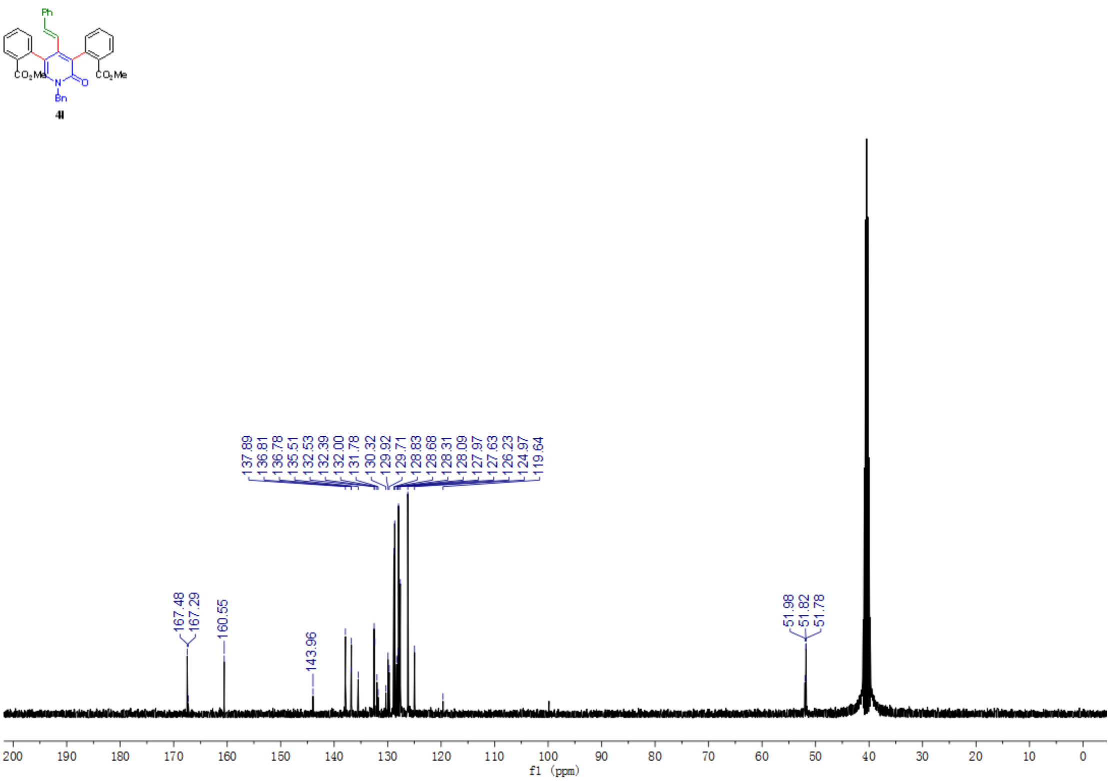

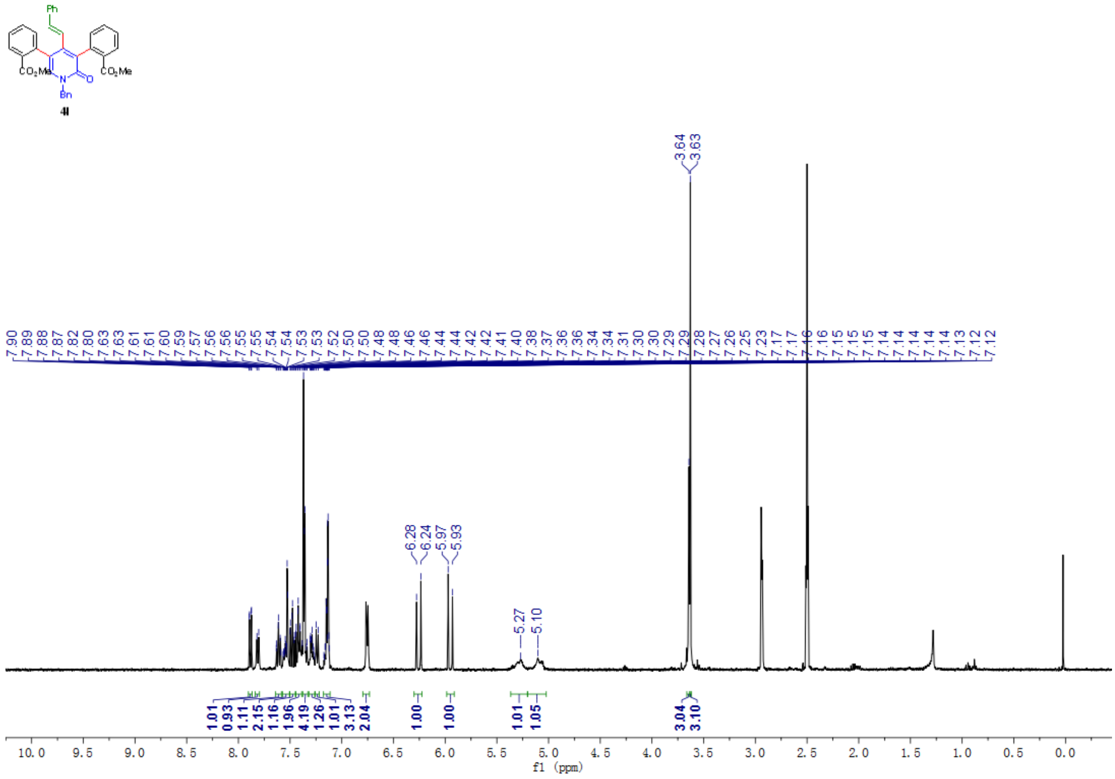

Supplementary Figure 73. NMR of **4l**

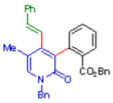

4J

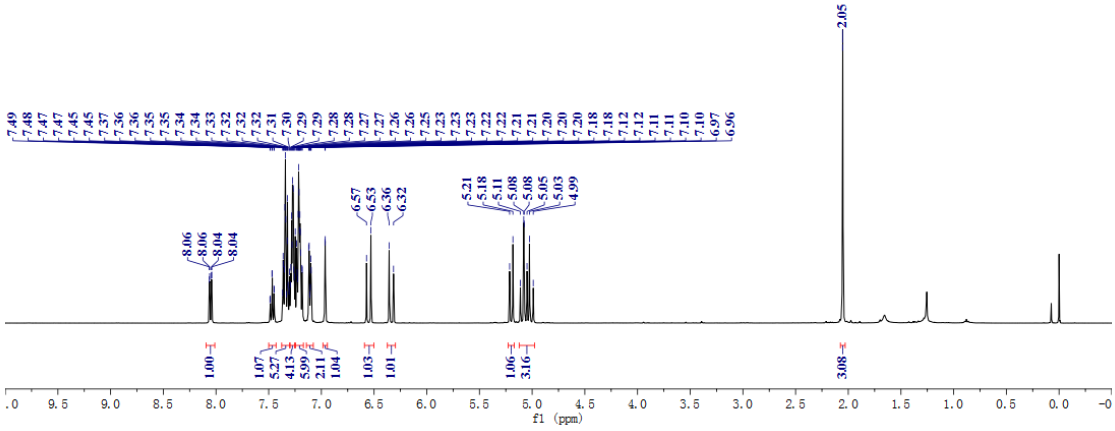

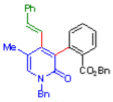

4J

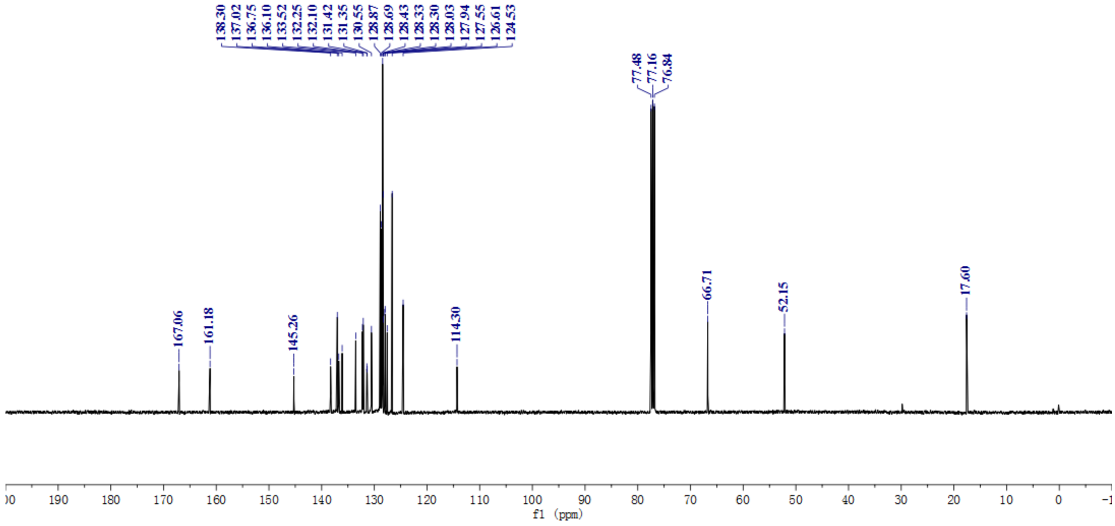

Supplementary Figure 74. NMR of **4J**

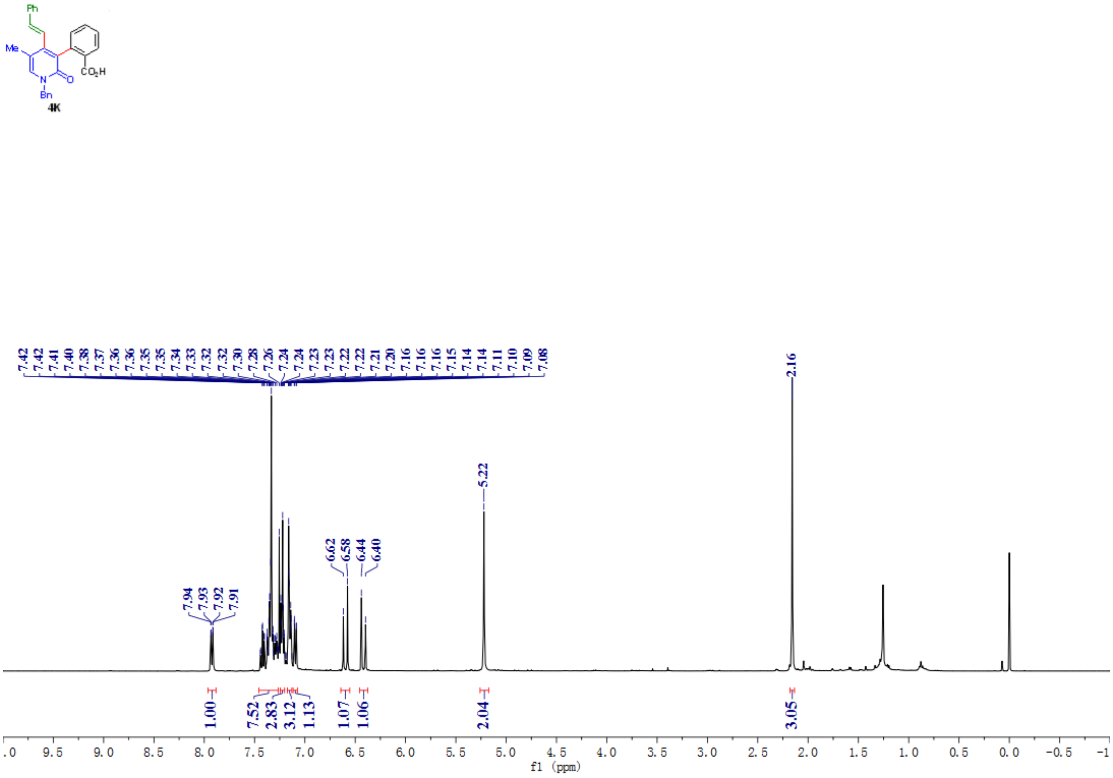

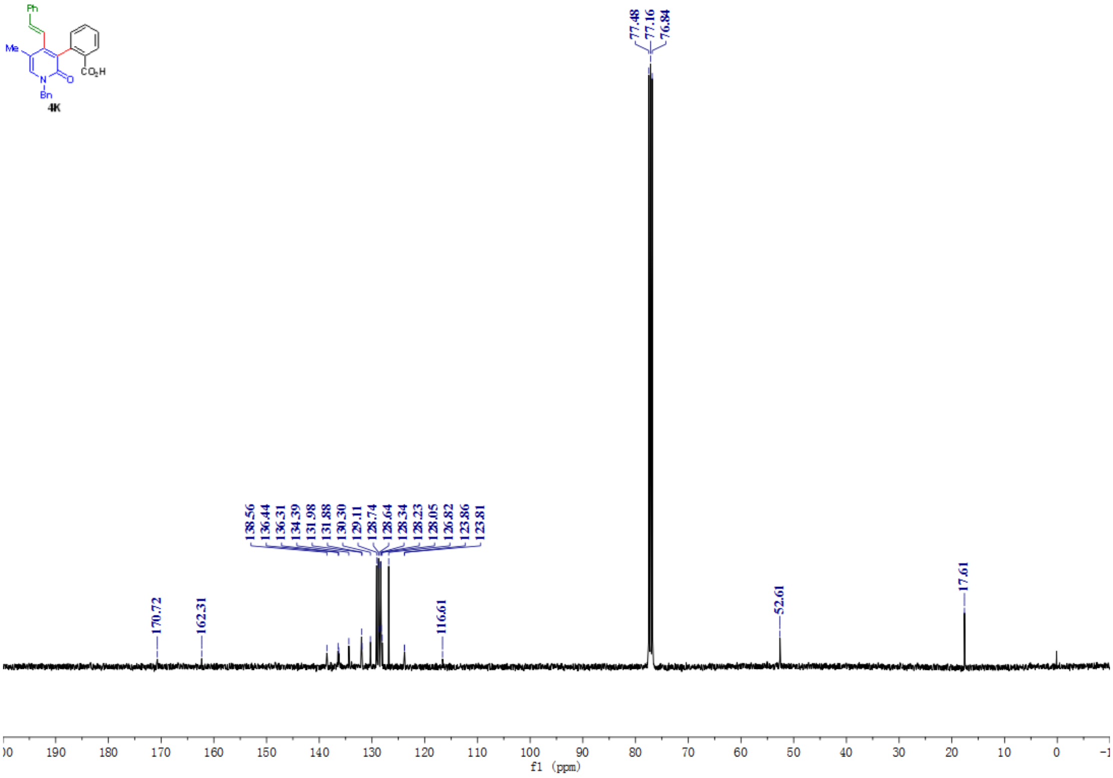

Supplementary Figure 75. NMR of **4K**

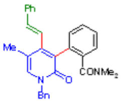

4L

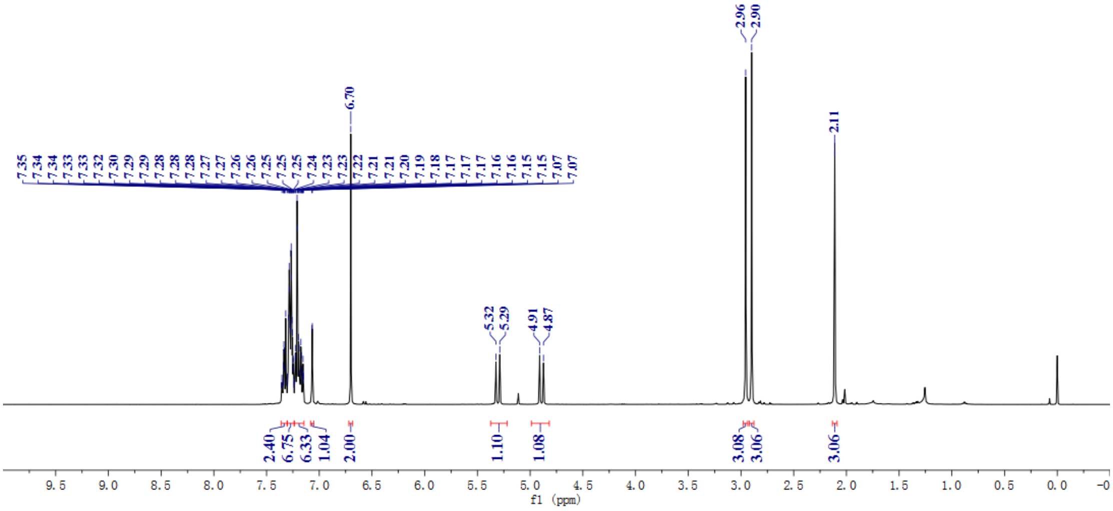

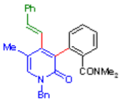

4L

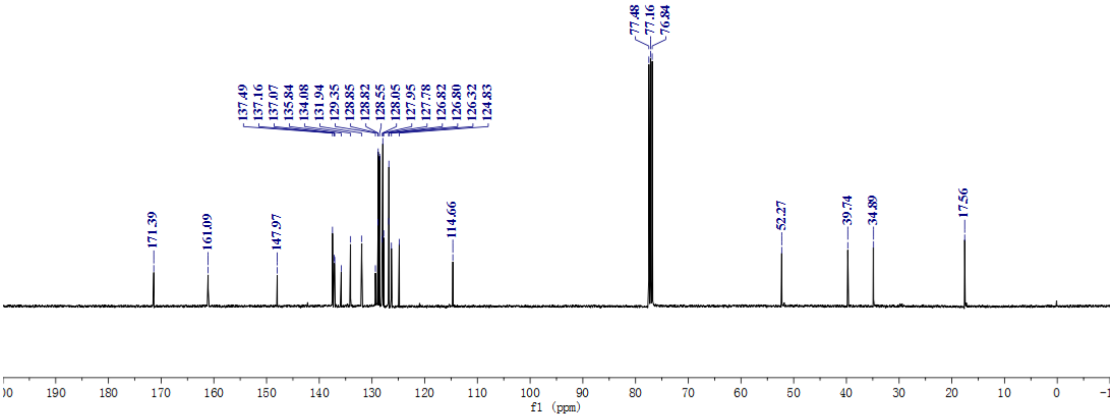

Supplementary Figure 76. NMR of **4L**

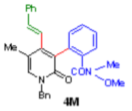

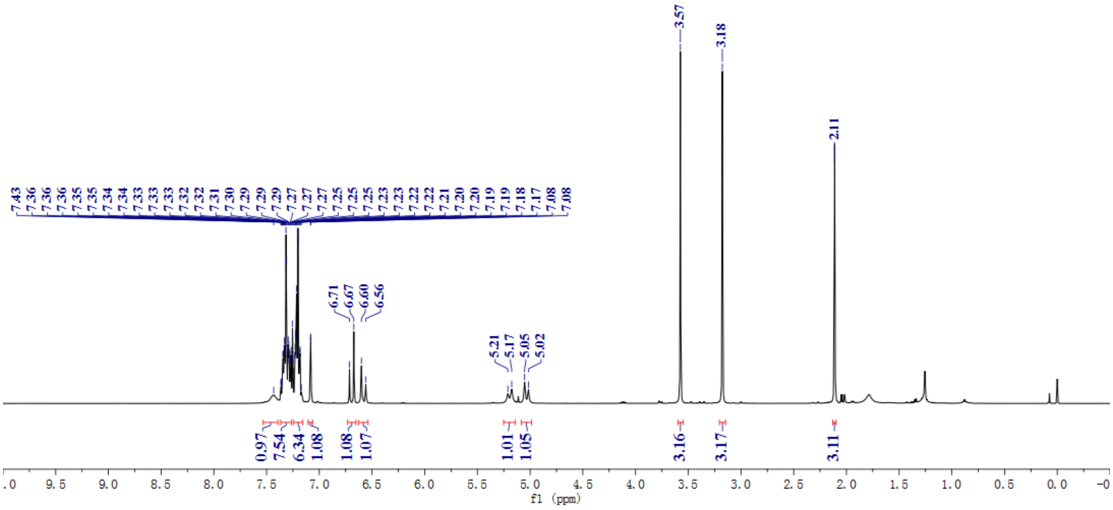

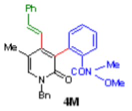

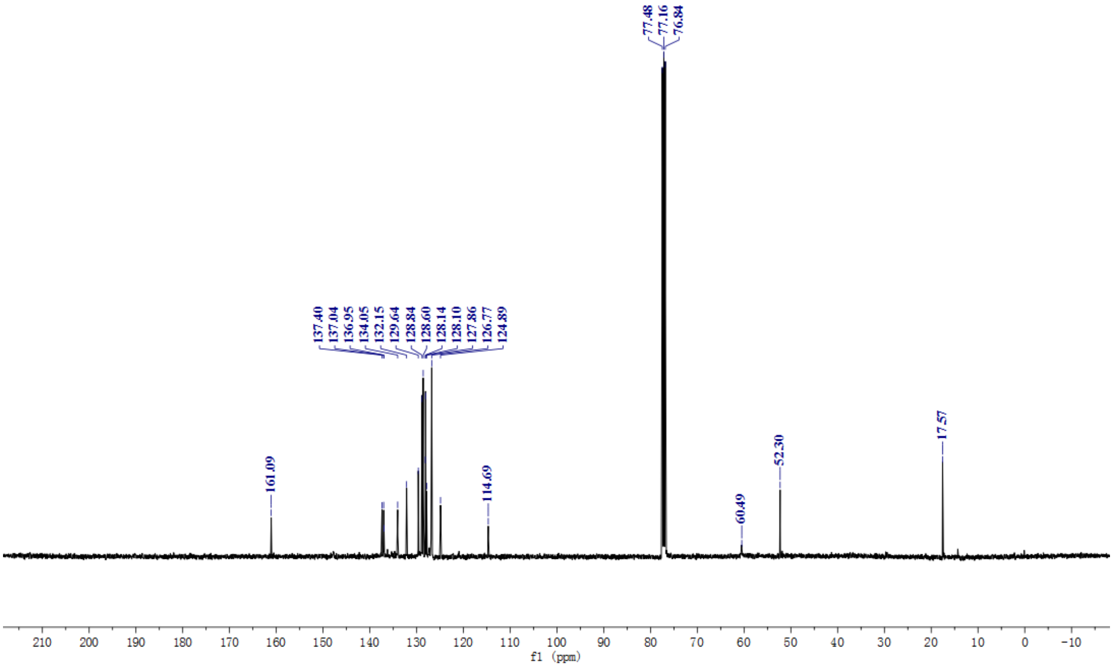

Supplementary Figure 77. NMR of **4M**

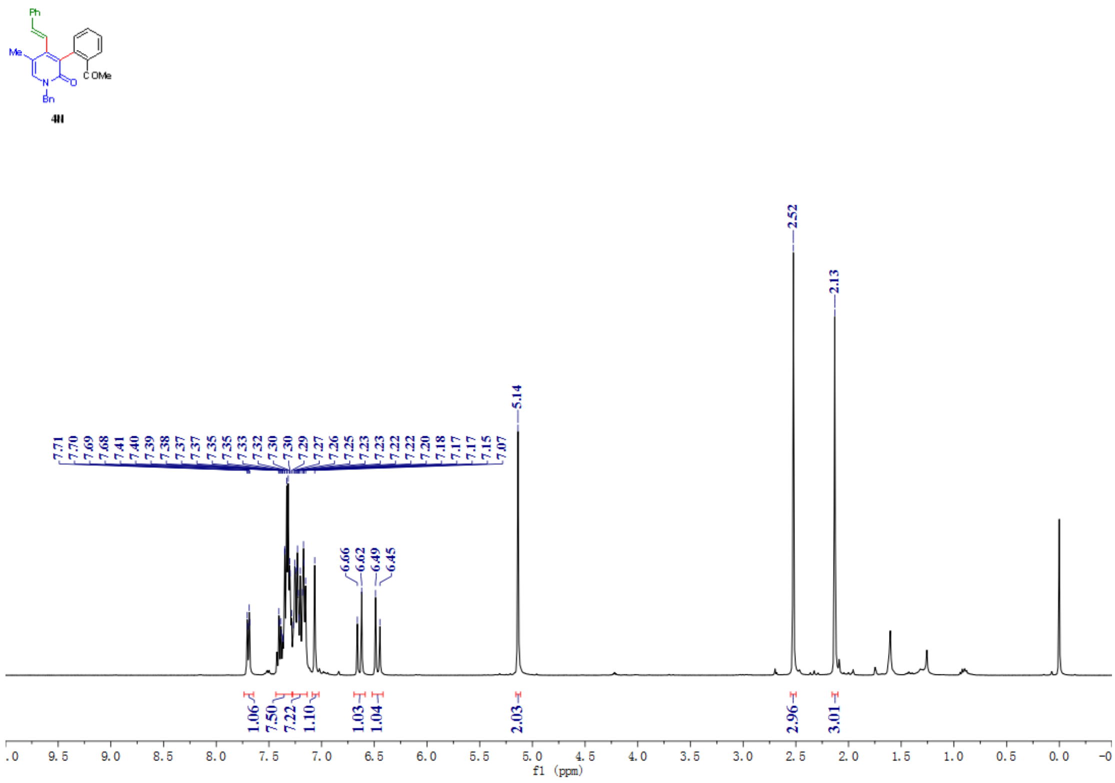

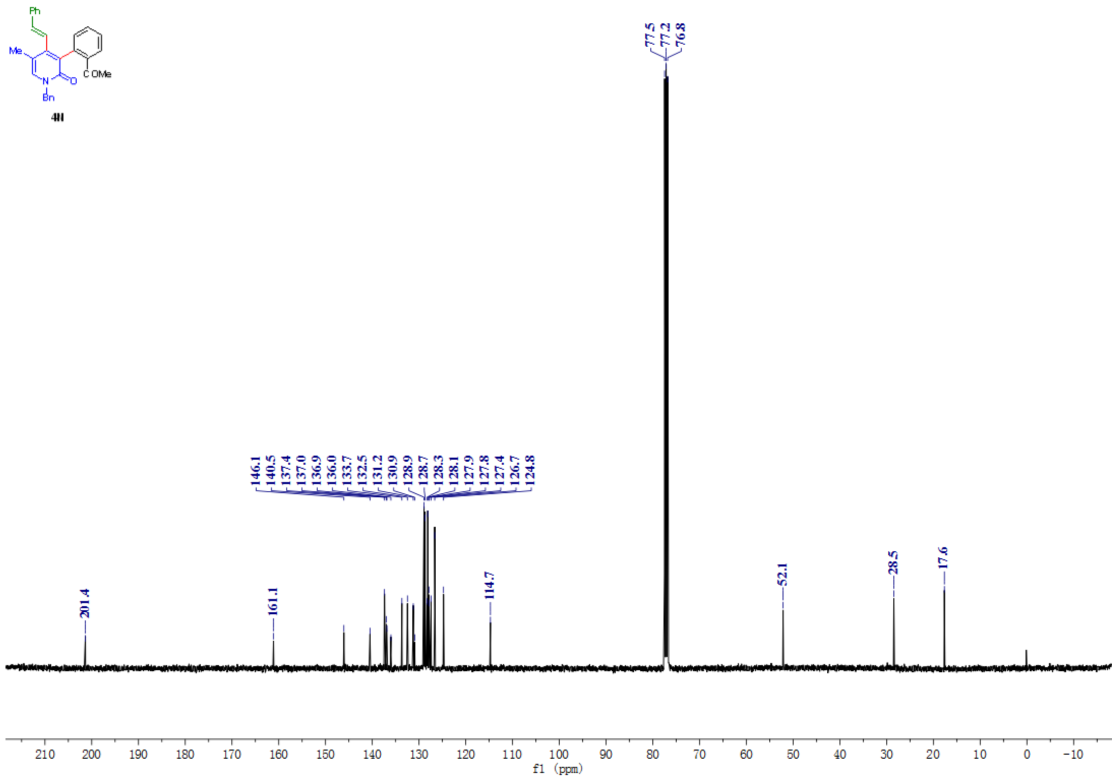

Supplementary Figure 78. NMR of **4N**

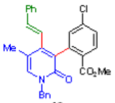

4O

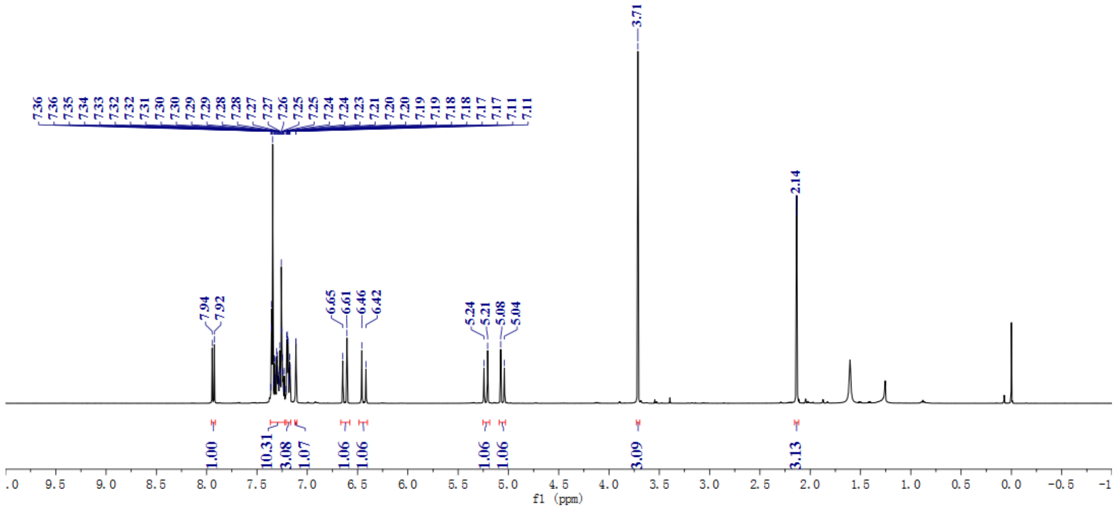

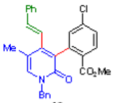

4O

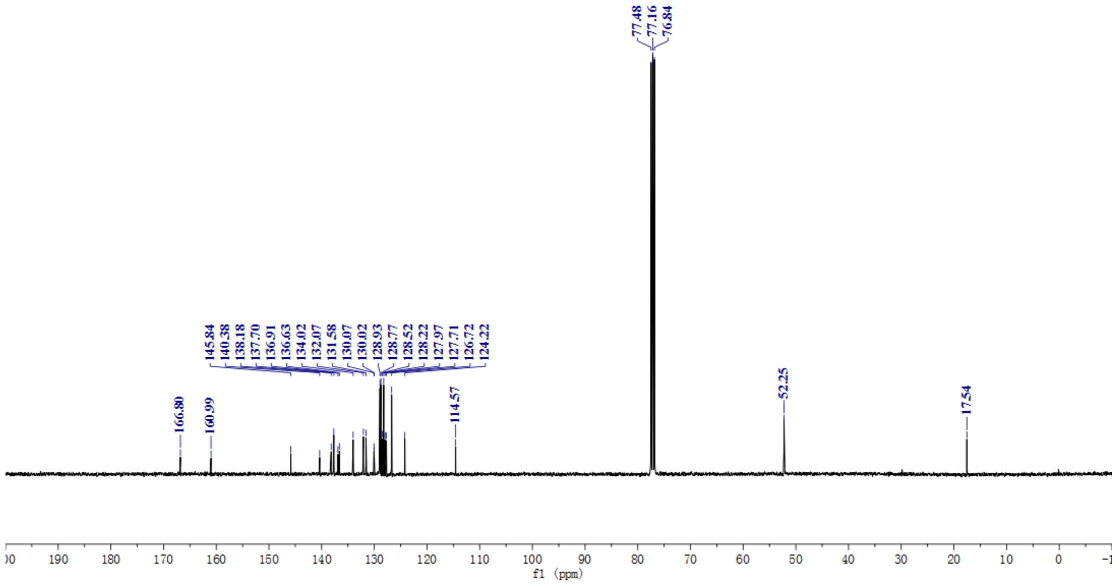

Supplementary Figure 79. NMR of **4O**

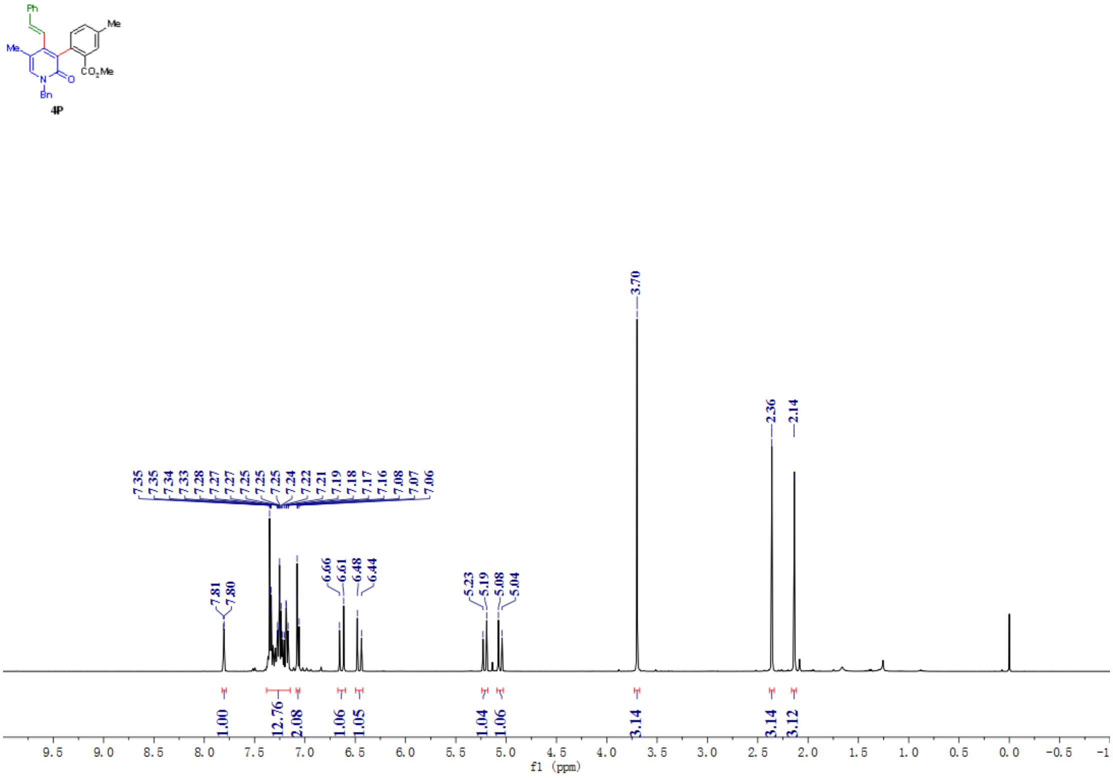

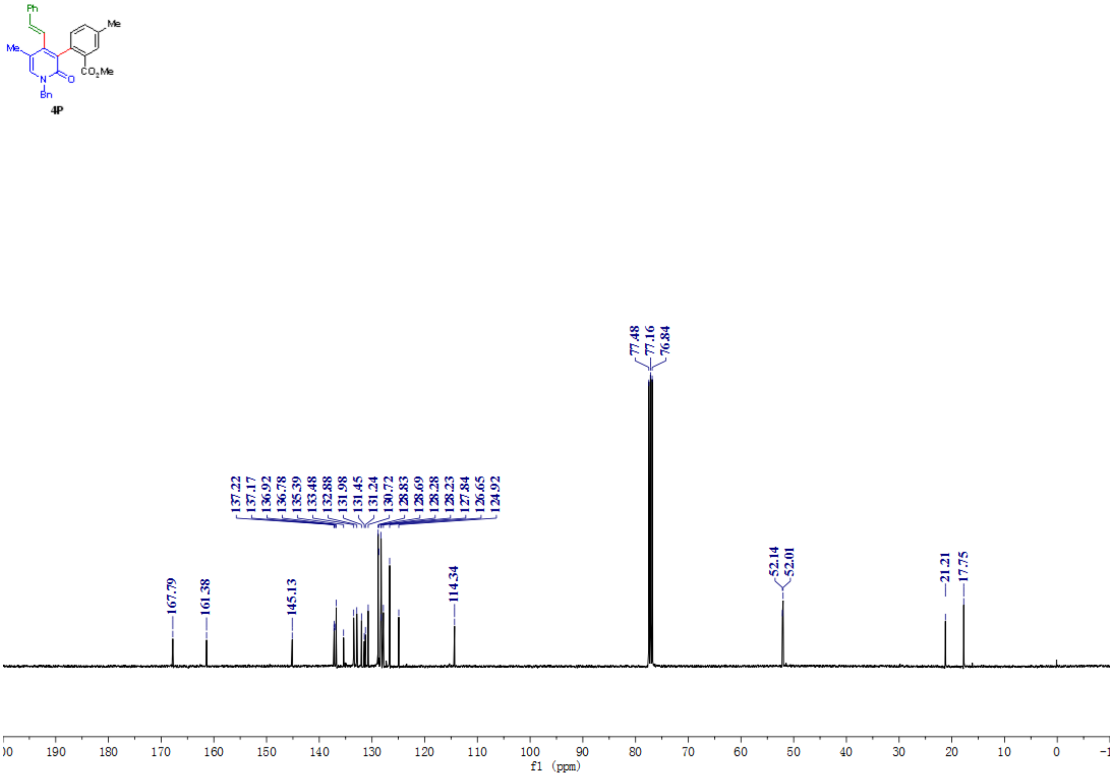

Supplementary Figure 80. NMR of **4P**

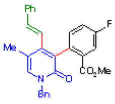

4Q

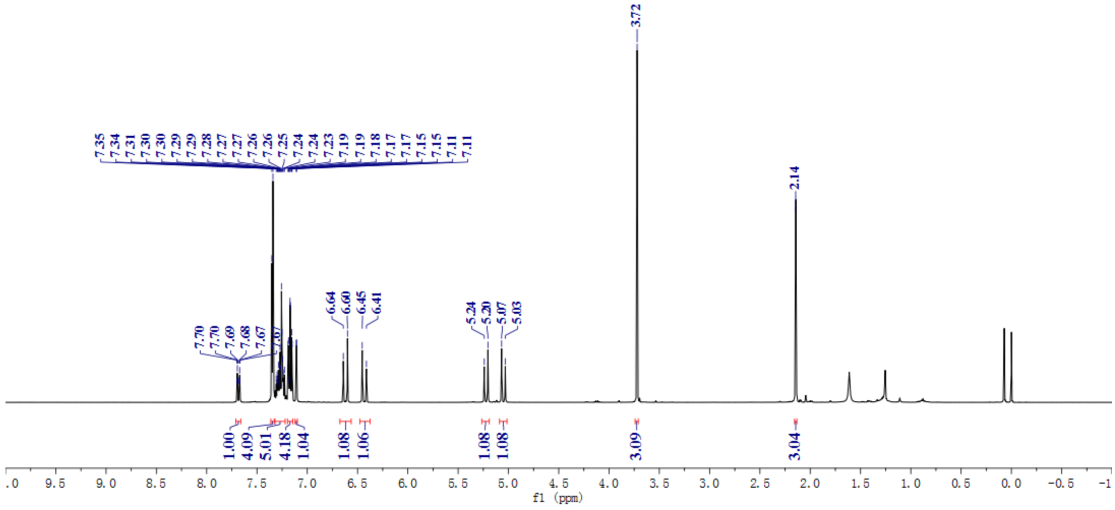

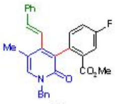

4Q

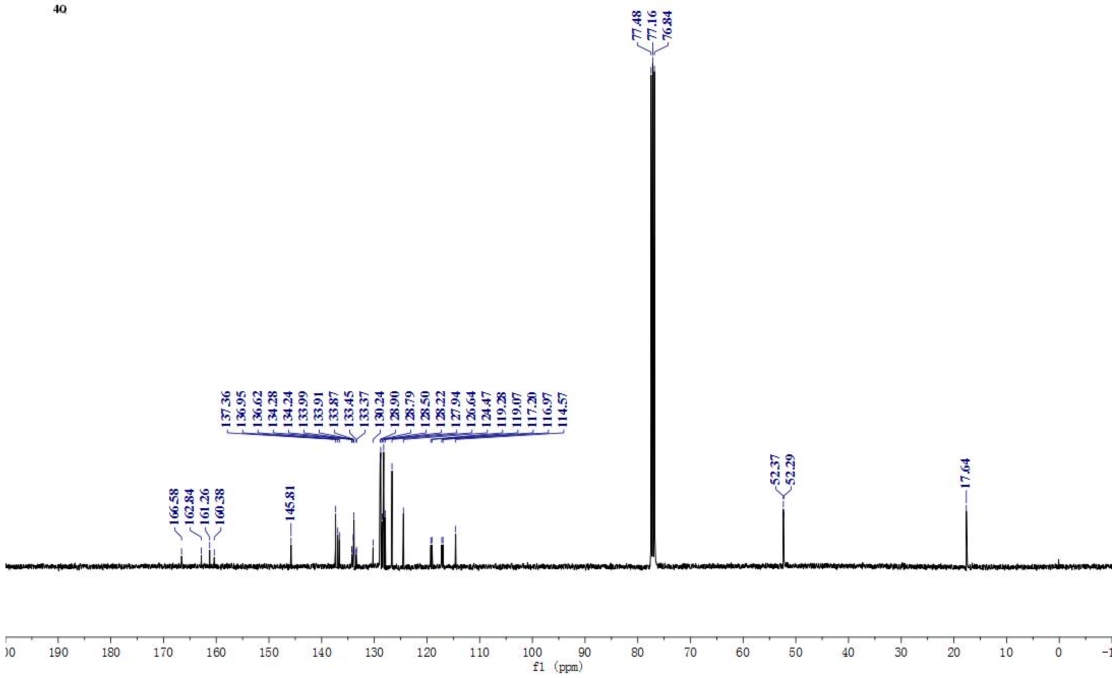

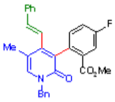

4Q

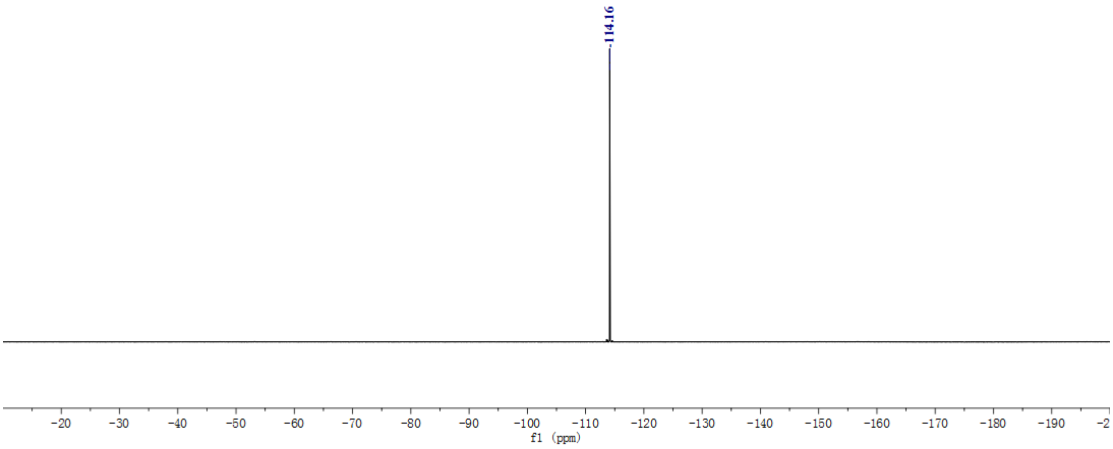

Supplementary Figure 81. NMR of **4Q**

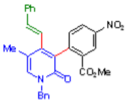

4R

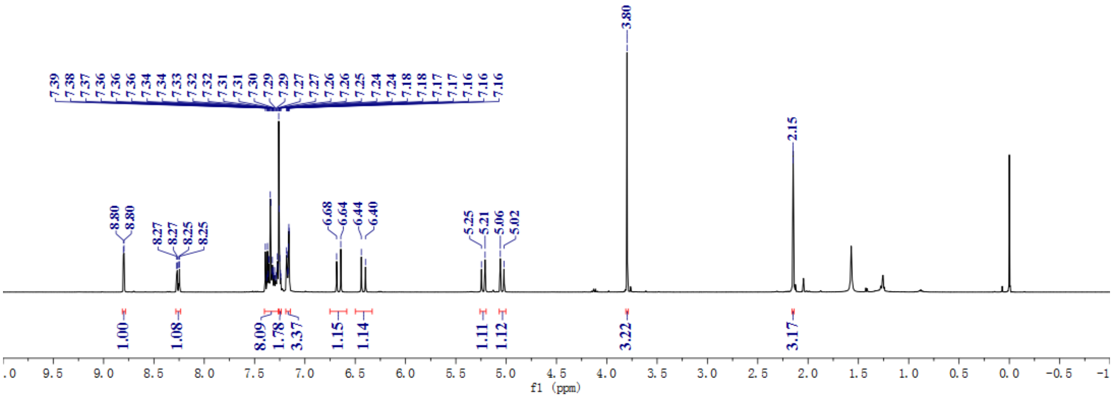

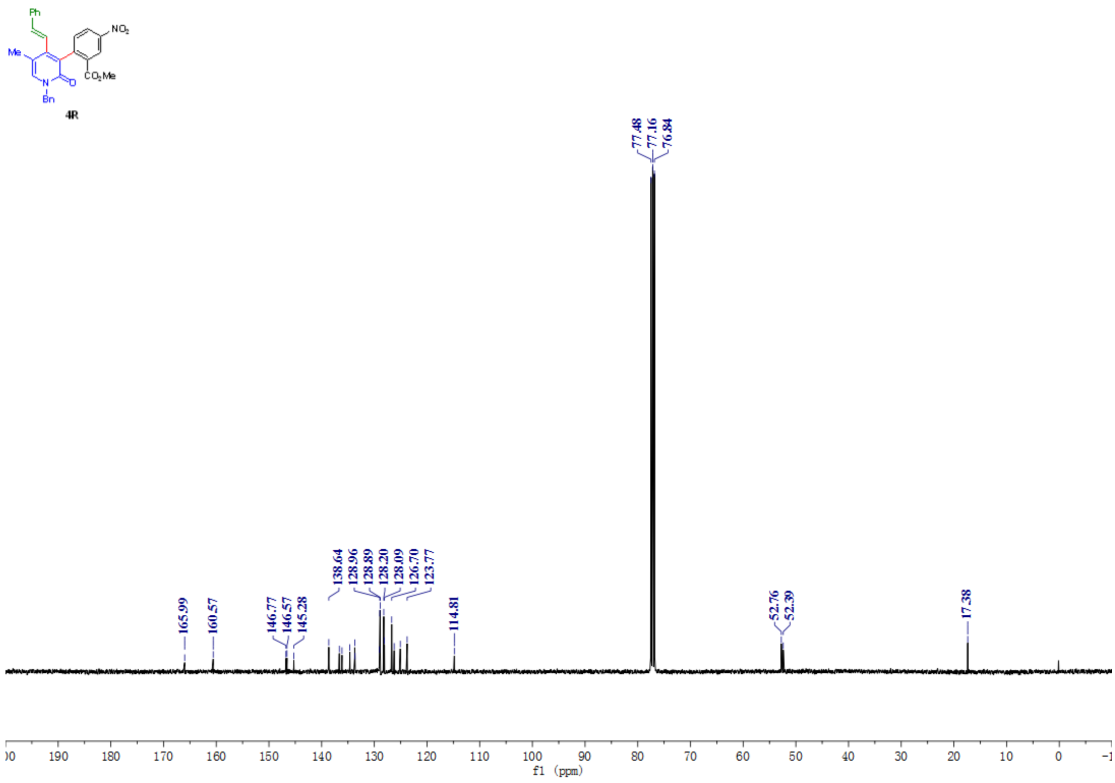

Supplementary Figure 82. NMR of **4R**

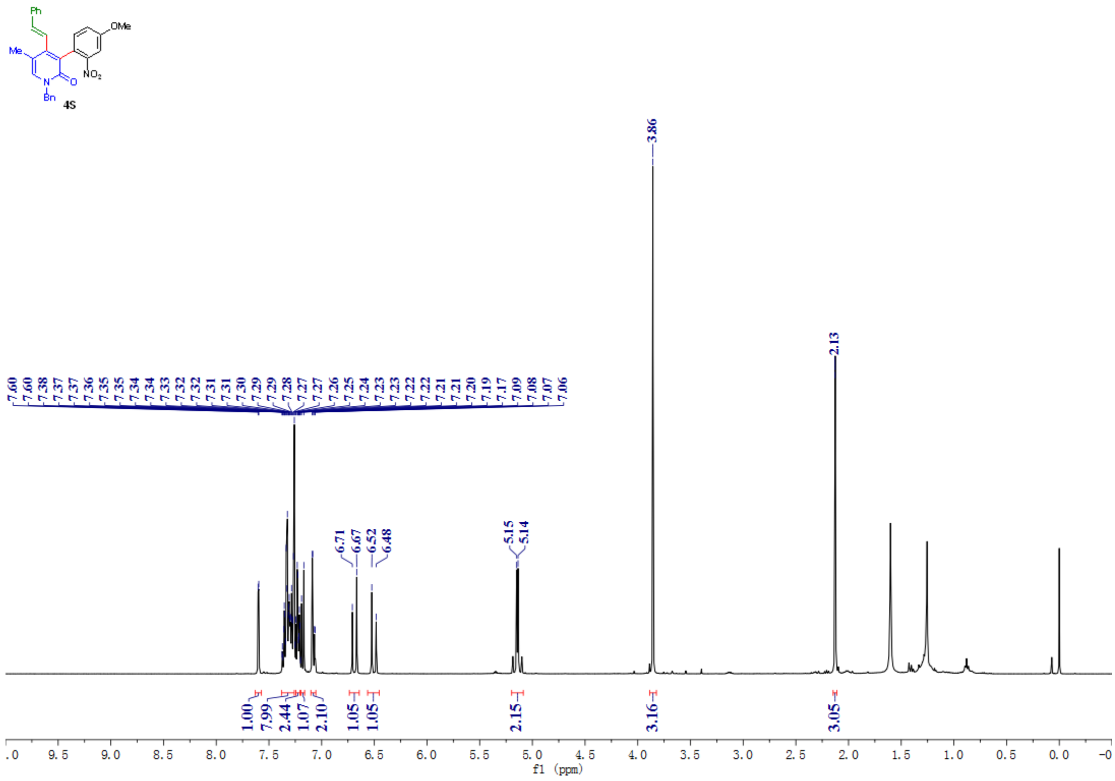

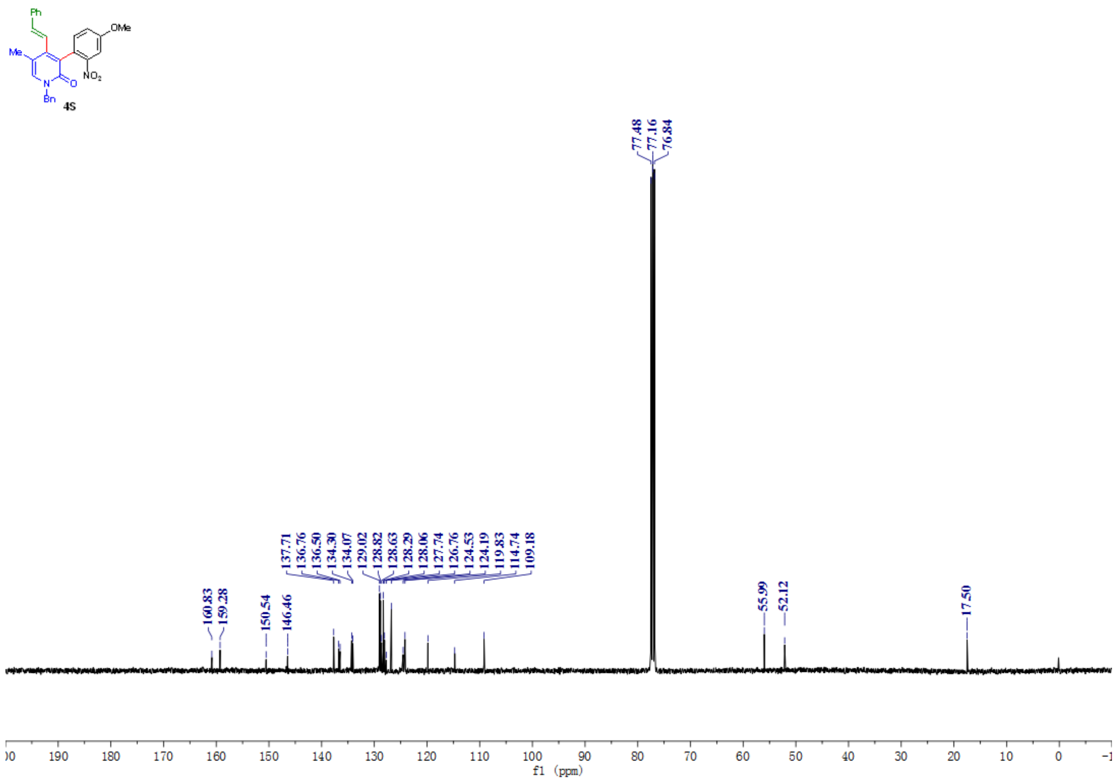

Supplementary Figure 83. NMR of **4S**

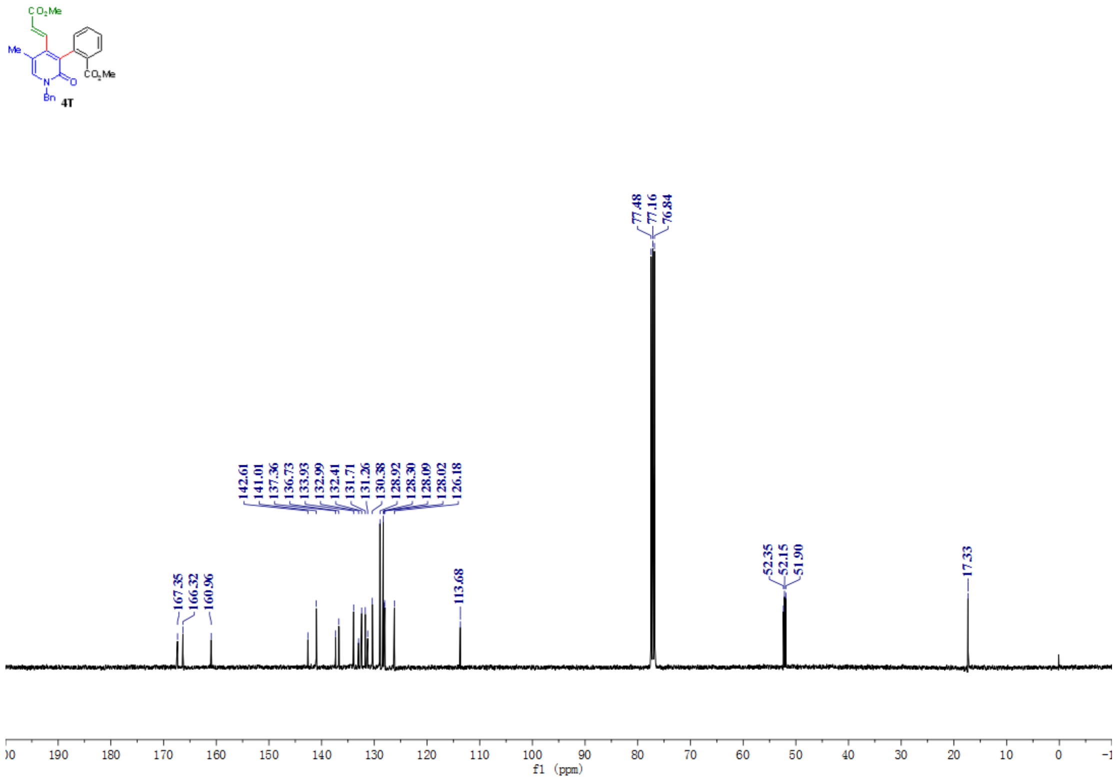

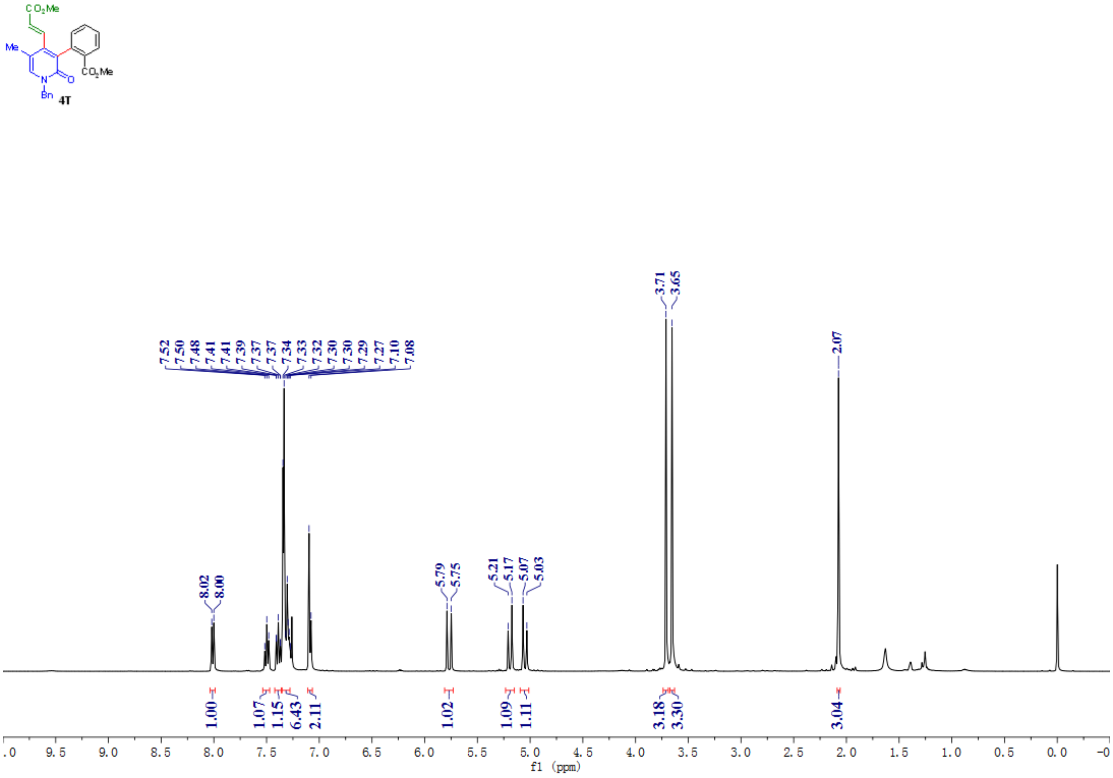

Supplementary Figure 84. NMR of **4T**

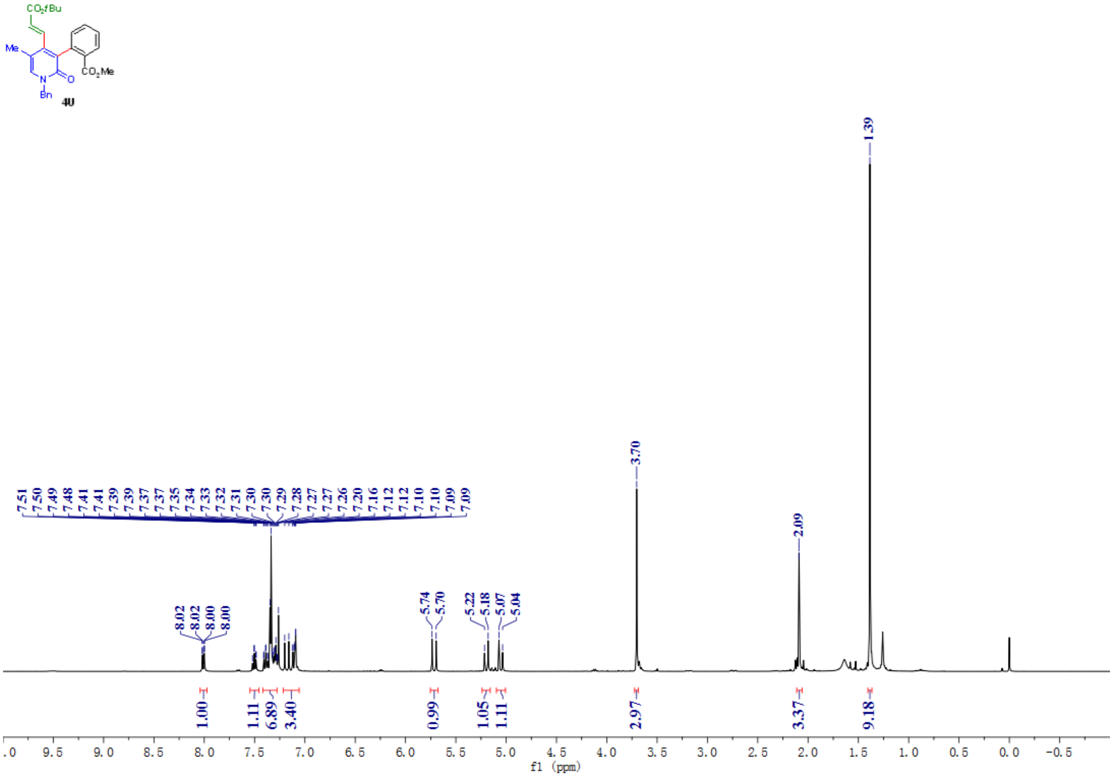

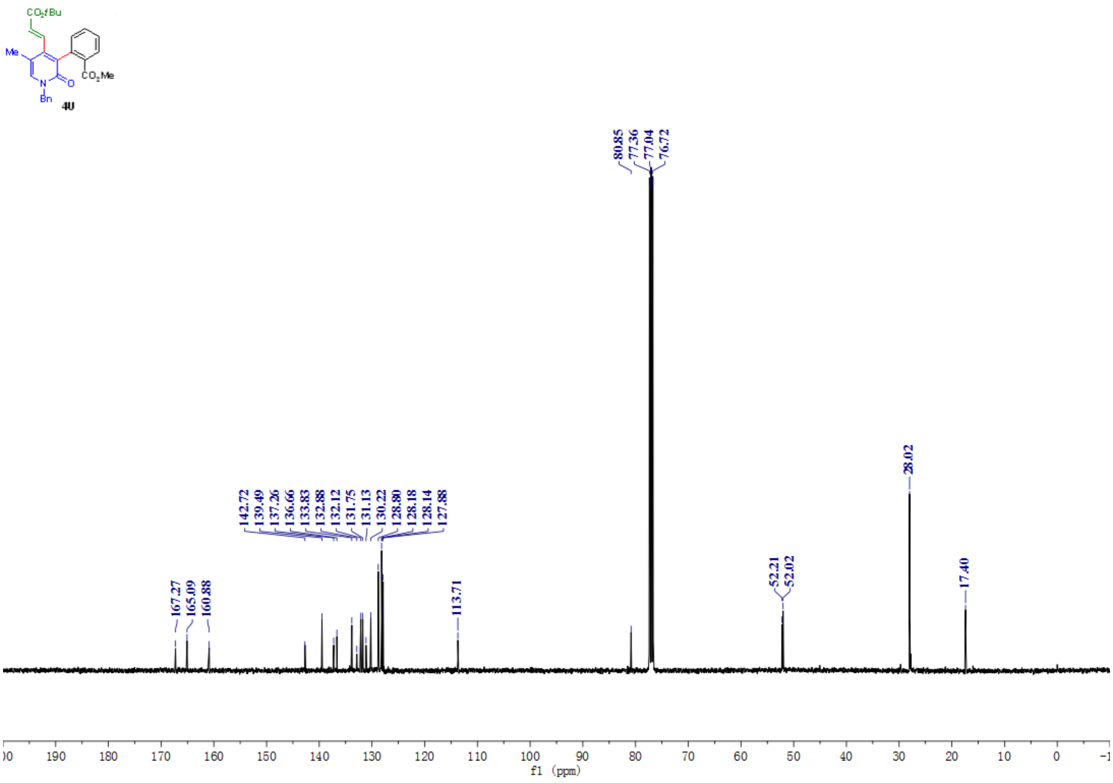

Supplementary Figure 85. NMR of **4U**

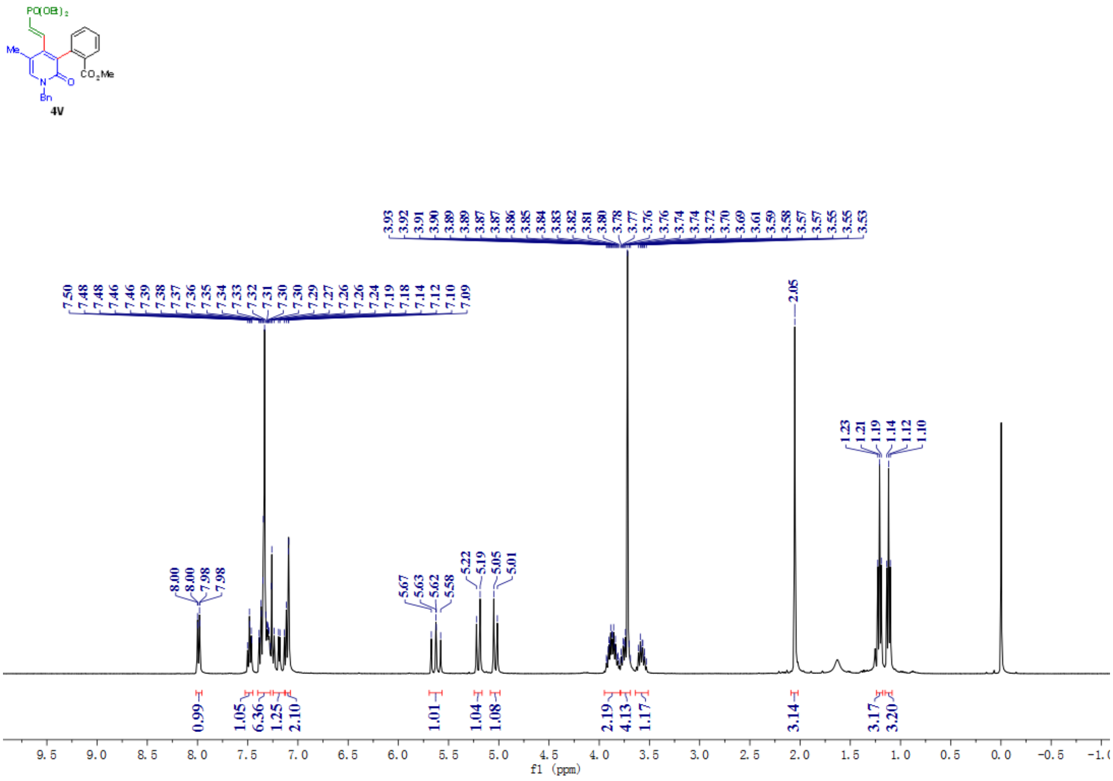

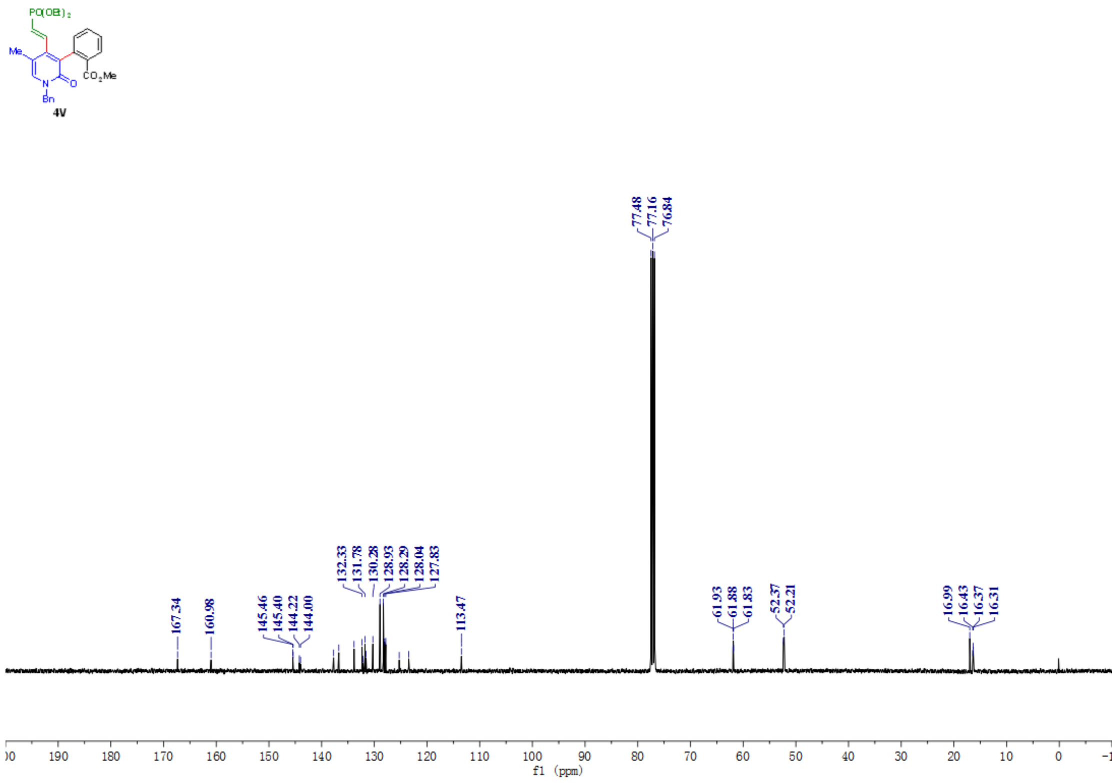

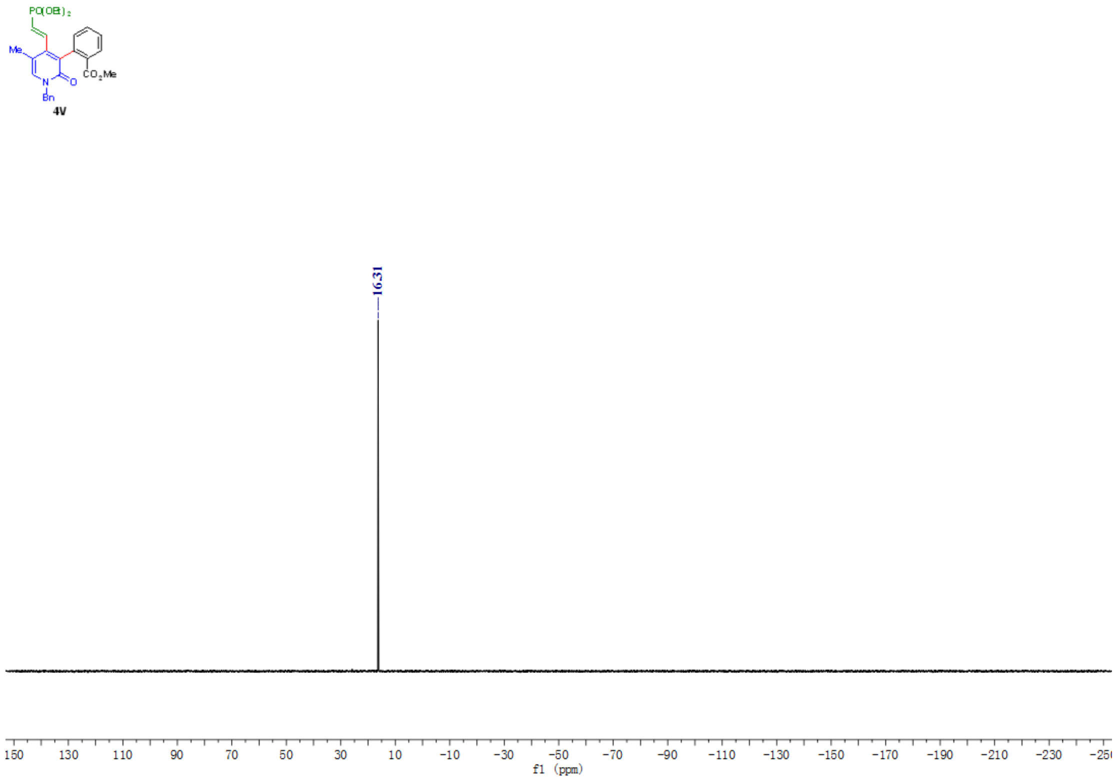

Supplementary Figure 86. NMR of **4V**

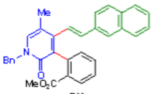

4W

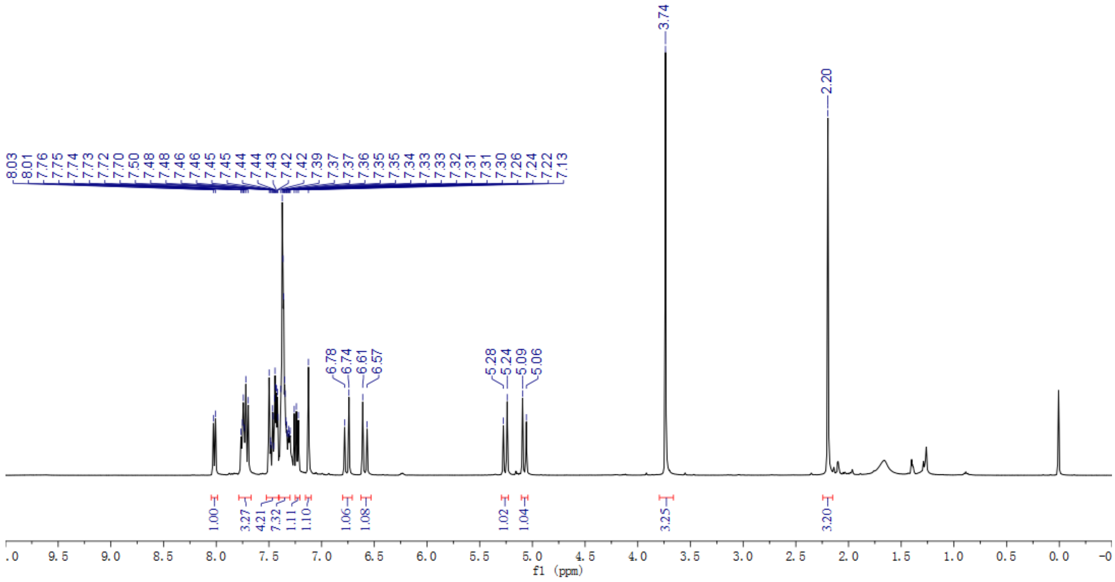

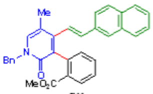

4W

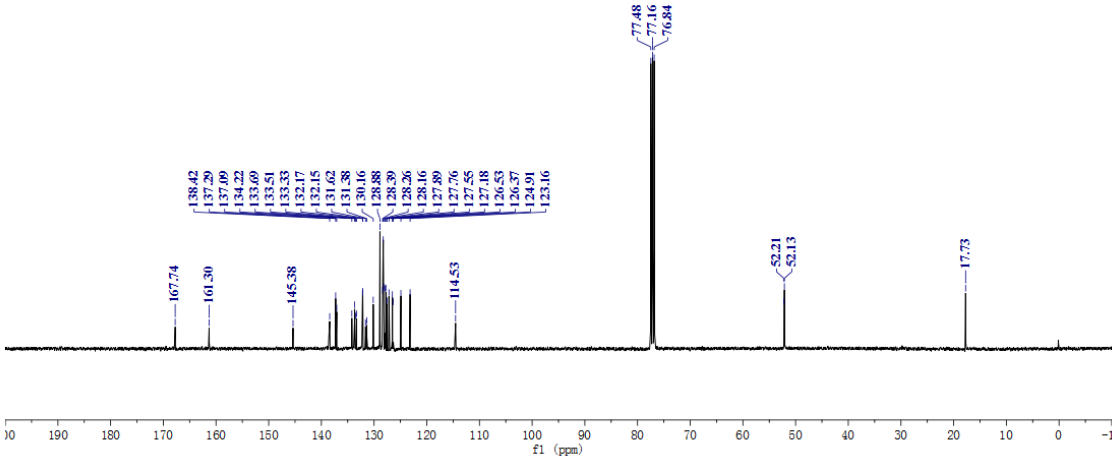

Supplementary Figure 87. NMR of **4W**

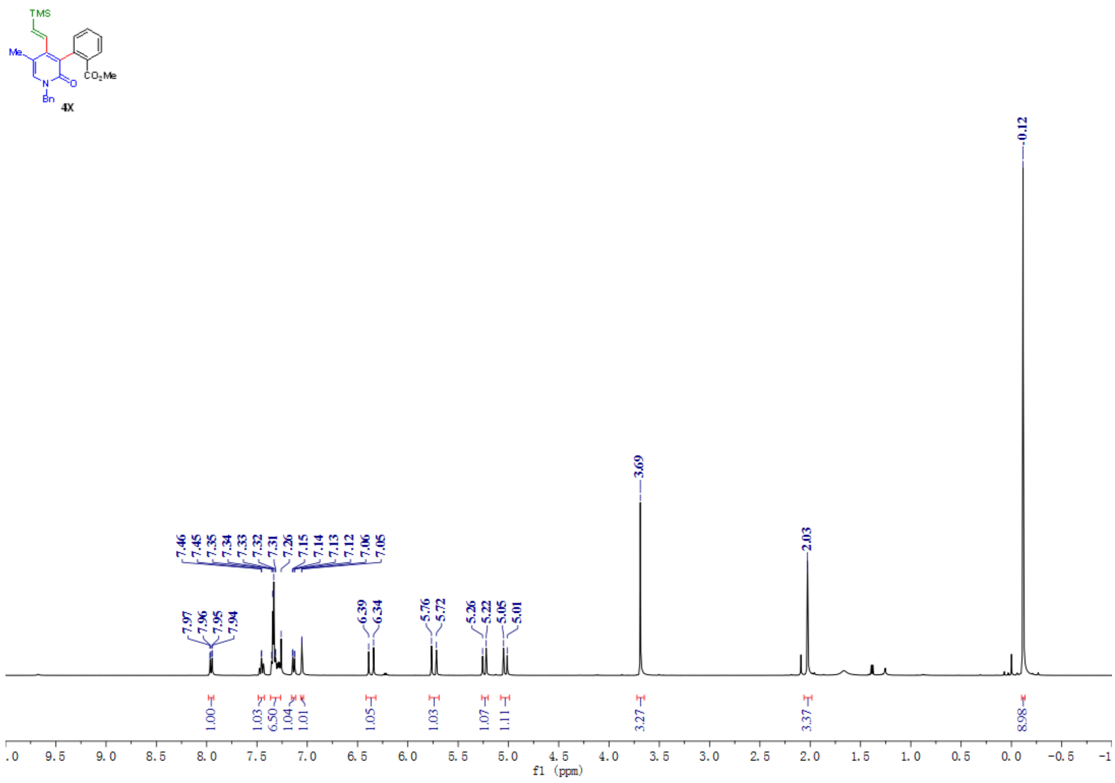

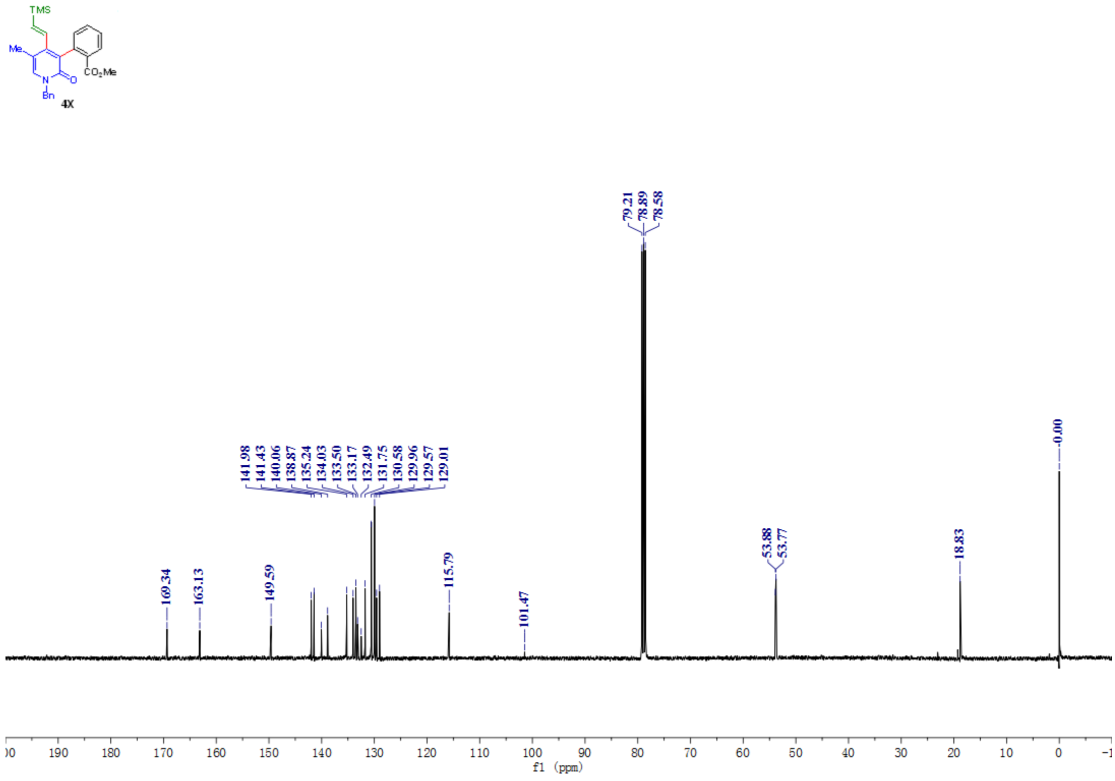

Supplementary Figure 88. NMR of **4X**

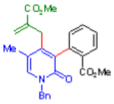

4Y

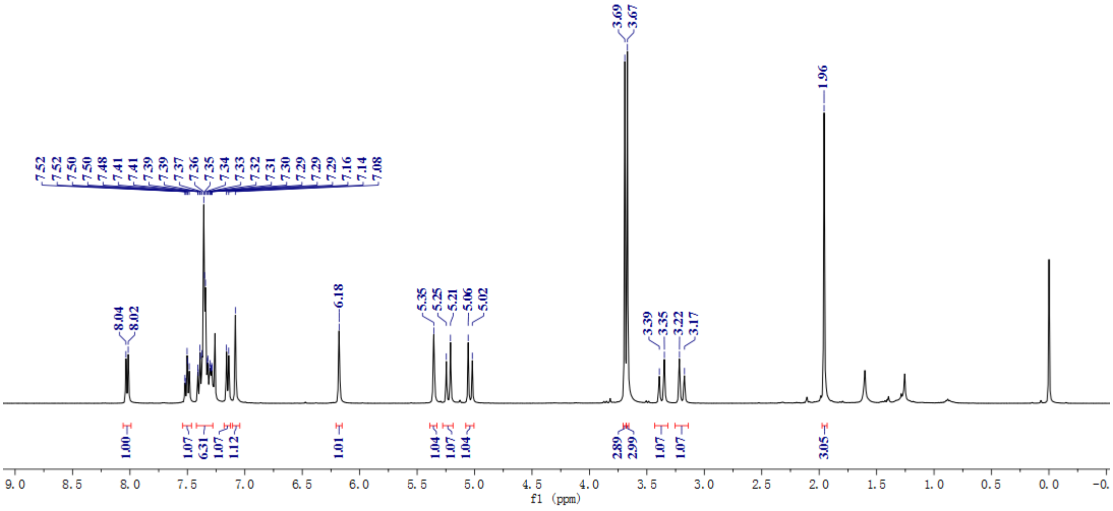

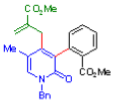

4Y

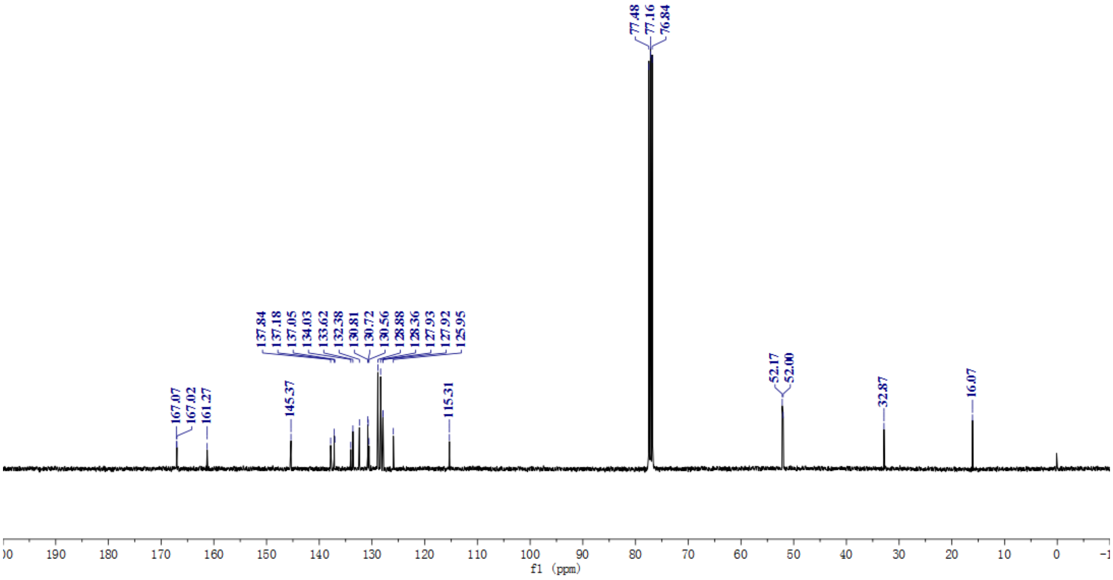

Supplementary Figure 89. NMR of **4Y**

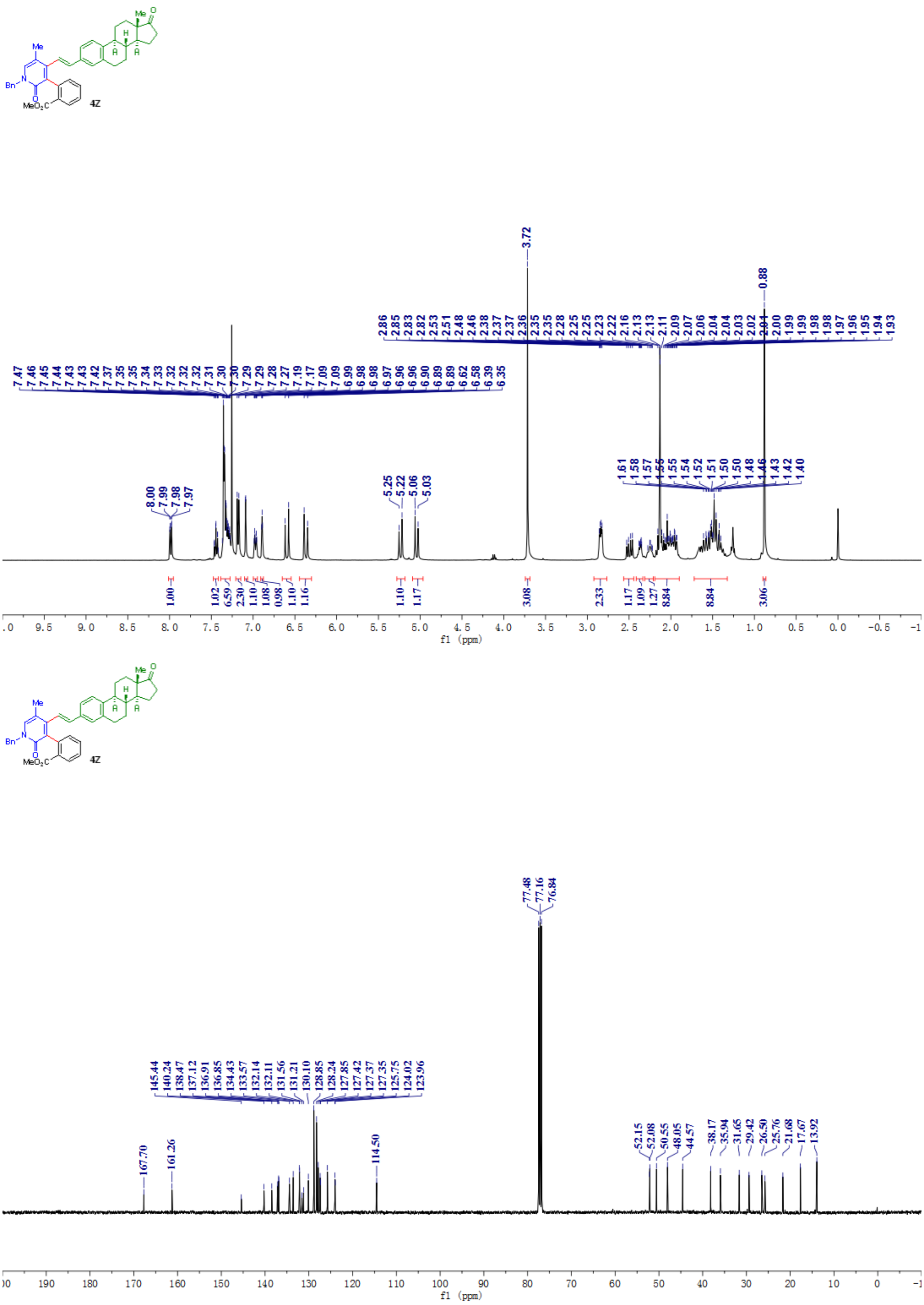

Supplementary Figure 90. NMR of **4Z**

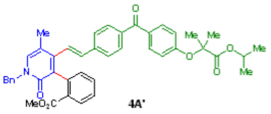

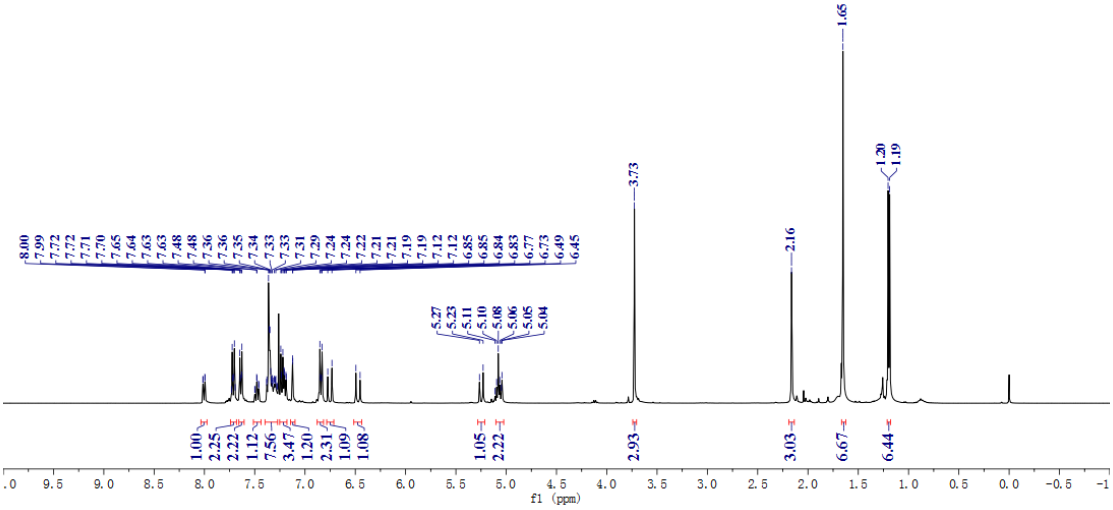

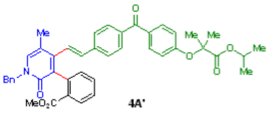

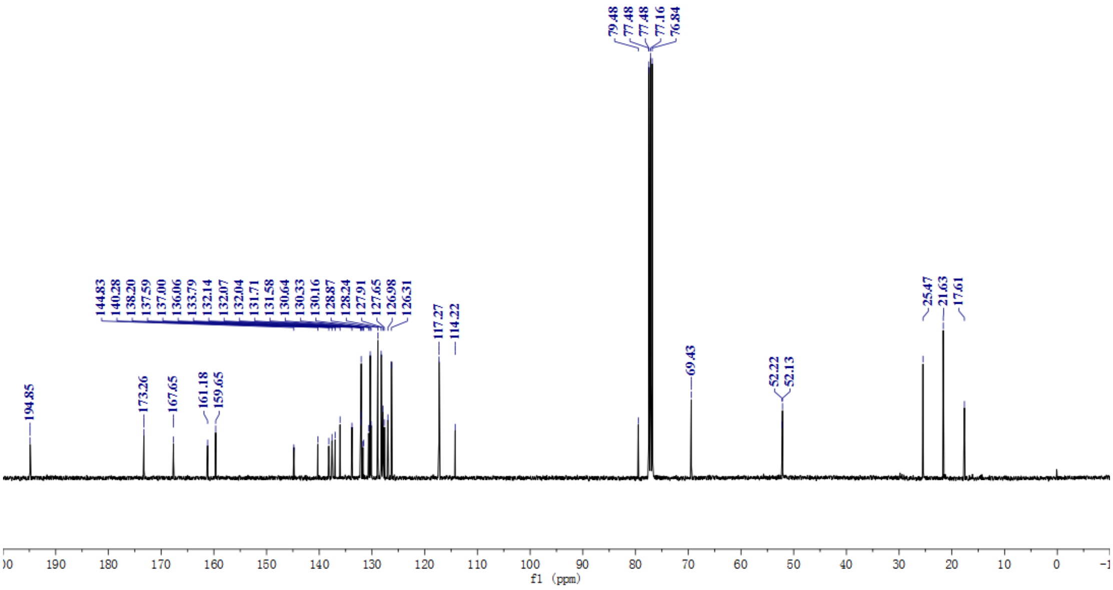

Supplementary Figure 91. NMR of **4A'**

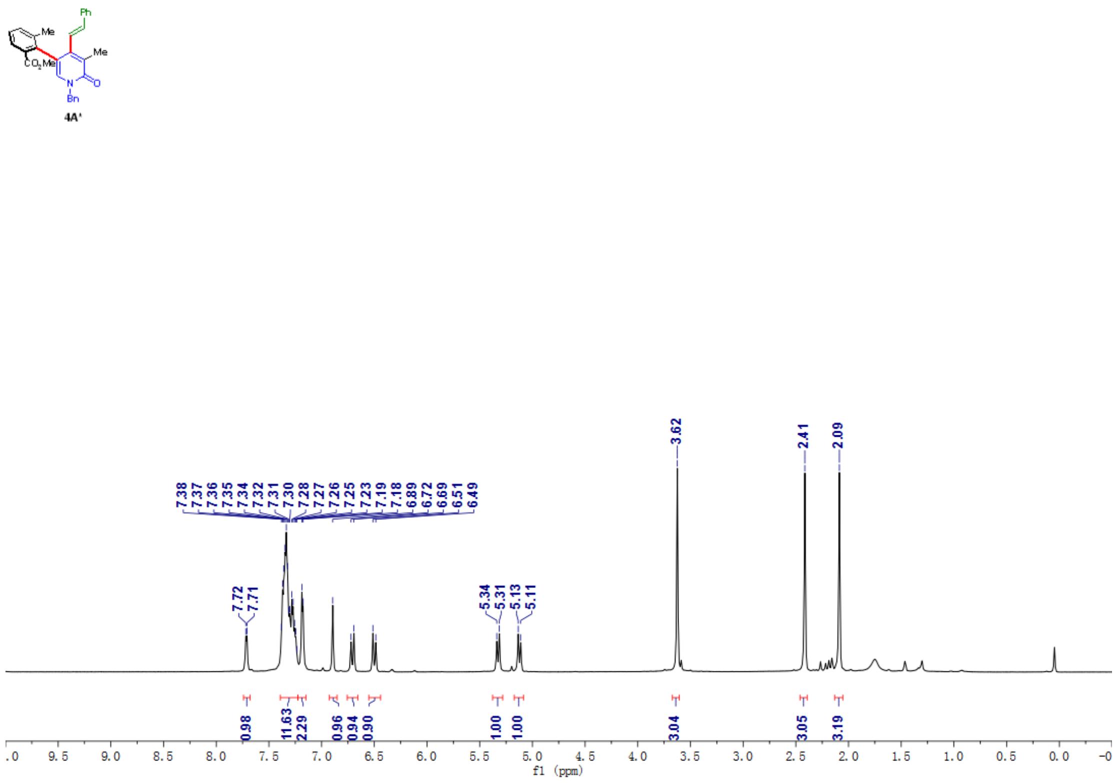

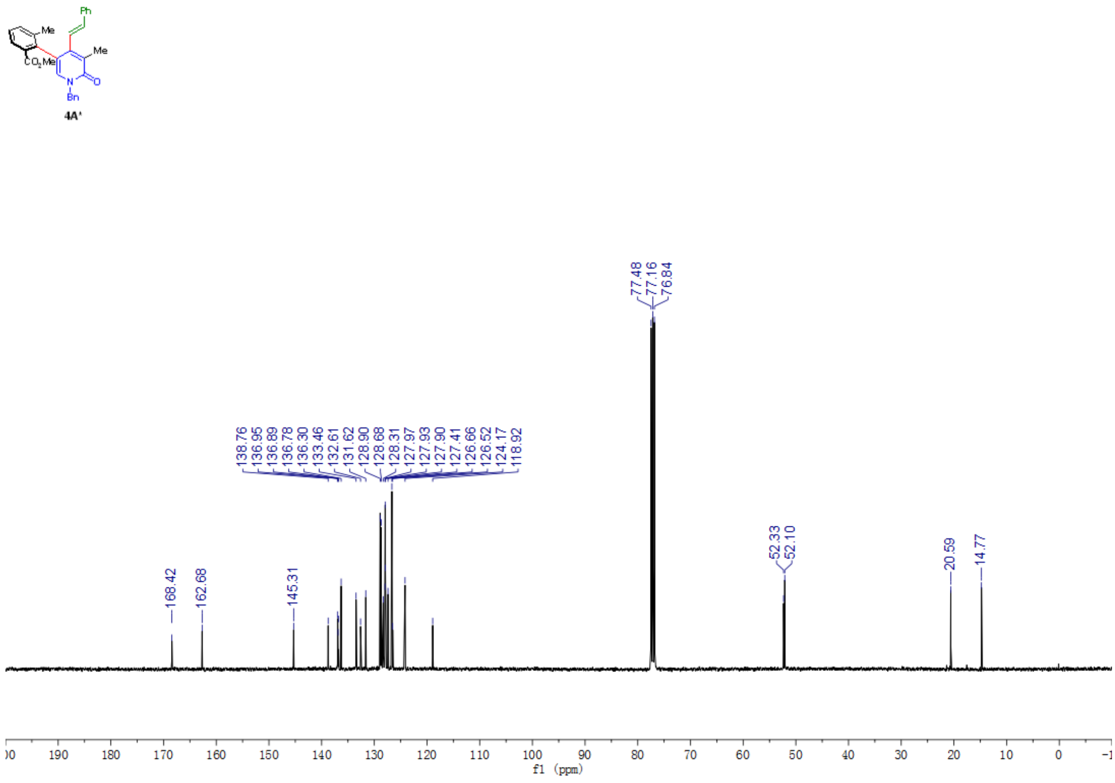

Supplementary Figure 92. NMR of **4A***

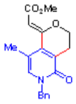

7

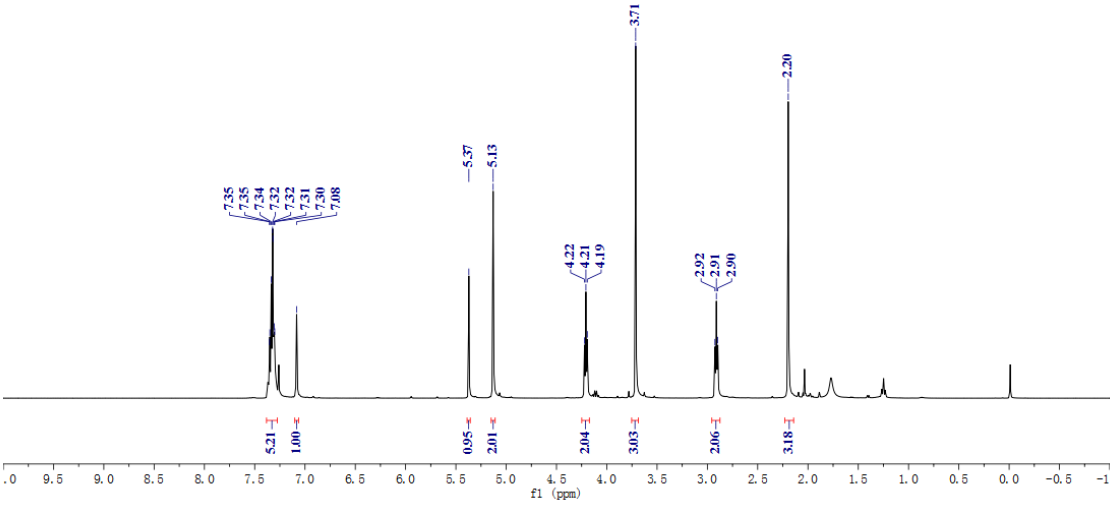

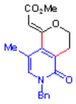

7

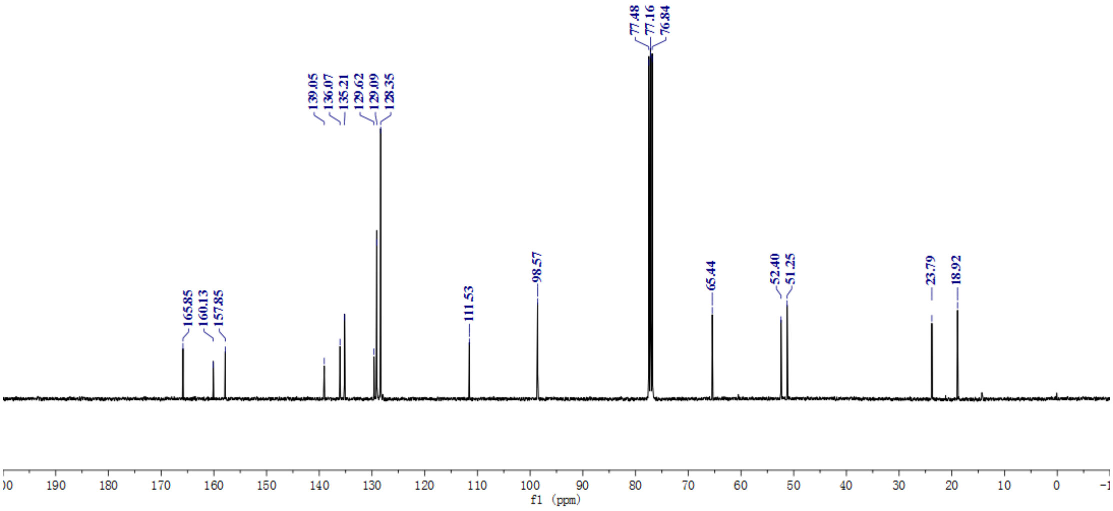

Supplementary Figure 93. NMR of **7**

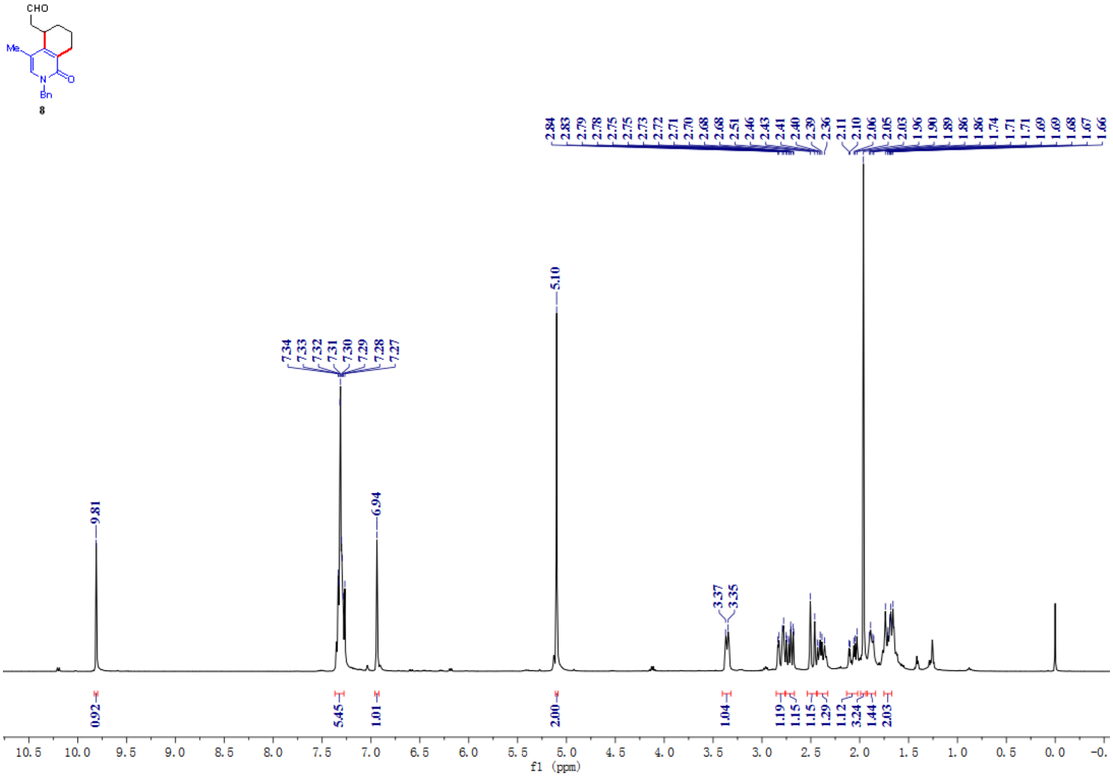

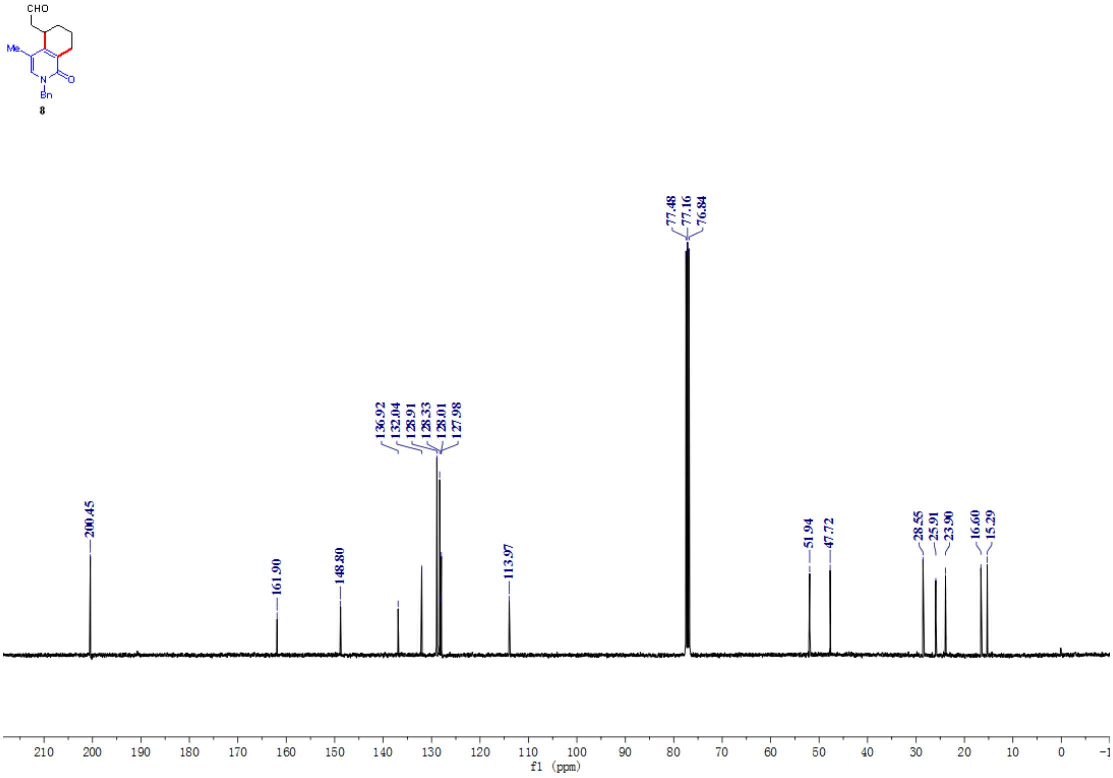

Supplementary Figure 94. NMR of **8**

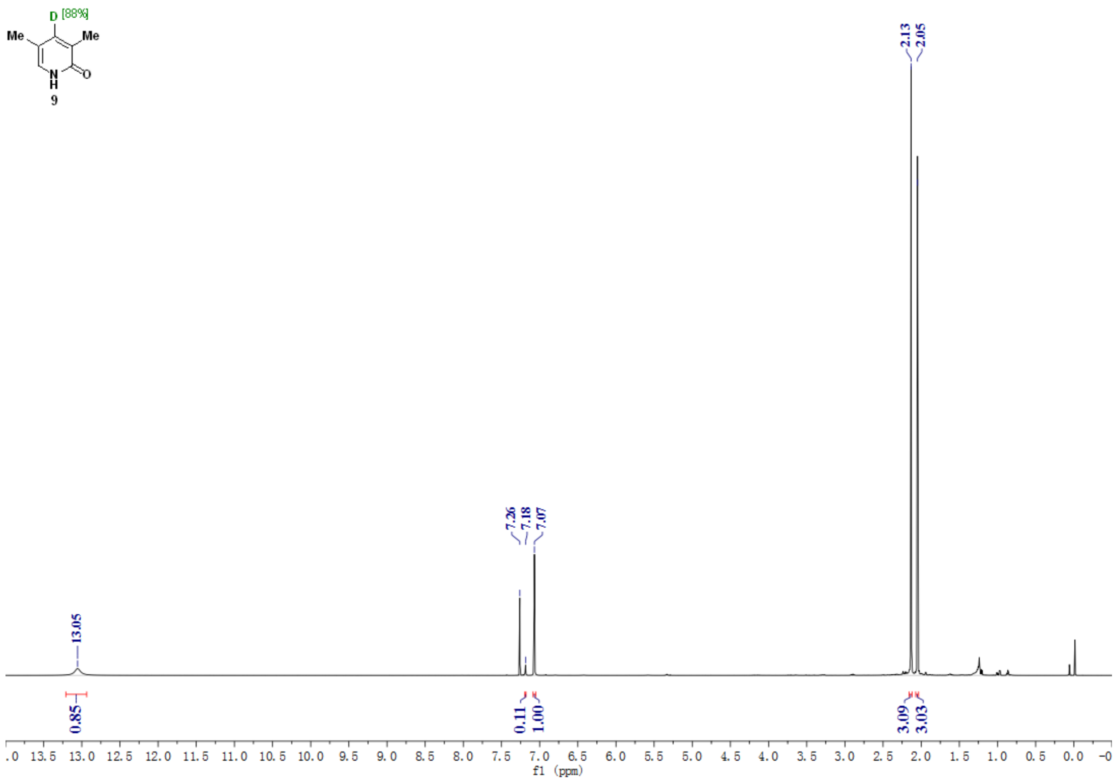

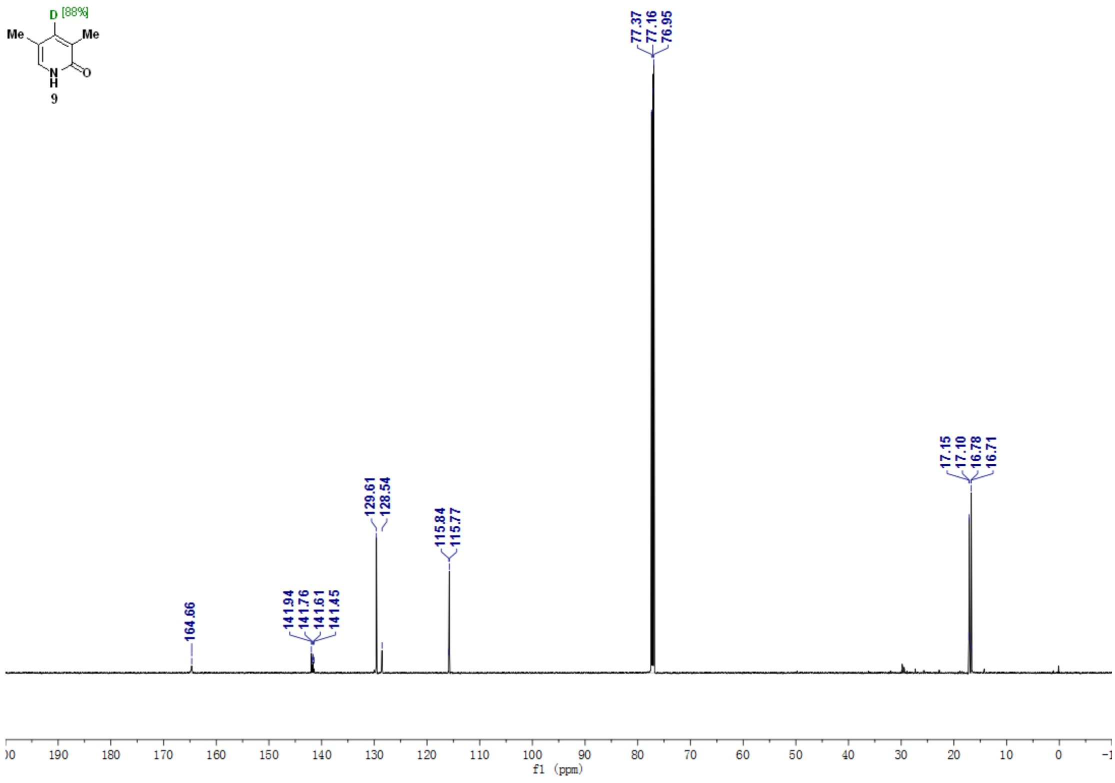

Supplementary Figure 95. NMR of **9**

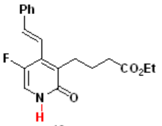
Ph
F
CO2Et
N
H
O

10

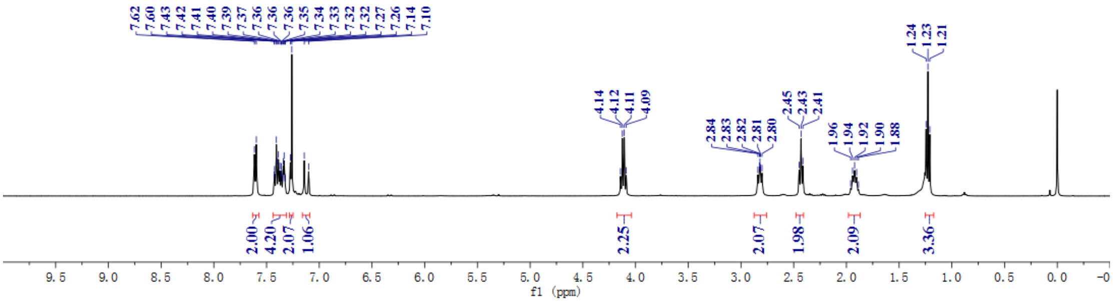
7.62 7.60 7.43 7.42 7.41 7.40 7.39 7.37 7.36 7.36 7.36 7.35 7.34 7.33 7.32 7.32 7.27 7.26 7.14 7.10
4.14 4.12 4.11 4.09
2.84 2.83 2.82 2.81 2.80
2.45 2.43 2.41
1.96 1.94 1.92 1.90 1.88
1.24 1.23 1.21
2.00 4.20 2.07 1.06 2.25 2.07 1.98 2.09 3.36
f1 (ppm)

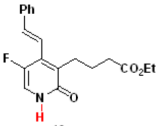
Ph
F
CO2Et
N
H
O

10

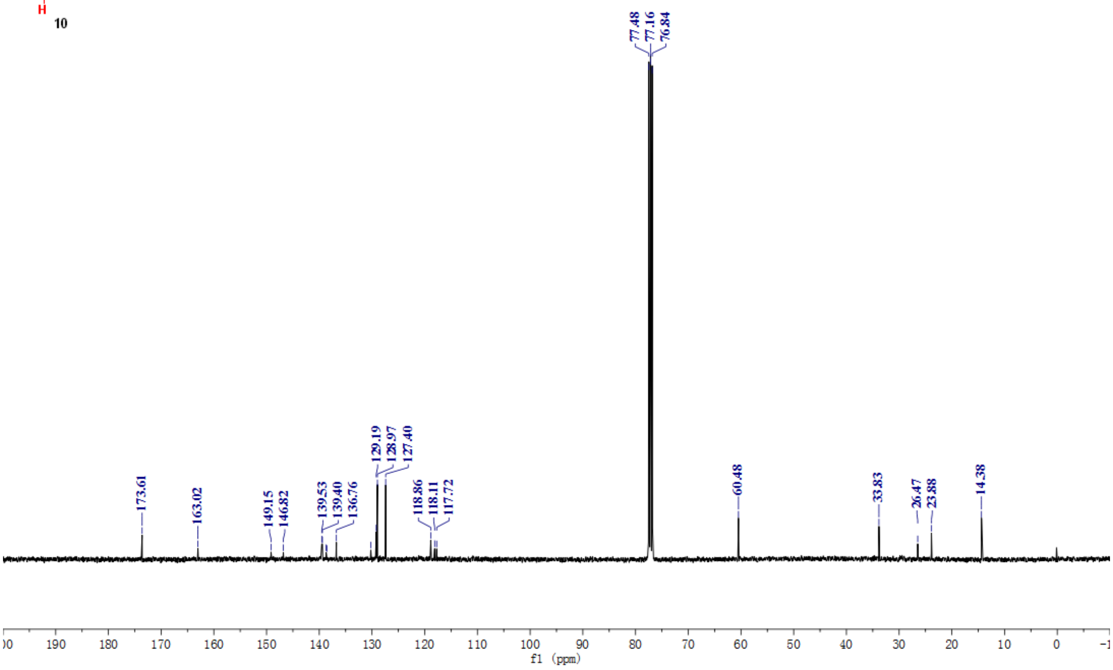
77.48 77.16 76.84
173.61
163.02
149.15 146.82
139.53 139.40 136.76
129.19 128.97 127.40
118.86 118.11 117.72
60.48
33.83
26.47 23.88
14.38
f1 (ppm)

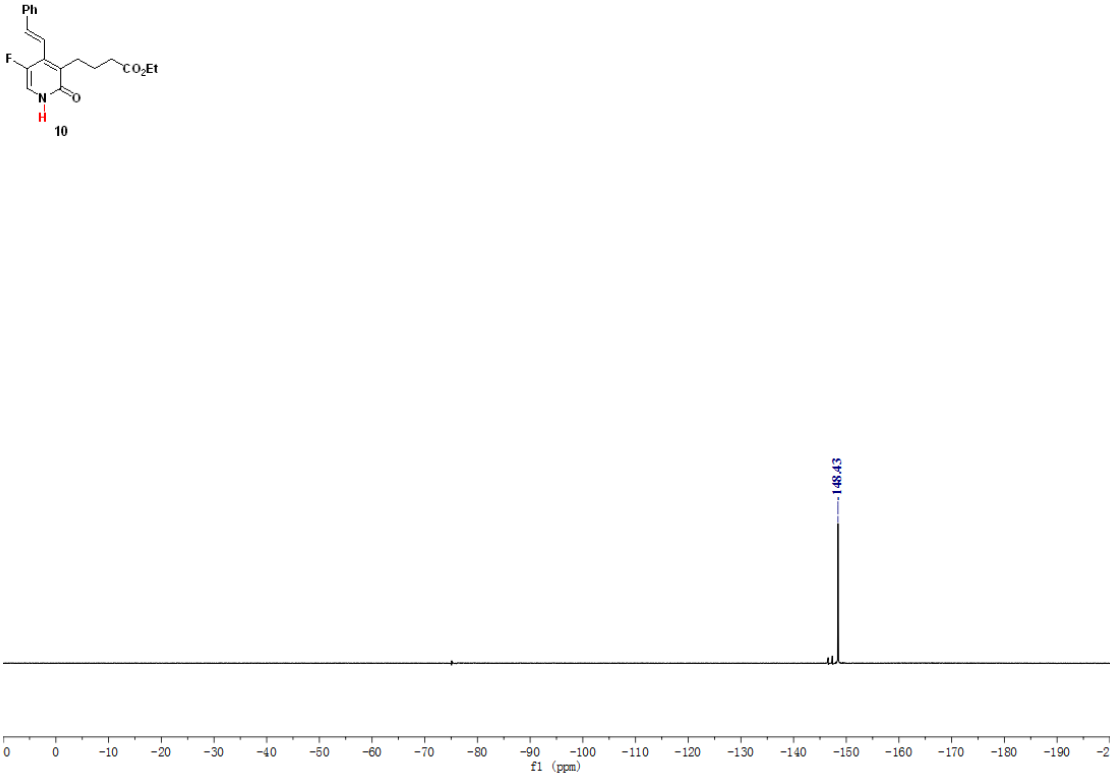

Supplementary Figure 96. NMR of **10**

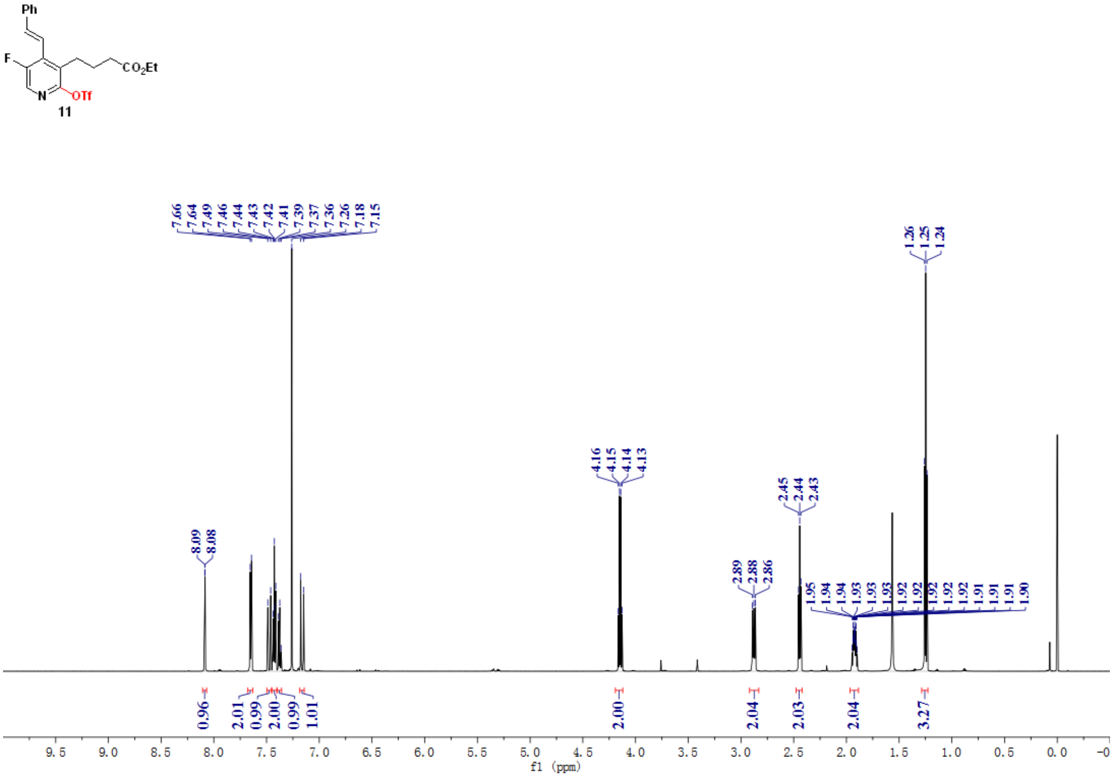

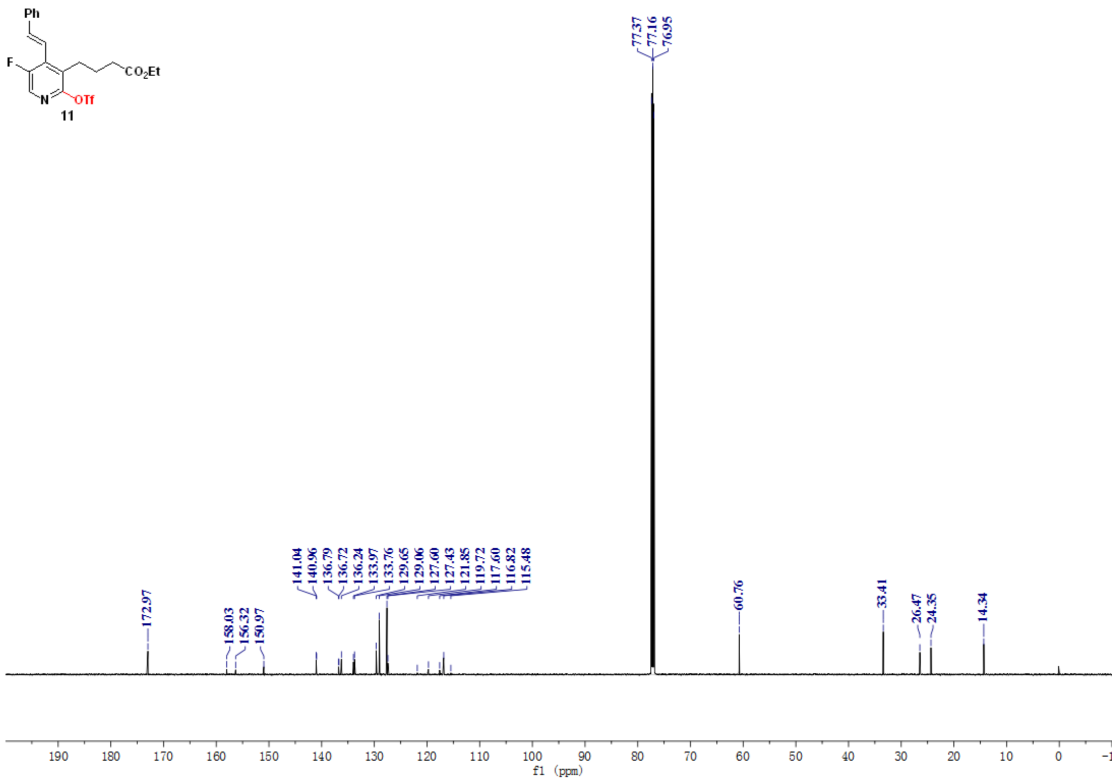

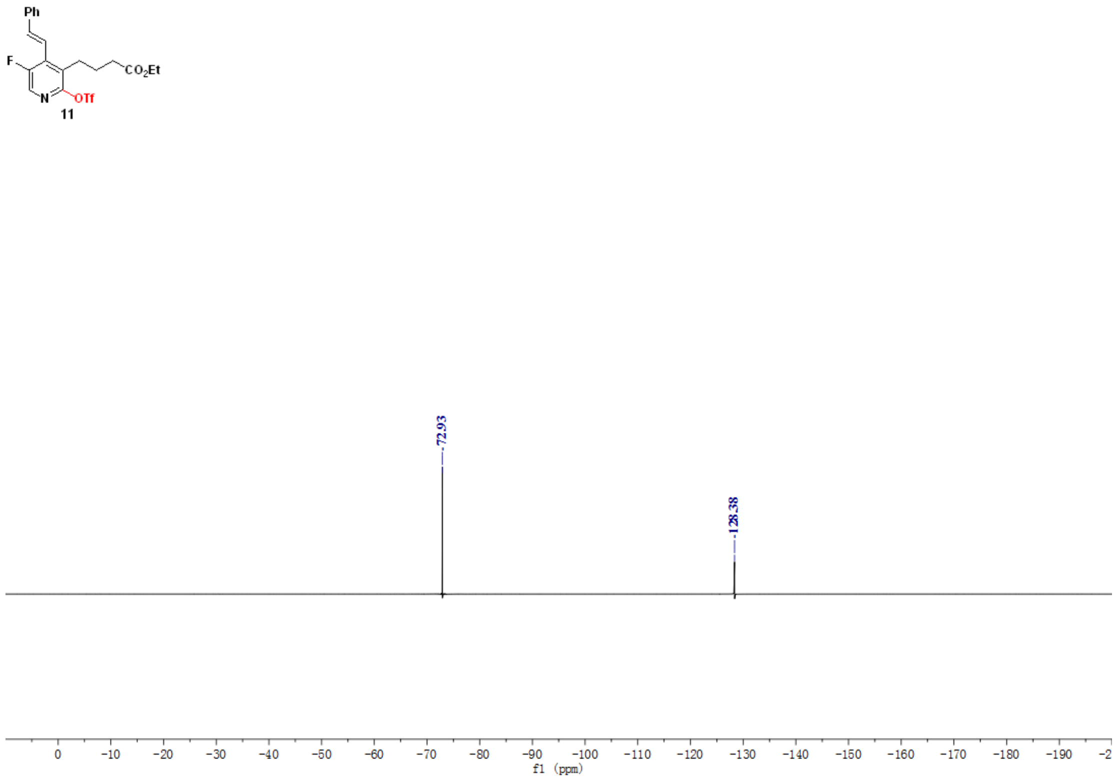

Supplementary Figure 97. NMR of **11**

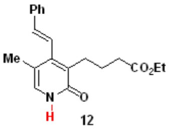

12

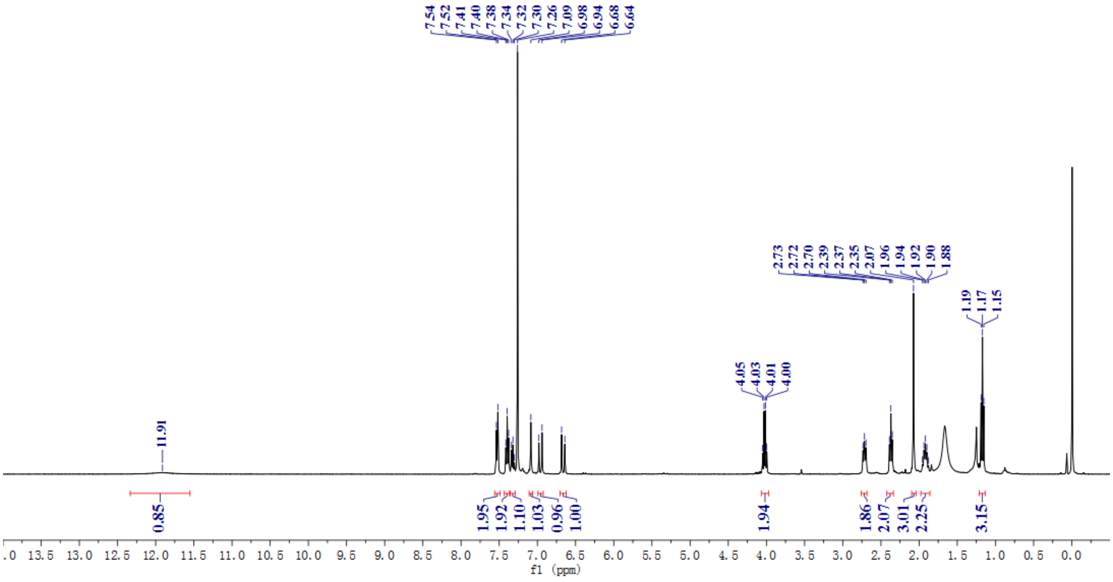

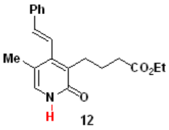

12

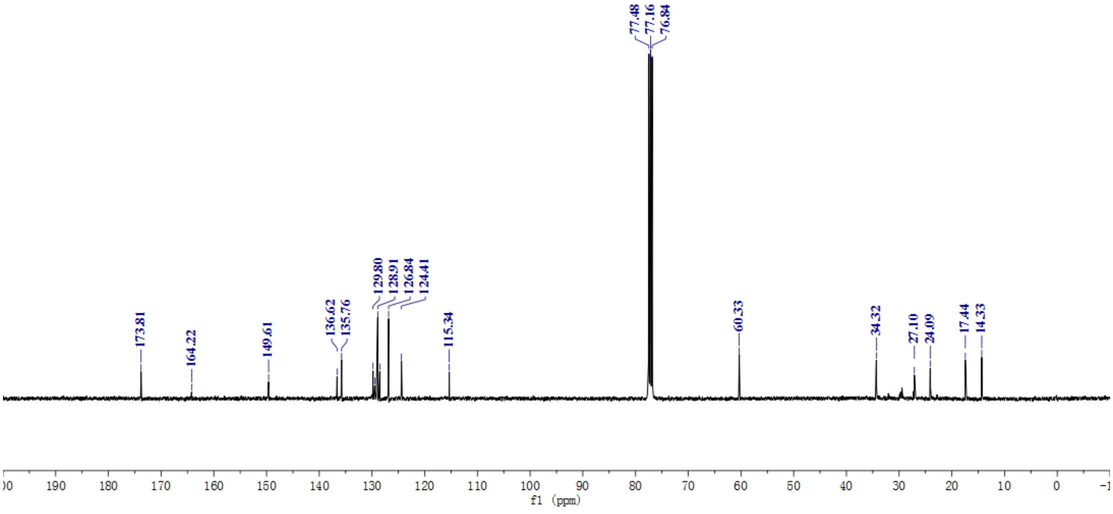

Supplementary Figure 98. NMR of **12**

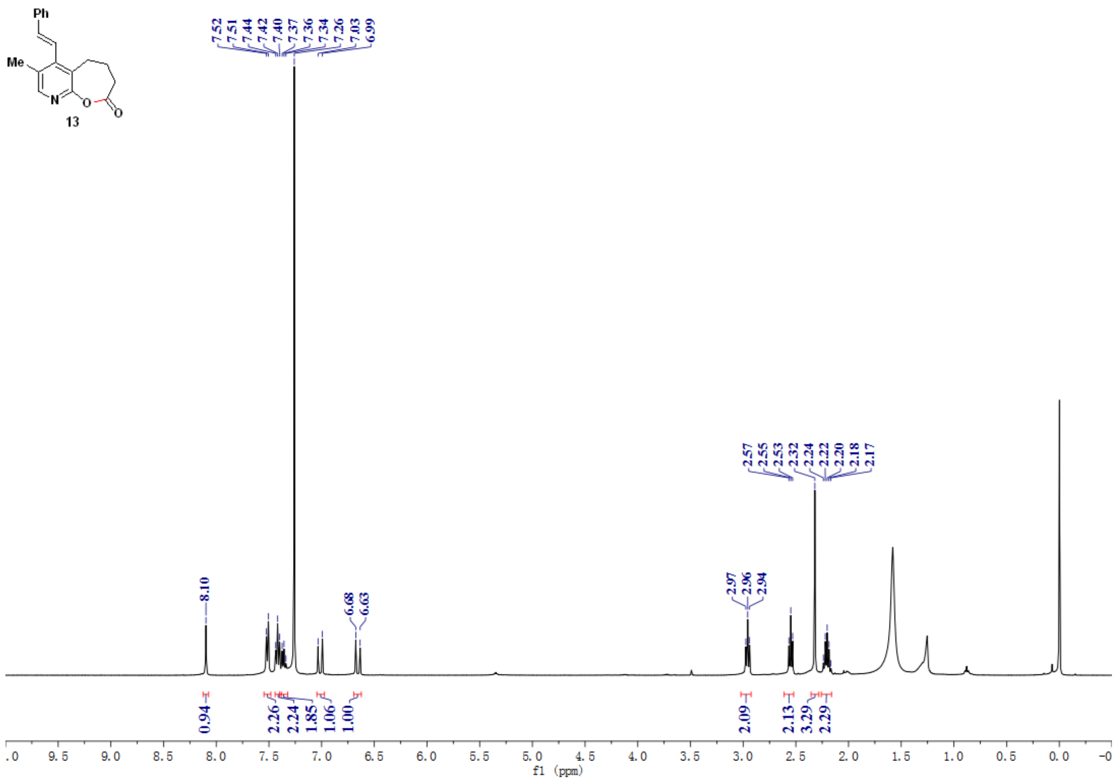

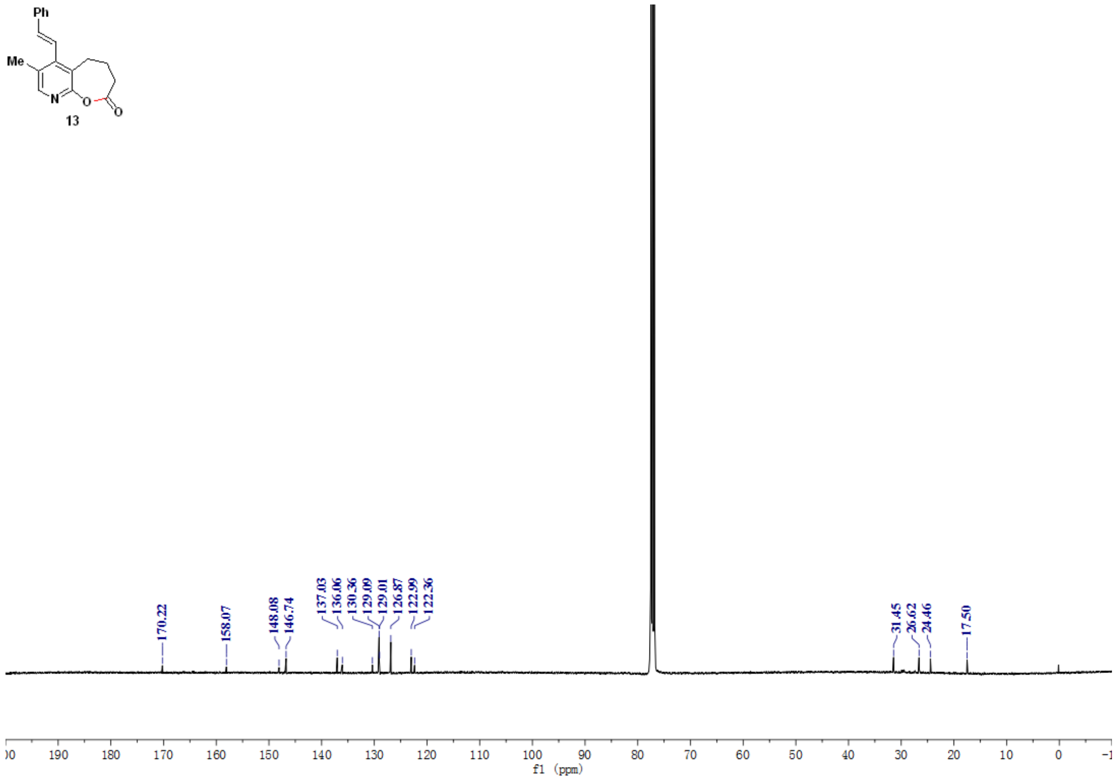

Supplementary Figure 99. NMR of **13**

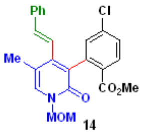

14

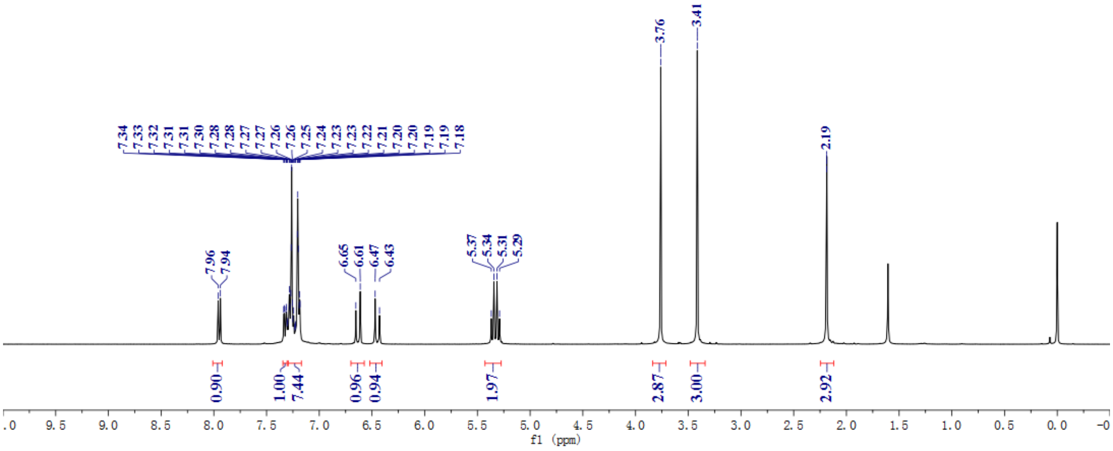

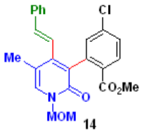

14

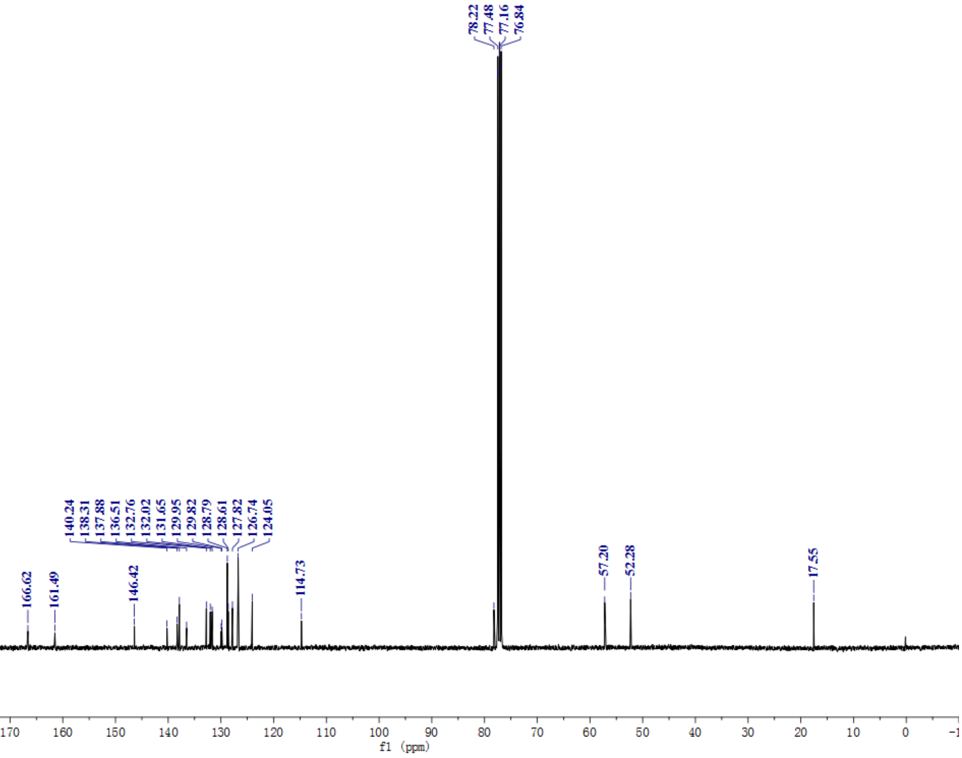

Supplementary Figure 100. NMR of **14**

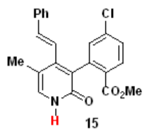

15

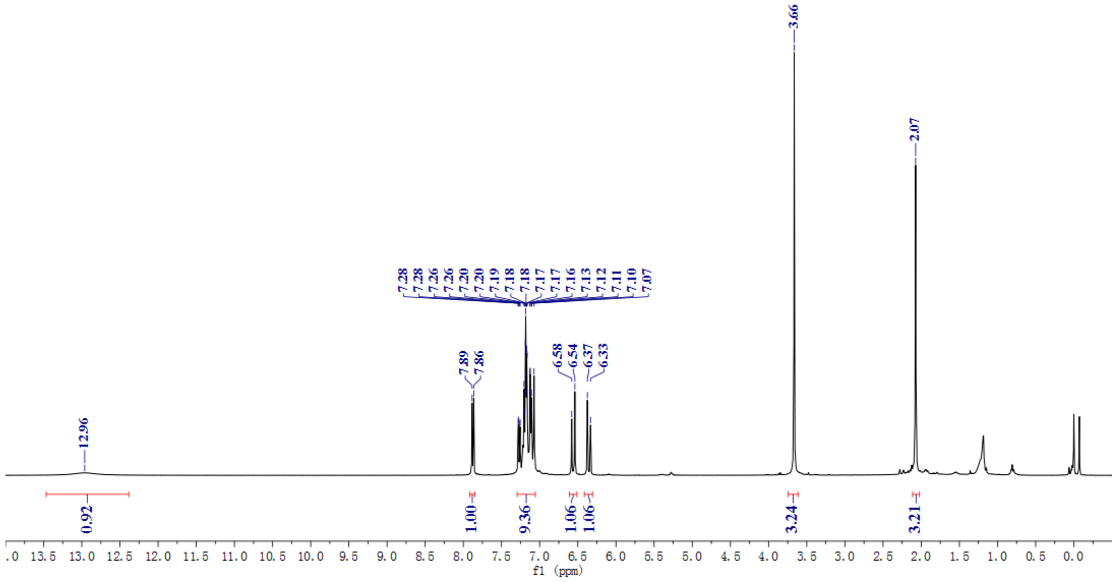

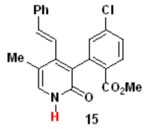

15

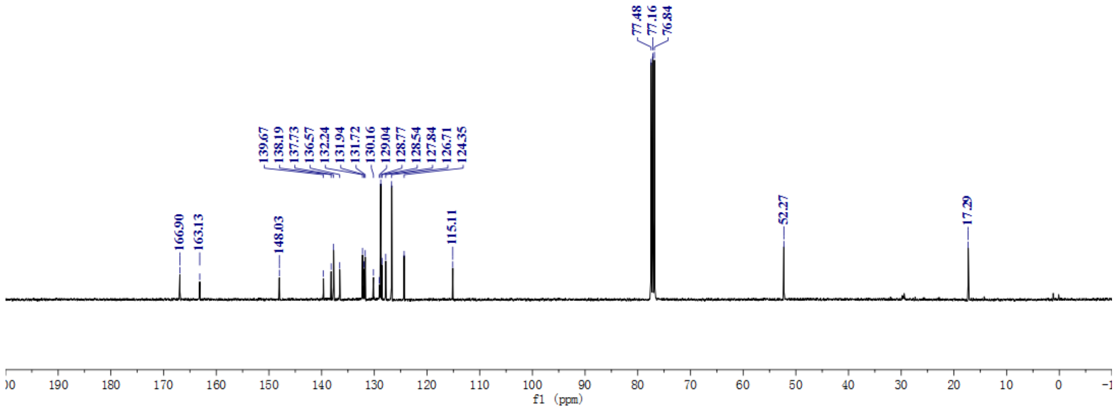

Supplementary Figure 101. NMR of **15**

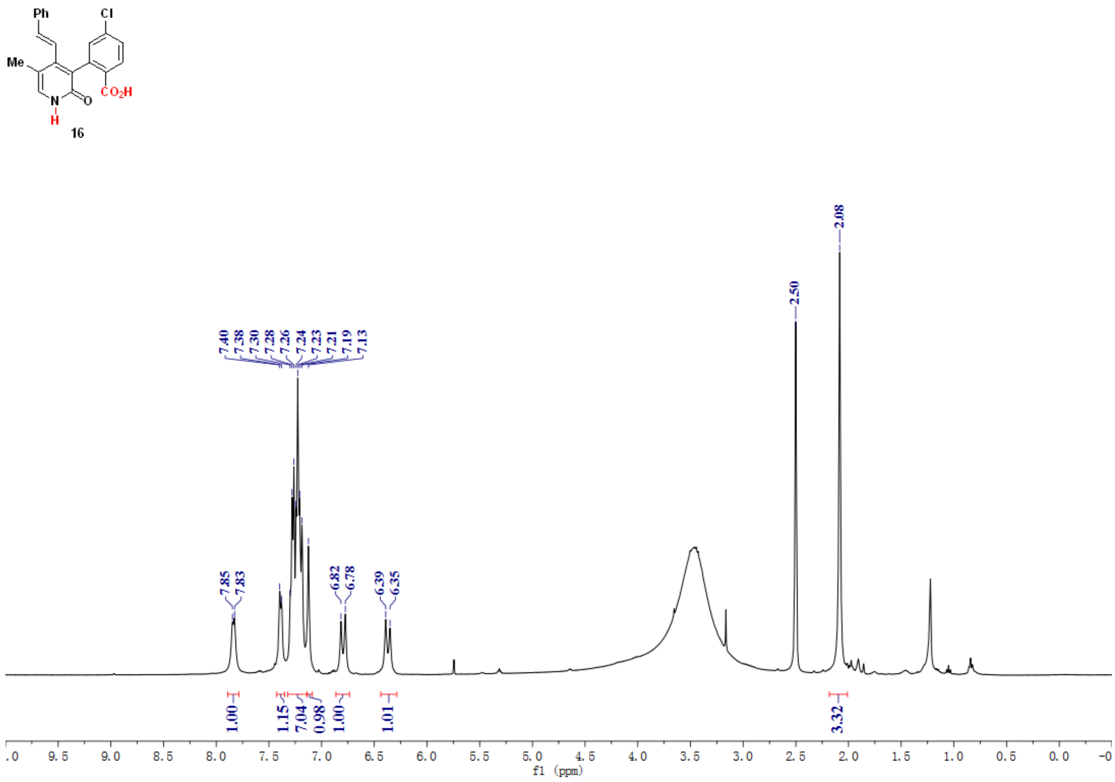

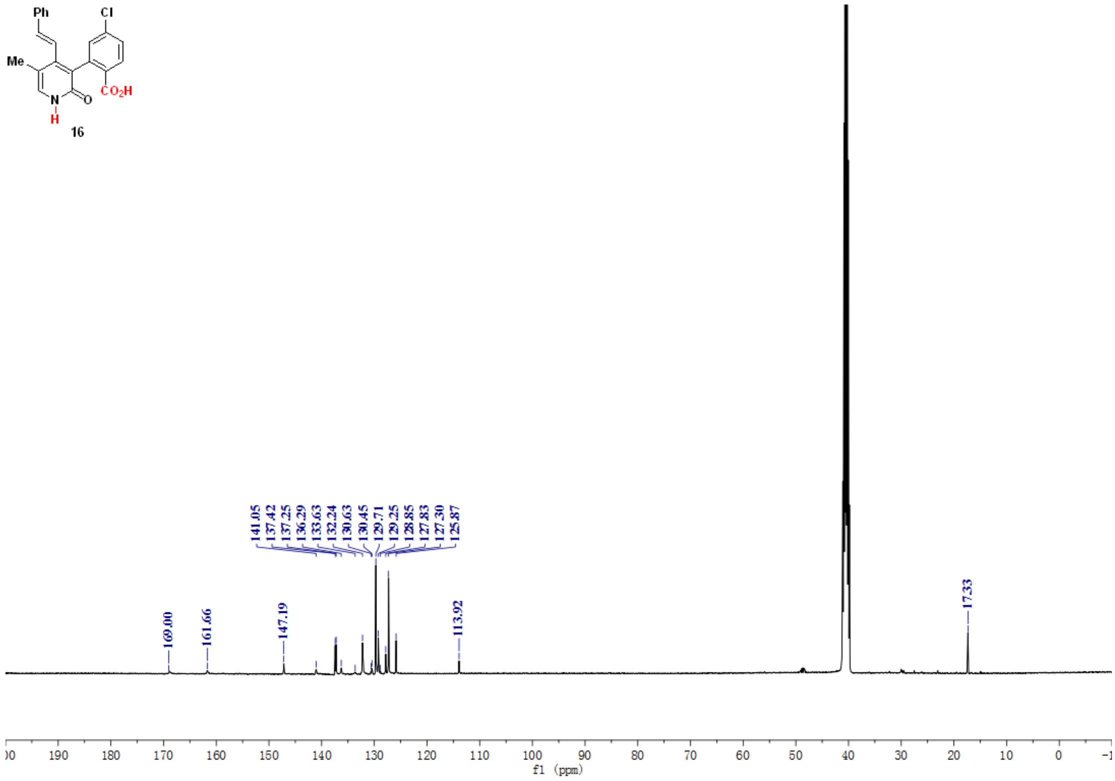

Supplementary Figure 102. NMR of **16**

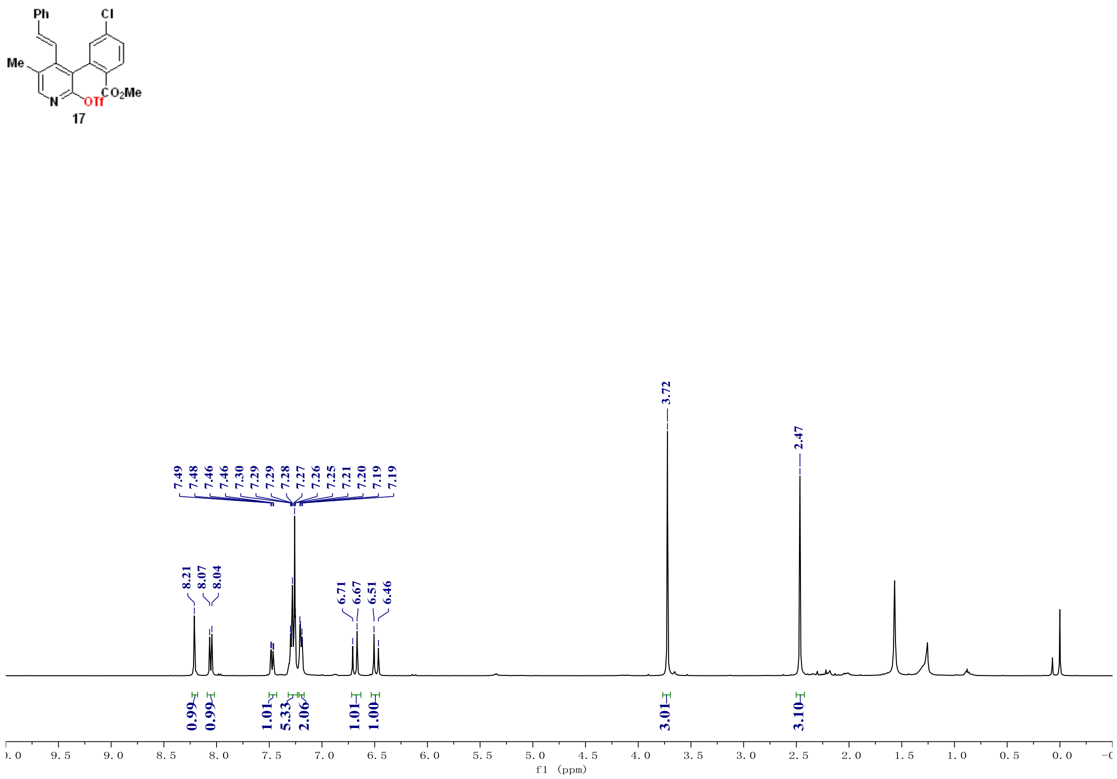

Ph
Cl
Me
N
OTf
CO2Me
17
8.21
8.07
8.04
7.49
7.48
7.46
7.46
7.30
7.29
7.29
7.28
7.27
7.26
7.25
7.21
7.20
7.19
7.19
6.71
6.67
6.51
6.46
3.72
2.47
0.99
0.99
1.01
5.33
2.06
1.01
1.00
3.01
3.10
f1 (ppm)

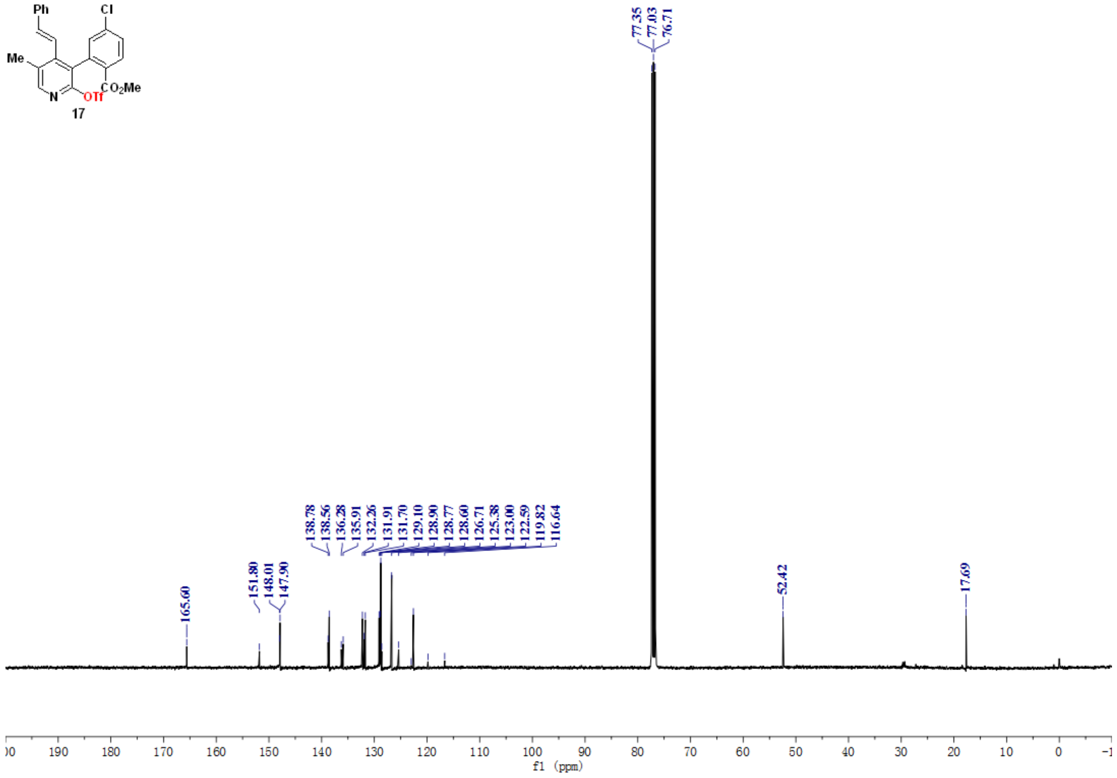

Ph
Cl
Me
N
OTf
CO2Me
17
165.60
151.80
148.01
147.90
138.78
138.56
136.28
135.91
132.26
131.91
131.70
129.10
128.90
128.77
128.60
126.71
125.38
123.00
122.59
119.82
116.64
77.35
77.03
76.71
52.42
17.69
f1 (ppm)

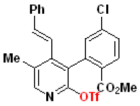

17

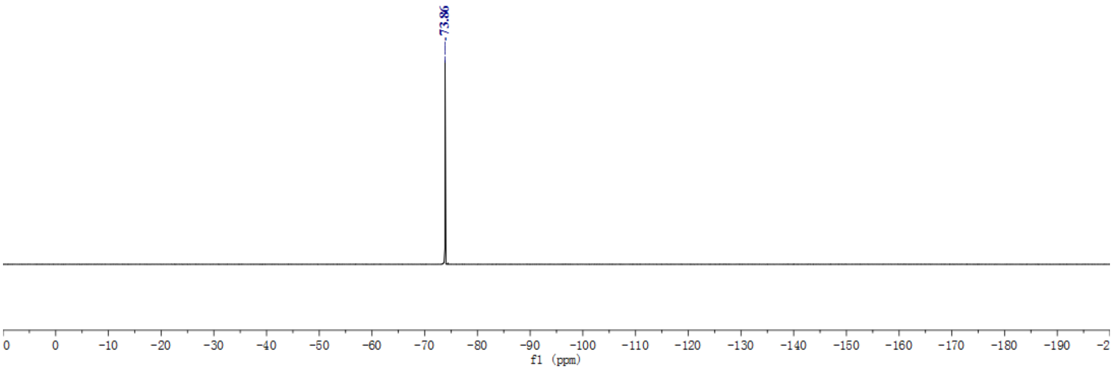

Supplementary Figure 103. NMR of **17**

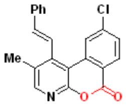

18

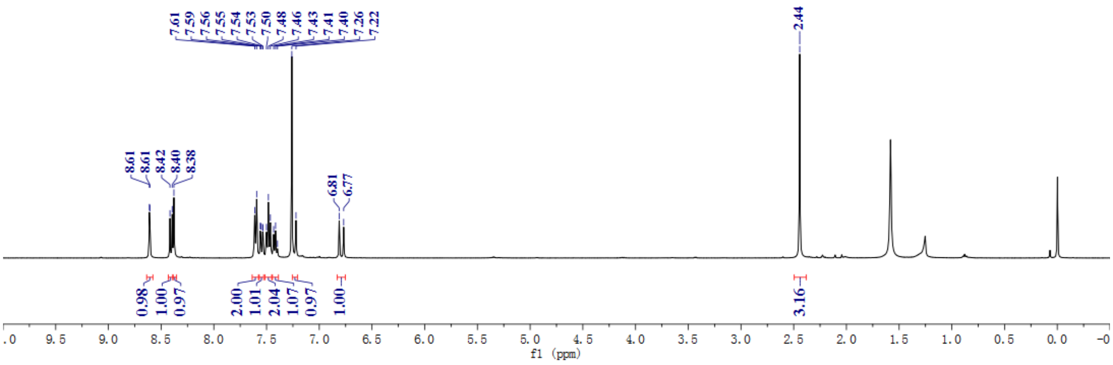

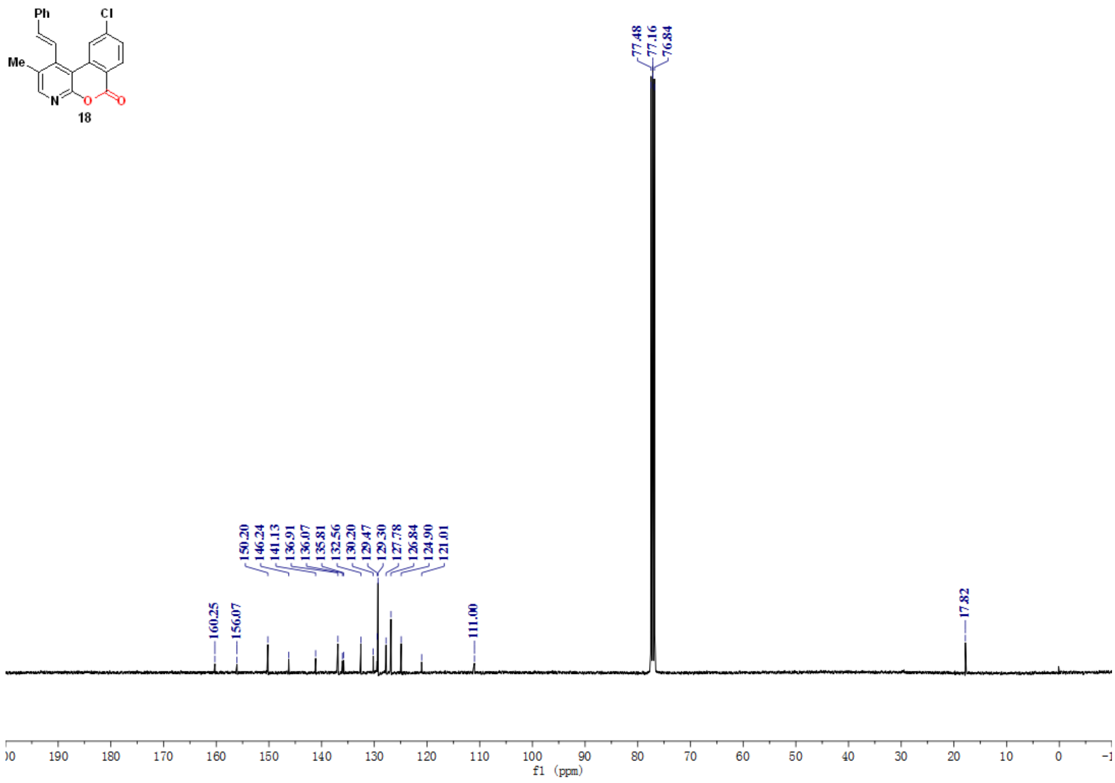

Supplementary Figure 104. NMR of **18**

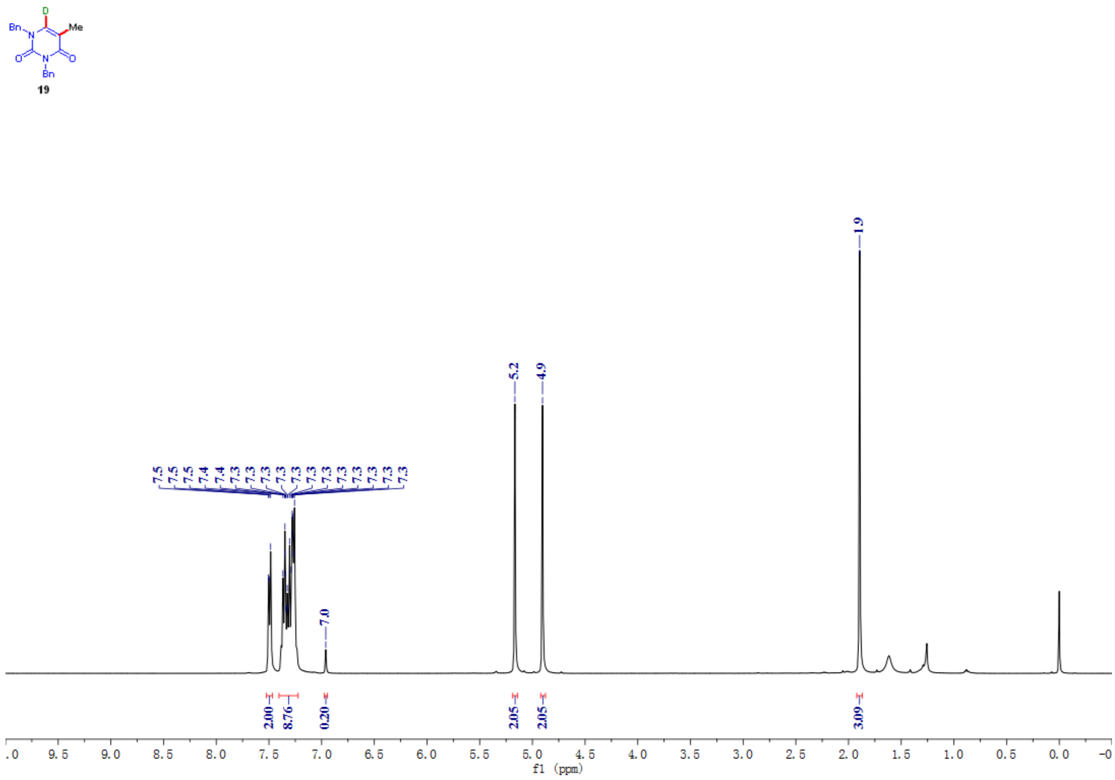

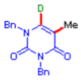

19

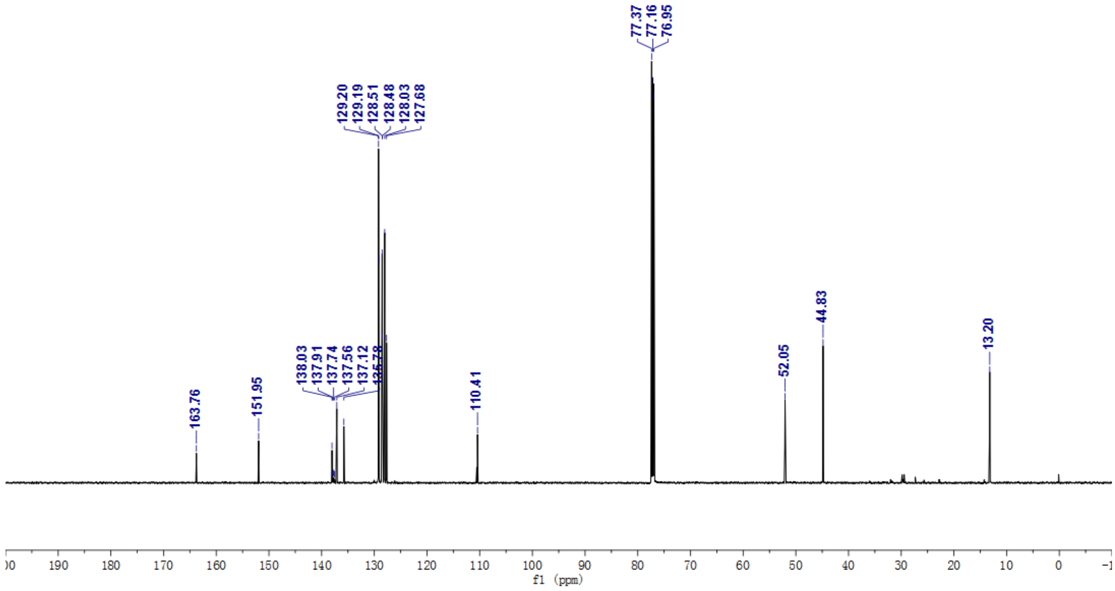

Supplementary Figure 105. NMR of **19**

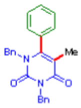

20

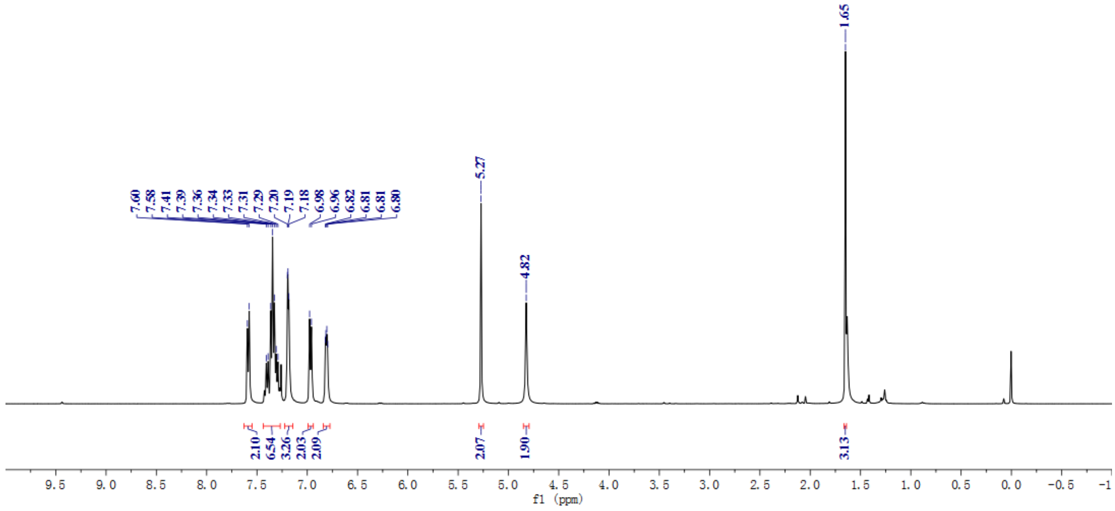

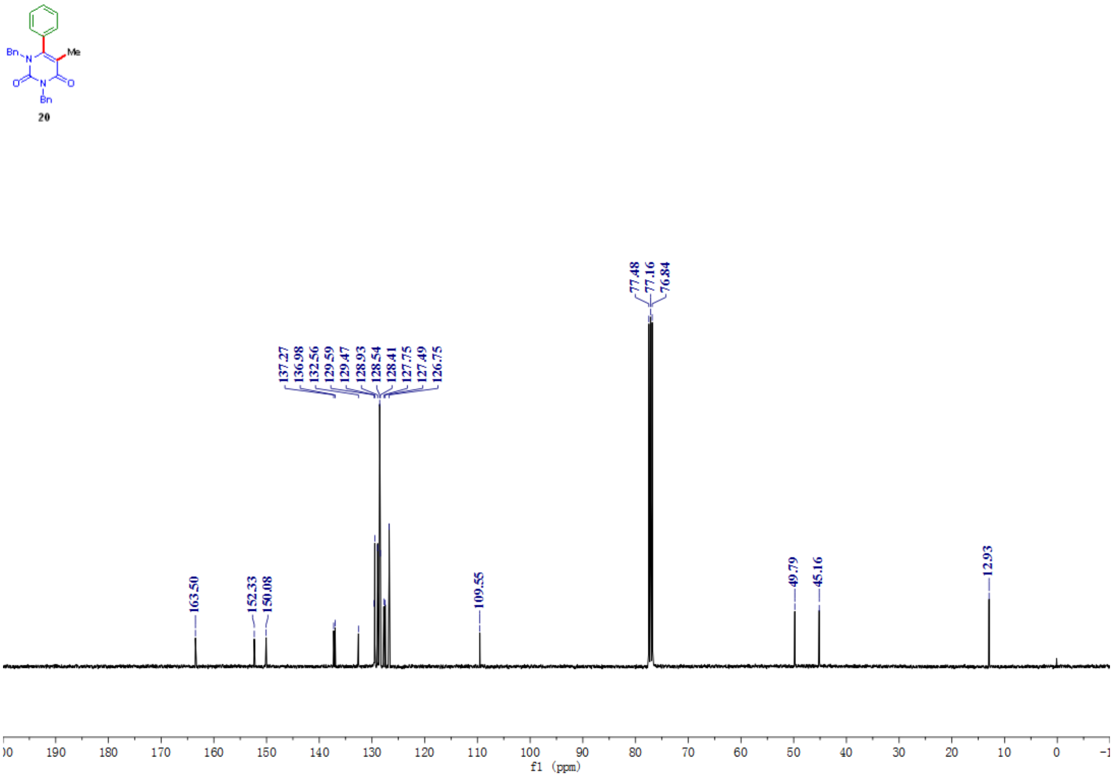

Supplementary Figure 106. NMR of **20**

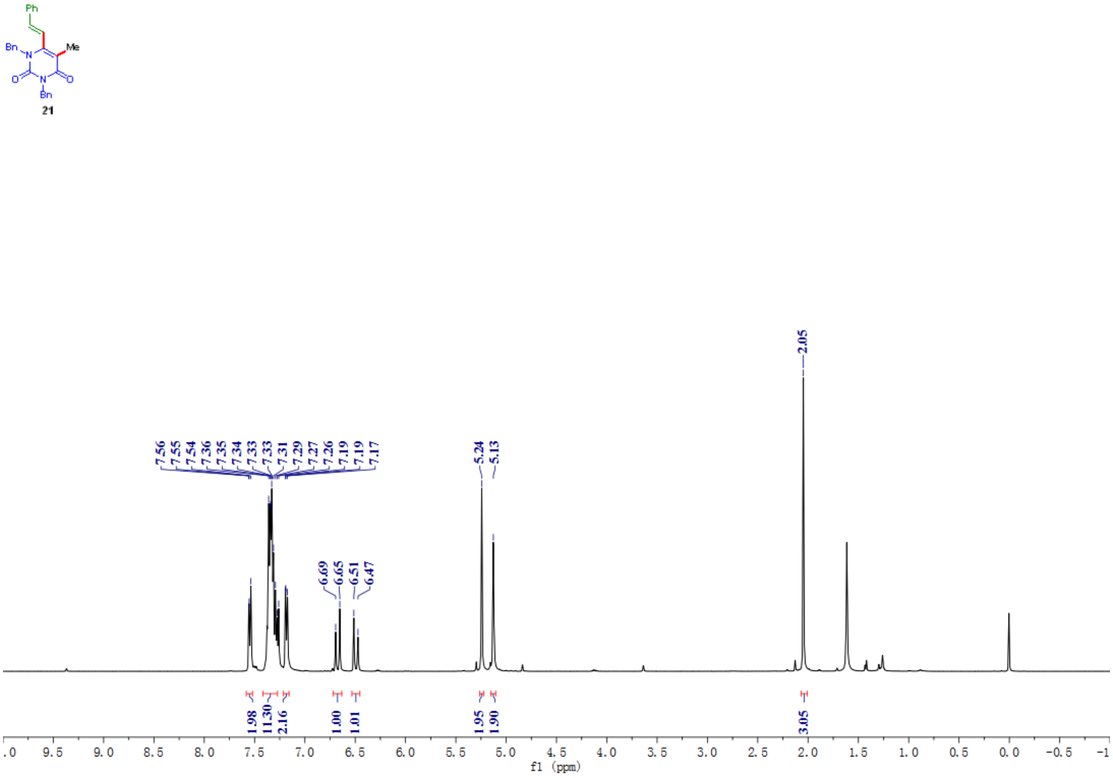

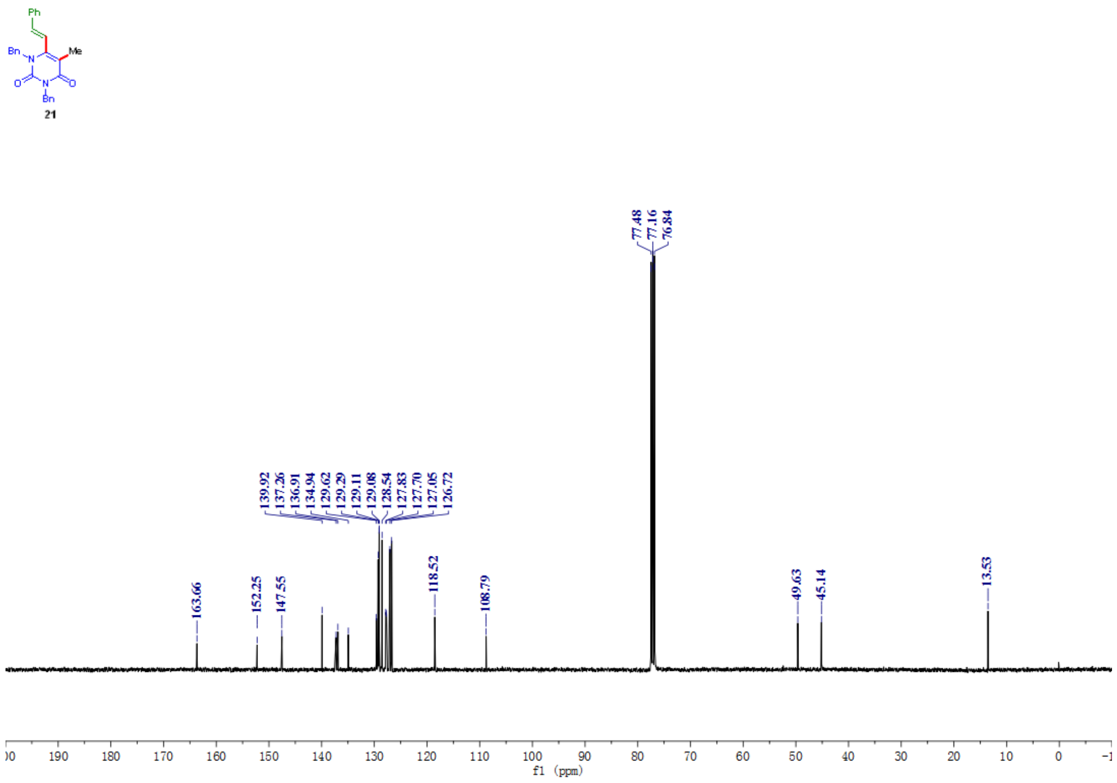

Supplementary Figure 107. NMR of **21**

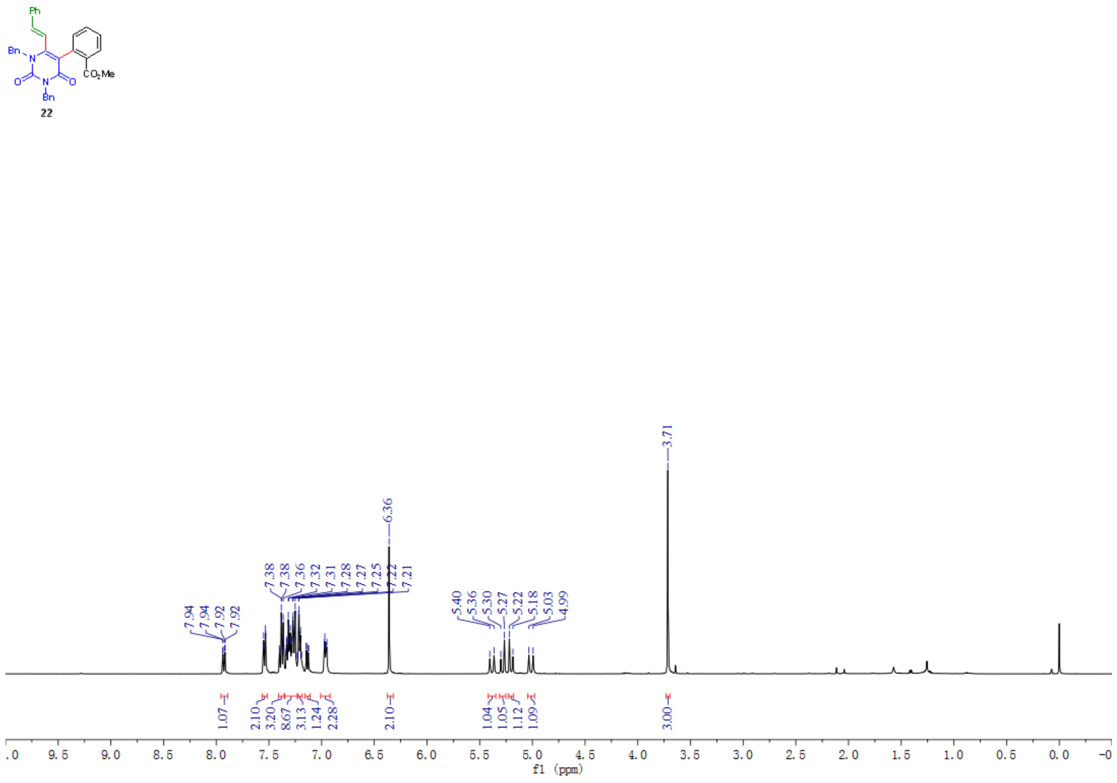

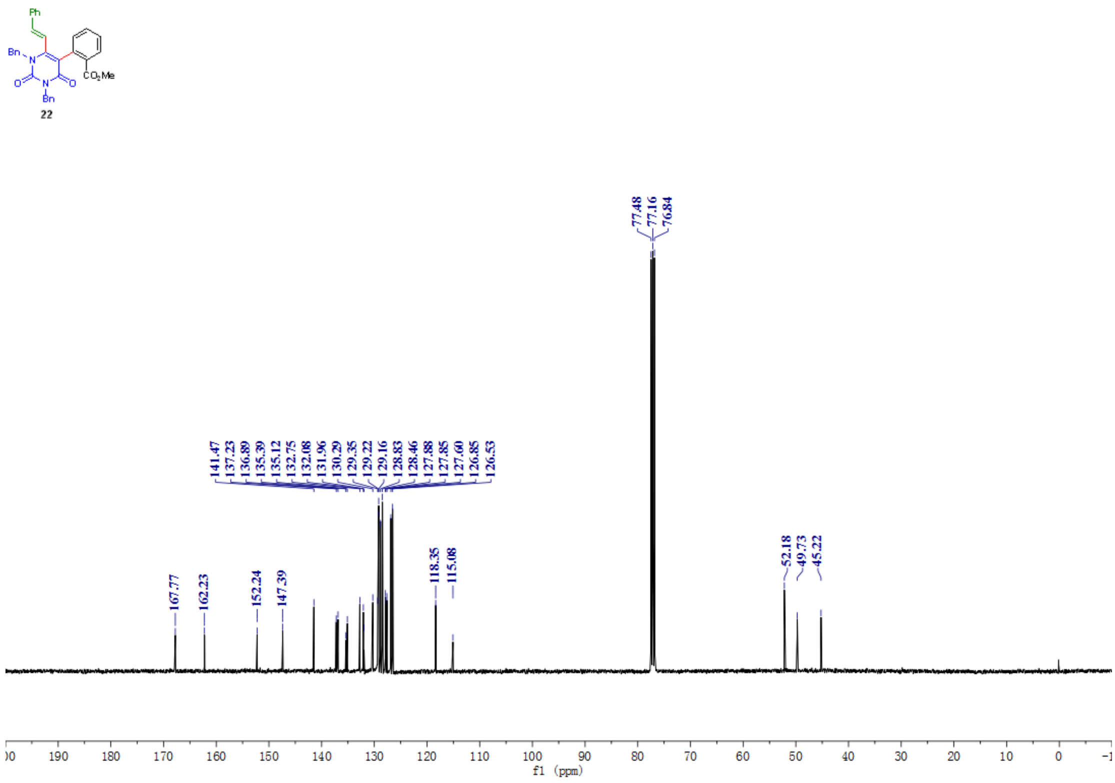

Supplementary Figure 108. NMR of **22**

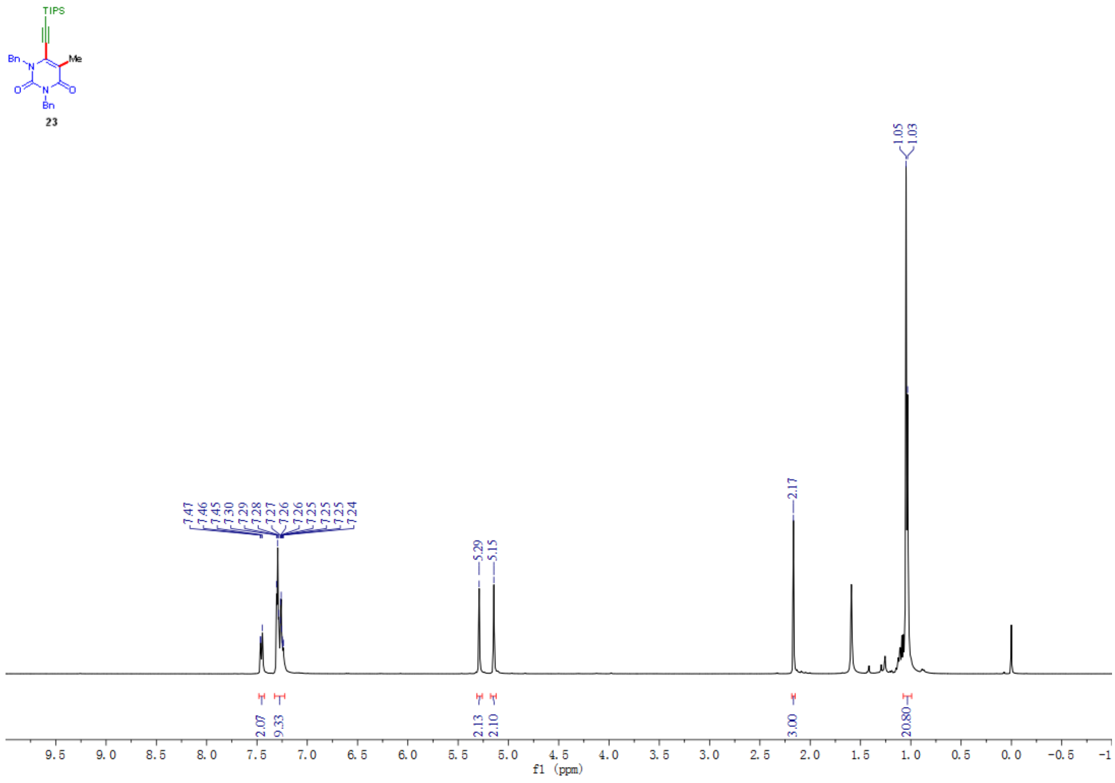

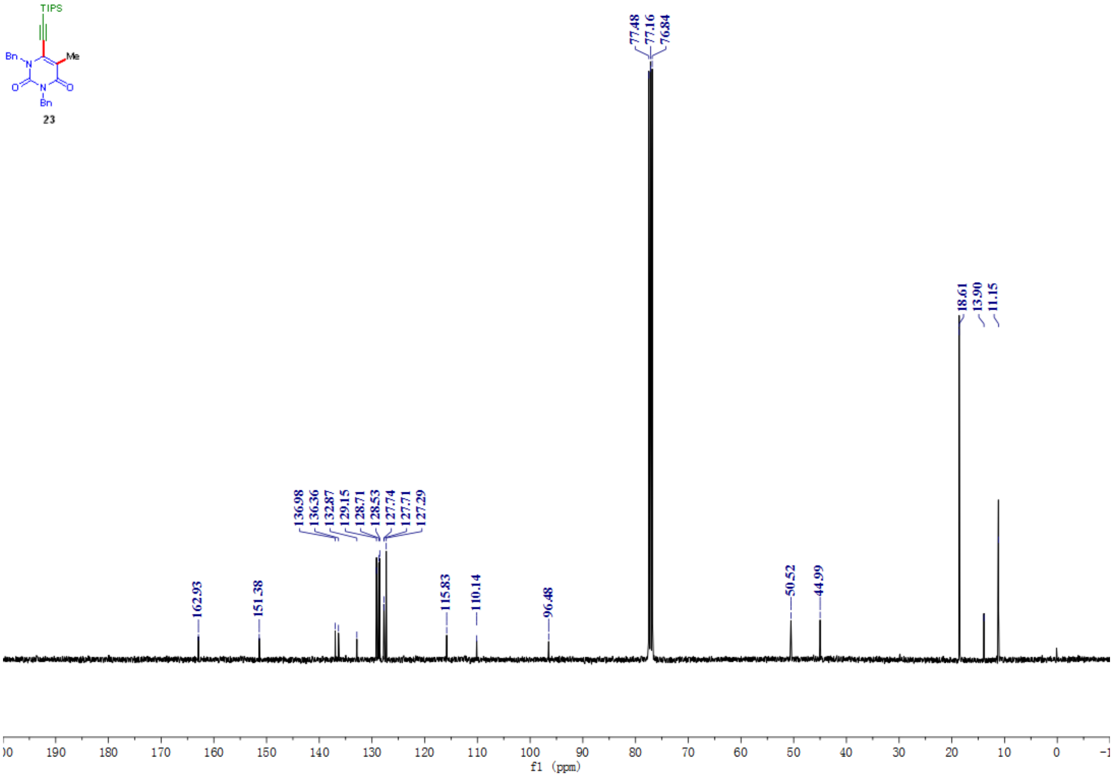

Supplementary Figure 109. NMR of **23**

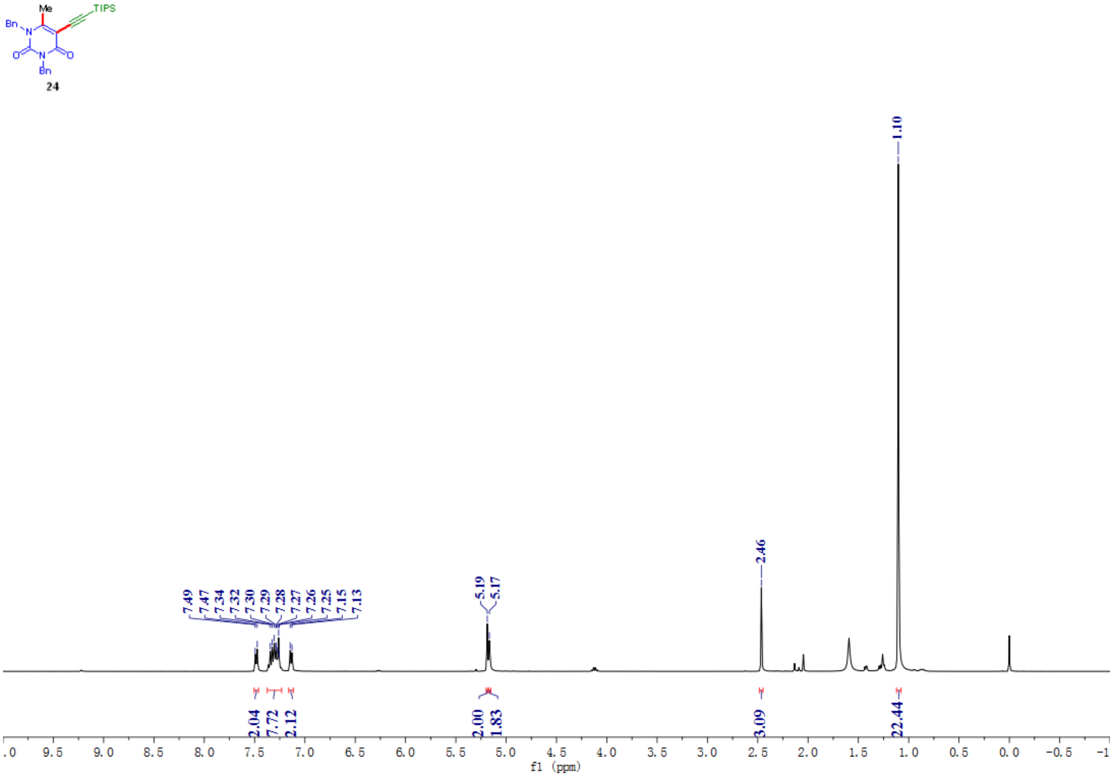

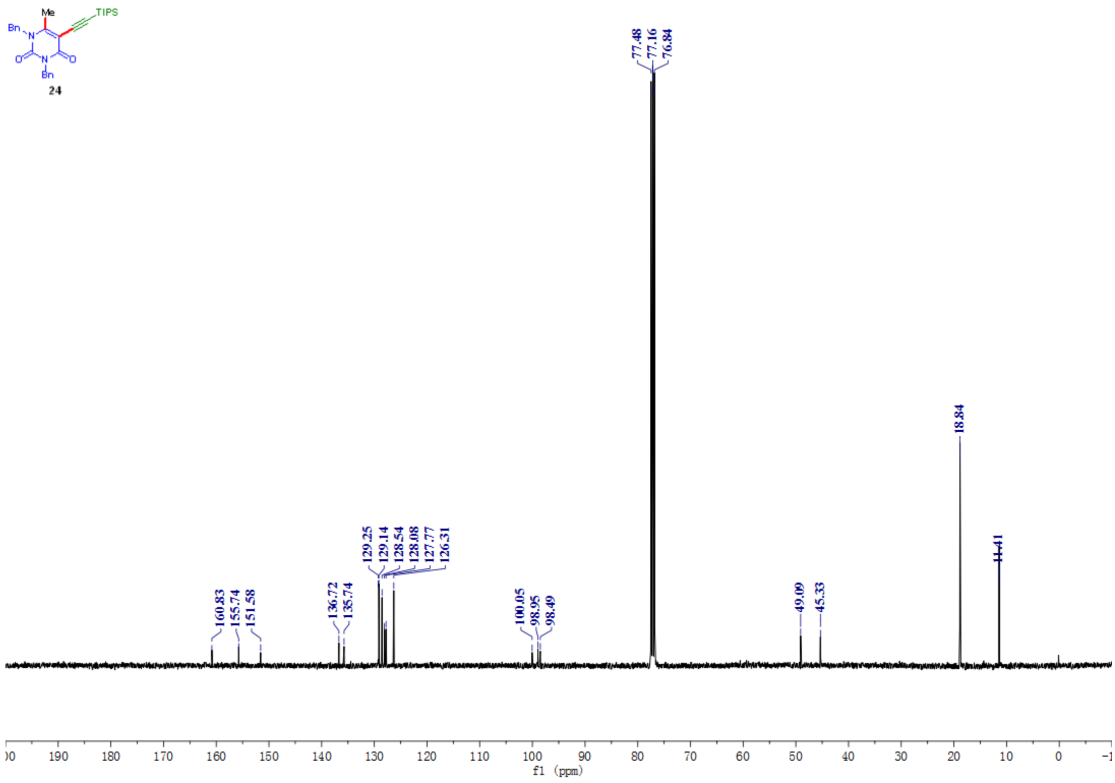

Supplementary Figure 110. NMR of **24**

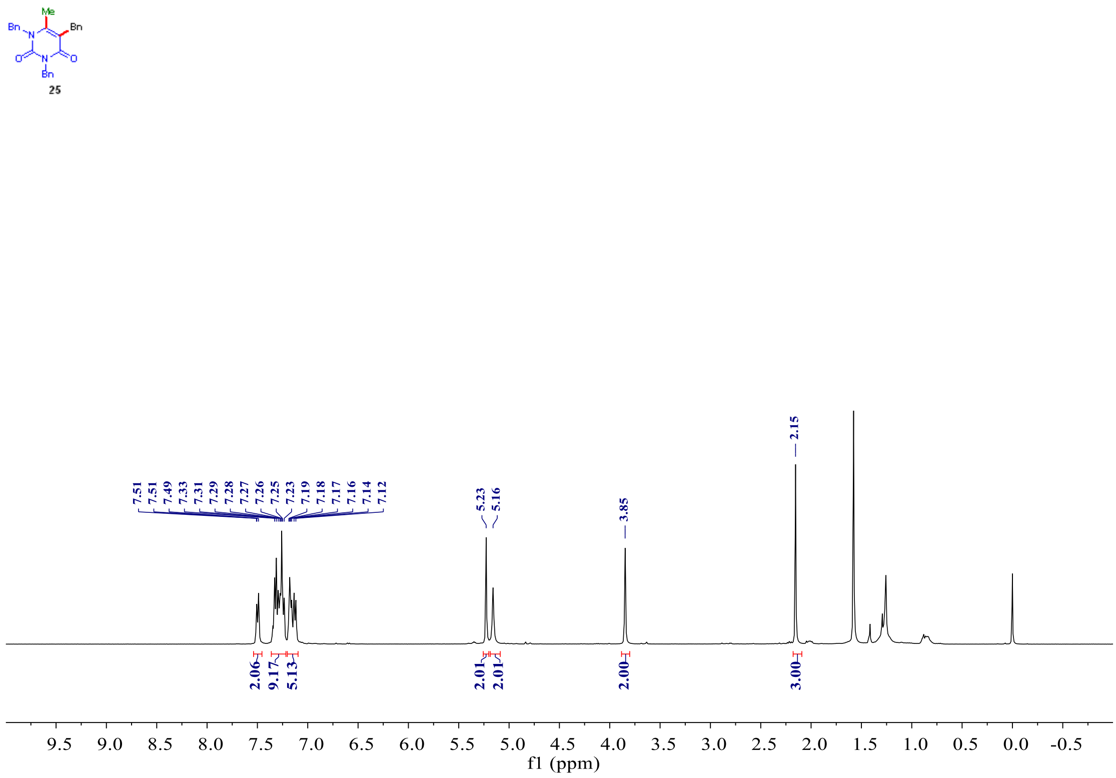

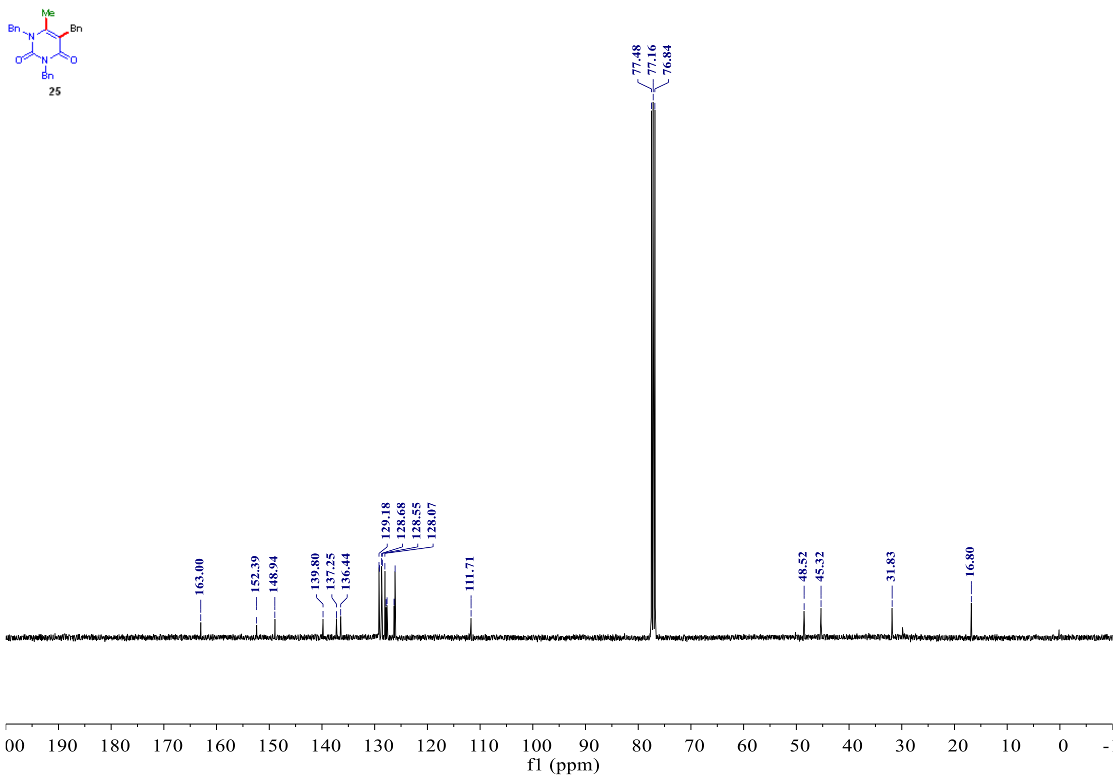

Supplementary Figure 111. NMR of **25**

2. Supplementary Tables

Supplementary Table 1 Screening of solvents for *ortho*-akylation

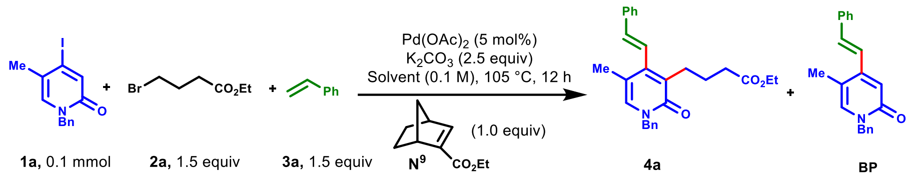

Entry	Solvent	Yield [%][a]		
		**1a**	**4a**	**BP**
1	DME	40	35	5
2	MeCN	80	14	2
3	THF	60	25	5
4	dioxane	35	40	5
5	MTBE	82	9	2
**6**[b]	**dioxane**	**trace**	**66**	**6**

[a] The reaction was performed on a 0.1 mmol scale. ^{1}H NMR yield with 1,3,5-trimethoxy-benzene as an internal standard. [b] 24 h.

Supplementary Table 2 Screening of temperature for *ortho*-akylation

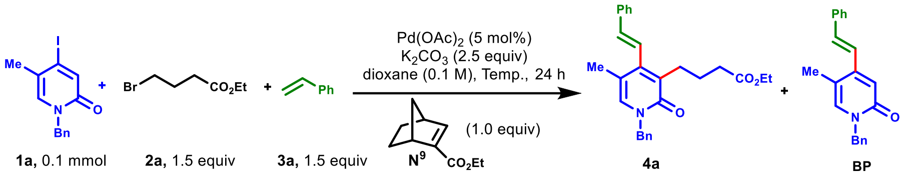

Entry	Temp. [°C]	Yield [%][a]	
		**4a**	**BP**
1	110	67	5
**2**	**105**	**68**	**4**
3	100	60	7
4	90	45	5

[a] The reaction was performed on a 0.1 mmol scale. ^{1}H NMR yield with 1,3,5-trimethoxy-benzene as an internal standard.

Supplementary Table 3 Screening of ligands for *ortho*-akylation

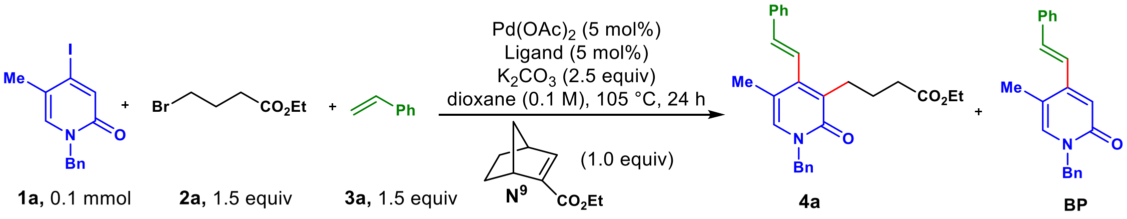

Entry	Ligand	Yield [%][a]		
		**1a**	**4a**	**BP**
**1**	**none**	**trace**	**65**	**7**
2	PPh_3	trace	60	15
3	TFP	trace	65	10
4	XPhos	trace	57	10
5	SPhos	trace	62	15
6	DavePhos	50	27	10
7	JohnPhos	40	29	15
8	$AsPh_3$	20	50	15
9[b]	DPPM	trace	57	5
10[b]	DPPP	trace	54	10
11[b]	DPEPhos	trace	53	8

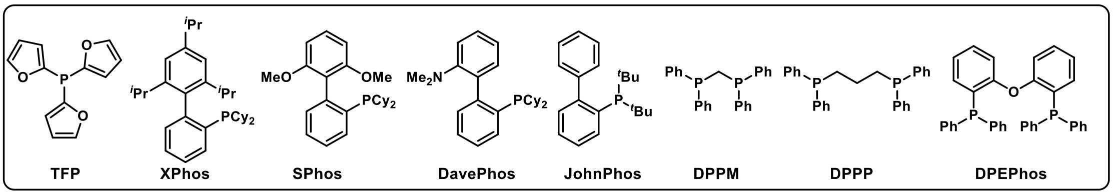

[a] The reaction was performed on a 0.1 mmol scale. ^{1}H NMR yield with 1,3,5-trimethoxy-benzene

as an internal standard. [b] With 2.5 % mol of Ligand

Supplementary Table 4 Screening of norbornene mediators[a] for *ortho*-akylation

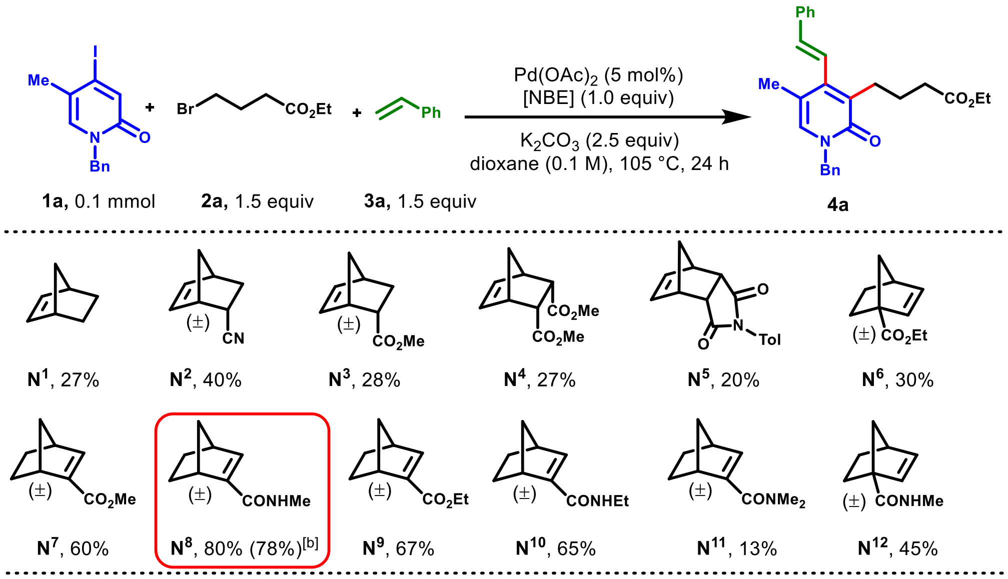

[a] The reaction was performed on a 0.1 mmol scale. ^{1}H NMR yield with 1,3,5-trimethoxy-benzene as an internal standard. [b] 0.5 equiv of **N**^{8} was applied.

Supplementary Table 5 Screening of reaction concentration, substrate molar ratio and reaction time for *ortho*-akylation

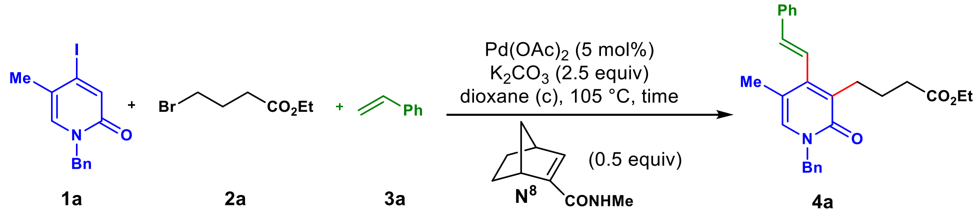

Entry	c [M]	**1a**: **2a**: **3a**	Time [h]	Yield [%][a]
1	0.1	1: 1.5: 1.5	24	80
2	0.2	1: 1.5: 1.5	24	60
3	0.1	1: 1.5: 1	24	83
4	0.1	1.2: 1.5: 1	24	92
**5**	**0.1**	**1.2: 1.5: 1**	**36**	**98 (95)**[b]

[a] The reaction was performed on a 0.1 mmol scale. ^{1}H NMR yield with 1,3,5-trimethoxy-benzene as an internal standard. [b] Isolated yield in parentheses.

Supplementary Table 6 Screening of norbornene mediators[a] for *ortho*-arylation

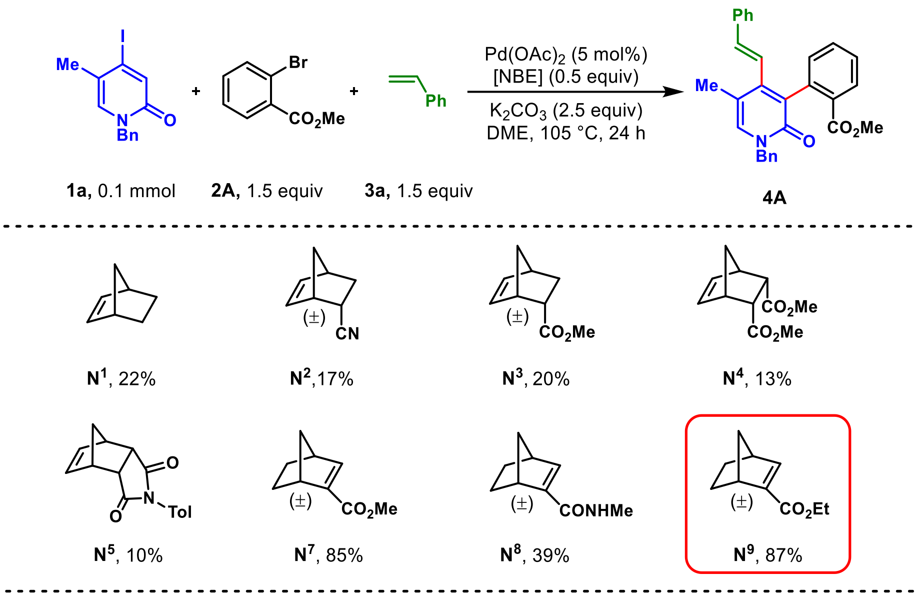

[a] The reaction was performed on a 0.1 mmol scale. ^{1}H NMR yield with 1,3,5-trimethoxy-benzene as an internal standard.

Supplementary Table 7 Screening of the loading of palladium catalyst and mediator for *ortho*-arylation

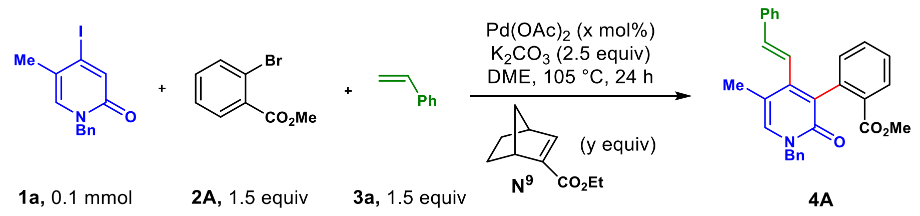

Entry	x	y	Yield [%][a]
1	5	0.5	87
2	2.5	0.5	88
**3**	**1**	**0.5**	**89 (93)**[b]
4	1	0.25	82
5	1	0.1	76

[a] The reaction was performed on a 0.1 mmol scale. ^{1}H NMR yield with 1,3,5-trimethoxy-benzene as an internal standard. [b] Isolated yield in parentheses.

Supplementary Table 8 Optimization of asymmetric reaction conditions

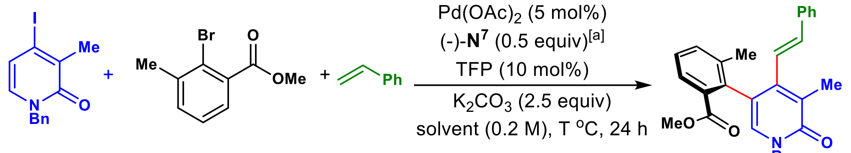

1k 0.1 mmol | **2L** 1.0 equiv | **3a** 1.5 equuv | **4A***

Entry	Solvent	T/°C	P/%[b]	Ee/%
1	THF	100	72	89
2	dioxane	100	71	85
3	MeCN	100	0	0
4	DCE	100	50	88
5	DME	100	76	86
6	toluene	100	52	85
7	DME	105	85	77
8	DME	100	76	86
9	DME	95	71	90
10	DME	95	73	93
11	DME	85	72	94
12	DME	80	53	95
**13[c]**	**DME**	**85**	**74 (73%)[d]**	**97**

[a] 99% ee of (-)-**N**[7] was used. [b] ^{1}H NMR yield,1,3,5-trimethoxybenzene as internal standard. [c] 99% ee of (-)-**N**[9] was used. [d] Isolated yield.

3. Supplementary Methods

All reactions dealing with air- or moisture-sensitive compound were performed by standard Schlenk techniques in oven-dried reaction vessels under argon atmosphere or in the argon-filled glove box. Unless otherwise noted, all solvents were dried by JC Meyer Solvent Drying System. Most reagents were purchased from commercial sources and used without further purification, unless otherwise stated. Reactions were monitored by thin layer chromatography (TLC) carried out on 0.2 mm commercial silica gel plates, using UV light as the visualizing agent or basic solution of $KMnO_4$ or acidic solution of *p*-anisaldehyde and heat as a developing agent. All NMR spectra were recorded on a Bruker spectrometer at 400 MHz (^{1}H NMR), 100 MHz (^{13}C NMR), 376 MHz (^{19}F NMR), 162 MHz (^{31}P NMR) and were calibrated using residual undeuterated solvent as an internal reference ($CDCl_3$ @ 7.26 ppm ^{1}H NMR, 77.16 ppm ^{13}C NMR). The following abbreviations were used to explain multiplicities: s = singlet, d = doublet, t = triplet, q = quartet, dd = doublet of doublets, dt = doublet of triplets, td = triplet of

doublets, ddd = doublet of doublet of doublets, m = multiplet, br = broad. High resolution mass spectra (HRMS) were recorded on DIONEX UltiMate 3000 & Bruker Compact TOF mass spectrometer. Enantiomeric excess (*ee*) was determined by chiral HPLC (Agilent 1260) with ^{n}hexane and ^{i}PrOH as solvents.

4. Supplementary Notes

Supplementary Note 1: Preparation of 1e, 1m, 1n.

Substrates **1e**[4], **1m**[9] and **1n**[10] were known compounds and synthesized following the reported procedure.

Supplementary Note 2: Synthesis of 4-iodo-2-pyridones **1a-h** and **1k-l**[1-5].

To the solution of **S1** (5.0 mmol, 1.0 equiv) in 1,4-dioxane:water (1:1, 5 mL) was added concentrated HCl (2 mL). The reaction mixture was heated to 100 ^{o}C and was stirred at this temperature for 2 h. Then, the reaction mixture was allowed to cool to room temperature and was diluted with water (6 mL) causing a precipitate to form. The suspension was stirred overnight at room temperature, and the solid was collected via filtration. The solid was washed with water and dried *in vacuum* to afford **S2**.

To the solution of **S2** (2.5 mmol, 1.0 equiv) in acetone (25 mL) was added anhydrous potassium carbonate (10.0 mmol) and the corresponding alkyl halides (3.75 mmol, 1.5 equiv) in a round bottom flask. The reaction mixture was allowed to stir at room temperature for 12 h (in case of 2,4,6-trimethyl benzyl chloride, the reaction mixture was refluxed for 12 h). After full consumption of the starting materials (monitored by TLC), acetone was removed *in vacuum* and the residue was dissolved in water. After that, it was extracted with ethyl acetate 3 times (50 mL×3). The organic layer was dried over anhydrous sodium sulfate and concentrated *in vacuum*. The products ware purified by flash silica gel column chromatography to afford **1a-h** and **1k-l**.

1-Benzyl-4-iodo-5-methylpyridin-2(1*H*)-one (**1a**)

Physical state: white solid;

Melting point: 110–115 ºC;

Yield: 67%;

R_f = 0.4 (silica gel, PE: EtOAc = 2:1);

^{1}H NMR (400 MHz, $CDCl_3$): δ 7.38 – 7.26 (m, 6H), 7.01 (s, 1H), 5.07 (s, 2H), 2.10 (s, 3H).

^{13}C NMR (100 MHz, $CDCl_3$): δ 160.5, 136.2, 132.6, 131.0, 129.1, 128.3, 128.3, 118.9, 117.6, 51.8, 23.5.

HRMS (ESI-TOF): calc'd for $C_{13}H_{12}INNaO^+$ [M+Na$^+$] 347.9856, found 347.9853.

4-Iodo-1,5-dimethylpyridin-2(1*H*)-one (**1b**)

Physical state: yellow solid;

Melting point: 70–75 ºC;

Yield: 75%;

R_f = 0.2 (silica gel, PE: EtOAc = 1:1);

^{1}H NMR (400 MHz, $CDCl_3$): δ 7.26 (s, 1H), 7.06 (s, 1H), 3.48 (s, 3H), 2.15 (s, 3H).

^{13}C NMR (100 MHz, $CDCl_3$): δ 160.9, 133.8, 130.5, 118.6, 117.3, 37.4, 23.3.

HRMS (ESI-TOF): calc'd for $C_7H_8INNaO^+$ [M+Na$^+$] 271.9543, found 271.9547.

4-Iodo-5-methyl-1-(2,4,6-trimethylbenzyl)pyridin-2(1*H*)-one (**1c**)

Physical state: yellow solid;

Melting point: 110–115 ºC;

Yield: 62%;

R_f = 0.3 (silica gel, PE: EtOAc = 2:1);

^{1}H NMR (400 MHz, $CDCl_3$): δ 7.30 (s, 1H), 6.93 (s, 2H), 6.48 (s, 1H), 5.02 (s, 2H), 2.31 (s, 3H), 2.19 (s, 6H), 2.00 (s, 3H).

^{13}C NMR (100 MHz, $CDCl_3$): δ 160.9, 138.9, 138.6, 130.0, 129.8, 127.6, 118.7, 117.4, 45.4, 23.7, 21.2, 19.8.

HRMS (ESI-TOF): calc'd for $C_{16}H_{18}INNaO^+$ [M+Na$^+$] 390.0325, found 390.0329.

4-Iodo-5-methylpyridin-2(1*H*)-one (**1e**)

Physical state: brown solid;

Melting point: 160–165 ºC;

Yield: 93%;

R_f = 0.2 (silica gel, PE: EtOAc = 1:1);

^{1}H NMR (400 MHz, DMSO-*d6*): δ 7.46 (s, 1H), 7.22 (s, 1H), 2.11 (s, 3H).

^{13}C NMR (100 MHz, DMSO-*d6*): δ 159.7, 132.1, 127.8, 121.6, 119.9, 22.4.

HRMS (ESI-TOF): calc'd for $C_6H_6INNaO^+$ [M+Na$^+$] 257.9386, found 257.9383.

Both the proton and carbon NMR match the literature reported data.[2]

1-Benzyl-5-fluoro-4-iodopyridin-2(1*H*)-one (**1f**)

Physical state: white solid;

Melting point: 130–135 ºC;

Yield: 64%;

R_f = 0.3 (silica gel, PE: EtOAc = 2:1);

^{1}H NMR (400 MHz, CDCl$_3$): δ 7.41 – 7.27 (m, 5H), 7.21 (d, *J* = 5.9 Hz, 1H), 7.08 (d, *J* = 3.5 Hz, 1H), 5.05 (s, 2H).

^{13}C NMR (100 MHz, CDCl$_3$): δ 159.4, 147.1 (d, *J* = 230.7 Hz), 135.3, 130.9, 129.3, 128.7, 128.6, 120.7 (d, *J* = 40.0 Hz), 101.8 (d, *J* = 27.5 Hz), 52.2.

^{19}F NMR (376 MHz, CDCl3): δ -132.0.

HRMS (ESI-TOF): calc'd for $C_{12}H_9FINNaO^+$ [M+Na$^+$] 351.9605, found 351.9609.

5-Fluoro-4-iodo-1-(4-methoxybenzyl)pyridin-2(1*H*)-one (**1g**)

Physical state: yellow solid;

Melting point: 110–115 ºC;

Yield: 56%;

R_f = 0.3 (silica gel, PE: EtOAc = 2:1);

^{1}H NMR (400 MHz, CDCl$_3$): δ 7.26 – 7.24 (m, 2H), 7.19 (d, *J* = 6.0 Hz, 1H), 7.07 (d, *J* = 3.6 Hz, 1H), 6.89 (d, *J* = 8.6 Hz, 2H), 4.98 (s, 2H), 3.81 (s, 3H).

^{13}C NMR (100 MHz, CDCl$_3$): δ 160.0, 159.4, 147.1 (d, *J* = 230.4 Hz), 130.8, 130.2,

127.2, 120.5 (d, J = 40.0 Hz), 114. 7, 101.7 (d, J = 27.0 Hz), 55.5, 51.7.

^{19}F NMR (376 MHz, CDCl3): δ -132.0.

HRMS (ESI-TOF): calc'd for $C_{13}H_{11}FINNaO_2^+$ [M+Na$^+$] 381.9711, found 381.9708.

1-Benzyl-5-chloro-4-iodopyridin-2(1*H*)-one (**1h**)

Physical state: white solid;

Melting point: 95–100 ºC;

Yield: 72%;

R_f = 0.3 (silica gel, PE: EtOAc = 5:1);

^{1}H NMR (400 MHz, $CDCl_3$): δ 7.39 – 7.36 (m, 3), 7.34 – 7.27 (m, 4H), 5.06 (s, 2H).

^{13}C NMR (100 MHz, $CDCl_3$): δ 159.5, 135.3, 133.0, 131.9, 129.3, 128.7, 128.5, 117.4, 114.4, 52.2.

HRMS (ESI-TOF): calc'd for $C_{12}H_9ClINNaO^+$ [M+Na$^+$] 367.9310, found 367.9314.

1-Benzyl-4-iodo-3-methylpyridin-2(1*H*)-one (**1k**)

Physical state: white solid;

Melting point: 100–105 ºC;

Yield: 76%;

R_f = 0.3 (silica gel, PE: EtOAc = 2:1);

^{1}H NMR (400 MHz, $CDCl_3$): δ 7.37 – 7.27 (m, 5H), 6.83 (d, J = 7.2 Hz, 1H), 6.56 (d, J = 7.2 Hz, 1H), 5.08 (s, 2H), 2.37 (s, 3H).

^{13}C NMR (100 MHz, $CDCl_3$): δ 159.9, 136.1, 135.1, 133.5, 129.1, 128.3 (2C), 116.4, 111.8, 52.5, 22.9.

HRMS (ESI-TOF): calc'd for $C_{13}H_{12}INNaO^+$ [M+Na$^+$] 347.9856, found 347.9859.

1-Benzyl-4-iodopyridin-2(1*H*)-one (**1l**)

Physical state: white solid;

Melting point: 90–95 ºC;

Yield: 68%;

R_f = 0.3 (silica gel, PE: EtOAc = 5:1);

^{1}H NMR (400 MHz, $CDCl_3$): δ 7.39 – 7.26 (m, 5H), 7.14 (s, 1H), 6.93 (d, *J* = 7.1 Hz, 1H), 6.47 – 6.45 (m, 1H), 5.07 (s, 2H).

^{13}C NMR (100 MHz, $CDCl_3$): δ 161.1, 136.6, 135.9, 130.5, 129.2, 128.4, 128.4, 115.7, 108.9, 51.9.

HRMS (ESI-TOF): calc'd for $C_{12}H_{10}INNaO^+$ [M+Na$^+$] 333.9699, found 333.9702.

Supplementary Note 3: 4-Iodo-1-(methoxymethyl)-5-methylpyridin-2(1*H*)-one (**1d**)[5].

To the solution of 4-iodo-5-methylpyridin-2(1*H*)-one (2.5 mmol, 1.0 equiv) in THF (5 mL) was added NaH (3.0 mmol, 1.2 equiv) slowly at 0 °C. The mixture was stirred for 1 h, treated with MOMCl (3.75 mmol, 1.5 equiv) and stirred for 2 h at 0 °C. Then the solution was warmed to room temperature, after full consumption of the starting materials (monitored by TLC), THF was removed *in vacuum* and the residue was dissolved in water. After that, it was extracted with ethyl acetate 3 times (50 mL×3). The organic layer was dried over anhydrous sodium sulfate and concentrated *in vacuum*. The products ware purified by flash silica gel column chromatography to afford **1d**.

Physical state: yellow solid;

Melting point: 100–105 °C;

Yield: 70%;

$\boldsymbol{R_f}$ = 0.3 (silica gel, PE: EtOAc = 2:1);

^{1}H NMR (400 MHz, $CDCl_3$): δ 7.27 (s, 1H), 7.12 (s, 1H), 5.24 (s, 2H), 3.38 (s, 3H), 2.17 (s, 3H).

^{13}C NMR (100 MHz, $CDCl_3$): δ 160.7, 131.3, 131. 2, 119.0, 118.7, 78.1, 57.3, 23.5.

HRMS (ESI-TOF): calc'd for $C_8H_{11}INO_2^+$ [M+H$^+$] 279.9829, found 279.9834.

Supplementary Note 4: Synthesis of 1-benzyl-4-iodo-5-(methoxymethyl)pyridin-2(1*H*)-one (**1i**)[1,2, 6,7]

To the solution of (6-fluoro-4-iodopyridin-3-yl)methanol[6, 7] (506 mg, 2.0 mmol, 1.0 equiv) in 1,4-dioxane: water (1:1, 5 mL) was added concentrated HCl (1 mL). The reaction mixture was heated to 100 °C and stirred at this temperature for 2 h. The

reaction mixture was removed out oil bath and MeOH (10 mL) was added quikly and stirred for another 0.5 h. The solvent was removed *in vacuum* to afford the crude product 4-iodo-5-(methoxymethyl)pyridin-2(1*H*)-one.

To the solution of the above crude product 4-iodo-5-(methoxymethyl)pyridin-2(1*H*)-one in acetone (25 mL) was added anhydrous potassium carbonate (1.105 g, 8.0 mmol, 4.0 equiv) and benzyl bromide (513 mg, 3.0 mmol, 1.5 equiv) in a round bottom flask. Then the reaction mixture was allowed to stir at room temperature for 12 h. After full consumption of the starting materials (monitored by TLC), acetone was removed *in vacuum* and the residue was dissolved in water. After that, it was extracted with ethyl acetate 3 times (50 mL ×3). The combined organic layers were washed with brine, dried over Na_2SO_4, filtered and concentrated *in vacuum*. The product was purified by flash silica gel column chromatography to afford **1i** (602 mg, 85% yield over 2 steps).

Physical state: white solid;

Melting point: 80–85 °C;

Yield: 40%;

R_f = 0.3 (silica gel, PE: EtOAc = 2:1);

^{1}H NMR (400 MHz, $CDCl_3$): δ 7.38 – 7.27 (m, 6H), 7.19 (s, 1H), 5.09 (s, 2H), 4.15 (s, 2H), 3.39 (s, 3H).

^{13}C NMR (100 MHz, $CDCl_3$): δ 160.7, 135.9, 134.9, 131.4, 129.1, 128.4, 128.3, 118.6, 113.4, 75.0, 58.5, 52.1.

HRMS (ESI-TOF): calc'd for $C_{14}H_{14}INNaO_2^+$ [M+Na$^+$] 377.9961, found 377.9964.

Supplementary Note 5: Synthesis of methyl 2-(1-benzyl-4-iodo-6-oxo-1,6-dihydropyridin-3-yl)acetate (**1j**)[1, 2, 6, 7]

To the solution of the 2-(6-fluoro-4-iodopyridin-3-yl)acetonitrile[6, 7] (514 mg, 2.0 mmol, 1.0 equiv) in 1,4-dioxane:water (1:1, 5 mL) was added concentrated HCl (1 mL). The reaction mixture was heated to 100 °C and stirred at this temperature for 2 h. Then the reaction mixture removed to atmospheres and the solvent was removed *in vacuum* to afford crude product 2-(4-iodo-6-oxo-1,6-dihydropyridin-3-yl)acetonitrile .

To the solution of the above crude product 2-(4-iodo-6-oxo-1,6-dihydropyridin-3-

yl)acetonitrile in MeOH (25 mL) was added anhydrous potassium carbonate (1.11g, 8.0 mmol, 4.0 equiv) and benzyl bromide (513 mg, 3.0 mmol, 1.5 equiv) in a round bottom flask. The reaction mixture was allowed to stir at room temperature for 12 h. After full consumption of the starting materials (monitored by TLC), acetone was removed in vacuum and the residue was dissolved in water. After that, it was extracted with ethyl acetate 3 times (50 mL ×3). The combined organic layers were washed with brine, dried over Na_2SO_4, filtered and concentrated *in vacuum*. The product was purified by flash silica gel column chromatography to afford **1j** (583 mg, 76% yield over 2 steps).

Physical state: white solid;

Melting point: 90–95 °C;

Yield: 73%;

R_f = 0.5 (silica gel, PE: EtOAc = 1:1);

^{1}H NMR (400 MHz, $CDCl_3$): δ 7.38 – 7.27 (m, 6H), 7.13 (s, 1H), 5.08 (s, 2H), 3.71 (s, 3H), 3.47 (s, 2H).

^{13}C NMR (100 MHz, $CDCl_3$): δ 170.8, 160.3, 135.8, 135.2, 131.6, 129.2, 128.4, 128.3, 116.0, 115.9, 52.6, 52.0, 41.6.

HRMS (ESI-TOF): calc'd for $C_{15}H_{14}INNaO_3^+$ [M+Na$^+$] 405.9911, found 405.9914.

Supplementary Note 6: General procedures for the synthesis of **4a−z** and **4a'−o'**

A 4 mL oven-dried Schlenk tube equipped with a magnetic stir bar was charged with palladium acetate (0.005 mmol), norbornene derivatives **N[8]** (0.05 mmol, 0.5 equiv), alkenyl iodide **1** (0.12 mmol, 1.2 equiv) and potassium carbonate (0.25 mmol, 2.5 equiv) and anhydrous 1,4-dioxane (1 mL) in the glove box. Then alkylating reagent **2** (0.15 mmol, 1.5 equiv) and terminating reagent **3** (0.1 mmol, 1.0 equiv) were added, and the mixture was heated to 105 °C and stirred for 36 h. After completion of the reaction (monitored by TLC), the mixture was cooled to r.t., filtered through a thin pad of celite, eluting with EtOAc (10 mL), and the combined filtrate was concentrated *in vacuo*. The residue was directly purified by column chromatography on silica gel or purified by PTLC (preparative thin layer chromatography) to give the desired product **4a−l, n-z** and **4a'−o'**.

Ethyl (*E*)-4-(1-benzyl-5-methyl-2-oxo-4-styryl-1,2-dihydropyridin-3-yl)butanoate (**4a**)

Physical state: yellow solid;

Melting point: 60–65 ºC;

Yield: 95%;

R_f = 0.4 (silica gel, PE: EtOAc = 2:1);

^{1}H NMR (400 MHz, $CDCl_3$): δ 7.55 – 7.47 (m, 2H), 7.43 – 7.26 (m, 8H), 6.99 (s, 1H), 6.93 (d, *J* = 16.7 Hz, 1H), 6.66 (d, *J* = 16.7 Hz, 1H), 5.13 (s, 2H), 4.02 (q, *J* = 7.1 Hz, 2H), 2.79 – 2.65 (m, 2H), 2.37 (t, *J* = 7.4 Hz, 2H), 2.02 (s, 3H), 1.99 – 1.85 (m, 2H), 1.17 (t, *J* = 7.1 Hz, 3H).

^{13}C NMR (100 MHz, $CDCl_3$): δ 173.8, 162.0, 147.3, 137.0, 136.6, 135.7, 132.2, 130.2, 128.9 (2C), 128.5, 128.2, 128.0, 126.8, 124.3, 114.5, 60.3, 52.2, 34.5, 28.0, 24.1, 17.6, 14.3.

HRMS (ESI-TOF): calc'd for $C_{27}H_{29}NNaO_3^+$ [M+Na$^+$] 438.2040, found 438.2044.

Ethyl (*E*)-4-(1,5-dimethyl-2-oxo-4-styryl-1,2-dihydropyridin-3-yl)butanoate (**4b**)

Physical state: yellow solid;

Melting point: 60–65 ºC;

Yield: 87%;

R_f = 0.2 (silica gel, PE: EtOAc = 1:1);

^{1}H NMR (400 MHz, $CDCl_3$): δ 7.56 – 7.48 (m, 2H), 7.43 – 7.28 (m, 3H), 6.99 (s, 1H), 6.93 (d, *J* = 16.7 Hz, 1H), 6.64 (d, *J* = 16.7 Hz, 1H), 4.02 (q, *J* = 7.1 Hz, 2H), 3.53 (s, 3H), 2.79 – 2.63 (m, 2H), 2.36 (t, *J* = 7.4 Hz, 2H), 2.06 (s, 3H), 1.95 – 1.84 (m, 2H), 1.16 (t, *J* = 7.1 Hz, 3H).

^{13}C NMR (100 MHz, $CDCl_3$): δ 173.8, 162.5, 147.4, 136.7, 135.6, 133.4, 129.8, 128.9, 128.5, 126.8, 124.4, 114.1, 60.3, 37.6, 34.5, 27.8, 24.2, 17.5, 14.3.

HRMS (ESI-TOF): calc'd for $C_{21}H_{25}NNaO_3^+$ [M+Na$^+$] 362.1727, found 362.1730.

Ethyl (*E*)-4-(5-methyl-2-oxo-4-styryl-1-(2,4,6-trimethylbenzyl)-1,2-dihydropyridin-3-

yl)butanoate (**4c**)

Physical state: yellow oil;

Yield: 80%;

R_f = 0.4 (silica gel, PE: EtOAc = 2:1);

^{1}H NMR (400 MHz, $CDCl_3$): δ 7.54 – 7.47 (m, 2H), 7.43 – 7.27 (m, 3H), 6.95 – 6.90 (m, 3H), 6.64 (d, J = 16.7 Hz, 1H), 6.44 (s, 1H), 5.12 (s, 2H), 4.06 – 4.01 (m, 2H), 2.85 – 2.68 (m, 2H), 2.39 (t, J = 7.5 Hz, 2H), 2.32 (s, 3H), 2.22 (s, 6H), 1.96 – 1.89 (m, 5H), 1.18 (t, J = 7.1 Hz, 3H).

^{13}C NMR (100 MHz, $CDCl_3$): δ 173.8, 162.4, 146.9, 138.6, 138.5, 136.7, 135.6, 129.6, 129.4, 129.3, 128.9, 128.6, 128.4, 126.8, 124.5, 114.2, 60.3, 45.4, 34.5, 28.0, 24.2, 21.2, 19.9, 17.9, 14.4.

HRMS (ESI-TOF): calc'd for $C_{30}H_{35}NNaO_3^+$ [M+Na$^+$] 480.2509, found 480.2516.

Ethyl (*E*)-4-(1-(methoxymethyl)-5-methyl-2-oxo-4-styryl-1,2-dihydropyridin-3-yl)butanoate (**4d**)

Physical state: white solid;

Melting point: 95–98 °C;

Yield: 95%;

R_f = 0.45 (silica gel, PE: EtOAc = 2:1);

^{1}H NMR (400 MHz, $CDCl_3$): δ 7.52 (d, J = 7.4 Hz, 2H), 7.39 (t, J = 7.5 Hz, 2H), 7.31 (t, J = 7.2 Hz, 1H), 7.07 (s, 1H), 6.93 (d, J = 16.7 Hz, 1H), 6.67 (d, J = 16.7 Hz, 1H), 5.31 (s, 2H), 4.02 (q, J = 7.1 Hz, 2H), 3.41 (s, 3H), 2.74–2.67 (m, 2H), 2.36 (t, J = 7.4 Hz, 2H), 2.07 (s, 3H), 1.89 (p, J = 7.5 Hz, 2H), 1.16 (t, J = 7.1 Hz, 3H);

^{13}C NMR (100 MHz, $CDCl_3$): δ 173.7, 162.5, 148.0, 136.6, 135.9, 131.0, 130.2, 128.9, 128.6, 126.9, 124.2, 114.6, 78.5, 60.3, 57.2, 34.4, 27.8, 24.1, 17.6, 14.3;

HRMS (ESI-TOF): calc'd for $C_{22}H_{28}NO_4^+$ [M+H$^+$] 370.2013, found 370.2017.

Diethyl 4,4'-(5-methyl-2-oxo-4-styrylpyridine-1,3(2*H*)-diyl)(*E*)-dibutyrate (**4e**)

Physical state: yellow oil;

Yield: 69%;

R_f = 0.3 (silica gel, PE: EtOAc = 2:1);

^{1}H NMR (400 MHz, $CDCl_3$): δ 7.51 (d, *J* = 7.2 Hz, 2H), 7.38 (t, *J* = 7.5 Hz, 2H), 7.34 – 7.28 (m, 1H), 7.00 – 6.89 (m, 2H), 6.65 (d, *J* = 16.7 Hz, 1H), 4.20 – 4.10 (m, 2H), 4.05 – 3.89 (m, 4H), 2.74 – 2.65 (m, 2H), 2.41 – 2.32 (m, 4H), 2.14 – 2.00 (m, 5H), 1.94 – 1.84 (m, 2H), 1.30 – 1.23 (m, 3H), 1.16 (t, *J* = 7.1 Hz, 3H).

^{13}C NMR (100 MHz, $CDCl_3$): δ 173.8, 173.1, 161.9, 147.3, 139.1, 136.7, 135.7, 132.6, 130.1, 128.9, 128.5, 126.8, 124.3, 114.3, 60.7, 60.3, 49.0, 34.5, 31.3, 27.9, 24.6, 24.1, 17.6, 14.4, 14.3.

HRMS (ESI-TOF): calc'd for $C_{26}H_{33}NNaO_5^+$ [M+Na$^+$] 462.2251, found 462.2256.

Ethyl (*E*)-4-(1-benzyl-5-fluoro-2-oxo-4-styryl-1,2-dihydropyridin-3-yl)butanoate (**4f**)

Physical state: yellow oil;

Yield: 83%;

R_f = 0.4 (silica gel, PE: EtOAc = 2:1);

^{1}H NMR (400 MHz, $CDCl_3$): δ 7.61 – 7.55 (m, 2H), 7.42 – 7.26 (m, 9H), 7.13 – 7.05 (m, 2H), 5.12 (s, 2H), 4.15 – 4.07 (m, 2H), 2.89 – 2.74 (m, 2H), 2.43 (t, *J* = 7.1 Hz, 2H), 1.91 (dq, *J* = 10.4, 7.3 Hz, 2H), 1.22 (t, *J* = 7.1 Hz, 3H).

^{13}C NMR (100 MHz, $CDCl_3$): δ 173.7, 160.8, 146.6 (d, *J* = 234.0 Hz), 139.0 (d, *J* = 11.7 Hz), 136.8 (d, *J* = 3.3 Hz), 136.7, 136.2, 131.2 (d, *J* = 3.0 Hz), 129.1(2C), 128.9, 128.5, 128.4, 127.3, 119.6 (d, *J* = 40.2 Hz), 119.0 (d, *J* = 2.7 Hz), 60.4, 52.4, 34.0, 27.4, 23.9, 14.4.

^{19}F NMR (376 MHz, $CDCl_3$): δ -148.8.

HRMS (ESI-TOF): calc'd for $C_{26}H_{26}FNNaO_3^+$ [M+Na$^+$] 442.1789, found 442.1782.

Ethyl (*E*)-4-(5-fluoro-1-(4-methoxybenzyl)-2-oxo-4-styryl-1,2-dihydropyridin-3-yl)butanoate (**4g**)

Physical state: yellow oil;

Yield: 75%;

R_f = 0.4 (silica gel, PE: EtOAc = 2:1);

^{1}H NMR (400 MHz, $CDCl_3$): δ 7.61 – 7.55 (m, 2H), 7.42 – 7.24 (m, 6H), 7.14 – 7.04 (m, 2H), 6.94 – 6.83 (m, 2H), 5.04 (s, 2H), 4.11 (q, *J* = 7.1 Hz, 2H), 3.81 (s, 3H), 2.98 – 2.75 (m, 2H), 2.42 (t, *J* = 7.1 Hz, 2H), 1.99 – 1.81 (m, 2H), 1.22 (t, *J* = 7.2 Hz, 3H).
^{13}C NMR (100 MHz, $CDCl_3$): δ 173.7, 160.8, 159.7, 146.5 (d, *J* = 234.2 Hz), 138.9 (d, *J* = 11.6 Hz), 136.8, 136.7 (d, *J* = 14.6 Hz), 131.1 (d, *J* = 2.2 Hz), 130.1, 129.1, 128.9, 128.1, 127.3, 119.4 (d, *J* = 40.4 Hz), 119.0 (d, *J* = 2.8 Hz), 114.5, 60.4, 55.5, 51.9, 34.0, 27.4, 23.9, 14.4.
^{19}F NMR (376 MHz, $CDCl_3$) δ -149.0.
HRMS (ESI-TOF): calc'd for $C_{27}H_{28}FNNaO_4^+$ [M+Na$^+$] 472.1895, found 472.1889.

Ethyl (*E*)-4-(1-benzyl-5-chloro-2-oxo-4-styryl-1,2-dihydropyridin-3-yl)butanoate (**4h**)
Physical state: colorless oil;
Yield: 60%;
R_f = 0.5 (silica gel, PE: EtOAc = 2:1);
^{1}H NMR (400 MHz, $CDCl_3$): δ 7.57 – 7.53 (m, 2H), 7.41 – 7.35 (m, 4H), 7.34 – 7.30 (m, 4H), 7.28 (s, 1H), 6.98 – 6.84 (m, 2H), 5.12 (s, 2H), 4.04 (q, *J* = 7.1 Hz, 2H), 2.87 – 2.65 (m, 2H), 2.38 (t, *J* = 7.3 Hz, 2H), 1.98 – 1.86 (m, 2H), 1.18 (t, *J* = 7.1 Hz, 3H).
^{13}C NMR (100 MHz, $CDCl_3$): δ 173.6, 161.3, 144.7, 137.6, 136.3, 136.1, 132.2, 131.8, 129.2, 128.9 (2C), 128.5, 128.4, 127.1, 122.3, 113.0, 60.4, 52.5, 34.3, 28.6, 24.0, 14.3.
HRMS (ESI-TOF): calc'd for $C_{26}H_{26}ClNNaO_3^+$ [M+Na$^+$] 458.1493, found 458.1496.

Ethyl (*E*)-4-(1-benzyl-5-(methoxymethyl)-2-oxo-4-styryl-1,2-dihydropyridin-3-yl)butanoate (**4i**)
Physical state: colorless oil;
Yield: 61%;
R_f = 0.4 (silica gel, PE: EtOAc = 2:1);
^{1}H NMR (400 MHz, $CDCl_3$): δ 7.58 – 7.47 (m, 2H), 7.43 – 7.25 (m, 8H), 7.23 (s, 1H), 7.04 (d, *J* = 16.7 Hz, 1H), 6.81 (d, *J* = 16.7 Hz, 1H), 5.15 (s, 2H), 4.13 (s, 2H), 4.03 (q, *J* = 7.2 Hz, 2H), 3.34 (s, 3H), 2.81 – 2.67 (m, 2H), 2.37 (t, *J* = 7.4 Hz, 2H), 1.97 – 1.86 (m, 2H), 1.17 (t, *J* = 7.1 Hz, 3H).

^{13}C NMR (100 MHz, $CDCl_3$): δ 173.8, 162.3, 146.8, 136.7 (2C), 136.1, 134.7, 130.5, 129.0, 128.9, 128.6, 128.3, 128.1, 127.0, 123.2, 115.1, 71.1, 60.3, 58.1, 52.5, 34.4, 27.9, 24.0, 14.3.

HRMS (ESI-TOF): calc'd for $C_{28}H_{31}NNaO_4^+$ [M+Na$^+$] 468.2145, found 468.2149.

Ethyl (*E*)-4-(1-benzyl-5-(2-methoxy-2-oxoethyl)-2-oxo-4-styryl-1,2-dihydropyridin-3-yl)butanoate (**4j**)

Physical state: yellow oil;

Yield: 83%;

R_f = 0.3 (silica gel, PE: EtOAc = 2:1);

^1H NMR (400 MHz, $CDCl_3$): δ 7.52 – 7.42 (m, 2H), 7.39 – 7.26 (m, 8H), 7.10 (s, 1H), 6.88 (d, J = 16.7 Hz, 1H), 6.58 (d, J = 16.7 Hz, 1H), 5.15 (s, 2H), 4.01 (q, J = 7.1 Hz, 2H), 3.59 (s, 3H), 3.38 (s, 2H), 2.77 – 2.64 (m, 2H), 2.35 (t, J = 7.5 Hz, 2H), 1.95 – 1.85 (m, 2H), 1.16 (t, J = 7.1 Hz, 3H).

^{13}C NMR (100 MHz, $CDCl_3$): δ 173.7, 172.0, 162.0, 147.2, 136.6, 136.4, 136.0, 134.3, 130.9, 129.0, 128.9, 128.6, 128.3, 128.1, 126.8, 123.6, 111.8, 60.3, 52.4, 52.2, 36.3, 34.5, 28.2, 24.0, 14.3.

HRMS (ESI-TOF): calc'd for $C_{29}H_{31}NNaO_5^+$ [M+Na$^+$] 496.2094, found 496.2098.

Ethyl (*E*)-4-(1-benzyl-5-methyl-6-oxo-4-styryl-1,6-dihydropyridin-3-yl)butanoate (**4k**)

Physical state: colorless oil;

Yield: 94%;

R_f = 0.4 (silica gel, PE: EtOAc = 2:1);

^1H NMR (400 MHz, $CDCl_3$): δ 7.54 – 7.46 (m, 2H), 7.41 – 7.37 (m, 2H), 7.41 – 7.29 (m, 6H), 7.00 – 6.88 (m, 2H), 6.63 (d, J = 16.6 Hz, 1H), 5.15 (s, 2H), 4.06 (q, J = 7.1 Hz, 2H), 2.49 – 2.36 (m, 2H), 2.28 – 2.22 (m, 5H), 1.83 – 1.73 (m, 2H), 1.19 (t, J = 7.1 Hz, 3H).

^{13}C NMR (100 MHz, $CDCl_3$): δ 173.3, 162.5, 146.5, 137.0, 136.6, 136.4, 131.7, 128.9, 128.5, 128.2, 128.0, 127.1, 126.8, 124.1, 118.1, 60.5, 52.5, 33.6, 30.0, 25.3, 15.1, 14.4.

HRMS (ESI-TOF): calc'd for $C_{27}H_{29}NNaO_3^+$ [M+Na$^+$] 438.2040, found 438.2046.

Diethyl 4,4'-(1-benzyl-2-oxo-4-styryl-1,2-dihydropyridine-3,5-diyl)(*E*)-dibutyrate (**4l**)

Physical state: colorless oil;

Yield: 39%;

R_f = 0.3 (silica gel, PE: EtOAc = 4:1);

^{1}H NMR (400 MHz, $CDCl_3$): δ 7.57 – 7.45 (m, 2H), 7.40-7.34 (m, 3H), 7.33 – 7.26 (m, 5H), 6.99 (s, 1H), 6.92 (d, J = 16.7 Hz, 1H), 6.62 (d, J = 16.7 Hz, 1H), 5.14 (s, 2H), 4.12 – 3.94 (m, 4H), 2.76 – 2.62 (m, 2H), 2.49 – 2.31 (m, 4H), 2.23 (t, J = 7.2 Hz, 2H), 1.95 – 1.85 (m, 2H), 1.82 – 1.73 (m, 2H), 1.21 – 1.13 (m, 6H).

^{13}C NMR (100 MHz, $CDCl_3$): δ 173.8, 173.3, 161.9, 147.3, 136.9, 136.6, 135.6, 132.5, 130.6, 129.0, 128.9, 128.6, 128.5, 128.2, 128.0, 126.9, 123.8, 118.2, 60.5, 60.3, 52.4, 34.5, 33.6, 30.0, 28.2, 25.3, 24.2, 14.3.

HRMS (ESI-TOF): calc'd for $C_{32}H_{37}NNaO_5^+$ [M+Na$^+$] 538.2564, found 538.2569.

(*E*)-1-benzyl-3,5-dimethyl-4-styrylpyridin-2(1*H*)-one (**4n**)

Physical state: colorless oil;

Yield: 92%;

R_f = 0.4 (silica gel, PE: EtOAc = 2:1);

^{1}H NMR (400 MHz, $CDCl_3$): δ 7.52 – 7.48 (m, 2H), 7.41-7.37 (m, 2H), 7.35– 7.27 (m, 6H), 6.97 (s, 1H), 6.90 (d, J = 16.6 Hz, 1H), 6.65 (d, J = 16.6 Hz, 1H), 5.15 (s, 2H), 2.27 (s, 3H), 2.03 (s, 3H).

^{13}C NMR (100 MHz, $CDCl_3$): δ 162.6, 146.7, 137.1, 136.7, 136.3, 131.5, 129.1, 128.9, 128.5, 128.3, 128.0, 126.7 (2C), 124.7, 114.6, 52.3, 17.4, 14.9.

HRMS (ESI-TOF): calc'd for $C_{22}H_{21}NNaO^+$ [M+Na$^+$] 338.1515, found 338.1519.

(*E*)-1-benzyl-5-methyl-3-(methyl-d3)-4-styrylpyridin-2(1*H*)-one (**4o**)

Physical state: colorless oil;

Yield: 85%;

R_f = 0.4 (silica gel, PE: EtOAc = 2:1);

^{1}H NMR (400 MHz, $CDCl_3$): δ 7.52 – 7.47 (m, 2H), 7.41-7.37 (m, 2H), 7.35 – 7.29 (m, 6H), 6.97 (s, 1H), 6.89 (d, *J* = 16.6 Hz, 1H), 6.65 (d, *J* = 16.6 Hz, 1H), 5.15 (s, 2H), 2.03 (s, 3H).

^{13}C NMR (100 MHz, $CDCl_3$): δ 162.7, 146.7, 137.1, 136.7, 136.3, 131.5, 129.1, 128.9, 128.5, 128.3, 127.9, 126.7, 124.7, 114.6, 52.2, 17.4.

HRMS (ESI-TOF): calc'd for $C_{22}H_{18}D_3NNaO^+$ [M+Na$^+$] 341.1704, found 341.1709.

(*E*)-1-benzyl-3-ethyl-5-methyl-4-styrylpyridin-2(1*H*)-one (**4p**)

Physical state: yellow oil;

Yield: 92%;

R_f = 0.4 (silica gel, PE: EtOAc = 2:1);

^{1}H NMR (400 MHz, $CDCl_3$): δ 7.51 – 7.48 (m, 2H), 7.41-7.37 (m, 2H), 7.36– 7.29 (m, 6H), 6.97 (s, 1H), 6.92 (d, *J* = 16.6 Hz, 1H), 6.66 (d, *J* = 16.6 Hz, 1H), 5.15 (s, 2H), 2.73 (q, *J* = 7.4 Hz, 2H), 2.01 (s, 3H), 1.17 (t, *J* = 7.4 Hz, 3H).

^{13}C NMR (100 MHz, $CDCl_3$): δ 162.0, 146.6, 137.1, 136.7, 135.3, 132.7, 131.8, 128.9, 128.4, 128.3, 127.9, 126.7, 124.5, 114.5, 52.1, 21.9, 17.6, 13.6.

HRMS (ESI-TOF): calc'd for $C_{23}H_{23}NNaO^+$ [M+Na$^+$] 352.1672, found 352.1675.

(*E*)-1-benzyl-3-butyl-5-methyl-4-styrylpyridin-2(1*H*)-one (**4q**)

Physical state: yellow oil;

Yield: 93%;

R_f = 0.4 (silica gel, PE: EtOAc = 2:1);

^{1}H NMR (400 MHz, $CDCl_3$): δ 7.53 – 7.45 (m, 2H), 7.43 – 7.37 (m, 2H) ,7.36 – 7.28 (m, 6H), 6.97 (s, 1H), 6.91 (d, *J* = 16.7 Hz, 1H), 6.65 (d, *J* = 16.7 Hz, 1H), 5.14 (s, 2H), 2.80 – 2.61 (m, 2H), 2.02 (s, 3H), 1.60 – 1.52 (m, 2H), 1.45 – 1.36 (m, 2H), 0.92 (t, *J* = 7.3 Hz, 3H).

^{13}C NMR (100 MHz, $CDCl_3$): δ 162.2, 146.7, 137.1, 136.8, 135.3, 131.8, 131.6, 128.9, 128.4, 128.2, 127.9, 126.7, 124.6, 114.5, 52.1, 31.2, 28.4, 23.2, 17.6, 14.1.

HRMS (ESI-TOF): calc'd for $C_{25}H_{27}NNaO^+$ [M+Na$^+$] 380.1985, found 380.1988.

(*E*)-1,3-dibenzyl-5-methyl-4-styrylpyridin-2(1*H*)-one (**4r**)

Physical state: colorless oil;

Yield: 91%;

R_f = 0.4 (silica gel, PE: EtOAc = 2:1);

^{1}H NMR (400 MHz, $CDCl_3$): δ 7.39-7.27 (m, 11H), 7.22 – 7.25 (m, 3H), 7.14-7.17 (m, 1H), 7.02 (s, 1H), 6.88 (d, *J* = 16.7 Hz, 1H), 6.55 (d, *J* = 16.7 Hz, 1H), 5.17 (s, 2H), 4.11 (s, 2H), 2.02 (s, 3H).

^{13}C NMR (100 MHz, $CDCl_3$): δ 162.4, 148.3, 141.2, 137.0, 136.5, 135.9, 132.7, 129.4, 129.0, 128.9, 128.6, 128.5 (2C), 128.2, 128.0, 126.8, 125.9, 124.3, 114.6, 52.3, 34.0, 17.5.

HRMS (ESI-TOF): calc'd for $C_{28}H_{26}NO^+$ [M+H$^+$] 392.2009, found 392.2003.

(*E*)-1-benzyl-3-(2-(2,3-dihydrobenzofuran-5-yl)ethyl)-5-methyl-4-styrylpyridin-2(1*H*)-one (**4s**)

Physical state: yellow oil;

Yield: 96%;

R_f = 0.3 (silica gel, PE: EtOAc = 2:1);

^{1}H NMR (400 MHz, $CDCl_3$): δ 7.44 – 7.28 (m, 10H), 7.04 (s, 1H), 7.01 – 6.98 (s, 1H), 6.86 – 6.82 (m, 1H), 6.71 – 6.61 (m, 2H), 6.47 (d, *J* = 16.7 Hz, 1H), 5.19 (s, 2H), 4.49 (t, *J* = 8.7 Hz, 2H), 3.05 (t, *J* = 8.6 Hz, 2H), 2.98 – 2.93 (m, 2H), 2.85 – 2.81 (m, 2H), 1.98 (s, 3H).

^{13}C NMR (100 MHz, $CDCl_3$): δ 162.0, 158.3, 147.6, 137.1, 136.7, 135.1, 134.5, 132.1, 130.2, 129.0, 128.9, 128.4, 128.2 (2C), 128.0, 127.0, 126.7, 125.4, 124.5, 114.5, 108.8, 71.2, 52.1, 34.3, 31.7, 29.8, 17.5.

HRMS (ESI-TOF): calc'd for $C_{31}H_{29}NNaO_2^+$ [M+Na$^+$] 470.2091, found 470.2094.

(*E*)-1-benzyl-3-(3-methoxypropyl)-5-methyl-4-styrylpyridin-2(1*H*)-one (**4t**)

Physical state: yellow oil;

Yield: 88%;

R_f = 0.4 (silica gel, PE: EtOAc = 2:1);

^{1}H NMR (400 MHz, $CDCl_3$): δ 7.55 – 7.46 (m, 2H), 7.41 – 7.27 (m, 8H), 6.98 (s, 1H), 6.95 (d, J = 16.7 Hz, 1H), 6.68 (d, J = 16.7 Hz, 1H), 5.14 (s, 2H), 3.43 (t, J = 6.5 Hz, 2H), 3.30 (s, 3H), 2.88 – 2.73 (m, 2H), 2.03 (s, 3H), 1.94 – 1.75 (m, 2H).

^{13}C NMR (100 MHz, $CDCl_3$): δ 162.1, 147.0, 137.1, 136.8, 135.6, 132.1, 130.7, 128.9 (2C), 128.4, 128.2, 128.0, 126.8, 124.5, 114.4, 72.7, 58.5, 52.2, 28.7, 25.2, 17.7.

HRMS (ESI-TOF): calc'd for $C_{25}H_{27}NNaO_2^+$ [M+Na$^+$] 396.1934, found 396.1931.

(*E*)-1-benzyl-3-(3-hydroxypropyl)-5-methyl-4-styrylpyridin-2(1*H*)-one (**4u**)

Physical state: colorless oil;

Yield: 72%;

R_f = 0.2 (silica gel, PE: EtOAc = 1:1);

^{1}H NMR (400 MHz, $CDCl_3$): δ 7.50 – 7.46 (m, 2H), 7.41-7.36 (m, 3H), 7.34 – 7.29 (m, 5H), 7.06 – 7.03 (m, 1H), 6.93 (d, J = 16.7 Hz, 1H), 6.67 (d, J = 16.7 Hz, 1H), 5.18 (s, 2H), 3.51 (t, J = 5.6 Hz, 2H), 2.88 (t, J = 6.6 Hz, 2H), 2.05 (s, 3H), 1.82 – 1.68 (m, 2H).

^{13}C NMR (100 MHz, $CDCl_3$): δ 163.3, 148.5, 136.8, 136.5, 135.9, 132.5, 130.2, 129.1, 129.0, 128.7, 128.1 (2C), 126.8, 123.9, 115.6, 60.5, 52.6, 32.4, 23.4, 17.6.

HRMS (ESI-TOF): calc'd for $C_{24}H_{25}NNaO_2^+$ [M+Na$^+$] 382.1778, found 382.1775.

(*E*)-4-(1-benzyl-5-methyl-2-oxo-4-styryl-1,2-dihydropyridin-3-yl)butanenitrile (**4v**)

Physical state: yellow oil;

Yield: 90%;

R_f = 0.4 (silica gel, PE:EtOAc = 2:1);

^{1}H NMR (400 MHz, $CDCl_3$): δ 7.54 – 7.48 (m, 2H), 7.42-7.36 (m, 3H), 7.34 – 7.31 (m, 5H), 7.03 (s, 1H), 6.92 (d, J = 16.7 Hz, 1H), 6.64 (d, J = 16.7 Hz, 1H), 5.13 (s, 2H), 2.92 – 2.76 (m, 2H), 2.39 (t, J = 7.4 Hz, 2H), 2.03 (s, 3H), 2.00 – 1.93 (m, 2H).

^{13}C NMR (100 MHz, $CDCl_3$): δ 162.0, 148.0, 136.8, 136.3, 136.1, 132.7, 129.0, 128.7 (2C), 128.2, 128.1, 126.8, 123.9, 120.0, 114.6, 52.3, 27.7, 24.7, 17.5, 17.3.

HRMS (ESI-TOF): calc'd for $C_{25}H_{24}N_2NaO^+$ [M+Na$^+$] 391.1781, found 391.1788.

(*E*)-1-benzyl-3-(4-chlorobutyl)-5-methyl-4-styrylpyridin-2(1*H*)-one (**4w**)

Physical state: yellow oil;

Yield: 92%;

R_f = 0.3 (silica gel, PE: EtOAc = 2:1);

^{1}H NMR (400 MHz, $CDCl_3$): δ 7.53 – 7.47 (m, 2H), 7.41-7.36 (m, 2H), 7.34– 7.28 (m, 8H), 6.99 (s, 1H), 6.91 (d, *J* = 16.7 Hz, 1H), 6.65 (d, *J* = 16.6 Hz, 1H), 5.14 (s, 2H), 3.56 (t, *J* = 6.6 Hz, 2H), 2.82 – 2.64 (m, 2H), 2.02 (s, 3H), 1.90 – 1.81 (m, 2H), 1.77 – 1.66 (m, 2H).

^{13}C NMR (100 MHz, $CDCl_3$): δ 162.1, 147.2, 137.0, 136.6, 135.6, 132.1, 130.6, 129.0, 128.6, 128.2, 128.0, 126.8, 124.3, 114.6, 52.2, 45.1, 32.9, 27.8, 26.2, 17.6.

HRMS (ESI-TOF): calc'd for $C_{25}H_{26}ClNNaO^+$ [M+Na$^+$] 414.1595, found 414.1597.

(*E*)-1-benzyl-3-(4-bromobutyl)-5-methyl-4-styrylpyridin-2(1*H*)-one (**4x**)

Physical state: yellow oil;

Yield: 45%;

R_f = 0.4 (silica gel, PE: EtOAc = 2:1);

^{1}H NMR (400 MHz, $CDCl_3$): δ 7.54 – 7.48 (m, 2H), 7.42 – 7.27 (m, 8H), 6.99 (s, 1H), 6.91 (d, *J* = 16.6 Hz, 1H), 6.66 (d, *J* = 16.6 Hz, 1H), 5.14 (s, 2H), 3.43 (t, *J* = 6.8 Hz, 1H), 3.21 (t, *J* = 7.0 Hz, 1H), 2.73 – 2.68 (m, 2H), 2.02 (s, 3H), 1.98 – 1.85 (m, 2H), 1.81 – 1.67 (m, 2H).

^{13}C NMR (100 MHz, $CDCl_3$): δ 162.1, 147.2, 137.0, 136.6, 135.6, 132.1, 130.5, 129.0, 128.5, 128.2, 128.0, 126.82, 126.78, 124.3, 114.6, 52.2, 34.1, 33.1, 27.7, 27.5, 17.6.

HRMS (ESI-TOF): calc'd for $C_{25}H_{26}BrNNaO^+$ [M+Na$^+$] 458.1090, found 458.1093.

(*E*)-6-(1-benzyl-5-methyl-2-oxo-4-styryl-1,2-dihydropyridin-3-yl)-N,N-diethylhexanamide (**4y**)

Physical state: yellow oil;

Yield: 75%;

R_f = 0.2 (silica gel, PE: EtOAc = 1:1);

^{1}H NMR (400 MHz, $CDCl_3$): δ 7.50 – 7.45 (m, 2H), 7.41 – 7.28 (m, 8H), 6.97 (s, 1H), 6.90 (d, *J* = 16.7 Hz, 1H), 6.63 (d, *J* = 16.7 Hz, 1H), 5.13 (s, 2H), 3.37 – 3.18 (m, 4H),

2.72 – 2.64 (m, 2H), 2.29 – 2.22 (m, 2H), 2.01 (s, 3H), 1.75 – 1.63 (m, 4H), 1.46 – 1.38 (m, 2H), 1.15 – 1.05 (m, 6H).

^{13}C NMR (100 MHz, $CDCl_3$): δ 172.5, 162.2, 146.9, 137.1, 136.7, 135.5, 131.9, 131.4, 129.0, 128.5, 128.2, 127.9, 126.7, 124.5, 114.6, 52.2, 42.1, 40.1, 33.3, 30.0, 28.9, 28.6, 25.6, 17.6, 14.5, 13.3.

HRMS (ESI-TOF): calc'd for $C_{31}H_{38}N_2NaO_2^+$ [M+Na$^+$] 493.2825, found 493.2829.

(*E*)-2-(2-(1-benzyl-5-methyl-2-oxo-4-styryl-1,2-dihydropyridin-3-yl)ethyl)isoindoline-1,3-dione (**4z**)

Physical state: yellow oil;

Yield: 52%;

R_f = 0.3 (silica gel, PE: EtOAc = 1:1);

^{1}H NMR (400 MHz, $CDCl_3$): δ 7.64 – 7.54 (m, 4H), 7.40 – 7.23 (m, 10H), 6.99 (s, 1H), 6.83 (d, J = 16.8 Hz, 1H), 6.37 (d, J = 16.8 Hz, 1H), 5.15 (s, 2H), 4.09 – 3.97 (m, 2H), 3.13 (t, J = 6.3 Hz, 2H), 1.94 (s, 3H).

^{13}C NMR (100 MHz, $CDCl_3$): δ 168.4, 162.0, 148.0, 137.0, 136.2, 136.0, 133.6, 133.2, 132.2, 128.9, 128.7, 128.5, 128.1, 127.9, 127.4, 126.7, 123.6, 123.1, 114.1, 52.0, 36.7, 27.7, 17.7.

HRMS (ESI-TOF): calc'd for $C_{31}H_{26}N_2NaO_3^+$ [M+Na$^+$] 497.1836, found 497.1833.

Ethyl (*E*)-4-(1-benzyl-4-(3-(tert-butoxy)-3-oxoprop-1-en-1-yl)-5-methyl-2-oxo-1,2-dihydropyridin-3-yl)butanoate (**4a'**)

Physical state: colorless oil;

Yield: 86%;

R_f = 0.5 (silica gel, PE: EtOAc = 2:1);

^{1}H NMR (400 MHz, $CDCl_3$): δ 7.46 (d, J = 16.4 Hz, 1H), 7.37 – 7.27 (m, 5H), 6.96 (s, 1H), 5.96 (d, J = 16.4 Hz, 1H), 5.10 (s, 2H), 4.10 (q, J = 7.1 Hz, 2H), 2.67 – 2.60 (m, 2H), 2.36 (t, J = 7.6 Hz, 2H), 1.95 (s, 3H), 1.89 – 1.78 (m, 2H), 1.53 (s, 9H), 1.24 (t, J = 7.1 Hz, 3H).

^{13}C NMR (100 MHz, $CDCl_3$): δ 173.5, 165.1, 161.7, 144.9, 139.5, 136.7, 132.5, 130.8, 129.0, 128.3, 128.1, 127.9, 113.5, 81.3, 60.3, 52.3, 34.4, 28.3, 27.9, 24.0, 17.3, 14.4.

HRMS (ESI-TOF): calc'd for $C_{26}H_{33}NNaO_5^+$ [M+Na$^+$] 462.2251, found 462.2258.

Ethyl (*E*)-4-(1-benzyl-4-(3-(dimethylamino)-3-oxoprop-1-en-1-yl)-5-methyl-2-oxo-1,2-dihydropyridin-3-yl)butanoate (**4b'**)

Physical state: colorless oil;

Yield: 82%;

R_f = 0.2 (silica gel, PE: EtOAc = 1:1);

^1H NMR (400 MHz, $CDCl_3$): δ 7.50 (d, J = 15.8 Hz, 1H), 7.36 – 7.27 (m, 5H), 6.97 (s, 1H), 6.52 (d, J = 15.8 Hz, 1H), 5.11 (s, 2H), 4.14 – 4.06 (m, 2H), 3.13 (s, 3H), 3.07 (s, 3H), 2.72 – 2.60 (m, 2H), 2.36 (t, J = 7.6 Hz, 2H), 1.95 (s, 3H), 1.90 – 1.80 (m, 2H), 1.22 (t, J = 7.1 Hz, 3H).

^{13}C NMR (100 MHz, $CDCl_3$): δ 173.6, 165.6, 161.8, 146.1, 138.3, 136.8, 132.3, 130.5, 129.0, 128.3, 128.1, 125.1, 113.8, 60.3, 52.3, 37.5, 36.1, 34.5, 28.2, 24.5, 17.3, 14.4.

HRMS (ESI-TOF): calc'd for $C_{24}H_{30}N_2NaO_4^+$ [M+Na$^+$] 433.2098, found 433.2105.

Ethyl (*E*)-4-(1-benzyl-4-(2-(diethoxyphosphoryl)vinyl)-5-methyl-2-oxo-1,2-dihydropyridin-3-yl)butanoate (**4c'**)

Physical state: yellow oil;

Yield: 84%;

R_f = 0.2 (silica gel, PE: EtOAc = 1:1);

^1H NMR (400 MHz, $CDCl_3$): δ 7.42 – 7.27 (m, 6H), 7.03 – 6.92 (s, 1H), 5.97 – 5.87 (m, 1H), 5.10 (s, 2H), 4.27 – 3.99 (m, 6H), 2.69 – 2.49 (m, 2H), 2.34 (t, J = 7.7 Hz, 2H), 1.94 (s, 3H), 1.88 – 1.74 (m, 2H), 1.36 (t, J = 7.1 Hz, 6H), 1.23 (t, J = 7.1 Hz, 3H).

^{13}C NMR (100 MHz, $CDCl_3$): δ 173.4, 161.7, 145.7 (d, J = 21.9 Hz), 144.8 (d, J = 5.3 Hz), 136.7, 132.7, 130.4, 129.0, 128.3, 128.1, 123.7 (d, J = 184.2 Hz), 112.9, 62.3, 62.2, 60.3, 52.3, 34.4, 27.9, 24.2, 17.2, 16.6, 16.5, 14.4.

^{31}P NMR (162 MHz, $CDCl_3$) δ 16.2.

HRMS (ESI-TOF): calc'd for $C_{25}H_{34}NNaO_6P^+$ [M+Na$^+$] 498.2016, found 498.2011.

Ethyl (*E*)-4-(1-benzyl-5-methyl-2-oxo-4-(2-(phenylsulfonyl)vinyl)-1,2-dihydropyridin-3-yl)butanoate (**4d'**)

Physical state: yellow oil;

Yield: 92%;

R_f = 0.2 (silica gel, PE: EtOAc = 1:2);

^{1}H NMR (400 MHz, $CDCl_3$): δ 8.02 – 7.93 (m, 2H), 7.70 – 7.53 (m, 4H), 7.38 – 7.23 (m, 5H), 6.98 (s, 1H), 6.63 (d, *J* = 15.8 Hz, 1H), 5.09 (s, 2H), 4.11 (q, *J* = 7.2 Hz, 2H), 2.59 – 2.46 (m, 2H), 2.22 (t, *J* = 7.6 Hz, 2H), 1.93 (s, 3H), 1.78 – 1.68 (m, 2H), 1.25 (t, *J* = 7.1 Hz, 3H).

^{13}C NMR (100 MHz, $CDCl_3$): δ 173.2, 161.4, 142.3, 140.0, 138.2, 136.5, 135.4, 134.0, 133.0, 131.5, 129.7, 129.0, 128.2 (2C), 128.0, 112.8, 60.5, 52.4, 34.2, 27.9, 24.0, 17.1, 14.4.

HRMS (ESI-TOF): calc'd for $C_{27}H_{29}NNaO_5S^+$ [M+Na$^+$] 502.1659, found 502.1666.

Ethyl 4-(1-benzyl-4-(2-(methoxycarbonyl)allyl)-5-methyl-2-oxo-1,2-dihydropyridin-3-yl)butanoate (**4e'**)

Physical state: yellow oil;

Yield: 80%;

R_f = 0.5 (silica gel, PE: EtOAc = 2:1);

^{1}H NMR (400 MHz, $CDCl_3$): δ 7.39 – 7.25 (m, 5H), 6.97 (s, 1H), 6.19 (s, 1H), 5.12 – 5.10 (m, 3H), 4.10 (q, *J* = 7.2 Hz, 2H), 3.82 (s, 3H), 3.52 (s, 2H), 2.62 – 2.49 (m, 2H), 2.37 (t, *J* = 7.5 Hz, 2H), 1.89 (s, 3H), 1.86 – 1.76 (m, 2H), 1.23 (t, *J* = 7.2 Hz, 3H).

^{13}C NMR (100 MHz, $CDCl_3$): δ 173.6, 167.2, 162.0, 146.4, 137.0, 136.9, 132.5, 131.8, 129.0, 128.3, 128.0, 125.4, 115.3, 60.3, 52.3, 52.2, 34.5, 31.5, 27.2, 24.3, 16.1, 14.4.

HRMS (ESI-TOF): calc'd for $C_{24}H_{29}NNaO_5^+$ [M+Na$^+$] 434.1938, found 434.1941.

Ethyl 4-(1-benzyl-5-methyl-2-oxo-4-vinyl-1,2-dihydropyridin-3-yl)butanoate (**4f'**)

Physical state: colorless oil;

Yield: 82%;

R_f = 0.5 (silica gel, PE: EtOAc = 2:1);

^{1}H NMR (400 MHz, $CDCl_3$): δ 7.37 – 7.27 (m, 5H), 6.94 (s, 1H), 6.52 (dd, J = 17.9, 11.7 Hz, 1H), 5.57 (dd, J = 11.8, 1.6 Hz, 1H), 5.32 (dd, J = 18.0, 1.6 Hz, 1H), 5.10 (s, 2H), 4.11 (q, J = 7.1 Hz, 2H), 2.82 – 2.59 (m, 2H), 2.37 (t, J = 7.5 Hz, 2H), 1.94 (s, 3H), 1.89 – 1.79 (m, 2H), 1.24 (t, J = 7.1 Hz, 3H).

^{13}C NMR (100 MHz, $CDCl_3$): δ 173.8, 162.0, 148.0, 137.0, 133.0, 132.1, 129.8, 128.9, 128.2, 127.9, 121.1, 114.1, 60.3, 52.2, 34.5, 27.8, 24.1, 17.4, 14.4.

HRMS (ESI-TOF): calc'd for $C_{21}H_{25}NNaO_3^+$ [M+Na$^+$] 362.1727, found 362.1733.

Ethyl (*E*)-4-(1-benzyl-4-(3-hydroxy-3-methylbut-1-en-1-yl)-5-methyl-2-oxo-1,2-dihydropyridin-3-yl)butanoate (**4g'**)

Physical state: colorless oil;

Yield: 90%;

R_f = 0.3 (silica gel, PE: EtOAc = 1:1);

^{1}H NMR (400 MHz, $CDCl_3$): δ 7.37 – 7.27 (m, 5H), 6.95 (s, 1H), 6.43 (d, J = 16.5 Hz, 1H), 5.92 (d, J = 16.5 Hz, 1H), 5.11 (s, 2H), 4.13 (q, J = 7.1 Hz, 2H), 2.70 – 2.53 (m, 2H), 2.39 (t, J = 6.5 Hz, 2H), 1.93 (s, 3H), 1.85 – 1.71 (m, 2H), 1.41 (s, 6H), 1.25 (t, J = 7.1 Hz, 3H).

^{13}C NMR (100 MHz, $CDCl_3$): δ 174.5, 162.0, 147.9, 144.4, 137.1, 132.0, 129.9, 128.9, 128.3, 128.0, 121.7, 114.5, 70.7, 60.6, 52.2, 33.9, 29.5, 27.6, 23.3, 17.2, 14.4.

HRMS (ESI-TOF): calc'd for $C_{24}H_{31}NNaO_4^+$ [M+Na$^+$] 420.2145, found 420.2141.

Ethyl (*E*)-4-(1-benzyl-5-methyl-4-(2-(naphthalen-2-yl)vinyl)-2-oxo-1,2-dihydropyridin-3-yl)butanoate (**4h'**)

Physical state: yellow oil;

Yield: 83%;

R_f = 0.3 (silica gel, PE: EtOAc = 2:1);

^{1}H NMR (400 MHz, $CDCl_3$): δ 7.91 – 7.80 (m, 3H), 7.75 – 7.73 (m, 1H), 7.53 – 7.44 (m, 2H), 7.33 (m, 6H), 7.13 – 6.97 (m, 2H), 6.83 (d, J = 16.7 Hz, 1H), 5.15 (s, 2H),

4.04 – 4.39 (m, 2H), 2.82 – 2.74 (m, 2H), 2.40 (t, *J* = 7.4 Hz, 2H), 2.07 (s, 3H), 1.99 – 1.91 (m, 2H), 1.14 (t, *J* = 7.1 Hz, 3H).

^{13}C NMR (100 MHz, $CDCl_3$): δ 173.8, 162.1, 147.3, 137.0, 135.9, 134.1, 133.7, 133.5, 132.3, 130.3, 129.0, 128.6, 128.3, 128.0, 127.9, 127.3, 126.6, 126.4, 124.6, 123.4, 114.5, 60.3, 52.2, 34.5, 28.1, 24.2, 17.7, 14.3.

HRMS (ESI-TOF): calc'd for $C_{31}H_{31}NNaO_3^+$ [M+Na$^+$] 488.2196, found 488.2191.

Ethyl (*E*)-4-(1-benzyl-5-methyl-2-oxo-4-(2-(1-tosyl-1*H*-indol-3-yl)vinyl)-1,2-dihydropyridin-3-yl)butanoate (**4i'**)

Physical state: yellow oil;

Yield: 88%;

R_f = 0.3 (silica gel, PE: EtOAc = 1:1);

^{1}H NMR (400 MHz, $CDCl_3$): δ 8.05 – 7.98 (m, 1H), 7.88 – 7.74 (m, 4H), 7.41 – 7.26 (m, 8H), 7.24 (s, 1H), 7.06 – 6.91 (m, 2H), 6.78 (d, *J* = 16.9 Hz, 1H), 5.14 (s, 2H), 4.02 (q, *J* = 7.1 Hz, 2H), 2.87 – 2.65 (m, 2H), 2.42 – 2.34 (m, 5H), 2.06 (s, 3H), 1.99 – 1.88 (m, 2H), 1.13 (t, *J* = 7.1 Hz, 3H).

^{13}C NMR (100 MHz, $CDCl_3$): δ 173.8, 162.1, 147.1, 145.3, 137.0, 135.6, 135.3, 132.4, 130.1, 129.0, 128.9, 128.3, 128.0, 127.1, 126.8, 125.3 (2C), 125.1, 123.8, 120.5, 120.0, 114.3, 114.0, 60.3, 52.2, 34.4, 28.1, 24.2, 21.8, 17.8, 14.3.

HRMS (ESI-TOF): calc'd for $C_{36}H_{36}N_2NaO_5S^+$ [M+Na$^+$] 631.2237, found 631.2239.

Ethyl (*E*)-4-(1-benzyl-4-(4-(4-((1-isopropoxy-2-methyl-1-oxopropan-2-yl)oxy)benzoyl)styryl)-5-methyl-2-oxo-1,2-dihydropyridin-3-yl)butanoate (**4j'**)

Physical state: yellow oil;

Yield: 90%;

R_f = 0.2 (silica gel, PE: EtOAc = 4:1);

^{1}H NMR (400 MHz, $CDCl_3$): δ 7.84 – 7.73 (m, 4H), 7.65 – 7.57 (m, 2H), 7.41 – 7.27 (m, 5H), 7.13 – 6.99 (m, 2H), 6.90 – 6.83 (m, 2H), 6.73 (d, *J* = 16.7 Hz, 1H), 5.18 – 5.04 (m, 3H), 4.04 (q, *J* = 7.1 Hz, 2H), 2.79 – 2.70 (m, 2H), 2.38 (t, *J* = 7.4 Hz, 2H), 2.04 (s, 3H), 1.97 – 1.87 (m, 2H), 1.66 (s, 6H), 1.24 – 1.14 (m, 9H).

^{13}C NMR (100 MHz, $CDCl_3$): δ 195.0, 173.8, 173.3, 162.0, 159.7, 146.8, 140.2, 137.8, 137.0, 134.8, 132.4, 132.1, 130.8, 130.5 (2C), 129.0, 128.3, 128.0, 126.7, 126.6, 117.3, 114.2, 79.5, 69.5, 60.3, 52.2, 34.4, 28.1, 25.5, 24.1, 21.7, 17.6, 14.4.

HRMS (ESI-TOF): calc'd for $C_{41}H_{45}NNaO_7^+$ [M+Na$^+$] 686.3088, found 686.3090.

1-Benzyl-3,5-dimethylpyridin-2(1*H*)-one (**4k'**)

Physical state: colorless oil;

Yield: 78%;

R_f = 0.3 (silica gel, PE: EtOAc = 2:1);

^1H NMR (400 MHz, $CDCl_3$): δ 7.38 – 7.25 (m, 5H), 7.07 (s, 1H), 6.93 (s, 1H), 5.12 (s, 2H), 2.16 (s, 3H), 2.00 (s, 3H).

^{13}C NMR (100 MHz, $CDCl_3$): δ 162.4, 139.7, 137.0, 132.1, 129.7, 128.9, 128.2, 127.9, 114.9, 52.2, 17.5, 17.4.

HRMS (ESI-TOF): calc'd for $C_{14}H_{16}NO^+$ [M+H$^+$] 214.1226, found 214.1221.

Both the proton and carbon NMR match the literature reported data.[11]

1-Benzyl-3,5-dimethylpyridin-2(1*H*)-one-4-*d* (**4l'**)

Physical state: colorless oil;

Yield: 74%;

R_f = 0.3 (silica gel, PE: EtOAc = 2:1);

^1H NMR (400 MHz, $CDCl_3$): δ 7.35 – 7.27 (m, 5H), 7.07 (s, 0.17 H), 6.93 (s, 1H), 5.12 (s, 2H), 2.15 (s, 3H), 2.00 (s, 3H).

^{13}C NMR (125 MHz, $CDCl_3$): δ 162.4, 139.7, 139.3 (t, J = 18.8 Hz) 137.0, 132.1, 129.6, 128.9, 128.2, 127.9, 114.8, 52.2, 17.4, 17.3.

HRMS (ESI-TOF): calc'd for $C_{14}H_{15}DNO^+$ [M+H$^+$] 215.1289, found 215.1281.

1-Benzyl-3,4,5-trimethylpyridin-2(1*H*)-one (**4m'**)

Physical state: colorless oil;

Yield: 52%;

R_f = 0.2 (silica gel, PE: EtOAc = 2:1);

^1H NMR (400 MHz, $CDCl_3$): δ 7.42 – 7.22 (m, 5H), 6.91 (s, 1H), 5.12 (s, 2H), 2.16 (s, 3H), 2.10 (s, 3H), 1.98 (s, 3H).

^{13}C NMR (100 MHz, $CDCl_3$): δ 162.3, 146.5, 137.2, 130.9, 128.9 (2C), 128.2, 128.1, 127.8, 126.4, 115.7, 52.2, 17.0, 16.5, 13.3.

HRMS (ESI-TOF): calc'd for $C_{15}H_{18}NO^+$ [M+H$^+$] 228.1383, found 228.1377.

1-Benzyl-3,5-dimethyl-4-phenylpyridin-2(1*H*)-one (**4n'**)

Physical state: yellow oil;

Yield: 80%;

R_f = 0.4 (silica gel, PE: EtOAc = 2:1);

^1H NMR (400 MHz, $CDCl_3$): δ 7.48 – 7.28 (m, 8H), 7.09 (s, 1H), 7.08 (s, 1H), 7.02 (s, 1H), 5.18 (s, 2H), 1.91 (s, 3H), 1.70 (s, 3H).

^{13}C NMR (100 MHz, $CDCl_3$): δ 162.5, 151.4, 138.6, 137.0, 131.4, 128.9, 128.7, 128.4, 128.0, 127.9, 127.6, 127.2, 114.8, 52.3, 17.4, 14.9.

HRMS (ESI-TOF): calc'd for $C_{20}H_{20}NO^+$ [M+H$^+$] 290.1539, found 290.1531.

1-Benzyl-3,5-dimethyl-4-((triisopropylsilyl)ethynyl)pyridin-2(1*H*)-one (**4o'**)

Physical state: yellow oil;

Yield: 72%;

R_f = 0.5 (silica gel, PE: EtOAc = 4:1);

^1H NMR (400 MHz, $CDCl_3$): δ 7.38 – 7.24 (m, 5H), 6.96 (s, 1H), 5.11 (s, 2H), 2.36 (s, 3H), 2.11 (s, 3H), 1.14 (s, 21H).

^{13}C NMR (100 MHz, $CDCl_3$): δ 161.8, 136.8, 133.2, 132.9, 131.4, 128.9, 128.1, 128.0, 115.7, 105.0, 101.9, 52.3, 18.8, 17.5, 15.9, 11.3.

HRMS (ESI-TOF): calc'd for $C_{25}H_{36}NOSi^+$ [M+H$^+$] 394.2561, found 394.2554.

Supplementary Note 7: General procedures for the synthesis of ethyl (*E*)-4-(1,3-dibenzyl-2,4-dioxo-6-styryl-1,2,3,4-tetrahydropyrimidin-5-yl)butanoate (**4m**).

A 4 mL oven-dried Schlenk tube equipped with a magnetic stir bar was charged with palladium acetate (0.01 mmol), norbornene derivatives $\mathbf{N^8}$ (0.1 mmol, 1.0 equiv),

alkenyl iodide **1l** (0.12 mmol, 1.2 equiv) and potassium carbonate (0.25 mmol, 2.5 equiv) and anhydrous 1,4-dioxane (1 mL) in the glove box. Then alkylating reagent **2a** (0.15 mmol, 1.5 equiv) and terminating reagent **3a** (0.1 mmol, 1.0 equiv) were added, and the mixture was heated to 130 °C and stirred for 48 h. After completion of the reaction (monitored by TLC), the mixture was cooled to r.t., filtered through a thin pad of celite, eluting with EtOAc (10 mL), and the combined filtrate was concentrated *in vacuo*. The residue was directly purified by column chromatography on silica gel to give the desired product **4m** (20.0 mg).

Physical state: colorless oil;

Yield: 37%;

R_f = 0.4 (silica gel, PE: EtOAc = 5:1);

^{1}H NMR (400 MHz, $CDCl_3$): δ 7.59 – 7.44 (m, 2H), 7.39 – 7.25 (m, 11H), 7.21 – 7.10 (m, 2H), 6.69 (d, J = 16.6 Hz, 1H), 6.48 (d, J = 16.6 Hz, 1H), 5.22 (s, 2H), 5.12 (s, 2H), 3.98 (q, J = 7.1 Hz, 2H), 2.53 – 2.43 (m, 2H), 2.31 (t, J = 7.3 Hz, 2H), 1.88 – 1.80 (m, 2H), 1.14 (t, J = 7.1 Hz, 3H).

^{13}C NMR (100 MHz, $CDCl_3$): δ 173.5, 163.1, 152.2, 148.4, 138.8, 137.2, 136.9, 134.9, 129.6, 129.3, 129.1, 128.6, 127.8, 127.7, 127.2, 126.8, 118.2, 112.7, 60.4, 49.7, 45.1, 34.2, 27.1, 25.0, 14.3.

HRMS (ESI-TOF): calc’d for $C_{32}H_{33}N_2O_4^+$ [M+H$^+$] 509.2435, found 509.2423.

Supplementary Note 8: General *ortho*-arylation procedure for the synthesis of **4A-A'**

A 4 mL oven-dried Schlenk tube equipped with a magnetic stir bar was charged with alkenyl iodide **1** (0.1 mmol, 1.0 equiv) and potassium carbonate (0.25 mmol, 2.5 equiv) and anhydrous DME (0.8 mL) in the glove box. Then 0.2 mL of DME solution with palladium acetate (0.001 mmol), norbornene derivatives **N**[9] (0.05 mmol, 0.5 equiv), aryl bromide **2** (0.15 mmol, 1.5 equiv) and olefin **3** (0.15 mmol, 1.5 equiv) were added, and the mixture was heated to 105 °C and stirred for 24 h. After completion of the reaction (monitored by TLC), the mixture was cooled to r.t., filtered through a thin pad of Celite, eluting with EtOAc (10 mL), and the combined filtrate was concentrated *in vacuo*. The residue was directly purified by column chromatography on silica gel or

purified by PTLC (preparative thin layer chromatography) to give the desired product **4A-A'**.

Methyl (*E*)-2-(1-benzyl-5-methyl-2-oxo-4-styryl-1,2-dihydropyridin-3-yl)benzoate (**4A**)

Physical state: yellow oil;

Yield: 93%;

R_f = 0.3 (silica gel, PE: EtOAc = 2:1);

^{1}H NMR (400 MHz, $CDCl_3$): δ 7.99 (dd, *J* = 7.8, 1.4 Hz, 1H), 7.46 (td, *J* = 7.5, 1.4 Hz, 1H), 7.39 – 7.08 (m, 13H), 6.62 (d, *J* = 16.6 Hz, 1H), 6.42 (d, *J* = 16.7 Hz, 1H), 5.24 (d, *J* = 14.5 Hz, 1H), 5.06 (d, *J* = 14.5 Hz, 1H), 3.71 (s, 3H), 2.15 (s, 3H).

^{13}C NMR (100 MHz, $CDCl_3$): δ 167.7, 161.3, 145.3, 138.4, 137.1, 136.8, 133.7, 132.1, 131.5, 131.4, 130.2, 129.0 (2C), 128.9, 128.7, 128.3 (2C), 127.9, 127.5, 126.6, 124.7, 114.5, 52.2, 52.1, 17.7.

HRMS (ESI-TOF): calc'd for $C_{29}H_{25}NNaO_3^+$ [M+Na$^+$] 458.1727, found 458.1729.

Methyl (*E*)-2-(1,5-dimethyl-2-oxo-4-styryl-1,2-dihydropyridin-3-yl)benzoate (**4B**)

Physical state: yellow oil;

Yield: 63%;

R_f = 0.3 (silica gel, PE: EtOAc = 1:2);

^{1}H NMR (400 MHz, $CDCl_3$): δ 8.00 (dd, *J* = 7.9, 1.4 Hz, 1H), 7.47 (td, *J* = 7.5, 1.5 Hz, 1H), 7.35 (td, *J* = 7.6, 1.3 Hz, 1H), 7.29 – 7.08 (m, 7H), 6.62 (d, *J* = 16.6 Hz, 1H), 6.41 (d, *J* = 16.6 Hz, 1H), 3.76 (s, 3H), 3.54 (s, 3H), 2.18 (s, 3H).

^{13}C NMR (100 MHz, $CDCl_3$): δ 167.5, 161.5, 145.4, 138.6, 136.9, 136.8, 134.9, 132.2, 132.1, 131.3, 131.0, 130.2, 128.7, 128.3, 127.5, 126.6, 124.7, 114.0, 52.1, 37.6, 17.6.

HRMS (ESI-TOF): calc'd for $C_{23}H_{21}NNaO_3^+$ [M+Na$^+$] 382.1414, found 382.1418.

Methyl (*E*)-2-(5-methyl-2-oxo-4-styryl-1-(2,4,6-trimethylbenzyl)-1,2-dihydropyridin-3-yl)benzoate (**4C**)

Physical state: yellow oil;

Yield: 69%;

R_f = 0.3 (silica gel, PE: EtOAc = 2:1);

^{1}H NMR (400 MHz, $CDCl_3$): δ 8.01 (d, J = 7.8 Hz, 1H), 7.48 (td, J = 7.5, 1.4 Hz, 1H), 7.35 (td, J = 7.6, 1.3 Hz, 1H), 7.29 – 7.10 (m, 6H), 6.95 (s, 2H), 6.62 (d, J = 16.6 Hz, 1H), 6.59 – 6.57 (s, 1H), 6.41 (d, J = 16.6 Hz, 1H), 5.25 (d, J = 15.2 Hz, 1H), 5.03 (d, J = 15.2 Hz, 1H), 3.77 (s, 3H), 2.32 (s, 3H), 2.27 (s, 6H), 2.03 (s, 3H).

^{13}C NMR (100 MHz, $CDCl_3$): δ 167.6, 161.5, 144.9, 138.6, 138.4, 137.0, 136.9, 132.2, 132.1, 131.5, 130.9, 130.2, 129.6, 128.8, 128.7, 128.2, 127.5, 126.6, 124.7, 114.2, 52.1, 45.1, 21.2, 19.9, 17.9.

HRMS (ESI-TOF): calc'd for $C_{32}H_{31}NNaO_3^+$ [M+Na$^+$] 500.2196, found 500.2199.

Methyl (*E*)-2-(1-(methoxymethyl)-5-methyl-2-oxo-4-styryl-1,2-dihydropyridin-3-yl)benzoate (**4D**)

Physical state: yellow oil;

Yield: 88%;

R_f = 0.5 (silica gel, PE: EtOAc = 2:1);

^{1}H NMR (400 MHz, $CDCl_3$): δ 8.00 (d, J = 7.8 Hz, 1H), 7.47 (t, J = 7.5 Hz, 1H), 7.34 (t, J = 7.7 Hz, 1H), 7.28–7.10 (m, 7H), 6.62 (d, J = 16.6 Hz, 1H), 6.43 (d, J = 16.6 Hz, 1H), 5.36 (d, J = 9.9 Hz, 1H), 5.29 (d, J = 9.7 Hz, 1H), 3.75 (s, 3H), 3.41 (s, 3H), 2.19 (s, 3H);

^{13}C NMR (100 MHz, $CDCl_3$): δ 167.5, 161.8, 145.9, 138.3, 137.3, 136.7, 132.4, 132.2, 132.1, 131.3, 130.2, 128.7, 128.4, 127.6, 126.7, 124.5, 114.6, 78.2, 57.2, 52.1, 17.7.

HRMS (ESI-TOF): calc'd for $C_{24}H_{24}NO_4^+$ [M+H$^+$] 390.1699, found 390.1697.

Methyl (*E*)-2-(1-benzyl-5-fluoro-2-oxo-4-styryl-1,2-dihydropyridin-3-yl)benzoate (**4E**)

Physical state: yellow oil;

Yield: 34%;

R_f = 0.3 (silica gel, PE: EtOAc = 2:1);

^{1}H NMR (400 MHz, $CDCl_3$): δ 8.10 (d, J = 7.9 Hz, 1H), 7.58 (td, J = 7.5, 1.4 Hz, 1H), 7.47 (td, J = 7.7, 1.4 Hz, 1H), 7.37 – 7.35 (m, 4H), 7.28 – 7.15 (m, 9H), 6.50 (d, J = 16.7 Hz, 1H), 5.19 (d, J = 14.5 Hz, 1H), 5.05 (d, J = 14.5 Hz, 1H), 3.70 (s, 3H).
^{13}C NMR (100 MHz, $CDCl_3$): δ 167.1, 160.1, 146.9 (d, J = 234.7 Hz), 138.4 (d, J = 12.4 Hz), 136.8 (d, J = 2.7 Hz), 136.8, 136.3, 135.5 (d, J = 14.5 Hz), 132.4, 132.3 (d, J = 4.4 Hz), 132.0, 131.3, 130.7, 129.1, 128.9, 128.8, 128.5, 128.4, 128.3, 127.1, 120.7 (d, J = 40.0 Hz), 120.4 (d, J = 2.9 Hz), 52.3, 52.2.
^{19}F NMR (376 MHz, $CDCl_3$): δ -149.8.
HRMS (ESI-TOF): calc'd for $C_{28}H_{22}FNNaO_3^+$ [M+Na$^+$] 462.1476, found 462.1479.

Methyl (*E*)-2-(1-benzyl-5-chloro-2-oxo-4-styryl-1,2-dihydropyridin-3-yl)benzoate (**4F**)
Physical state: yellow oil;
Yield: 48%;
R_f = 0.3 (silica gel, PE: EtOAc = 1:1);
^{1}H NMR (400 MHz, $CDCl_3$): δ 8.04 (dd, J = 7.8, 1.4 Hz, 1H), 7.49 (td, J = 7.5, 1.4 Hz, 1H), 7.43 – 7.28 (m, 7H), 7.28 – 7.12 (m, 6H), 6.70 (d, J = 16.6 Hz, 1H), 6.57 (d, J = 16.6 Hz, 1H), 5.20 (d, J = 14.5 Hz, 1H), 5.06 (d, J = 14.5 Hz, 1H), 3.72 (s, 3H).
^{13}C NMR (100 MHz, $CDCl_3$): δ 167.4, 160.6, 142.6, 139.0, 137.7, 136.5, 136.2, 133.5, 132.5, 131.7, 131.4, 130.4, 129.1, 128.7, (2C), 128.5, 128.3, 128.0, 126.9, 122.6, 113.1, 52.5, 52.2.
HRMS (ESI-TOF): calc'd for $C_{28}H_{22}ClNNaO_3^+$ [M+Na$^+$] 478.1180, found 478.1182.

Methyl (*E*)-2-(1-benzyl-5-(methoxymethyl)-2-oxo-4-styryl-1,2-dihydropyridin-3-yl)benzoate (**4G**)
Physical state: yellow oil;
Yield: 87%;
R_f = 0.2 (silica gel, PE: EtOAc = 2:1);
^{1}H NMR (400 MHz, $CDCl_3$): δ 8.01 (d, J = 7.9 Hz, 1H), 7.48 – 7.44 (m, 1H), 7.40 – 7.14 (m, 13H), 6.67 (d, J = 4.3 Hz, 2H), 5.29 (d, J = 14.5 Hz, 1H), 5.03 (d, J = 14.5 Hz, 1H), 4.33 (d, J = 11.1 Hz, 1H), 4.13 (d, J = 11.1 Hz, 1H), 3.70 (s, 3H), 3.41 (s, 3H).

^{13}C NMR (100 MHz, $CDCl_3$): δ 167.6, 161.5, 145.1, 138.0, 137.3, 136.9, 136.8, 136.3, 132.3, 132.2, 131.7, 131.4, 130.2, 128.9, 128.7, 128.4, 128.3, 128.0, 127.7, 126.8, 123.9, 114.9, 71.2, 58.0, 52.4, 52.1.

HRMS (ESI-TOF): calc'd for $C_{30}H_{27}NNaO_4^+$ [M+Na$^+$] 488.1832, found 488.1833.

Methyl (*E*)-2-(1-benzyl-5-methyl-6-oxo-4-styryl-1,6-dihydropyridin-3-yl)benzoate (**4H**)

Physical state: white solid;

Melting point: 145–150 ºC;

Yield: 94%;

R_f = 0.3 (silica gel, PE: EtOAc = 2:1);

^1H NMR (400 MHz, $CDCl_3$): δ 7.84 (dd, J = 7.8, 1.4 Hz, 1H), 7.49 – 7.44 (m, 1H), 7.50 – 7.44 (m, 5H), 7.31 – 7.19 (m, 5H), 7.17 – 7.13 (m, 2H), 7.03 (s, 1H), 6.60 (d, J = 16.6 Hz, 1H), 6.32 (d, J = 16.6 Hz, 1H), 5.20 (s, 2H), 3.59 (s, 3H), 2.34 (s, 3H).

^{13}C NMR (100 MHz, $CDCl_3$): δ 167.7, 162.6, 145.1, 138.2, 137.1, 136.9, 136.7, 132.3, 132.1, 131.8, 131.7, 130.3, 128.9, 128.7, 128.3, 128.1, 127.9, 127.8, 126.6, 126.2, 124.5, 120.7, 52.5, 52.1, 14.8.

HRMS (ESI-TOF): calc'd for $C_{29}H_{25}NNaO_3^+$ [M+Na$^+$] 458.1727, found 458.1725.

Dimethyl 2,2'-(1-benzyl-2-oxo-4-styryl-1,2-dihydropyridine-3,5-diyl)(E)-dibenzoate (**4I**)

Physical state: yellow solid;

Melting point: 215 – 218 ºC;

Yield: 69%;

R_f = 0.3 (silica gel, PE:EtOAc = 1:1);

This compound exists as a mixture of rotamers (the ratio is about 1:1) at room temperature. However, it becomes a single compound at elevated temperature. So, the NMR experiments were performed at 110 ºC in DMSO-d_6 to obtain the simplified NMR spectra.

^{1}H NMR (400 MHz, DMSO-d_6, 110 ºC): δ 7.88 (dd, J = 7.8, 1.3 Hz, 1H), 7.81 (d, J = 6.9 Hz, 1H), 7.64 – 7.58 (m, 1H), 7.57 – 7.51 (m, 2H), 7.48 (td, J = 7.6, 1.3 Hz, 1H), 7.42 (td, J = 7.6, 1.2 Hz, 2H), 7.38 – 7.33 (m, 4H), 7.32 – 7.26 (m, 1H), 7.24 (d, J = 7.9 Hz, 1H), 7.18 – 7.11 (m, 3H), 6.76 (s, 2H), 6.26 (d, J = 16.6 Hz, 1H), 5.95 (d, J = 16.6 Hz, 1H), 5.27 (s, 1H), 5.10 (s, 1H), 3.64 (s, 3H), 3.63 (s, 3H);

^{13}C NMR (100 MHz, DMSO-d_6, 110 ºC): δ 167.5, 160.6, 140.0, 136.8 (2C), 135.5, 132.5, 132.4, 132.0, 129.9, 129.7, 128.8, 128.7, 128.7, 128.3, 128.1, 128.0, 127.6, 126.2, 125.0, 52.0, 51.8 (2C);

HRMS (ESI-TOF): calc'd for $C_{36}H_{29}NNaO_5^+$ [M+Na$^+$] 578.1938, found 578.1939.

Benzyl (*E*)-2-(1-benzyl-5-methyl-2-oxo-4-styryl-1,2-dihydropyridin-3-yl)benzoate (**4J**)

Physical state: yellow oil;

Yield: 87%;

R_f = 0.4 (silica gel, PE: EtOAc = 1:1);

^{1}H NMR (400 MHz, $CDCl_3$): δ 8.05 (dd, J = 7.9, 1.3 Hz, 1H), 7.47 (td, J = 7.5, 1.5 Hz, 1H), 7.37-7.30 (m, 5Hz), 7.29 – 7.25 (m, 4H), 7.23 – 7.18 (m, 6H), 7.13 – 7.09 (m, 2H), 6.96 (s, 1H), 6.55 (d, J = 16.6 Hz, 1H), 6.34 (d, J = 16.6 Hz, 1H), 5.24 – 4.96 (m, 4H), 2.05 (s, 3H).

^{13}C NMR (100 MHz, $CDCl_3$): δ 167.1, 161.2, 145.3, 138.3, 137.0, 136.8, 136.1, 133.5, 132.3, 132.1, 131.4 (2C), 130.6, 128.9, 128.7, 128.4, 128.3 (2C), 128.0, 127.9, 127.6, 126.6, 124.5, 114.3, 66.7, 52.2, 17.6.

HRMS (ESI-TOF): calc'd for $C_{35}H_{29}NNaO_3^+$ [M+Na$^+$] 534.2040, found 534.2042.

(*E*)-2-(1-benzyl-5-methyl-2-oxo-4-styryl-1,2-dihydropyridin-3-yl) benzoic acid (**4K**)

Physical state: yellow oil;

Yield: 52%;

R_f = 0.2 (silica gel, PE: EtOAc = 1:2);

^{1}H NMR (400 MHz, $CDCl_3$): δ 7.92 (d, *J* = 7.7 Hz, 1H), 7.42 (td, *J* = 7.5, 1.5 Hz, 1H), 7.39 – 7.07 (m, 13H), 6.60 (d, *J* = 16.7 Hz, 1H), 6.42 (d, *J* = 16.6 Hz, 1H), 5.22 (s, 2H), 2.16 (s, 3H).

^{13}C NMR (100 MHz, $CDCl_3$): δ 170.8, 162.3, 148.3, 138.5, 136.5, 136.4, 134.3, 132.0, 131.9, 130.4, 130.3, 129.1, 128.7, 128.6, 128.3, 128.2, 128.0, 126.8, 123.9, 116.5, 52.6, 17.6.

HRMS (ESI-TOF): calc'd for $C_{28}H_{23}NNaO_3^+$ [M+Na$^+$] 444.1570, found 444.1573.

(*E*)-2-(1-benzyl-5-methyl-2-oxo-4-styryl-1,2-dihydropyridin-3-yl)-N,N-dimethylbenzamide (**4L**)

Physical state: white solid;

Melting point: 130 – 135 ºC;

Yield: 72%;

R_f = 0.2 (silica gel, PE: EtOAc = 1:1);

^{1}H NMR (400 MHz, $CDCl_3$): δ 7.3 – 7.31(m, 2H), 7.30 – 7.24 (m, 6H), 7.23-7.15 (m, 6H), 7.07 (s, 1H), 6.70 (s, 2H), 5.31 (d, *J* = 14.6 Hz, 1H), 4.89 (d, *J* = 14.6 Hz, 1H), 2.96 (s, 3H), 2.90 (s, 3H), 2.11 (s, 3H).

^{13}C NMR (100 MHz, $CDCl_3$): δ 171.4, 161.1, 148.0, 137.5, 137.2, 137.1, 135.8, 134.1, 131.9, 129.4, 128.9, 128.8, 128.6, 128.1, 128.0, 127.8, 126.8 (2C), 126.3, 124.8, 114.7, 52.3, 39.7, 34.9, 17.6.

HRMS (ESI-TOF): calc'd for $C_{30}H_{28}N_2NaO_2^+$ [M+Na$^+$] 471.2043, found 471.2049.

(*E*)-2-(1-benzyl-5-methyl-2-oxo-4-styryl-1,2-dihydropyridin-3-yl)-N-methoxy-N-methylbenzamide (**4M**)

Physical state: white solid;

Melting point: 130 – 135 ºC;

Yield: 87%;

R_f = 0.2 (silica gel, PE: EtOAc = 1:1);

^{1}H NMR (400 MHz, $CDCl_3$): δ 7.43 (s, 1H), 7.36-7.27 (m, 7), 7.23 – 7.16 (m, 6H), 7.08 (s, 1H), 6.69 (d, J = 16.6 Hz, 1H), 6.58 (d, J = 16.5 Hz, 1H), 5.19 (d, J = 14.6 Hz, 1H), 5.03 (d, J = 14.5 Hz, 1H), 3.57 (s, 3H), 3.18 (s, 3H), 2.11 (s, 3H).

^{13}C NMR (100 MHz, $CDCl_3$): δ 161.1, 137.4, 137.0 (2C), 134.0, 132.1, 129.6, 128.8, 128.6, 128.1(2C), 127.8, 126.8, 124.9, 114.6, 60.5, 52.3, 17.6.

HRMS (ESI-TOF): calc'd for $C_{30}H_{28}N_2NaO_3^+$ [M+Na$^+$] 487.1992, found 487.1998.

(*E*)-3-(2-acetylphenyl)-1-benzyl-5-methyl-4-styrylpyridin-2(1*H*)-one (**4N**)

Physical state: colorless oil;

Yield: 42%;

R_f = 0.3 (silica gel, PE: EtOAc = 2:1);

^{1}H NMR (400 MHz, $CDCl_3$): δ 7.70 (d, J = 7.6 Hz, 1H), 7.47 –7.27 (m, 6H), 7.26-7.15 (m, 7H), 7.07 (s, 1H), 6.64 (d, J = 16.5 Hz, 1H), 6.47 (d, J = 16.6 Hz, 1H), 5.14 (s, 2H), 2.52 (s, 2H), 2.13 (s, 3H).

^{13}C NMR (100 MHz, $CDCl_3$): δ 201.4, 161.1, 146.1, 140.5, 137.4, 137.0, 136.9, 136.02, 133.7, 132.7, 131.2, 130.9, 128.9, 128.7, 128.3, 128.1, 127.9, 127.8, 127.4, 126.7, 124.8, 114.7, 52.1, 28.5, 17.6.

HRMS (ESI-TOF): calc'd for $C_{29}H_{25}NNaO_2^+$ [M+Na$^+$] 442.1778, found 442.1785.

Methyl (*E*)-2-(1-benzyl-5-methyl-2-oxo-4-styryl-1,2-dihydropyridin-3-yl)-4-chlorobenzoate (**4O**)

Physical state: yellow solid;

Melting point: 140 – 145 °C;

Yield: 82%;

R_f = 0.4 (silica gel, PE: EtOAc = 2:1);

^{1}H NMR (400 MHz, $CDCl_3$): δ 7.93 (d, J = 8.4 Hz, 1H), 7.44 – 7.123 (m, 10H), 7.21 – 7.17 (m, 3H), 7.11 (s, 1H), 6.63 (d, J = 16.5 Hz, 1H), 6.44 (d, J = 16.6 Hz, 1H), 5.23 (d, J = 14.5 Hz, 1H), 5.06 (d, J = 14.5 Hz, 1H), 3.71 (s, 3H), 2.13 (s, 3H).

^{13}C NMR (100 MHz, $CDCl_3$): δ 166.8, 161.0, 145.8, 140.4, 138.2, 137.7, 136.9, 136.6, 134.0, 132.1, 131.6, 130.1, 130.0, 128.9, 128.8, 128.5, 128.2, 128.0, 127.7, 126.7, 124.2, 114.6, 52.3, 17.5.

HRMS (ESI-TOF): calc'd for $C_{29}H_{24}ClNNaO_3^+$ [M+Na$^+$] 492.1337, found 492.1343.

Methyl (*E*)-2-(1-benzyl-5-methyl-2-oxo-4-styryl-1,2-dihydropyridin-3-yl)-5-methylbenzoate (**4P**)

Physical state: yellow solid;

Melting point: 190 – 195 ºC;

Yield: 72%;

R_f = 0.3 (silica gel, PE: EtOAc = 2:1);

^1H NMR (400 MHz, $CDCl_3$): δ 7.81 (s, 1H), 7.35 – 7.32 (m, 4H), 7.30 – 7.25 (m, 3H), 7.24 – 7.16 (m, 4H), 7.07 (d, *J* = 7.8 Hz, 2H), 6.64 (d, *J* = 16.6 Hz, 1H), 6.46 (d, *J* = 16.7 Hz, 1H), 5.21 (d, *J* = 14.5 Hz, 1H), 5.06 (d, *J* = 14.5 Hz, 1H), 3.70 (s, 3H), 2.36 (s, 3H), 2.14 (s, 3H).

^{13}C NMR (100 MHz, $CDCl_3$): δ 167.8, 161.4, 145.1, 137.2 (2C), 136.9, 136.8, 135.4, 133.5, 132.9, 132.0, 131.5, 131.2, 130.7, 128.8, 128.7, 128.3, 128.2, 127.8, 126.7, 124.9, 114.3, 52.1, 52.0, 21.2, 17.8.

HRMS (ESI-TOF): calc'd for $C_{30}H_{27}NNaO_3^+$ [M+Na$^+$] 472.1883, found 472.1885.

Methyl (*E*)-2-(1-benzyl-5-methyl-2-oxo-4-styryl-1,2-dihydropyridin-3-yl)-5-fluorobenzoate (**4Q**)

Physical state: yellow solid;

Melting point: 185 – 190 ºC;

Yield: 52%;

R_f = 0.4 (silica gel, PE: EtOAc = 2:1);

^1H NMR (400 MHz, $CDCl_3$): δ 7.68 (dt, *J* = 9.3, 1.6 Hz, 1H), 7.35 (d, *J* = 4.4 Hz, 4H), 7.32 – 7.22 (m, 4H), 7.17 (td, *J* = 7.3, 6.8, 1.8 Hz, 4H), 7.11 (s, 1H), 6.62 (d, *J* = 16.6 Hz, 1H), 6.43 (d, *J* = 16.7 Hz, 1H), 5.22 (d, *J* = 14.5 Hz, 1H), 5.05 (d, *J* = 14.5 Hz, 1H), 3.72 (s, 3H), 2.14 (s, 3H).

^{13}C NMR (100 MHz, $CDCl_3$): δ 166.6 (d, *J* = 2.7 Hz), 162.8, 161.3, 160.4, 145.8, 137.4, 136.8 (d, *J* = 33.4 Hz), 134.3 (d, *J* = 3.6 Hz), 134.0 (d, *J* = 7.6 Hz), 133.9, 133.4 (d, *J* = 7.9 Hz), 130.2, 128.9, 128.8, 128.5, 128.2, 127.9, 126.6, 124.5, 119.2 (d, *J* = 21.0 Hz), 117.1 (d, *J* = 23.3 Hz), 114.6, 52.4, 52.3, 17.6.

^{19}F NMR (376 MHz, CDCl3): δ -114.2.

HRMS (ESI-TOF): calc'd for $C_{29}H_{24}FNNaO_3^+$ [M+Na$^+$] 476.1632, found 476.1633.

Methyl (*E*)-2-(1-benzyl-5-methyl-2-oxo-4-styryl-1,2-dihydropyridin-3-yl)-5-nitrobenzoate (**4R**)

Physical state: yellow solid;

Melting point: 220–225 ºC;

Yield: 89%;

R_f = 0.3 (silica gel, PE: EtOAc = 1:1);

^1H NMR (400 MHz, $CDCl_3$): δ 8.80 (d, *J* = 2.4 Hz, 1H), 8.26 (dd, *J* = 8.5, 2.5 Hz, 1H), 7.39 – 7.27 (m, 8H), 7.25 – 7.22 (m, 2H), 7.18 – 7.16 (m, 3H), 6.66 (d, *J* = 16.5 Hz, 1H), 6.42 (d, *J* = 16.5 Hz, 1H), 5.23 (d, *J* = 14.5 Hz, 1H), 5.04 (d, *J* = 14.5 Hz, 1H), 3.80 (s, 3H), 2.15 (s, 3H).

^{13}C NMR (100 MHz, $CDCl_3$): δ 166.0, 160.6, 146.8, 146.6, 145.3, 138.6, 136.6, 136.1, 134.7, 133.7 (2C), 129.0 (2C), 128.9, 128.2, 128.1, 126.7, 126.2, 125.1, 123.8, 114.8, 52.8, 52.4, 17.4.

HRMS (ESI-TOF): calc'd for $C_{29}H_{24}N_2NaO_5^+$ [M+Na$^+$] 503.1577, found 503.1578.

(*E*)-1-benzyl-3-(4-methoxy-2-nitrophenyl)-5-methyl-4-styrylpyridin-2(1*H*)-one (**4S**)

Physical state: yellow solid;

Melting point: 220 – 225 ºC;

Yield: 35%;

R_f = 0.3 (silica gel, PE: EtOAc = 2:1);

^1H NMR (400 MHz, $CDCl_3$): δ 7.60 (d, *J* = 2.6 Hz, 1H), 7.38 – 7.26 (m, 8H), 7.24-7.20 (m, 2H), 7.18 (d, *J* = 8.0 Hz, 1H) ,7.10 – 7.05 (m, 2H), 6.69 (d, *J* = 16.5 Hz, 1H), 6.50 (d, *J* = 16.6 Hz, 1H), 5.19 – 5.10 (m, 2H), 3.86 (s, 3H), 2.13 (s, 3H).

^{13}C NMR (100 MHz, $CDCl_3$): δ 160.8, 159.3, 150.5, 146.4, 137.7, 136.8, 136.5, 134.3, 134.1, 129.0, 128.8, 128.6, 128.3, 128.1, 127.7, 126.8, 124.5, 124.2, 119.8, 114.7, 109.2, 56.0, 52.1, 17.5.

HRMS (ESI-TOF): calc'd for $C_{28}H_{24}N_2NaO_4^+$ [M+Na$^+$] 475.1628, found 475.1633.

Methyl (*E*)-2-(1-benzyl-4-(3-methoxy-3-oxoprop-1-en-1-yl)-5-methyl-2-oxo-1,2-dihydropyridin-3-yl)benzoate (**4T**)

Physical state: yellow oil;

Yield: 81%;

R_f = 0.3 (silica gel, PE: EtOAc = 2:1);

^1H NMR (400 MHz, $CDCl_3$): δ 8.03 – 7.09 (m, 1H), 7.50 (td, J = 7.5, 1.4 Hz, 1H), 7.39 (t, J = 8.0 Hz, 1H), 7.34 – 7.27 (m, 6H), 7.12 – 7.06 (m, 2H), 5.77 (d, J = 16.3 Hz, 1H), 5.31 – 4.90 (m, 2H), 3.71 (s, 3H), 3.66 (s, 3H), 2.08 (s, 3H).

^{13}C NMR (100 MHz, $CDCl_3$): δ 167.4, 166.3, 161.0, 142.6, 141.0, 137.3, 136.7, 133.9, 132.4, 131.7, 131.3, 130.4, 128.9, 128.3, 128.1, 128.0, 126.2, 113.7, 52.4, 52.2, 51.9, 17.3.

HRMS (ESI-TOF): calc'd for $C_{25}H_{23}NNaO_5^+$ [M+Na$^+$] 440.1468, found 440.1470.

Methyl (*E*)-2-(1-benzyl-4-(3-(tert-butoxy)-3-oxoprop-1-en-1-yl)-5-methyl-2-oxo-1,2-dihydropyridin-3-yl)benzoate (**4U**)

Physical state: yellow oil;

Yield: 69%;

R_f = 0.4 (silica gel, PE: EtOAc = 2:1);

^1H NMR (400 MHz, $CDCl_3$): δ 8.03 – 7.09 (m, 1H), 7.50 (td, J = 7.6, 1.4 Hz, 1H), 7.39 (td, J = 7.7, 1.3 Hz, 1H), 7.36 – 7.27 (m, 5H), 7.18 (d, J = 16.4 Hz, 1H), 7.13 – 7.07 (m, 2H), 5.72 (d, J = 16.4 Hz, 1H), 5.26 – 4.93 (m, 2H), 3.70 (s, 3H), 2.09 (s, 3H), 1.39 (s, 9H).

^{13}C NMR (100 MHz, $CDCl_3$): δ 167.4, 165.2, 161.0, 142.8, 139.6, 137.4, 136.8, 133.9, 133.0, 132.2, 131.9, 131.2, 130.3, 128.9, 128.3 (2C), 128.0, 113.8, 81.0, 52.3, 52.1, 28.1, 17.5.

HRMS (ESI-TOF): calc'd for $C_{28}H_{29}NNaO_5^+$ [M+Na$^+$] 482.1938, found 482.1932.

Methyl (*E*)-2-(1-benzyl-4-(2-(diethoxyphosphoryl)vinyl)-5-methyl-2-oxo-1,2-dihydropyridin-3-yl)benzoate (**4V**)

Physical state: colorless oil;

Yield: 67%;

R_f = 0.3 (silica gel, PE: EtOAc = 1:2);

^{1}H NMR (400 MHz, $CDCl_3$): δ 7.98 (d, *J* = 6.5 Hz, 1H), 7.48 (td, *J* = 7.6, 1.4 Hz, 1H), 7.41 – 7.25 (m, 6H), 7.18 (dd, *J* = 23.2, 17.7 Hz, 1H), 7.10 (d, *J* = 8.1 Hz, 2H), 5.62 (dd, *J* = 19.5, 17.7 Hz, 1H), 5.25 – 4.96 (m, 2H), 3.96 – 3.80 (m, 2H), 3.79 – 3.67 (m, 4H), 3.63 – 3.48 (m, 1H), 2.04 (s, 3H), 1.20 (t, *J* = 7.1 Hz, 3H), 1.11 (t, *J* = 7.0 Hz, 3H).

^{13}C NMR (100 MHz, $CDCl_3$): δ 167.3, 160.9, 145.4 (d, *J* = 5.9 Hz), 144.1 (d, *J* = 22.5 Hz), 137.7, 136.7, 133.8, 132.3, 132.1, 131.7, 131.5, 130.2, 128.9, 128.3, 127.9 (d, *J* = 20.3 Hz), 124.3 (d, *J* = 182.3 Hz), 113.4, 61.9 (t, *J* = 5.3 Hz), 52.3, 52.2, 17.0, 16.3 (t, *J* = 6.0 Hz).

^{31}P NMR (162 MHz, $CDCl_3$): δ 16.3.

HRMS (ESI-TOF): calc'd for $C_{27}H_{30}NNaO_6P^+$ [M+Na$^+$] 518.1703, found 518.1712.

Methyl (*E*)-2-(1-benzyl-5-methyl-4-(2-(naphthalen-2-yl)vinyl)-2-oxo-1,2-dihydropyridin-3-yl)benzoate (**4W**)

Physical state: yellow oil;

Yield: 89%;

R_f = 0.2 (silica gel, PE: EtOAc = 2:1);

^{1}H NMR (400 MHz, $CDCl_3$): δ 8.02 (d, *J* = 7.9 Hz, 1H), 7.78 – 7.67 (m, 3H), 7.52 – 7.42 (m, 4H), 7.39 – 7.22 (m, 7H), 7.28 (m, 11H), 7.24 (d, *J* = 7.6 Hz, 1H), 7.13 (s, 1H), 6.77 (d, *J* = 16.6 Hz, 1H), 6.60 (d, *J* = 16.6 Hz, 1H), 5.26 (d, *J* = 14.5 Hz, 1H), 5.08 (d, *J* = 14.5 Hz, 1H), 3.74 (s, 3H), 2.20 (s, 3H).

^{13}C NMR (100 MHz, $CDCl_3$): δ 167.7, 161.3, 145.3, 138.4, 137.3, 137.1, 134.2, 133.7, 133.5, 133.3, 132.2, 132.1, 131.6, 131.4, 130.1, 128.9, 128.4, 128.3, 128.1, 127.9, 127.8, 127.5, 127.2, 126.5, 126.4, 124.9, 123.2, 114.4, 52.2, 52.1, 17.7.

HRMS (ESI-TOF): calc'd for $C_{33}H_{27}NNaO_3^+$ [M+Na$^+$] 508.1883, found 508.1887.

Methyl (*E*)-2-(1-benzyl-5-methyl-2-oxo-4-styryl-1,2-dihydropyridin-3-yl)benzoate (**4X**)

Physical state: yellow oil;

Yield: 42%;

R_f = 0.4 (silica gel, PE: EtOAc = 2:1);

^{1}H NMR (400 MHz, $CDCl_3$): δ 7.98 – 7.93 (m, 1H), 7.48 – 7.43 (m, 1H), 7.38 – 7.25 (m, 6H), 7.15 – 7.12 (m, 1H), 7.05 (s, 1H), 6.37 (d, *J* = 19.6 Hz, 1H), 5.74 (d, *J* = 19.6 Hz, 1H), 5.24 (d, *J* = 14.5 Hz, 1H), 5.03 (d, *J* = 14.5 Hz, 1H), 3.69 (s, 3H), 2.03 (s, 3H), -0.12 (s, 9H).

^{13}C NMR (100 MHz, $CDCl_3$): δ 169.3, 163.1, 149.6, 142.0, 141.4, 140.1, 138.9, 135.2, 134.0, 133.5, 133.2, 132.5, 131.8, 130.6, 130.0, 129.6, 129.0, 115.8, 101.5, 53.9, 53.8, 18.8.

HRMS (ESI-TOF): calc'd for $C_{26}H_{29}NNaO_3Si^+$ [M+Na$^+$] 454.1809, found 454.1811.

Methyl 2-(1-benzyl-4-(2-(methoxycarbonyl)allyl)-5-methyl-2-oxo-1,2-dihydropyridin-3-yl)benzoate (**4Y**)

Physical state: colorless oil;

Yield: 80%;

R_f = 0.2 (silica gel, PE: EtOAc = 2:1);

^{1}H NMR (400 MHz, $CDCl_3$): δ 8.03 (d, *J* = 7.9 Hz, 1H), 7.55 – 7.46 (m, 1H), 7.42 – 7.28 (m, 6H), 7.15 (d, *J* = 7.6 Hz, 1H), 7.08 (s, 1H), 6.18 (s, 1H), 5.35 (s, 1H), 5.23 (d, *J* = 14.4 Hz, 1H), 5.04 (d, *J* = 14.4 Hz, 1H), 3.69 (s, 3H), 3.67 (s, 3H), 3.37 (d, *J* = 17.5 Hz, 1H), 3.19 (d, *J* = 17.5 Hz, 1H), 1.96 (s, 3H).

^{13}C NMR (100 MHz, $CDCl_3$): δ 167.1, 167.0, 161.3, 145.4, 137.8, 137.2, 137.1, 134.0, 133.6, 132.4, 130.8, 130.7, 130.6, 128.9, 128.4, 127.9 (2C), 126.0, 115.3, 52.2, 52.0, 32.9, 16.1.

HRMS (ESI-TOF): calc'd for $C_{26}H_{25}NNaO_5^+$ [M+Na$^+$] 454.1625, found 454.1620.

Methyl 2-(1-benzyl-5-methyl-4-((*E*)-2-((8*R*,9*S*,13*S*,14*S*)-13-methyl-17-oxo-7,8,9,11,12,13,14,15,16,17-decahydro-6*H*-cyclopenta[a]phenanthren-3-yl)vinyl)-2-oxo-1,2-dihydropyridin-3-yl)benzoate (**4Z**)

Physical state: yellow oil;

Yield: 92%;

R_f = 0.2 (silica gel, PE: EtOAc = 2:1);

^{1}H NMR (400 MHz, $CDCl_3$): δ 8.01 – 7.97 (m, 1H), 7.47 – 7.43 (m, 1H), 7.37 – 7.27 (m, 6H), 7.18 (d, *J* = 8.0 Hz, 2H), 7.09 (s, 1H), 6.97 (d, *J* = 8.2 Hz, 1H), 6.89 (s, 1H), 6.60 (d, *J* = 16.6 Hz, 1H), 6.37 (d, *J* = 16.6 Hz, 1H), 5.24 (d, *J* = 14.5 Hz, 1H), 5.04 (d, *J* = 14.5 Hz, 1H), 3.72 (s, 3H), 2.86 – 2.82 (m, 2H), 2.53 – 2.46 (m, 1H), 2.41 – 2.20 (m, 2H), 2.20 – 1.88 (m, 7H), 1.72 – 1.34 (m, 6H), 0.88 (s, 3H).

^{13}C NMR (100 MHz, $CDCl_3$): δ 167.7, 161.3, 145.4, 140.2, 138.5, 137.1, 136.9, 136.9, 134.4, 133.6, 132.1, 132.1, 131.6, 131.2, 130.1, 128.9, 128.2, 127.9, 127.4 (3C), 125.8, 124.0 (2C), 114.5, 52.2, 52.1, 50.6, 48.1, 44.6, 38.2, 35.9, 31.7, 29.4, 26.5, 25.8, 21.7, 17.7, 13.9.

HRMS (ESI-TOF): calc'd for $C_{41}H_{41}NNaO_4^+$ [M+Na$^+$] 634.2928, found 634.2930.

Methyl (*E*)-2-(1-benzyl-4-(4-(4-((1-isopropoxy-2-methyl-1-oxopropan-2-yl)oxy)benzoyl)styryl)-5-methyl-2-oxo-1,2-dihydropyridin-3-yl)benzoate (**4A'**)

Physical state: yellow oil;

Yield: 70%;

R_f = 0.2 (silica gel, PE: EtOAc = 2:1);

^{1}H NMR (400 MHz, $CDCl_3$): δ 8.02 – 7.98 (m, 1H), 7.75 – 7.61 (m, 4H), 7.50 – 7.46 (m, 1H), 7.40 – 7.27 (m, 6H), 7.25 – 7.18 (m, 3H), 7.12 (s, 1H), 6.87 – 6.81 (m, 2H), 6.75 (d, *J* = 16.6 Hz, 1H), 6.47 (d, *J* = 16.6 Hz, 1H), 5.25 (d, *J* = 14.5 Hz, 1H), 5.13 – 5.04 (m, 2H), 3.73 (s, 3H), 2.16 (s, 3H), 1.65 (s, 6H), 1.21 – 1.18 (m, 6H).

^{13}C NMR (100 MHz, $CDCl_3$): δ 194.9, 173.3, 167.7, 161.2, 159.7, 144.8, 140.3, 138.2, 137.6, 137.0, 136.1, 133.8, 132.14, 132.07, 132.0, 131.7, 131.6, 130.6, 130.3, 130.2, 128.9, 128.2, 127.9, 127.7, 127.0, 126.3, 117.27, 114.2, 79.5, 69.4, 52.2, 52.1, 25.5, 21.6, 17.6.

HRMS (ESI-TOF): calc'd for $C_{43}H_{41}NNaO_7^+$ [M+Na$^+$] 706.2775, found 706.2777.

Supplementary Note 9: Preliminary asymmetric studies.

The reaction was operated in an argon-filled glove box, an oven-dried 4.0 mL vial equipped with a magnetic stir bar was charged with $Pd(OAc)_2$ (0.005 mmol, 5 mol%), TFP (0.01 mmol, 10 mol%), **N⁹** (0.05 mmol, 0.5 equiv), K_2CO_3 (0.25 mmol, 2.5 equiv), alkenyl iodide **1k** (0.1 mmol, 1.0 equiv), bromide **2L** (0.1 mmol, 1.0 equiv), styrene **3a** **(**1.5 equiv**)**, and dry DME (0.5 mL). The vial was sealed with a cap and stirred at r.t. for about 5 min, and then the reaction mixture was heated at 85 °C for 24 h. After the reaction vessel was cooled to r.t., the mixture was filtered and concentrated *in vacuo*. The residue was directly purified by PTLC to yield the desired product **4A*.**

Methyl (*E*)-2-(1-benzyl-6-oxo-4-styryl-1,6-dihydropyridin-3-yl)-3-methylbenzoate (**4A***)

Physical state: pale yellow oil;

Yield: 73%;

HPLC: 97% ee. Daicel Chiralpak IA column, 10% *i*-PrOH in *n*-hexane, 1 mL/min, λ = 220 nm, t_R (major) = 12.784 min, t_R (minor) = 11.860 min;

$[\alpha]_D^{25}$: 127.8 (c 1.0, CH_2Cl_2);

^1H NMR (400 MHz, $CDCl_3$): δ 7.71 (d, *J* = 7.1 Hz, 1H), 7.40 – 7.23 (m, 10H), 7.18 (d, *J* = 7.4 Hz, 2H), 6.89 (s, 1H), 6.71 (d, *J* = 16.5 Hz, 1H), 6.50 (d, *J* = 16.6 Hz, 1H), 5.33 (d, *J* = 14.5 Hz, 1H), 5.12 (d, *J* = 14.6 Hz, 1H), 3.62 (s, 3H), 2.41 (s, 3H), 2.09 (s, 3H);

^{13}C NMR (100 MHz, $CDCl_3$): δ 162.7, 145.3, 138.8, 137.0, 136.9, 136.8, 136.3, 133.5, 132.6, 131.6, 128.9, 128.7, 128.3, 128.0, 127.9 (2C), 127.4, 126.7, 126.5, 124.2, 118.9, 52.3, 52.1, 20.6, 14.8;

HRMS (ESI-TOF): calc'd for $C_{30}H_{27}NNaO_3^+$ [M+Na$^+$] 472.1883, found 472.1883.

Supplementary Note 10: Scale-up experiment

A 100 mL oven-dried Schlenk tube equipped with a magnetic stir bar was charged with palladium acetate (34 mg, 0.15 mmol, 0.05 equiv), norbornene derivatives **N⁸** (227 mg, 1.5 mmol, 0.5 equiv), alkenyl iodide **1a** (1.17 g, 3.6 mmol, 1.2 equiv) and potassium carbonate (1.035 g, 7.5 mmol, 2.5 equiv) and anhydrous 1,4-dioxane (30 mL) in the

glove box. Then alkyl bromide **2a** (878 mg, 4.5 mmol, 1.5 equiv) and olefin **3a** (313 mg, 3.0 mmol, 1.0 equiv) were added, and the mixture was heated to 105 °C and stirred for 36 h. After completion of the reaction (monitored by TLC), the mixture was cooled to r.t., filtered through a thin pad of celite, eluting with EtOAc (10 mL), and the combined filtrate was concentrated *in vacuo*. The residue was directly purified by column chromatography on silica gel to give the desired product **4a** as a yellow solid (1.221 g, 98%). We also recovered mediator $\mathbf{N^8}$ (200 mg, 87% yield).

Supplementary Note 11: Two-component annulation experiment

A 4 mL oven-dried Schlenk tube equipped with a magnetic stir bar was charged with palladium acetate (0.005 mmol), norbornene derivatives $\mathbf{N^8}$ (0.05 mmol, 0.5 equiv), alkenyl iodide **1a** (0.1 mmol, 1.0 equiv) and potassium carbonate (0.25 mmol, 2.5 equiv) and anhydrous 1,4-dioxane (1 mL) in the glove box. Then bifunctional reagent **5** or **6** (0.12 mmol, 1.2 equiv) was added, and the mixture was heated to 105 °C and stirred for 36 h. After completion of the reaction (monitored by TLC), the mixture was cooled to r.t., filtered through a thin pad of celite, eluting with EtOAc (10 mL), and the combined filtrate was concentrated *in vacuo*. The residue was directly purified by column chromatography on silica gel or purified by PTLC to give the desired product **7** or **8**.

Methyl (*Z*)-2-(6-benzyl-8-methyl-5-oxo-3,4,5,6-tetrahydro-1*H*-pyrano[4,3-c]pyridin-1-ylidene)acetate (**7**)

Physical state: yellow oil;

Yield: 90%;

R_f = 0.2 (silica gel, PE: EtOAc = 1:1);

^{1}H NMR (400 MHz, $CDCl_3$): δ 7.38 – 7.28 (m, 5H), 7.08 (s, 1H), 5.37 (s, 1H), 5.13 (s, 2H), 4.21 (t, J = 5.6 Hz, 2H), 3.71 (s, 3H), 2.91 (t, J = 5.6 Hz, 2H), 2.20 (s, 3H).

^{13}C NMR (100 MHz, $CDCl_3$): δ 165.9, 160.1, 157.9, 139.1, 136.1, 135.2, 129.6, 129.1, 128.4, 111.5, 98.6, 65.4, 52.4, 51.3, 23.8, 18.9.

HRMS (ESI-TOF): calc'd for $C_{19}H_{20}NO_4^+$ [M+H$^+$] 326.1387, found 326.1374.

2-(2-Benzyl-4-methyl-1-oxo-1,2,5,6,7,8-hexahydroisoquinolin-5-yl)acetaldehyde (**8**)

Physical state: colorless oil;

Yield: 91%;

R_f = 0.2 (silica gel, PE: EtOAc = 2:1);

^{1}H NMR (400 MHz, $CDCl_3$): δ 9.81 (s, 1H), 7.37 – 7.25 (m, 5H), 6.94 (s, 1H), 5.10 (s, 2H), 3.36 (d, J = 10.5 Hz, 1H), 2.90 – 2.64 (m, 2H), 2.55 – 2.30 (m, 2H), 2.19 – 2.00 (m, 1H), 1.96 (s, 3H), 1.90 – 1.86 (m, 1H), 1.78 – 1.66 (m, 2H).

^{13}C NMR (100 MHz, $CDCl_3$): δ 200.5, 161.9, 148.8, 136.9, 132.0, 128.9, 128.3, 128.0 (2C), 114.0, 51.9, 47.7, 28.6, 25.9, 23.9, 16.6, 15.3.

HRMS (ESI-TOF): calc'd for $C_{19}H_{22}NO_2^+$ [M+H$^+$] 296.1645, found. 296.1635.

Supplementary Note 12: Synthesis procedure of 3,5-Dimethylpyridin-2(1*H*)-one-4-D (**9**)[12]

A 4 mL oven-dried Schlenk tube equipped with a magnetic stir bar was charged with **4l'** (0.1 mmol, 1.0 equiv), $Pd(OH)_2$ (10 mol%) and EtOH (1 mL). Hydrogen balloon was installed and charged gas for three times, and the mixture was heated to 60 °C and stirred for 6 h. After completion of the reaction (monitored by TLC), the mixture was cooled to room temperature, filtered through a thin pad of celite, eluting with EtOH (10 mL), and the combined filtrate was concentrated *in vacuo*. The residue was directly purified by column chromatography on silica gel to give the desired product **9**.

Physical state: whit solid;

Melting point: 80–83 °C;

Yield: 77% (5% H/D exchange);

R_f = 0.4 (silica gel, PE: EtOAc = 1:2);

^{1}H NMR (600 MHz, $CDCl_3$): δ 13.05 (brs, 1H), 7.18 (s, 0.12H), 7.07 (s, 1H), 2.13 (s, 3H), 2.05 (s, 3H);

^{13}C NMR (125 MHz, $CDCl_3$): δ 164.7, 141.9, 141.6 (t, J = 5.0 Hz), 129.6, 128.5, 115.8 (2C), 17.2, 17.1, 17.8, 16.7;

HRMS (ESI-TOF): calc'd for $C_7H_9DNO^+$ [M+H$^+$] 125.0820, found 125.0816.

Supplementary Note 13: Synthesis procedure of **11**[13,14]

A 4 mL oven-dried Schlenk tube equipped with a magnetic stir bar was charged with **4g** (0.2 mmol) and CF_3CO_2H (1 mL). And the mixture was heated to 110 ℃ and stirred for 12 h. After completion of the reaction (monitored by TLC), the mixture was cooled to room temperature, the solvent was removed in *vacuo*. The residue was directly purified by column chromatography on silica gel to give the desired product **10**.

A 4 mL oven-dried Schlenk tube equipped with a magnetic stir bar was charged with **10** (0.1 mmol), pyridine (0.2 mmol, 2.0 equiv) and anhydrous DCM (1 mL). Tf_2O (0.15 mmol, 1.5 equiv) was drop wised and the mixture was stirred another for 0.5 h at room temperature. After completion of the reaction (monitored by TLC), the mixture was filtered through a thin pad of celite, eluting with EtOAc (10 mL), and the combined filtrate was concentrated in *vacuo*. The residue was directly purified by column chromatography on silica gel to give the desired product **11**.

Ethyl (*E*)-4-(5-fluoro-2-oxo-4-styryl-1,2-dihydropyridin-3-yl)butanoate (**10**)

Physical state: white solid;

Melting point: 110–112 ^{o}C;

Yield: 87%;

R_f = 0.4 (silica gel, PE: EtOAc = 1:2);

^{1}H NMR (400 MHz, $CDCl_3$): δ 7.61 (d, *J* = 7.3 Hz, 2H), 7.44 – 7.31 (m, 4H), 7.27 (d, *J* = 5.7 Hz, 2H), 7.12 (d, *J* = 16.6 Hz, 1H), 4.12 (q, *J* = 7.1 Hz, 2H), 2.88–2.76 (m, 2H), 2.43 (t, *J* = 7.0 Hz, 2H), 1.92 (p, *J* = 7.2 Hz, 2H), 1.23 (t, *J* = 7.1 Hz, 3H).

^{13}C NMR (100 MHz, $CDCl_3$): δ 173.6, 163.0, 148.0 (d, *J* = 234.7 Hz), 139.5 (d, *J* = 12.2 Hz), 138.6 (d, *J* = 13.1 Hz), 136.8, 130.2, 129.2, 129.0, 127.4, 118.9, 117.9 (d, J = 38.3 Hz), 60.5, 33.8, 26.5, 23.9, 14.4;

^{19}F (376 MHz, $CDCl_3$): -148.4;

HRMS (ESI-TOF): calc'd for $C_{19}H_{21}FNO_3^+$ [M+H$^+$] 330.1500, found 330.1496.

Ethyl (*E*)-4-(5-fluoro-4-styryl-2-(((trifluoromethyl)sulfonyl)oxy)pyridin-3-

yl)butanoate (**11**)

Physical state: white solid;

Melting point: 97–99 °C;

Yield: 81%;

R_f = 0.7 (silica gel, PE: EtOAc = 2:1);

^{1}H NMR (400 MHz, $CDCl_3$): δ 8.08 (d, *J* = 1.9 Hz, 1H), 7.65 (d, *J* = 7.1 Hz, 2H), 7.47 (d, *J* = 16.6 Hz, 1H), 7.43 (t, *J* = 7.4 Hz, 2H), 7.38 (d, *J* = 7.3 Hz, 1H), 7.16 (d, *J* = 16.6 Hz, 1H), 4.15 (q, *J* = 7.2 Hz, 2H), 2.92 – 2.83 (m, 2H), 2.44 (t, *J* = 6.7 Hz, 2H), 1.96 – 1.88 (m, 2H), 1.25 (t, *J* = 7.2 Hz, 3H);

^{13}C NMR (100 MHz, $CDCl_3$): δ 172.97, 157.18 (d, *J* = 258.4 Hz), 150.97, 141.00 (d, *J* = 13.0 Hz), 136.75 (d, *J* = 10.9 Hz), 136.24, 133.86 (d, *J* = 21 Hz), 129.65, 129.06, 127.60, 127.43, 118.7 (q, *J* = 218.0 Hz), 116.8, 60.8, 33.4, 26.5, 24.4, 14.3;

^{19}F (376 MHz, $CDCl_3$): -72.9, -128.4;

HRMS (ESI-TOF): calc'd for $C_{20}H_{19}F_4NNaO_5S^+$ [M+Na$^+$] 484.0812, found 484.0804.

Supplementary Note 14: Synthesis procedure of **13**[15, 16]

Under Ar atmosphere, a 25 mL oven-dried flask equipped with a magnetic stir bar was charged with **4d** (0.2 mmol) and toluene (1 mL). And the mixture was cooled to -20 ℃ and stirred for 0.5 h. Then BBr_3 (0.5 mmol, 5.0 equiv) was added slowly, and the mixture was stirred for 7 h at -20 ℃. After completion of the reaction (monitored by TLC), the mixture was quenched with H_2O (10 mL) and EtOAc (10 mL), separated organic phase and aqueous extracted with EtOAc for three times. Combined organic phase and concentrated in vacuo. The residue was directly purified by column chromatography on silica gel to give the desired product **12**.

A 10 mL oven-dried flask equipped with a magnetic stir bar was charged with **12** (0.1 mmol), KOH (5.0 equiv.) and mixture solvent (1mL) (THF/MeOH/H_2O 4:1:1). And stirred at 50 °C for 3 h. After completion of the reaction (monitored by TLC), the mixture was quenched with mixture solvent $CHCl_3$ (10 mL) and MeOH (1 mL), separated organic phase and aqueous extracted with mixture solvent DCM (10 mL) and MeOH (1 mL) for three times. Combined organic phase and concentrated in *vacuo*. The

residue was dissolved in anhydrous DCM (1 mL), DCC (1.1 equiv) and DMAP (10 mol%) were added, and the mixture was stirred for 4 h at room temperature under Ar atmosphere. After completion of the reaction (monitored by TLC), the mixture was concentrated in *vacuo*. The residue was directly purified by column chromatography on silica gel to give the desired product **13**.

Ethyl (*E*)-4-(5-methyl-2-oxo-4-styryl-1,2-dihydropyridin-3-yl)butanoate (**12**)

Physical state: white solid;

Melting point: 112–115 °C;

Yield: 85%;

R_f = 0.4 (silica gel, PE: EtOAc = 1:2);

^{1}H NMR (400 MHz, $CDCl_3$): δ 11.91 (brs, 1H), 7.53 (d, *J* = 7.3 Hz, 2H), 7.40 (t, *J* = 7.4 Hz, 2H), 7.32 (t, *J* = 7.3 Hz, 1H), 7.09 (s, 1H), 6.96 (d, *J* = 16.7 Hz, 1H), 6.66 (d, *J* = 16.7 Hz, 1H), 4.02 (q, *J* = 7.2 Hz, 2H), 2.76 – 2.68 (m, 2H), 2.37 (t, *J* = 7.4 Hz, 2H), 2.07 (s, 3H), 1.96 – 1.88 (m, 2H), 1.17 (t, *J* = 7.1 Hz, 3H).

^{13}C NMR (100 MHz, $CDCl_3$): δ 173.8, 164.2, 149.6, 136.6, 135.8, 129.8, 129.5, 128.9, 128.5, 126.8, 124.4, 115.3, 60.3, 34.3, 27.1, 24.1, 17.4, 14.3;

HRMS (ESI-TOF): calc'd for $C_{20}H_{23}NNaO_3^+$ [M+Na$^+$] 348.1570, found 348.1571.

(*E*)-7-methyl-6-styryl-4,5-dihydrooxepino[2,3-b]pyridin-2(3*H*)-one (**13**)

Physical state: white solid;

Melting point: 75–78 °C;

Yield: 51%;

R_f = 0.7 (silica gel, PE: EtOAc = 2:1);

^{1}H NMR (400 MHz, $CDCl_3$): δ 8.10 (s, 1H), 7.51 (d, *J* = 7.3 Hz, 2H), 7.42 (t, *J* = 7.3 Hz, 2H), 7.40 – 7.32 (m, 1H), 7.01 (d, *J* = 16.6 Hz, 1H), 6.66 (d, *J* = 16.6 Hz, 1H), 2.96 (t, *J* = 7.1 Hz, 2H), 2.55 (t, *J* = 7.2 Hz, 2H), 2.32 (s, 3H), 2.24-2.17 (m, 2H);

^{13}C NMR (100 MHz, $CDCl_3$): δ 170.2, 158.1, 148.1, 146.7, 137.0, 136.1, 130.4, 129.1, 129.0, 126.9, 123.0, 122.4, 31.5, 26.6, 24.5, 17.5.

HRMS (ESI-TOF): calc'd for $C_{18}H_{18}NO_2^+$ [M+H$^+$] 280.1332, found 280.1326.

Supplementary Note 15: Synthesis procedure of **17** and **18**[14, 15, 16]

A 4 mL oven-dried Schlenk tube equipped with a magnetic stir bar was charged with palladium acetate (1 mol%), norbornene derivatives **N**[9] (0.05 mmol, 50 mol%), alkenyl iodide **1d** (0.1 mmol, 1.0 equiv) and potassium carbonate (0.25 mmol, 2.5 equiv) and anhydrous DME (1 mL) in the glove box. Then reagent **2G** (0.15 mmol, 1.5 equiv) and **3a** (0.15 mmol, 1.5 equiv) was added, and the mixture was heated to 105 ℃ and stirred for 24 h. After completion of the reaction (monitored by TLC), the mixture was cooled to r.t., filtered through a thin pad of celite, eluting with EtOAc (10 mL), and the combined filtrate was concentrated *in vacuo*. The residue was directly purified by column chromatography on silica gel or purified by pre-TLC to give the desired product **14**.

Under Ar atmosphere, a 25 mL oven-dried flask equipped with a magnetic stir bar was charged with **14** (0.2 mmol) and toluene (1 mL). And the mixture was cooled to -20 ℃ and stirred for 0.5 h. Then BBr_3 (0.5 mmol, 5.0 equiv) was added slowly, and the mixture was stirred for 7 h at -20 ℃. After completion of the reaction (monitored by TLC), the mixture was quenched with H_2O (10 mL) and EtOAc (10 mL), separated organic phase and aqueous extracted with EtOAc (10 mL) for three times. Combined organic phase and concentrated in *vacuo*. The residue was directly purified by column chromatography on silica gel to give the desired product **15** and **16**.

A 4 mL oven-dried Schlenk tube equipped with a magnetic stir bar was charged with **15** (0.1 mmol), pyridine (0.2 mmol, 2.0 equiv) and anhydrous DCM (1 mL). Tf_2O (0.15 mmol, 1.5 equiv) was drop wised and the mixture was stirred another for 0.5 h at room temperature. After completion of the reaction (monitored by TLC), the mixture was filtered through a thin pad of celite, eluting with EtOAc (10 mL), and the combined filtrate was concentrated in vacuo. The residue was directly purified by column chromatography on silica gel to give the desired product **17**.

Under Ar atmosphere, a 10 mL oven-dried flask equipped with a magnetic stir bar was charged with **16** (0.1 mmol), DCC (1.1 equiv) and DMAP (10 mol%). And then anhydrous DCM (1 mL) was added, and the mixture was stirred for 4 h at room

temperature. After completion of the reaction (monitored by TLC), the mixture was concentrated in *vacuo*. The residue was directly purified by column chromatography on silica gel to give the desired product **18**.

Methyl (*E*)-4-chloro-2-(1-(methoxymethyl)-5-methyl-2-oxo-4-styryl-1,2-dihydropyridin-3-yl)benzoate (**14**)
Physical state: yellow solid;
Melting point: 88–91 ^{o}C;
Yield: 84%;
R_f = 0.4 (silica gel, PE: EtOAc = 2:1);
^{1}H NMR (400 MHz, $CDCl_3$): δ 7.95 (d, *J* = 8.4 Hz, 1H), 7.32 (dd, *J* = 8.4, 2.2 Hz, 1H), 7.29 – 7.17 (m, 7H), 6.63 (d, *J* = 16.6 Hz, 1H), 6.45 (d, *J* = 16.6 Hz, 1H), 5.43 – 5.27 (m, 2H), 3.76 (s, 3H), 3.41 (s, 3H), 2.19 (s, 3H);
^{13}C NMR (100 MHz, $CDCl_3$): δ 166.6, 161.5, 146.4, 138.3, 137.9, 136.5, 132.8, 132.0, 131.7, 129.8, 128.8, 128.6, 127.8, 126.7, 124.1, 114.7, 78.2, 57.2, 52.3, 17.6.
HRMS (ESI-TOF): calc'd for $C_{24}H_{23}ClNO_4^+$ [M+H$^+$] 424.1310, found 424.1315.

Methyl (*E*)-4-chloro-2-(5-methyl-2-oxo-4-styryl-1,2-dihydropyridin-3-yl)benzoate (**15**)
Physical state: yellow solid;
Melting point: 85–88 ^{o}C;
Yield: 27%;
R_f = 0.4 (silica gel, PE: EtOAc = 1:2);
^{1}H NMR (400 MHz, $CDCl_3$): δ 12.96 (brs, 1H), 7.87 (d, *J* = 8.4 Hz, 1H), 7.29 – 7.06 (m, 8H), 6.56 (d, *J* = 16.6 Hz, 1H), 6.35 (d, *J* = 16.6 Hz, 1H), 3.66 (s, 3H), 2.07 (s, 3H);
^{13}C NMR (100 MHz, $CDCl_3$): δ 166.9, 163.1, 148.0, 139.7, 138.2, 137.7, 136.6, 132.2, 131.9, 131.7, 130.2, 129.0, 128.8, 128.5, 127.8, 126.7, 124.4, 115.1, 52.3, 17.3;
HRMS (ESI-TOF): calc'd for $C_{22}H_{19}ClNO_3^+$ [M+H$^+$] 380.1048, found 380.1047.

(*E*)-4-chloro-2-(5-methyl-2-oxo-4-styryl-1,2-dihydropyridin-3-yl)benzoic acid (**16**)
Physical state: white solid;

Melting point: 315–317 ^{o}C;

Yield: 42%;

R_f = 0.4 (silica gel, DCM: MeOH = 5:1);

^{1}H NMR (400 MHz, DMSO-d6) δ 11.50 (brs, 1H), 7.84 (d, *J* = 8.2 Hz, 1H), 7.39 (d, *J* = 7.6 Hz, 1H), 7.40 – 7.13 (m, 7H), 7.13 (s, 1H), 6.80 (d, *J* = 16.4 Hz, 1H), 6.37 (d, *J* = 16.4 Hz, 1H), 2.08 (s, 3H).

^{13}C NMR (400 MHz, DMSO-d6): δ 169.0, 161.7, 147.2, 141.1, 137.4. 137.3, 136.3, 133.6, 132.2, 130.6, 130.5, 129.7, 129.3, 129.9, 127.8, 127.3, 125.9, 113.9, 17.3.

HRMS (ESI-TOF): calc'd for $C_{21}H_{15}ClNO_3^-$ [M-H$^+$]$^-$ 364.0735, found 364.0735.

Methyl (*E*)-4-chloro-2-(5-methyl-4-styryl-2-(((trifluoromethyl)sulfonyl)oxy)pyridin-3-yl)benzoate (**17**)

Physical state: white solid;

Melting point: 87–90 ^{o}C;

Yield: 82%;

R_f = 0.7 (silica gel, PE: EtOAc = 2:1);

^{1}H NMR (400 MHz, $CDCl_3$): δ 8.21 (s, 1H), 8.05 (d, *J* = 8.5 Hz, 1H), 7.47 (dd, *J* = 8.5, 2.2 Hz, 1H), 7.32 – 7.23 (m, 4H), 7.23 – 7.17 (m, 2H), 6.69 (d, *J* = 16.7 Hz, 1H), 6.49 (d, *J* = 16.6 Hz, 1H), 3.72 (s, 3H), 2.47 (s, 3H);

^{13}C NMR (100 MHz, $CDCl_3$): δ 165.6, 151.8, 148.0, 147.9, 138.8, 138.6, 136.3, 135.9, 132.3, 131.9, 131.7, 129.1, 128.9, 128.8, 128.6, 126.7, 125.4, 122.6 117.7 (d, *J* = 218.0 Hz), 52.4, 17.7;

^{19}F (376 MHz, $CDCl_3$): -73.9;

HRMS (ESI-TOF): calc'd for $C_{23}H_{18}ClF_3NO_5S^+$ [M+H$^+$] 512.0541, found 512.0542.

(*E*)-9-chloro-2-methyl-1-styryl-6*H*-isochromeno[3,4-b]pyridin-6-one (**18**)

Physical state: white solid;

Melting point: 192–195 ^{o}C;

Yield: 65%;

R_f = 0.7 (silica gel, PE: EtOAc = 2:1);

^{1}H NMR (400 MHz, $CDCl_3$): δ 8.61 (d, *J* = 1.7 Hz, 1H), 8.41 (d, *J* = 8.5 Hz, 1H), 8.38 (s, 1H), 7.60 (d, *J* = 7.4 Hz, 2H), 7.55 (dd, *J* = 8.5, 1.8 Hz, 1H), 7.48 (t, *J* = 7.4 Hz, 2H), 7.41 (t, *J* = 7.3 Hz, 1H), 7.24 (d, *J* = 16.9 Hz, 1H), 6.79 (d, *J* = 16.9 Hz, 1H), 2.44 (s, 3H).

^{13}C NMR (100 MHz, $CDCl_3$): δ 160.3, 156.1, 150.2, 146.2, 141.1, 136.9, 136.1, 135.8, 132.6, 130.2, 129.5, 129.3, 127.8, 126.8, 124.9, 121.0, 110.0, 17.8.

HRMS (ESI-TOF): calc'd for $C_{21}H_{15}ClNO_2^+$ [M+H$^+$] 348.0785, found 348.0786.

Supplementary Note 16: Diversity-oriented functionalization of uracils

A 4 mL oven-dried Schlenk tube equipped with a magnetic stir bar was charged with palladium acetate (0.005 mmol), norbornene derivatives **N^8** (0.05 mmol, 0.5 equiv), alkenyl iodide **1** (0.12 mmol, 1.2 equiv) and potassium carbonate (0.25 mmol, 2.5 equiv) and anhydrous 1,4-dioxane (1 mL) in the glove box. Then MeOTs **2d** (0.15 mmol, 1.5 equiv) and nucleophile **3** (0.1 mmol, 1.0 equiv) were added, and the mixture was heated to 130 °C and stirred for 48 h. After completion of the reaction (monitored by TLC), the mixture was cooled to r.t., filtered through a thin pad of celite, eluting with EtOAc (10 mL), and the combined filtrate was concentrated *in vacuo*. The residue was directly purified by column chromatography on silica gel or purified by PTLC to give the desired product **19-24**.

1,3-Dibenzyl-5-methylpyrimidine-2,4(1*H*,3*H*)-dione-6-*d* (**19**)

Physical state: colorless oil;

Yield: 65%;

R_f = 0.4 (silica gel, PE: EtOAc = 5:1);

^{1}H NMR (400 MHz, $CDCl_3$): δ 7.50 – 7.48 (m, 2H), 7.38 – 7.33 (m, 3H), 7.30 – 7.23 (m, 5H), 6.96 (s, <u>0.2H</u>), 5.17 (s, 2H), 4.90 (s, 2H), 1.89 (s, 3H).

^{13}C NMR (125 MHz, $CDCl_3$): δ 163.8, 152.0, 138.0, 137.7 (t, *J* = 22.5 Hz), 137.1, 135.8, 129.2 (2C), 128.5(2C), 128.0, 127.7, 110.4, 52.1, 44.8, 13.2.

HRMS (ESI-TOF): calc'd for $C_{19}H_{18}DN_2O_2^+$ [M+H$^+$] 308.1504, found 308.1498.

1,3-Dibenzyl-5-methyl-6-phenylpyrimidine-2,4(1*H*,3*H*)-dione (**20**)

Physical state: colorless oil;

Yield: 52%;

R_f = 0.5 (silica gel, PE: EtOAc = 5:1);

^{1}H NMR (400 MHz, $CDCl_3$): δ 7.59 (d, *J* = 7.3 Hz, 2H), 7.45 – 7.29 (m, 6H), 7.23 – 7.11 (m, 3H), 6.97 (d, *J* = 7.4 Hz, 2H), 6.82 – 6.80 (m, 2H), 5.27 (s, 2H), 4.82 (s, 2H), 1.65 (s, 3H).

^{13}C NMR (100 MHz, $CDCl_3$): δ 163.5, 152.3, 150.1, 137.3, 137.0, 132.6, 129.6, 129.5, 128.9, 128.5, 128.4, 127.8, 127.5, 126.8, 109.6, 49.8, 45.2, 12.9.

HRMS (ESI-TOF): calc'd for $C_{25}H_{23}N_2O_2^+$ [M+H$^+$] 383.1754, found 383.1750.

(*E*)-1,3-dibenzyl-5-methyl-6-styrylpyrimidine-2,4(1*H*,3*H*)-dione (**21**)

Physical state: colorless oil;

Yield: 47%;

R_f = 0.5 (silica gel, PE: EtOAc = 5:1);

^{1}H NMR (400 MHz, $CDCl_3$): δ 7.58 – 7.50 (m, 2H), 7.40 – 7.27 (m, 11H), 7.21 – 7.15 (m, 2H), 6.67 (d, *J* = 16.5 Hz, 1H), 6.49 (d, *J* = 16.5 Hz, 1H), 5.24 (s, 2H), 5.13 (s, 2H), 2.05 (s, 3H).

^{13}C NMR (100 MHz, $CDCl_3$): δ 163.7, 152.3, 147.6, 139.9, 137.3, 136.9, 134.9, 129.6, 129.3, 129.1(2C), 128.5, 127.8, 127.7, 127.1, 126.7, 118.5, 108.8, 49.6, 45.1, 13.5.

HRMS (ESI-TOF): calc'd for $C_{27}H_{25}N_2O_2^+$ [M+H$^+$] 409.1911, found 409.1902.

1,3-Dibenzyl-5-methyl-6-((triisopropylsilyl)ethynyl)pyrimidine-2,4(1*H*,3*H*)-dione (**23**)

Physical state: colorless oil;

Yield: 32%;

R_f = 0.4 (silica gel, PE: EtOAc = 10:1);

^{1}H NMR (400 MHz, $CDCl_3$): δ 7.49 – 7.42 (m, 2H), 7.33 – 7.21 (m, 8H), 5.29 (s, 2H), 5.15 (s, 2H), 2.17 (s, 3H), 1.06 – 0.98 (m, 21H).

^{13}C NMR (100 MHz, $CDCl_3$): δ 162.9, 151.4, 137.0, 136.4, 132.9, 129.2, 128.7, 128.5, 127.74, 127.71, 127.3, 115.8, 110.1, 96.5, 50.5, 45.0, 18.6, 13.9, 11.2.

HRMS (ESI-TOF): calc'd for $C_{30}H_{39}N_2O_2Si^+$ [M+H$^+$] 487.2775, found 487.2766.

1,3-Dibenzyl-6-methyl-5-((triisopropylsilyl)ethynyl)pyrimidine-2,4(1*H*,3*H*)-dione (**24**)
Physical state: colorless oil;
Yield: 39%;
R_f = 0.4 (silica gel, PE: EtOAc = 10:1);
^{1}H NMR (400 MHz, $CDCl_3$): δ 7.48 (d, *J* = 8.3 Hz, 2H), 7.38 – 7.24 (m, 6H), 7.14 (d, *J* = 7.3 Hz, 2H), 5.19 (s, 2H), 5.17 (s, 2H), 2.46 (s, 3H), 1.10 (s, 21H).
^{13}C NMR (100 MHz, $CDCl_3$): δ 160.8, 155.7, 151.6, 136.7, 135.7, 129.3, 129.1, 128.5, 128.1, 127.8, 126.3, 100.1, 99.0, 98.5, 49.1, 45.3, 18.8, 11.4.
HRMS (ESI-TOF): calc'd for $C_{30}H_{39}N_2O_2Si^+$ [M+H$^+$] 487.2775, found 487.2770.

Supplementary Note 17: *Ortho*-arylation of uracils
A 4 mL oven-dried Schlenk tube equipped with a magnetic stir bar was charged with alkenyl iodide **1m** (0.1 mmol, 1.0 equiv) and potassium carbonate (0.25 mmol, 2.5 equiv) and anhydrous DME (0.8 mL) in the glove box. Then 0.2 mL of DME solution with palladium acetate (0.001 mmol), norbornene derivatives **N**^{9} (0.05 mmol, 0.5 equiv), aryl bromide **2A** (0.15 mmol, 1.5 equiv) and olefin **3a** (0.15 mmol, 1.5 equiv) were added, and the mixture was heated to 105 °C and stirred for 24 h. After completion of the reaction (monitored by TLC), the mixture was cooled to r.t., filtered through a thin pad of Celite, eluting with EtOAc (10 mL), and the combined filtrate was concentrated *in vacuo*. The residue was directly purified by column chromatography on silica gel or purified by PTLC to give the desired product **22**.

Methyl (*E*)-2-(1,3-dibenzyl-2,4-dioxo-6-styryl-1,2,3,4-tetrahydropyrimidin-5-yl) benzoate (**22**)
Physical state: yellow oil;
Yield: 61%;
R_f = 0.3 (silica gel, PE: EtOAc = 5:1);

^{1}H NMR (400 MHz, $CDCl_3$): δ 7.93 (dd, *J* = 7.8, 1.4 Hz, 1H), 7.55 – 7.53 (m, 2H), 7.40 – 7.36 (m, 3H), 7.34-7.24 (m, 9H), 7.23-7.19 (m, 3H), 7.15 – 7.12 (m, 1H), 7.00 – 6.94 (m, 2H), 6.36 (s, 2H), 5.38 (d, *J* = 16.1 Hz, 1H), 5.28 (d, *J* = 13.7 Hz, 1H), 5.20 (d, *J* = 13.5 Hz, 1H), 5.01 (d, *J* = 16.0 Hz, 1H), 3.71 (s, 3H).
^{13}C NMR (100 MHz, $CDCl_3$): δ 167.8, 162.2, 152.2, 147.4, 141.5, 137.2, 136.9, 135.4, 135.1, 132.8, 132.1, 132.0, 130.3, 129.4, 129.2 (2C), 128.8, 128.5, 127.9 (2C), 127.6, 126.9, 126.5, 118.4, 115.1, 52.2, 49.7, 45.2.
HRMS (ESI-TOF): calc'd for $C_{34}H_{28}N_2NaO_4^+$ [M+Na$^+$] 551.1941, found 551.1947.

Supplementary Note 18: one-step formal synthesis of anti-HIV-1 agent **26**
A 4 mL oven-dried Schlenk tube equipped with a magnetic stir bar was charged with palladium acetate (0.01 mmol), norbornene derivatives **N^8** (0.1 mmol, 1.0 equiv), alkenyl iodide **1m** (0.1 mmol, 1.0 equiv) and potassium carbonate (0.25 mmol, 2.5 equiv) and anhydrous 1,4-dioxane (1 mL) in the glove box. Then BnCl **2h** (0.15 mmol, 1.5 equiv) and $MeB(OH)_2$ **3** (0.2 mmol, 2.0 equiv) were added, and the mixture was heated to 130 °C and stirred for 48 h. After completion of the reaction (monitored by TLC), the mixture was cooled to r.t., filtered through a thin pad of celite, eluting with EtOAc (10 mL), and the combined filtrate was concentrated *in vacuo*. The residue was directly purified by column chromatography on silica gel or purified by PTLC to give the desired product **25** (20.0 mg).

1,3,5-Tribenzyl-6-methylpyrimidine-2,4(1*H*,3*H*)-dione (**25**)[17]
Physical state: colorless oil;
Yield: 50%;
R_f = 0.4 (silica gel, PE: EtOAc = 4:1);
^{1}H NMR (400 MHz, $CDCl_3$): δ 7.50 (d, *J* = 7.0 Hz, 2H), 7.36 – 7.22 (m, 8H), 7.20 – 7.10 (m, 5H), 5.23 (s, 2H), 5.16 (s, 2H), 3.85 (s, 2H), 2.15 (s, 3H).
^{13}C NMR (100 MHz, $CDCl_3$): δ 163.0, 152.4, 148.9, 139.8, 137.3, 136.4, 129.2, 129.1, 128.7, 128.6, 128.1, 127.8, 127.7, 126.3, 126.1, 111.7, 48.5, 45.3, 31.8, 16.8.
HRMS (ESI-TOF): calc'd for $C_{26}H_{24}N_2NaO_2^+$ [M+Na$^+$] 419.1730, found 419.1732.

5. Supplementary References

[1] Yamamoto, Y., Murayama, T., Jiang, J., Yasui, T. & Shibuya, M. The vinylogous Catellani reaction: a combined computational and experimental study. *Chem. Sci.*, **9**, 1191–1199 (2018).

[2] Yamamoto, Y., Jiang, J. & Yasui, T. Palladium-catalyzed [3+2] and [2+2+2] annulations of 4-iodo-2-quinolones with activated alkynes through selective C−H activation. *Chem. A Eur. J.* **26**, 3749–3757 (2020).

[3] Maity, S., Das, D., Sarkar, S. & Samanta, R. Direct Pd(II)-catalyzed site-selective C5-arylation of 2-pyridone using aryl iodides. *Org. Lett.*, **20**, 5167–5171 (2018).

[4] Montgomery, J., Brown, M. & Reilly, U. Pyridone methylsulfone hydroxamate LpxC inhibitors for the treatment of serious gram-negative infections. *J. Med. Chem.* **55**, 1662–1670 (2012).

[5] Ziegler, D. S., Greiner, R., Lumpe, Kqiku, L., Karaghiosoff, K. & Knochel, P. Directed zincation or magnesiation of the 2-pyridone and 2,7-naphthyridone scaffold using TMP bases. *Org. Lett.* **19**, 5760–5763 (2017).

[6] Brooks, H., Crich, J., Henry, J., Hu, H., Jiang, D., Li, H.-Y., Maniar, S., McMillen, W., Sawyer, J., Slater, M. & Wang, Y. 2008**.** WO208144222A2.

[7] Li, H.-Y., Brooks, H., Crich, J., Henry, J., Slater, M. & Wang, Y. 2008. WO2008076704A1.

[8] Vulcano, R., Pengo, P., Velari, S., Wouters, J., De Vita, A. D, Tecilla, P. & Bonifazi, D. Toward fractioning of isomers through binding-induced acceleration of azobenzene switching. *J. Am. Chem. Soc.* **139**, 18271–18280 (2017).

[9] Prasad, B. & Knochel, P. Preparation and reactions of 2-zincated 2-cyclohexen-1-one and related heterocycles. *Tetrahedron* **53**, 16711–16720 (1997).

[10] Das, B. & Kundu, G. An efficient method for the iodination of C5-position of dialkoxy pyrimidines and uracil bases. *Synth. Commun.* **18**, 855–867 (1988).

[11] Sato, E., Ikeda, Y. & Kanaoka, Y. Photosensitized oxyganetion of 2-pyridones. *Heterocycles*, **25**, 65–68 (1987).

[12] Chavan, S. P., Pathak, A. B. & Kalkote, U. R. A practical formal synthesis of camptothecin. *Tetrahedron Lett.* **48**, 6561-6563 (2007).

[13] Lipshutz, B. H. & Amorelli, B. Total synthesis of Piericidin A1. Application of a modified Negishi carboalumination-nickel-catalyzed cross-coupling. *J. Am. Chem. Soc.*, **131**, 1396–1397 (2009).

[14] Xu, X.-H., Wang, X., Liu, G.-K., Tokunaga, E. & Shibata, N. Regioselective synthesis of heteroaryl triflones by LDA (Lithium diisopropylamide)-mediated anionic Thia-Fries rearrangement. *Org. Lett.* **14**, 2544–2547 (2012).

[15] Chen, J., Peng, H., He, J., Huan, X., Miao, Z. & Yang, C. Synthesis of isoquinolinone-based tricycles as novel poly(ADP-ribose) polymerase-1 (PARP-1) inhibitors. *Bioorg. & Med. Chem. Lett.* **24**, 2669–2673 (2014).

[16].Yamada, S. & Abe, M. Selective deprotection and amidation of 2-pyridyl esters via N-methylation. *Tetrahedron* **66**, 8667–8671 (2010).

[17] Chen, Y., Guo, Y., Yang, H. & Wang, Liu, J. Synthesis of l-(alkoxymethyl)-5-benzyl-6-methyluracil as potential nonnucleoside HIV-1 RT inhibitors. *Synth. Commun.* **36**, 2913–2920 (2006).
